# Organophosphorus zwitterions engaged in a conjugated macrocycle on fullerene

**DOI:** 10.1038/s42004-020-00340-x

**Published:** 2020-07-21

**Authors:** Yoshifumi Hashikawa, Shu Okamoto, Yasujiro Murata

**Affiliations:** Institute for Chemical Research, Kyoto University, Uji, Kyoto 611-0011 Japan

**Keywords:** Structure elucidation, Carbon nanotubes and fullerenes, Reaction mechanisms

## Abstract

Organophosphorus zwitterions are one of the most important but elusive intermediates for carbon–carbon bond formation in synthetic chemistry and biology. However, a lack of isolated examples due to their lability has hampered in-depth understanding of structures and their reaction mechanisms. In this study, we crystallographically reveal the solid-state structure of a phosha-Michael adduct engaged in a cage-opened C_60_ skeleton, which is formed as a kinetic product. This compound exhibits dark brown colour in solution with an intense absorption band that extends to 1000 nm, reflecting intramolecular charge transfer transitions. From the 1,2-dicarbonyl moiety on the conjugated orifice, β-oxo-phosphorus ylide is formed as a thermodynamic product. The reaction mechanism that has long been disputed is examined by experimental and theoretical studies, showing a pathway which includes an S_N_2 reaction as a key step instead of the hitherto considered carbene pathway.

## Abstract (web summary)

Organophosphorous zwitterions are important intermediates in organic synthesis but can be unstable and difficult to characterise. Here, two analogues of such intermediates stabilised by conjugation to an open-cage fullerene derivative allow for characterisation by single-crystal X-ray diffraction, with mechanistic implications.

## Introduction

Owing to large synthetic utility of phosphorus ylides demonstrated by Wittig in 1953^1,2^ as well as the discovery of the first naturally occurring compound bearing a C–P bond in 1959^3^, organophosphorus compounds have received growing attention^4^. Currently, their unique structural and electronic properties are often utilized in functionalized *π*-conjugated motifs^5^. Importantly, trivalent phosphorus compounds possess characteristics both of nucleophilicity and leaving-group ability, which allow them to proceed a variety of catalytic reactions commenced with their conjugate addition to α,β-unsaturated carbonyl compounds, leading to numerous biologically and synthetically valuable materials^6^. Even though zwitterionic forms of betaines have long been postulated as elusive key intermediates in such phospha-Michael addition^7^, their isolation, structural characterization, and mechanistic aspects have been still limited and remain formidable challenging issue^8,9^. Another long-felt but unsolved issue in phosphorous chemistry is a mechanism for forming β-oxo-phosphorous ylides from 1,2-dicarbonyl compounds, in which a carbene intermediate has been proposed to be formed from Kukhtin–Ramirez adduct without definitive evidence^10–12^.

Herein, we report isolation and crystallographic characterization of phosha-Michael adduct (1-phosphonium-5-oxabetaine) and β-oxo-phosphorus ylide embedded in a conjugated macrocyclic orifice on fullerene C_60_. The characteristic features of C_60_, i.e., excellent electron-accepting ability and *π*-conjugation along with the entire sphere, enabled the addition of phosphorus nucleophiles^13–15^ and stabilize reactive zwitterionic species. The mechanistic studies disclosed a rational pathway for forming β-oxo-phosphorus ylide, which is different from hitherto considered carbene pathway, as supported both experimentally and theoretically. These spherically *π*-conjugated zwitterions demonstrate significant change in electronic structures displaying an intense NIR absorption band up to 1000 nm with drastically elevated HOMO level.

## Results and discussion

### Synthesis

**Fig. 1:**
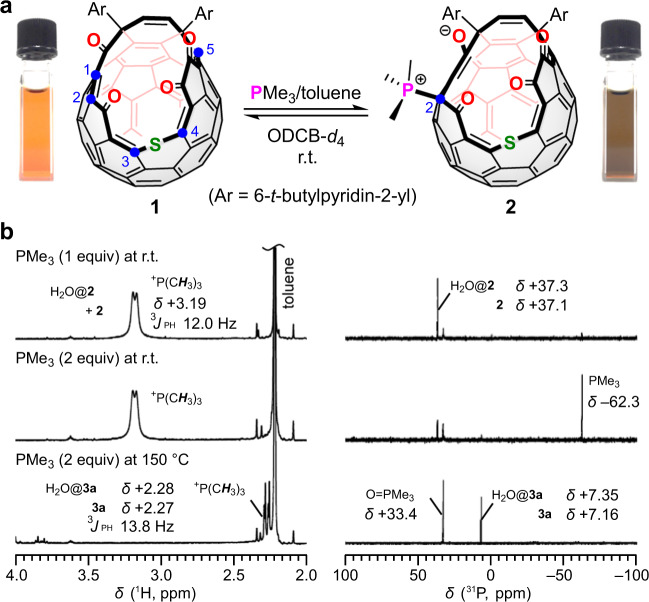
Synthesis of 1-phosphonium-5-oxabetaine. **a** Reaction of **1** with PMe_3_. **b** ^1^H (500 MHz) and ^31^P (202 MHz) NMR spectra (ODCB-*d*_4_).

As a conjugate macrocyclic orifice on the C_60_ cage, we made a choice of open-cage derivative **1** having α,β-unsaturated carbonyl and 1,2-dicarbonyl moieties (Fig. 1a). The reaction of **1** with the smallest alkyl phosphine (PMe_3_) was examined in *o*-dichlorobenzene-*d*_4_ (ODCB-*d*_4_) at room temperature. The addition of 1 equiv. PMe_3_ (1.0 M in toluene) to **1** in a sealed vessel caused the rapid change in color from reddish orange to dark brown. The APCI (atmospheric-pressure chemical ionization) mass spectrum of this solution clearly showed a molecular ion peak at *m*/*z* 1211.2073 ([**1** + PMe_3_ + H]^+^), which is identical to a betaine derivative. The NMR spectra showed the quantitative conversion of **1** into the sole product (**2**) bearing a PMe_3_ substituent with a broad doublet ^1^H signal (*δ* + 3.19 ppm, ^3^*J*_PH_ = 12.0 Hz) and a singlet ^31^P signal (*δ* + 37.1 ppm) (Fig. 1b). The ^31^P signal observed at *δ* + 37.3 ppm corresponds to H_2_O@**2** formed via spontaneous encapsulation of a water molecule. Once the reaction system was opened into air, the solution turned back to the original color of **1** within a few minutes. The structure of **2** was unambiguously determined by the single-crystal X-ray analysis (vide infra). This is the first example to characterize 1-phosphonium-5-oxabetaine engaged in a polycyclic aromatic compound. Among five possible sites C(1)–C(5) for the phosha-Michael addition (Fig. 1a), the reactions at C(1) and C(5) should be sterically prohibited due to the bulky aryl substituents. According to DFT calculations at the M06-2X/6-31 G(d,p) level of theory in which *t*-butyl groups were replaced with hydrogen atoms, the energy required for forming a betaine derivative is rather small for **2′** (Ar = 2-pyridyl) obtained by the reaction at C(2) (Δ*G*^‡^ + 11.9 kcal/mol at 298 K) compared to those at C(3) and C(4) (Δ*G*^‡^ + 21.0 and +21.9 kcal/mol, respectively) (Supplementary Figs. 38, 39).

Notably, further addition of PMe_3_ resulted in less considerable NMR spectral change, demonstrating that **1** does not undergo any multiple addition of phosphine (Fig. 1b). However, by heating the 1:2 reaction mixture at 150 °C for 15 min, the solution color changed to greenish brown with the complete consumption of 2 equiv. PMe_3_, affording O=PMe_3_ and a new compound bearing a PMe_3_ substituent (**3a**) in a 1:1 ratio (Fig. 1b). The ^13^C NMR spectrum of **3a** (CDCl_3_, 126 MHz) showed a new doublet signal corresponding to a fullerenyl *sp*^3^-carbon atom directly bound to a phosphorus atom (*δ* + 75.7 ppm, ^1^*J*_CP_ 122.6 Hz) together with three carbonyl carbon signals including a doublet one at *δ* + 188.63 ppm weakly coupled with ^31^P (^2^*J*_CP_ = 13.2 Hz). These data convinced us of the structure for **3a** to be a β-oxo-phosphorus ylide^16,17^ formed by the reaction at the 1,2-dicarbonyl moiety in **1**.

Subsequently, we performed the reaction using several phosphines, which gave **3a**–**e** in moderate to high yields (Table 1). Increased concentration of **1** was quite effective for lower-nucleophilic phosphines^18^, PPh_3_ and dppf. Contrary to **3b**–**e** bearing a PR_3_ substituent with a cone angle^19^ exceeding 140° (PCy_3_: 170°, P(NMe_2_)_3_: 157°, and PPh_3_: 145°), **3a** (PMe_3_: 118°) was significantly transformed into methylene derivative **4** during the reaction and the workup process. The hydrolysis^20^ of **3a** in ODCB at 150 °C for 1 day resulted in quantitative conversion into **4**, whereas **3d** was not considerably hydrolyzed (13%) even after 6 days. These results are indicative of the steric protection for phosphorus centers in **3b**–**e**.

**Table 1:** Synthesis of β-oxo-phosphorus ylides **3a**–**e**.


Entry	PR_3_ (equiv.)		Temp. (°C)	Time (h)	3 (%)	4 (%)
1^a^	PMe_3_^c^	(10)	60	1	44 (**3a**)	42
2^b^	PCy_3_	(10)	150	0.25	48 (**3b**)	8
3^a^	P(NMe_3_)_2_	(10)	150	4	82 (**3c**)	—
4^b^	PPh_3_	(20)	150	4	72 (**3d**)	3
5^b^	dppf^d^	(10)	150	5	49 (**3e**)	12

^a^**1**: ca. 9 mM.

^b^**1**: ca. 30 mM.

^c^1.0 M in toluene.

^d^dppf: 1,1′-bis(diphenylphosphino)ferrocene.

### Crystallography

**Fig. 2:**
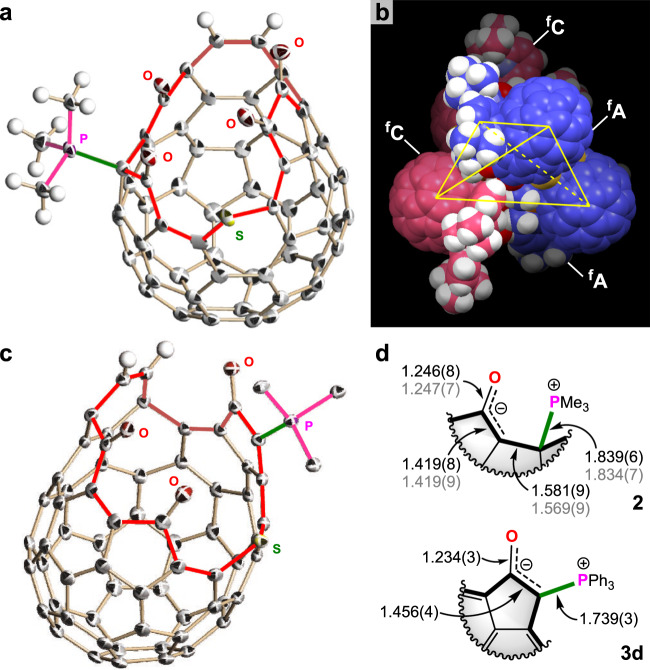
Single-crystal X-ray structures. **a** Structure of ^f^A-**2**. **b** Packing mode of **2**. **c** Structure of **3d**. **d** Selected bond lengths (units in Å). Thermal ellipsoids are shown in 50% probability. The encapsulated molecules, solvent molecules, aryl groups, and another independent molecule (^f^C-**2**) are omitted for clarity. The bond lengths shown with gray color belong to ^f^C-**2**.

To our delight, the black platelet single crystals of **2** were obtained by slow diffusion of PMe_3_/toluene to a CHCl_3_ solution of **1** under argon atmosphere at 5 °C. The X-ray diffraction analysis revealed that an asymmetric unit accommodates a racemate including two enantiomeric isomers ^f^C-**2** and ^f^A-**2** arranged as two crystallographically independent molecules (nomenclature based on criteria stated by Diederich^21,22^). Interestingly, a unit of the racemate is assembled with another one as a tetrahedral configuration via heterochiral recognition (Fig. 2a, b). The driving force for this self-assembly should be attributed to the intermolecular electrostatic attraction between ^+^PMe_3_ and C–O^−^ moieties (3.646(4) and 3.668(4) Å for P•••O) and thus the reactive sites (phosphonium betaine) were fully covered with the four fullerene cages in total. From the bond lengths, the structure of **2** can be rationalized by describing a resonance hybrid consisting of 1-phosphonium-5-oxabetaine and 1-phosphonium-3-carbabetaine as major contributing forms (Fig. 2d). For β-oxo-phosphorus ylide **3d**, the black block-shaped single crystals were grown from a CH_2_Cl_2_/hexane solution. As shown in Fig. 2c, the phosphorus ylide was formed at the 1,2-dicarbonyl moiety in **1**. The bond length of the C–P bond in **3d** is 1.739(3) Å, which is explicitly longer than that for PPh_3_P=CH_2_ (1.66 Å)^23^. This is suggestive of the charge delocalization along with C–C–O and other C_60_ moieties (Fig. 2d), being consistent with the natural population and Wiberg bond order analyses (Supplementary Note 3).

### Mechanism

**Fig. 3:**
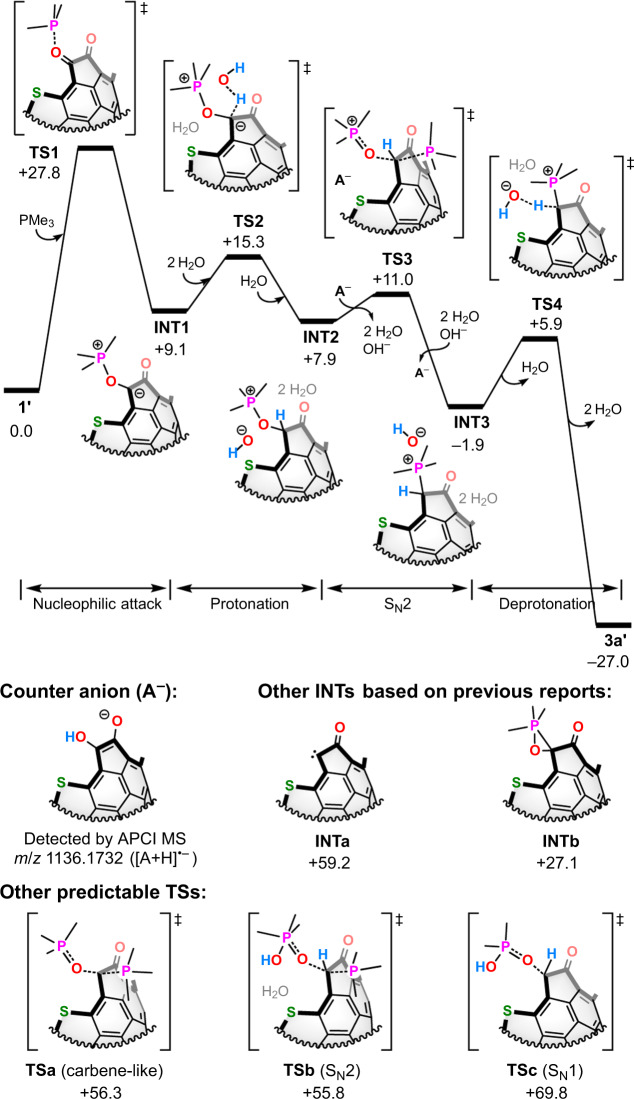
Plausible mechanism. The Δ*G* (kcal/mol at 298 K) values were calculated at the M06-2X/6-31 G(d,p) level of theory.

To verify the possible pathway for providing **3a** from **1** and PMe_3_, we performed DFT calculations (M06-2X/6-31 G(d,p), 298 K) (Fig. 3; Supplementary Notes 1 and 2). The first step is the nucleophilic attack of PMe_3_ to the carbonyl group in **1′** (Ar = 2-pyridyl) to give **INT1** via **TS1** (Δ*G*^‡^ + 27.8 kcal/mol), which is slightly lower than that of the phospha-Brook rearrangement (+28.5 kcal/mol)^24^. Even though the formation of carbene intermediates has been addressed as the second step^25^, the calculated energy of **INTa** (+59.2 kcal/mol) obviously excludes such possibility as well as pentavalent phosphorus intermediate **INTb** (+27.1 kcal/mol)^26,27^. This results are in line with our experimental observation that carbene scavengers, e.g., terminal olefins, could not trap any species. Since the hydrolysis of an **INT1**-type intermediate has been reported by Gan and co-workers^28^, we computed the process toward **INT2**, showing Δ*G*^‡^ + 15.3 kcal/mol (**TS2**). The further transformation into **INT3** in an S_N_2 fashion^29^ with the loss of O=PMe_3_ needs Δ*G*^‡^ + 11.0 kcal/mol (**TS3**), which is substantially lower than alternative pathways **TSa**–**c**. In this step, we adopted **A**^−^ as a possible counter anion^30,31^, which was observed in the crude mixture of our reactions (Table 1, entry 1) by APCI MS. Finally, deprotonation of **INT3** proceeds to furnish desired **3a′** via **TS4** (Δ*G*^‡^ + 5.9 kcal/mol).

### Electronic properties

**Fig. 4:**
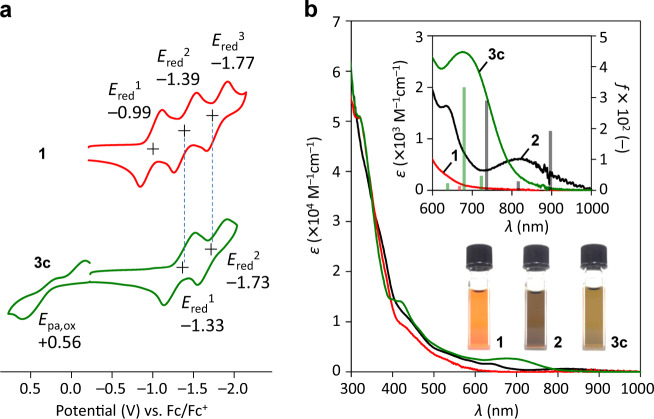
Electrochemical and absorption properties. **a** Cyclic voltammograms of **1** and **3c** (1 mM in ODCB, 0.10 M *n*-Bu_4_N ∙ BF_4_, 100 mV/s). **b** UV–vis-NIR absorption spectra of **1**, **2**, and **3c** (50 μM in toluene). The inset shows an expanded view of long wavelength region with calculated oscillator strengths, which were plotted at corresponding transition energies calibrated by a factor of 0.72^32^ (TD CAM-B3LYP/6-31G(d)//B3LYP/6-31G(d)).

The cyclic voltammogram of **3c** showed the reversible first and second reduction waves at *E*_red_ –1.33 and –1.73 V, which are close to the second and third ones for **1** at *E*_red_ –1.39 and –1.77 V, respectively (Fig. 4a). Thus, **3c** has the electron configuration similar to the monoanionic state of **1**, further suggesting that the anionic charge, which stems from the C^–^–P^+^ bond, is effectively delocalized on the C_60_ skeleton. Since the HOMO is mainly localized on the C^–^–P^+^ bond (Supplementary Fig. 53), a single electron oxidation of **3c** at *E*_pa,ox_ + 0.56 V is supposed to generate a radical cation with a C^•^–P^+^ character. As shown in Figs. 2 and 3c, **4b** exhibited intense absorption bands at NIR region, which tail to 1000 and 900 nm, respectively. Whereas the HOMO and LUMO of **1** are delocalized over the entire C_60_ skeleton, **2** and **3c** show the HOMO localized at the betaine and ylide moieties, respectively. Thereby, intramolecular charge transfer transitions (HOMO → LUMO and HOMO → LUMO + 1) with large oscillator strengths (*f* = 0.0192 at 897 nm for **2** and *f* = 0.0334 at 680 nm for **3c**) should contribute to the observed intense absorption with remarkable bathochromic shift up to 300 nm relative to **1** (TD CAM-B3LYP/6-31G(d)//B3LYP/6-31G(d)) (Supplementary Note 4).

In summary, by the reaction of phosphines with a conjugated macrocycle on **1**, betaine and ylide derivatives were found to be formed as kinetic and thermodynamic products, respectively. The first phospha-Michael adduct engaged in a polycyclic aromatic compound (**2**) showed a self-assembled structure in the solid state. The rational reaction mechanism was proposed for forming ylide **3** from 1,2-dicarbonyl compound **1** based on experimental and computational studies, suggestive of the S_N_2 reaction as a key step instead of hitherto considered carbene pathway^25^. Importantly, the introduction of the PR_3_ moiety drastically changes the properties of the *π*-skeleton, resulting in intense NIR absorption with elevated HOMO level. These findings are expected to promote further explorations of phosphorus-bearing *π*-conjugated materials.

## Methods

### General

All reactions were carried out under Ar atmosphere. Unless otherwise noted, materials purchased from commercial suppliers were used without further purification. Note that all the compounds shown herein encapsulate a water molecule inside their cages with an occupation level of 10–70% at room temperature. For the simplification, the encapsulated water molecules are omitted for clarity in chemical structures for all figures. Compound **1** was synthesized according to the literature^33^. The detailed procedures and the data required for characterization are provided in the Supplementary Methods.

### Synthesis of 2

Compound **1** (10.0 mg) was placed into a Schlenk-type NMR tube and degassed through three vacuum-Ar cycles. Distilled ODCB-*d*_4_ (0.650 mL, 13.6 mM) and then PMe_3_ (1.0 M in toluene, 8.80 μL, 1.0 equiv.) were added at room temperature. The quantitative conversion into **2** was confirmed by NMR and mass analyses.

### Synthesis of 3

The typical synthetic procedure of **3a** was shown. Other β-oxo-phosphorus ylides **3b**–**e** were synthesized by the procedure shown in the Supplementary Information. Compound **1** was placed into a Schlenk tube and degassed through three vacuum-Ar cycles. ODCB (1.0 mL, 8.8 mM) and then PMe_3_ (1.0 M in toluene, 88.1 μL, 10 equiv.) were added to the tube. The resulting mixture was heated at 60 °C for 1 h. After reaction, residual PMe_3_ and its oxide were removed under reduced pressure. The chromatographic purification by silica gel (CS_2_/EtOAc (10:1 to 4:1)) gave **3a** (4.63 mg) in 44% isolated yield as black powder.

### Preparation of single crystals

Since **2** is moisture- and air-sensitive, the crystals were grown under inert conditions. A micro tube containing **1** (ca. 0.5 mg) was capped with a rubber septum and degassed through vacuum-Ar cycles. After ca. 0.2 mL of CHCl_3_ was added to dissolve **1**, toluene was slowly added to make a thin-layer. To the tube, an excessive amount of PMe_3_ in toluene was added in one portion at room temperature. The resulting solution was kept at 5 °C overnight. The black platelet single crystals were formed onto the bottom surface. The single crystals of **3d** were obtained from a CH_2_Cl_2_/hexane solution by slow evaporation at room temperature.

### Electrochemical analysis

Cyclic voltammetry was conducted using a three-electrode cell with a glassy carbon working electrode, a platinum wire counter electrode, and an Ag/AgNO_3_ reference electrode. The measurements were carried out under N_2_ atmosphere using ODCB solutions of 1.0 mM samples and 0.10 M tetrabutylammonium tetrafluoroborate (*n*-Bu_4_N·BF_4_) as a supporting electrolyte. The redox potentials were calibrated with ferrocene used as an internal standard, which was added after each measurement.

## Supplementary information

Peer Review File

Supplementary Information

Description of Additional Supplementary Files

Supplementary Data 1

Supplementary Data 2

## Data Availability

Detailed experimental procedures, characterization data (Supplementary Figs. 1–37), and computational results (Supplementary Figs. 38–53 and Supplementary Tables 1–59) are provided in the Supplementary Information. CCDC 1988945 (**2**) and 1988944 (**3d**) contain the supplementary crystallographic data for this paper (Supplementary Data 1 and 2). These data are provided free of charge by The Cambridge Crystallographic Data Centre.
