# Supplementary material for: Organophosphorus zwitterions engaged in a conjugated macrocycle on fullerene
Source: Commun Chem. 2020 Jul 21;3:90. doi: 10.1038/s42004-020-00340-x (PMC9814461; doi:10.1038/s42004-020-00340-x)
Supplement: Supplementary file 2 — Supplementary Information [file 42004_2020_340_MOESM2_ESM.pdf]

Supplementary Information

Organophosphorus Zwitterions Engaged in a Conjugated Macrocycle on Fullerene

Yoshifumi Hashikawa, Shu Okamoto, and Yasujiro Murata*

Institute for Chemical Research, Kyoto University, Uji, Kyoto 611-0011, Japan
Fax: (+81)774-38-3178
E-mail: yasujiro@scl.kyoto-u.ac.jp

Contents

Supplementary Methods
1. General 4
2. Computational Methods 5
3. Synthesis of 1-Phophonium-5-oxabetaine (**2**) 6
4. Synthesis of β-Oxo-phosphorus Ylides (**3**) 9
 4.1. Synthesis of **3a** by the Reaction with PMe_3 9
 4.2. Synthesis of **3b** by the Reaction with PCy_3 13
 4.3. Synthesis of **3c** by the Reaction with $P(NMe_2)_3$ 16
 4.4. Synthesis of **3d** by the Reaction with PPh_3 19
 4.5. Synthesis of **3e** by the Reaction with dppf 22
5. Synthesis of Methylene Derivative (**4**) 26
6. UV-Vis-NIR Absorption Spectra 30
7. Cyclic Voltammograms 32
8. IR Spectra 33
9. Single Crystal X-Ray Structures 34
 9.1. Crystal Structure of $[(H_2O)_{0.60(4)}(N_2)_{0.24(3)}$@**2**]•$[(H_2O)_{0.60(4)}(N_2)_{0.21(3)}$@**2**] •$(C_6H_5CH_3)_{6.11}$•$(CHCl_3)_{1.07}$ 34
 9.2. Crystal Structure of (**3d**)•$(3CH_2Cl_2)_{0.4}$•$(hexane)_{0.6}$ 36

Supplementary Note 1
10. Formation Mechanism of 1,5-Betaines (**2'**) 38

Supplementary Note 2
11. Formation Mechanism of β-Oxo-phosphorus Ylide (**3a'**) 52
 11.1. Previously Proposed Intermediates (**INTa** and **INTb**) 52
 11.2. Formation of 1,3-Betaines (**INT1**) 58
 11.3. Phospha-Brook Rearrangement 71
 11.4. Kukhtin-Ramirez Intermediates 76
 11.5. Hydrolysis of **INT1** 78
 11.6. P–C Bond Formation from **INT1** via a Concerted Pathway 88
 11.7. P–C Bond Formation from **INT2-^{V}P** in an S_N1 or S_N2 Fashion 93
 11.8. P–C Bond Formation from **INT2-(2H$_2$O)**/OH^- in an S_N2 Fashion 97
 11.9. P–C Bond Formation from **INT2-(2H$_2$O)/A$^-$** in an S_N2 Fashion 101

11.10. Formation of **3a'** by the Deprotonation of **INT3** 115

Supplementary Note 3
12. NBO Analyses of **2** and **3d** 117

Supplementary Note 4
13. TD-DFT Calculations 125

Supplementary References
14. References 129

Supplementary Methods

1. General

The ^{1}H, ^{13}C, and ^{31}P NMR measurements were carried out at room temperature (unless otherwise noted) with JEOL JNM ECA500 instruments. The NMR chemical shifts were reported in ppm with reference to residual protons and carbons of $CDCl_3$ (δ 7.26 ppm in ^{1}H NMR, δ 77.00 ppm in ^{13}C NMR), CD_2Cl_2 (δ 5.32 ppm in ^{1}H NMR, δ 53.80 ppm in ^{13}C NMR), benzene-d_6 (δ 7.15 ppm in ^{1}H NMR), and *o*-dichlorobenzene-d_4 (ODCB-d_4) (δ 7.20 ppm in ^{1}H NMR). The ^{31}P NMR chemical shifts were reported in ppm with reference to the chemical shift of H_3PO_4 (δ 0.00 ppm) in a glass sealed capillary insert inside the NMR tube filled with D_2O. APCI (atmospheric pressure chemical ionization) mass spectra were measured on a Bruker micrOTOF-Q II. UV-vis absorption spectra were measured with a Shimadzu UV-3150 spectrometer. IR spectra were taken with a Shimadzu IR-Affinity 1S. Cyclic voltammetry was conducted on a BAS Electrochemical Analyzer ALS620C using a three-electrode cell with a glassy carbon working electrode, a platinum wire counter electrode, and a $Ag/AgNO_3$ reference electrode. The measurements were carried out under N_2 atmosphere using ODCB solutions of 1.0 mM samples and 0.10 M tetrabutylammonium tetrafluoroborate (n-$Bu_4N{\cdot}BF_4$) as a supporting electrolyte. The redox potentials were calibrated with ferrocene used as an internal standard which was added after each measurement. The high-performance liquid chromatography (HPLC) was performed with the use of a Cosmosil Buckyprep column (250 mm in length, 4.6 mm in inner diameter) for analytical purpose and the same columns (250 mm in length, 20 mm in inner diameter) for preparative purpose. Thin layer chromatography (TLC) was performed on glass plates coated with 0.25 mm thick silica gel 60F-254 (Merck). Column chromatography was performed using PSQ 60B or 100B (Fuji Silysia).

Fullerene C_{60} was purchased from SES Research Co. Toluene was purchased from Kanto Chemical Co., Inc. Ethyl acetate and toluene (SPR grade) were purchased from Nacalai Tesque, Inc. Trimethylphosphine (1.0 M toluene solution) and ODCB (SPR grade) were purchased from Sigma-Aldrich Co. LLC. Triphenylphosphine and 1,1'-bis(diphenylphosphino)ferrocene (dppf) were purchased from Tokyo Chemical Industry Co. Ltd. H_3PO_4, carbon disulfide, acetonitrile, $CHCl_3$ (super dehydrated), and tris(dimethylamino)phosphine were purchased from FUJIFILM Wako Pure Chemical Corporation. Tricyclohexylphosphine was purchased from Strem Chemicals Inc. Open-cage tetraketo C_{60} derivative **1** was synthesized according to a literature[1].

All reactions were carried out under Ar atmosphere. Unless otherwise noted, materials purchased from commercial suppliers were used without further purification. Note that all the compounds shown herein spontaneously encapsulate a water molecule inside their cages with an occupation level of 10–70% at room temperature. For the simplification, the encapsulated water molecules are omitted for clarity in chemical structures shown in all schemes and figures.

2. Computational Methods

All calculations were conducted with the Gaussian 09 program package. For the mechanistic studies, all structures at stationary and transition states were optimized at the M06-2X/6-31G(d,p) level of theory. All structures were confirmed by the frequency analyses at the same level of theory. The single point calculations for the natural population and natural bond orbital analyses were conducted at the M06-2X/6-31G(d,p) level of theory. Using geometries optimized at the B3LYP/6-31G(d) level of theory, TD DFT calculations were carried out at the CAM-B3LYP/6-31G(d) level of theory.

3. Synthesis of 1-Phophonium-5-oxabetaine (2)

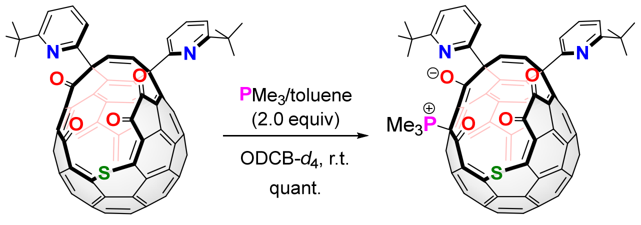

Compound **1** (10.0 mg, 8.81 μmol) was placed into a Schlenk-type NMR tube and degassed through three vacuum-Ar cycles. *o*-Dichlorobenzene-*d*4 (0.650 mL, 13.6 mM) and then PMe_3 (1.0 M in toluene, 8.80 μL, 8.8 μmol, 1.0 equiv) were added into the tube at room temperature. The solution color turned to dark brown from reddish orange immediately after addition of PMe_3. The quantitative conversion of **1** into **2** was confirmed by NMR and APCI mass analyses.

H_2O@**2** (H_2O: 14%): ^{1}H NMR (500 MHz, ODCB-d_4) δ 8.21 (br s, 1H), 7.85 (br s, 1H), 7.68 (br s, 1H), 6.20 (br s, 1H), 3.19 (br d, $^2J_{HP}$ = 12 Hz, 9H), 1.34 (s, 9H), 1.17 (s, 9H), –9.98 (br s, 0.28H) (Four signals corresponding to aryl groups are overlapped with a solvent peak.); ^{31}P NMR (202 MHz, ODCB-d_4) δ 37.3 (H_2O@**2**), 37.1 (**2**). HRMS (APCI, positive ion mode) calcd for $C_{85}H_{36}N_2O_4PS$ ([**2**+H]$^+$) 1211.2128, found 1211.2073.

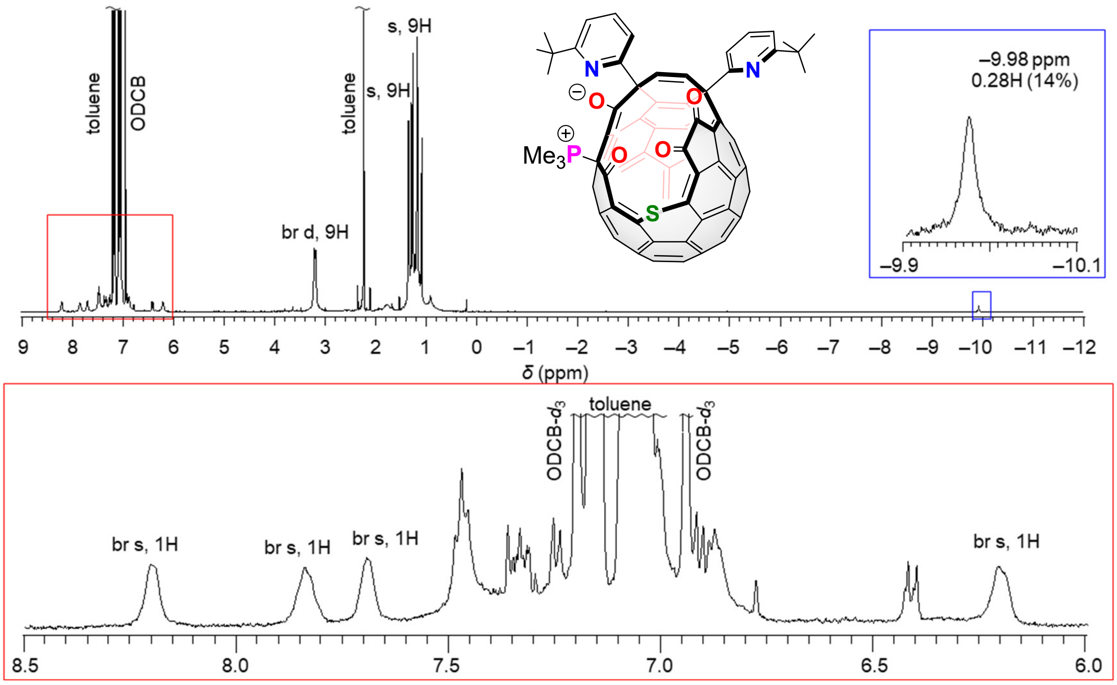

Supplementary Fig. 1. ^{1}H NMR spectra (500 MHz, ODCB-d_4) of **2**.

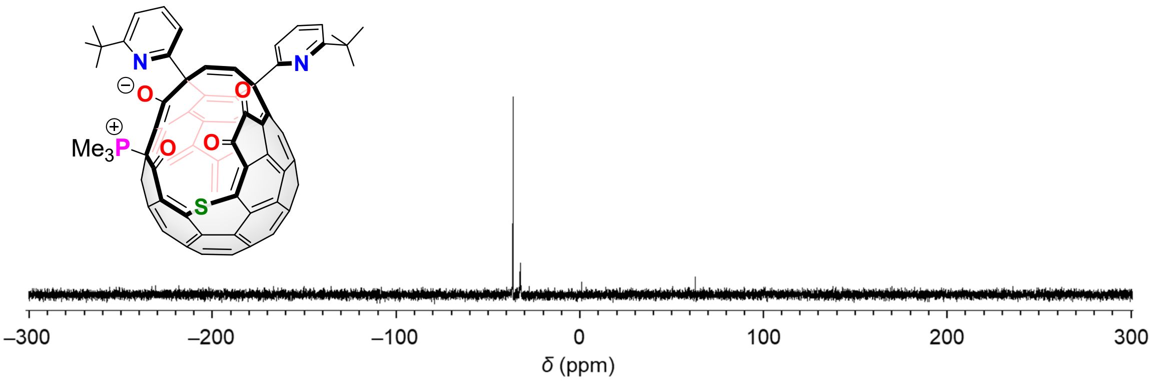

Supplementary Fig. 2. ^{31}P NMR spectrum (202 MHz, ODCB-d_4) of **2**.

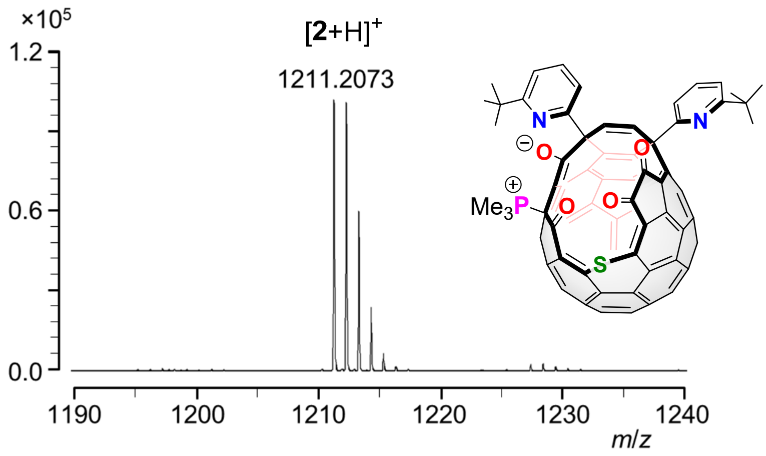

Supplementary Fig. 3. APCI mass spectrum (positive ion mode) of **2**.

4. Synthesis of β-Oxo-phosporus Ylides (3)

4.1. Synthesis of 3a by the Reaction with PMe_3

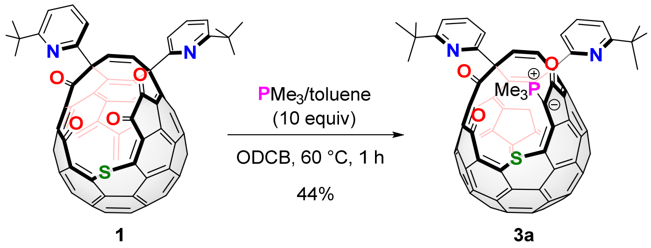

1 (10.0 mg, 8.83 μmol) was placed into a Schlenk tube and degassed through three vacuum-Ar cycles. *o*-Dichlorobenzene (1.0 mL, 8.8 mM) and then trimethylphosphine (1.0 M in toluene, 88.1 μL, 88 μmol, 10 equiv) were added to the tube. The resulting mixture was heated at 60 °C for 1 h. After reaction, residual PMe_3 and its oxide were removed under reduced pressure. The chromatographic purification by silica gel (CS_2/AcOEt (10:1 to 4:1)) gave **3a** (4.63 mg, 3.87 μmol) in 44% isolated yield as black powder.

H_2O@**3a** (H_2O: 70%): ^{1}H NMR (500 MHz, $CDCl_3$) δ 7.55 (t, J = 7.8 Hz, 1H), 7.41 (t, J = 7.9 Hz, 1H), 7.36 (d, J = 7.8 Hz, 1H), 7.22–7.24 (m, 2H), 7.14 (d, J = 7.8 Hz, 1H), 7.04 (d, J = 7.9 Hz, 1H), 6.49 (d, J = 10.3 Hz, 1H), 2.46 (d, $^2J_{HP}$ = 13.7 Hz, 9H), 1.15 (s, 9H), 1.09 (s, 9H), –10.94 (s, 1.39H); ^{13}C NMR (126 MHz, $CDCl_3$) δ 192.49, 192.38, 188.77 (d, $^2J_{CP}$ = 13.2 Hz), 188.63 (d, $^2J_{CP}$ = 13.2 Hz), 184.70, 167.97, 167.94, 167.86, 167.81, 164.23, 164.17, 164.12, 164.05, 153.28, 153.23, 153.18, 151.62, 151.57, 151.52, 151.16, 150.94, 150.86, 150.62, 150.55, 150.45, 150.33, 150.24, 150.13, 150.03, 149.96, 149.75, 149.67, 149.41, 149.36, 149.19, 148.95, 148.81, 148.74, 148.41, 148.17, 147.71, 147.42, 147.39, 145.68, 145.64, 145.42, 145.28, 145.26, 145.17, 144.91, 144.76, 144.69, 144.64, 144.48, 144.45, 144.33, 144.04, 143.95, 143.79, 143.74, 143.55, 143.50, 143.22, 143.04, 142.91, 142.82, 142.72, 142.63, 141.65, 141.55, 141.41, 141.17, 140.91, 140.81, 140.60, 140.52, 140.41, 140.34, 140.30, 140.22, 140.08, 139.88, 139.73, 138.72, 138.19, 138.15, 137.71, 137.43, 137.31, 137.01, 136.80, 136.71, 136.69, 136.56, 136.47, 136.40, 136.15, 136.11, 135.52, 135.22, 135.06, 134.19, 132.73, 132.70, 132.63, 132.46, 132.42, 132.21, 132.13, 132.06, 131.93, 131.58, 131.29, 130.81, 130.45, 126.31, 126.23, 124.76, 124.68, 122.60, 122.48, 120.61, 120.28, 120.23, 119.90, 119.87, 116.83, 116.79, 116.61, 116.57, 75.72 (d, $^1J_{CP}$ = 122.2 Hz), 58.34, 58.26, 54.34, 54.27, 37.53, 29.86, 13.99 (d, $^1J_{CP}$ = 61.2 Hz). (The carbon signals of H_2O@**3a** and **3a** were partly distinguishable. The sum of carbon signals for each molecule must be 79 in theory. Observed 138 out of 158. Three

signals corresponding to C=O and C–P with 7 sp^3 signals are overlapped at the same positions, respectively, while 11 sp^2 signals are overlapped in the aromatic region.); ^{31}P NMR (202 MHz, $CDCl_3$) δ 7.16 (H_2O@**3a**); HRMS (APCI, negative ion mode) calcd for $C_{85}H_{35}N_2O_3PS$ (**3a**$^{\bullet-}$) 1194.2111, found 1194.2130.

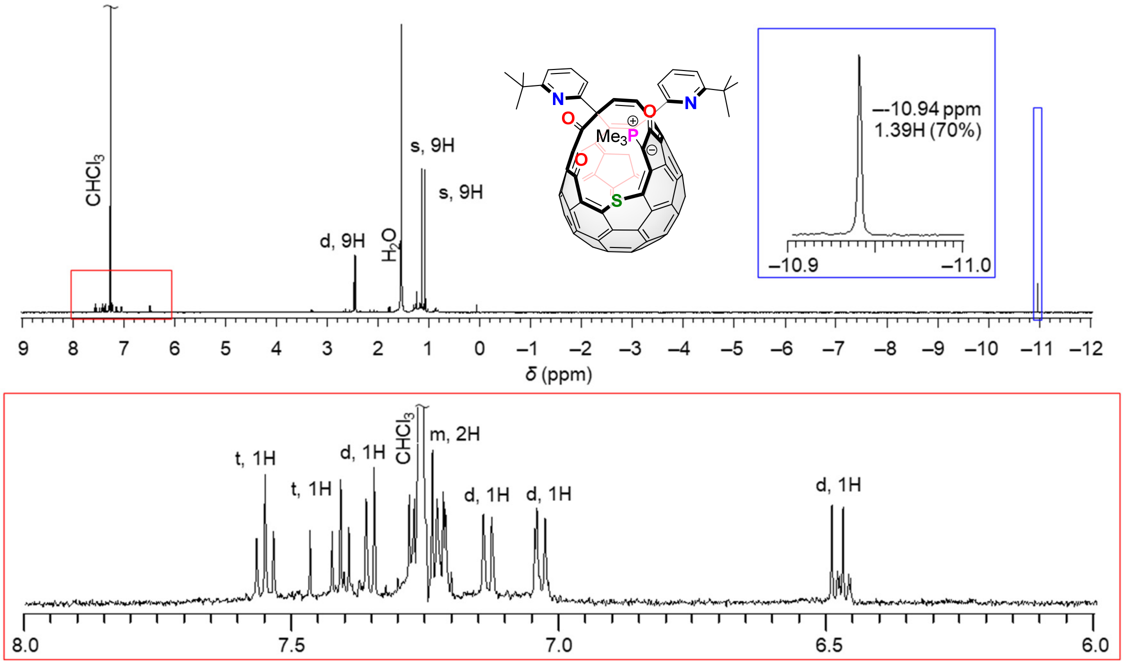

Supplementary Fig. 4. ^{1}H NMR spectra (500 MHz, $CDCl_3$) of **3a**.

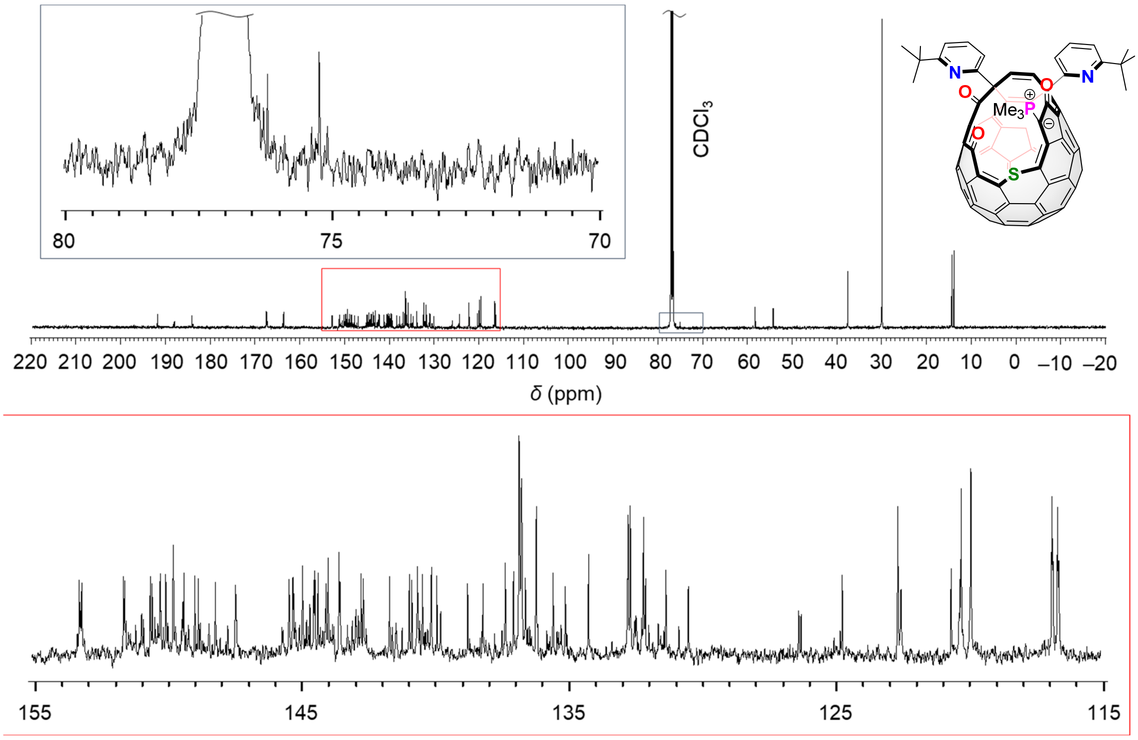

Supplementary Fig. 5. ^{13}C NMR spectra (126 MHz, $CDCl_3$) of **3a**.

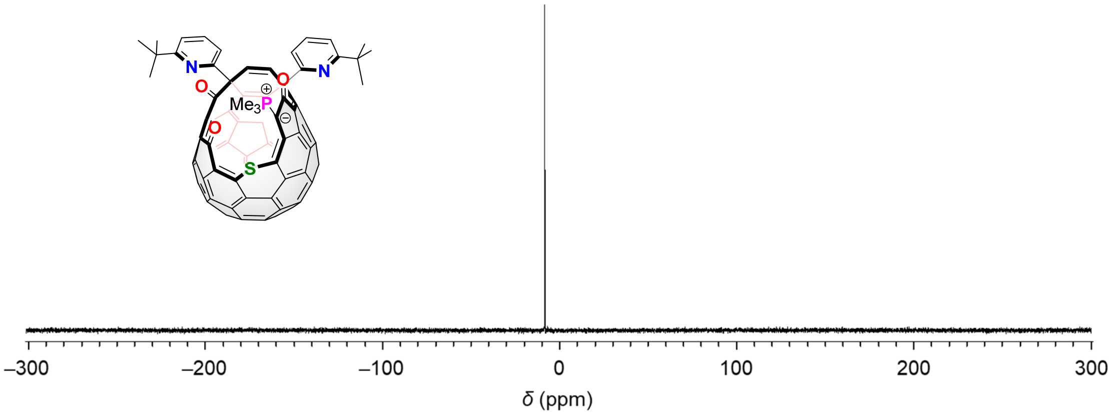

Supplementary Fig. 6. ^{31}P NMR spectrum (202 MHz, $CDCl_3$) of **3a**.

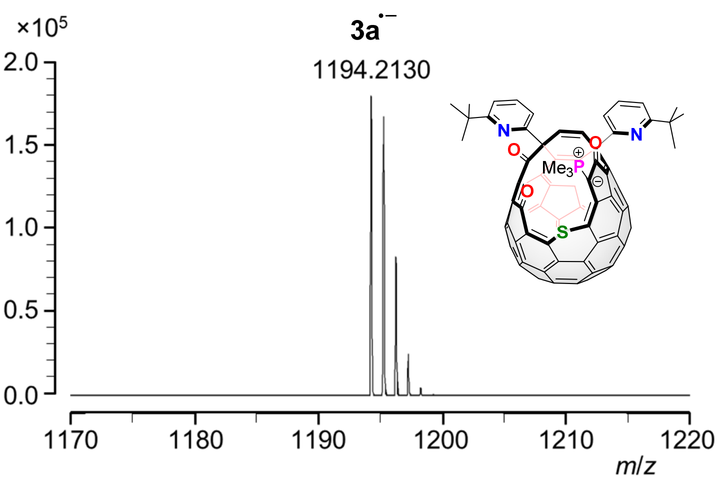

Supplementary Fig. 7. APCI mass spectrum (negative ion mode) of **3a**.

4.2. Synthesis of 3b by the Reaction with PCy_3

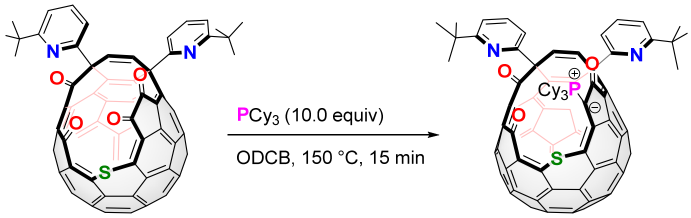

Compound **1** (10.0 mg, 8.84 μmol) was placed into a Schlenk tube and degassed through three vacuum-Ar cycles. Tricyclohexylphosphine (352 mM in ODCB, 0.250 mL, 88.0 μmol, 10.0 equiv) was added to the tube. The resulting solution was heated at 150 °C for 15 min. After reaction, ODCB was removed under reduced pressure. The crude mixture was purified by HPLC (Buckyprep column, 7.5 mL/min, toluene) to give **3b** as a mixture with O=PCy_3. The reprecipitation from its CS_2 solution by addition of CH_3CN gave pure **3b** (5.99 mg, 4.28 μmol) in 48% yield as black powder.

H_2O@**3b** (H_2O: 51%): ^{1}H NMR (500 MHz, benzene-d_6, 70 °C) δ 7.95–7.99 (m, 1H), 7.35–7.39 (m, 2H), 7.11 (d, J = 7.9 Hz, 1H), 7.05–7.08 (m, 1H), 6.85 (d, J = 10.3 Hz, 1H), 6.82 (d, J = 7.3 Hz, 1H), 6.74 (d, J = 7.9 Hz, 1H), 3.21 (br quartet, J = 12.4 Hz, 3H), 2.30 (br s, 6H), 2.18 (br s, 3H), 2.04 (br s, 3H), 1.83–1.92 (br m, 6H), 1.75 (br s, 3H), 1.55–1.59 (br m, 6H), 1.44–1.49 (br s, 3H), 1.16 (s, 9H), 1.10 (s, 9H), –10.77 (s, 1.02H); ^{13}C NMR (126 MHz, $CDCl_3$) δ 191.97, 189.29 (d, $^2J_{CP}$ = 9.6 Hz), 184.18, 168.03, 167.92, 163.88, 153.50, 153.33, 153.27, 153.02, 151.98, 151.71, 150.75, 150.65, 150.22, 150.10, 150.01, 149.74, 149.40, 149.15, 148.86, 148.27, 147.30, 145.57, 145.17, 144.97, 144.92, 144.46, 143.97, 143.89, 143.78, 143.74, 143.61, 143.32, 143.22, 143.13, 142.14, 141.54, 140.91, 140.62, 140.34, 140.01, 139.91, 139.78, 138.55, 137.92, 137.24, 136.93, 136.72, 136.62, 136.45, 136.06, 135.60, 135.49, 133.81, 132.96, 132.59, 132.53, 131.84, 131.07, 130.56, 130.36, 128.31, 128.25, 124.75, 122.05, 121.73, 120.08, 119.81, 116.77, 116.51, 72.12 (d, $^1J_{CP}$ = 98.4 Hz), 58.23, 54.29, 37.57, 37.52, 33.89 (d, $^1J_{CP}$ = 46.8 Hz), 29.88, 29.84, 28.76, 27.93, 27.70 (d, $^2J_{CP}$ = 7.2 Hz), 27.60 (d, $^2J_{CP}$ = 8.4 Hz), 26.18 (The sum of carbon signals must be 84 in theory. Observed 84.); ^{31}P NMR (202 MHz, $CDCl_3$) δ 7.40 (H_2O@**3b**); HRMS (APCI, negative ion mode) calcd for $C_{100}H_{59}N_2O_3SP$ (**3b**$^{\bullet-}$) 1399.4023, found 1399.4041.

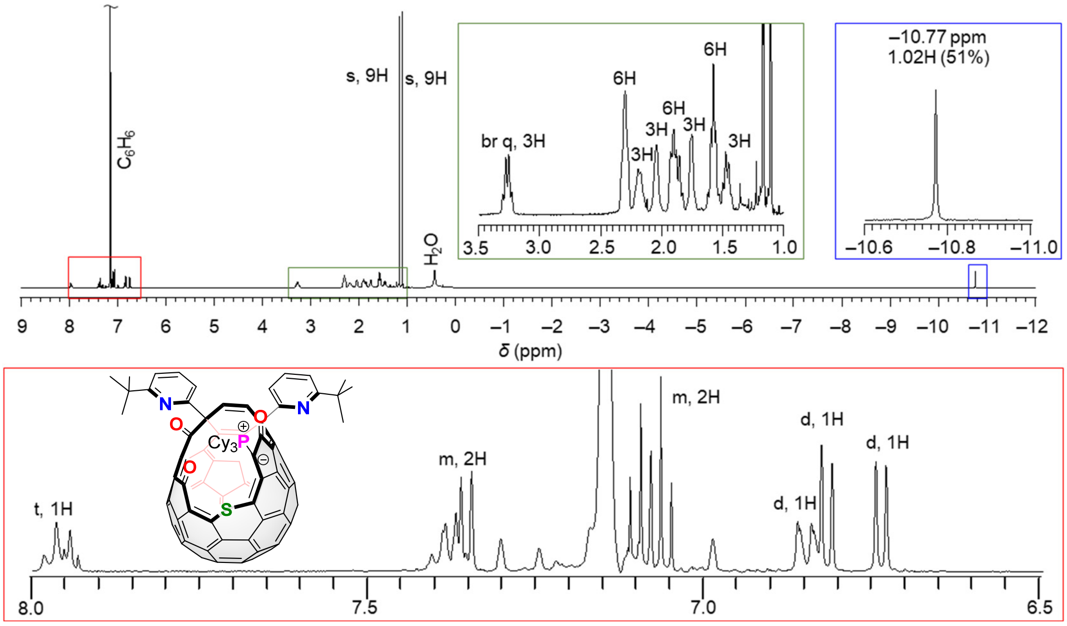

Supplementary Fig. 8. ^{1}H NMR spectra (500 MHz, benzene-d_6, 70 °C) of **3b**.

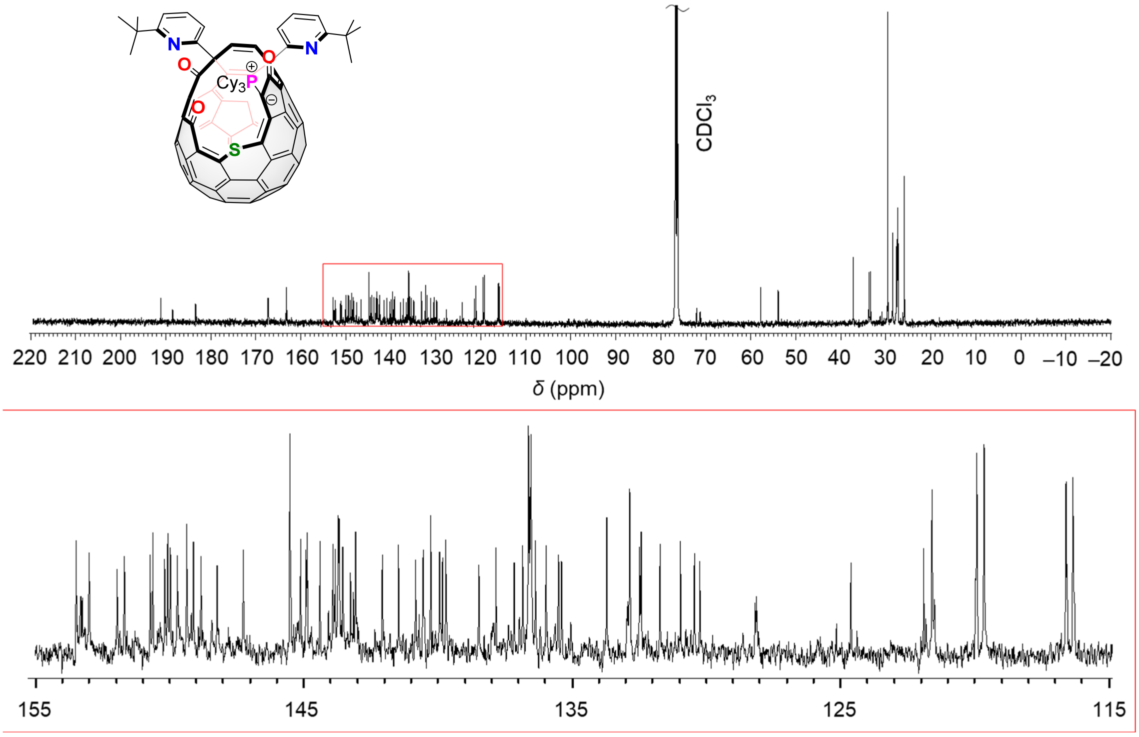

Supplementary Fig. 9. ^{13}C NMR spectra (126 MHz, $CDCl_3$) of **3b**.

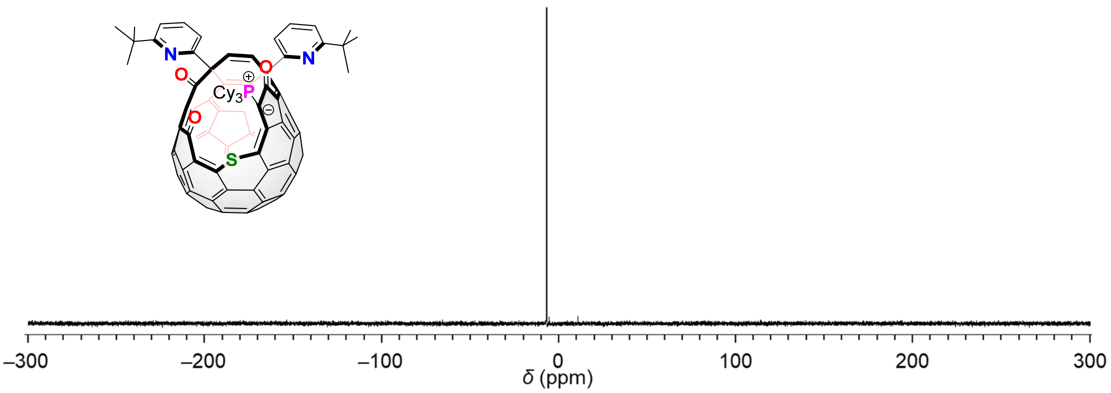

Supplementary Fig. 10. ^{31}P NMR spectrum (202 MHz, $CDCl_3$) of **3b**.

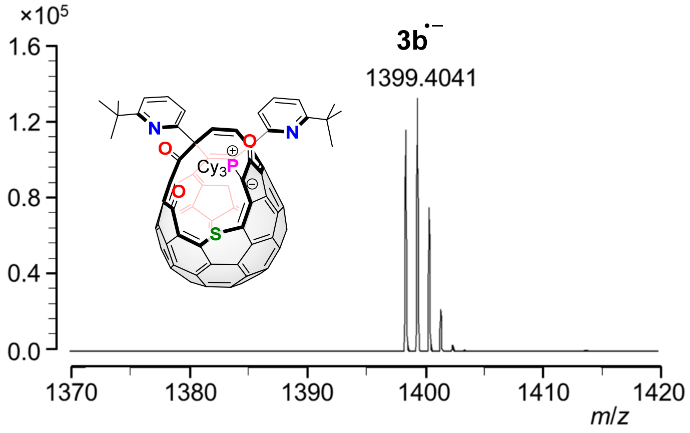

Supplementary Fig. 11. APCI mass spectrum (negative ion mode) of **3b**.

4.3. Synthesis of 3c by the Reaction with $P(NMe_2)_3$

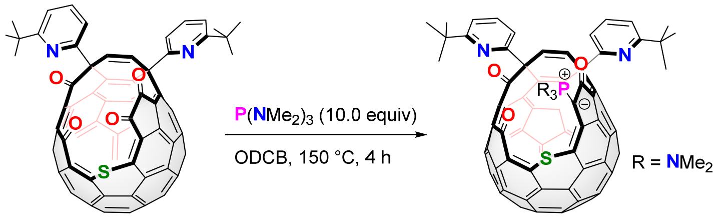

Compound **1** (9.97 mg, 8.78 μmol) was placed into a Schlenk tube and degassed through three vacuum-Ar cycles. *o*-Dichlorobenzene (1.0 mL, 8.8 mM) and then $P(NMe_2)_3$ (16.0 μL, 87.8 mmol, 10.0 equiv) were added to the tube. The resulting solution was heated at 150 °C for 4 hours. After reaction, ODCB, $P(NMe_2)_3$, and its oxide were removed under reduced pressure. The chromatographic purification using silica gel (toluene/AcOEt (10:1)) gave **3c** (9.29 mg, 7.24 μmol) in 82% isolated yield as black powder.

H_2O@**3c** (H_2O: 61%): IR (KBr) *v* 1686 (C=O), 1700 (C=O), 1736 (C=O) cm^{-1}; ^{1}H NMR (500 MHz, $CDCl_3$) δ 7.55 (t, J = 7.8 Hz, 1H), 7.48 (d, J = 10.4 Hz, 1H), 7.44 (d, J = 7.7 Hz, 1H), 7.35 (t, J = 7.7 Hz, 1H), 7.18 (d, J = 7.8 Hz, 1H), 7.12 (d, J = 7.7 Hz, 1H), 7.00 (d, J = 7.8 Hz, 1H), 6.43 (d, J = 10.4 Hz, 1H), 3.08 (d, $^{3}J_{HP}$ = 9.6 Hz, 18H), 1.12 (s, 9H), 1.06 (s, 9H), –11.11 (s, 1.22H); ^{13}C NMR (126 MHz, $CDCl_3$) δ 192.46, 188.56 (d, $^{2}J_{CP}$ = 13.2 Hz), 183.92, 167.83, 167.79, 164.25, 163.94, 153.40, 152.77, 152.11, 151.70, 151.38, 151.29, 150.80, 150.50, 150.17, 150.03, 149.87, 149.71, 149.41, 148.94, 148.83, 148.15, 147.27, 145.46, 145.00, 144.94, 144.62, 144.43, 144.09, 143.99, 143.94, 143.84, 143.79, 143.63, 143.51, 143.01, 142.66, 141.94, 141.28, 140.86, 140.61, 140.53, 139.98, 139.50, 138.31, 137.84, 137.13, 137.08, 136.69, 136.62, 136.46, 136.29, 135.82, 135.49, 133.40, 133.22, 133.12, 132.44, 132.20, 132.14, 131.78, 130.94, 130.37, 124.30, 123.81, 121.64, 120.31, 119.92, 116.72, 116.54, 80.52 (d, $^{1}J_{CP}$ = 193.1 Hz), 58.14, 54.28, 38.16 (d, $^{2}J_{CP}$ = 4.8 Hz), 37.46, 29.82 (The sum of carbon signals must be 79 in theory. Observed 77. Two sp3 signals are overlapped in the aliphatic region.); ^{31}P NMR (202 MHz, $CDCl_3$) δ 51.97 (H_2O@**3c**); HRMS (APCI, negative ion mode) calcd for $C_{88}H_{44}N_5O_3PS$ (**3c**$^{\bullet-}$) 1281.2908, found 1281.2871.

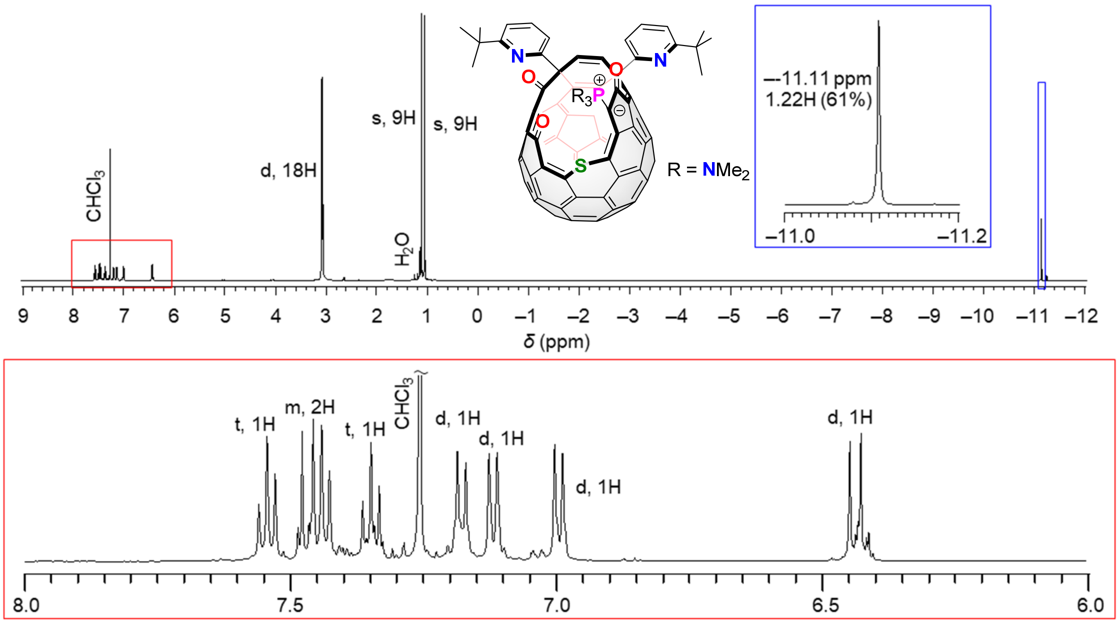

Supplementary Fig. 12. ^{1}H NMR spectra (500 MHz, $CDCl_3$) of **3c**.

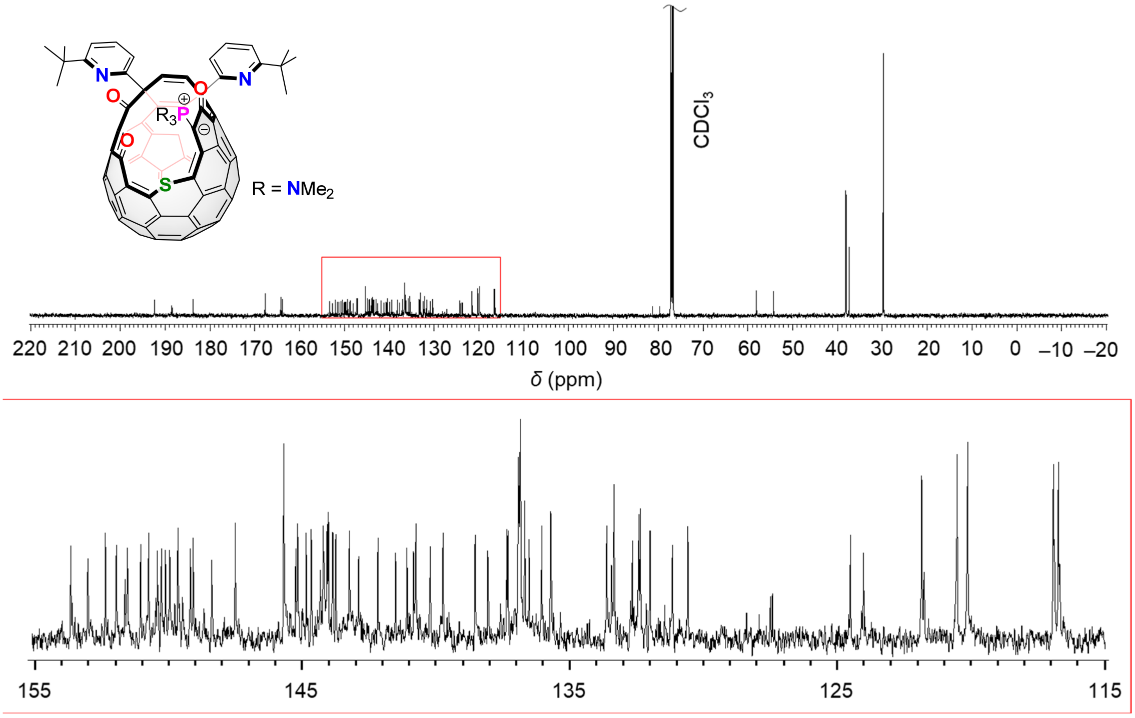

Supplementary Fig. 13. ^{13}C NMR spectra (126 MHz, $CDCl_3$) of **3c**.

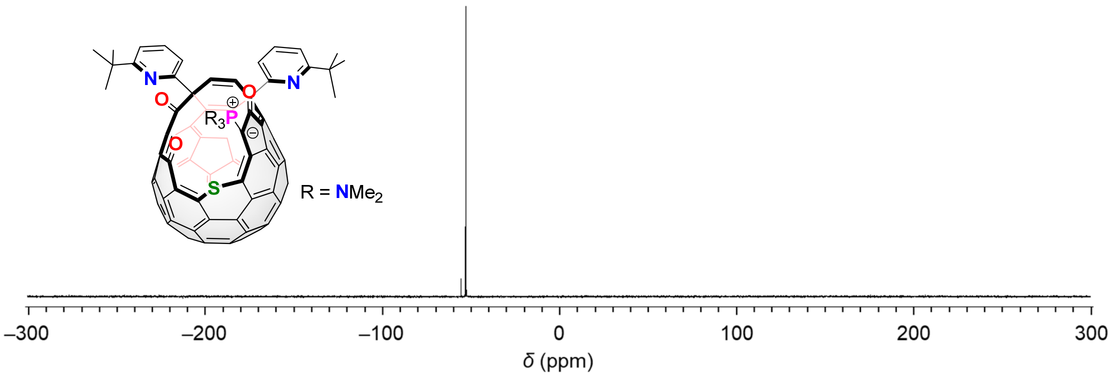

Supplementary Fig. 14. ^{31}P NMR spectrum (202 MHz, $CDCl_3$) of **3c**.

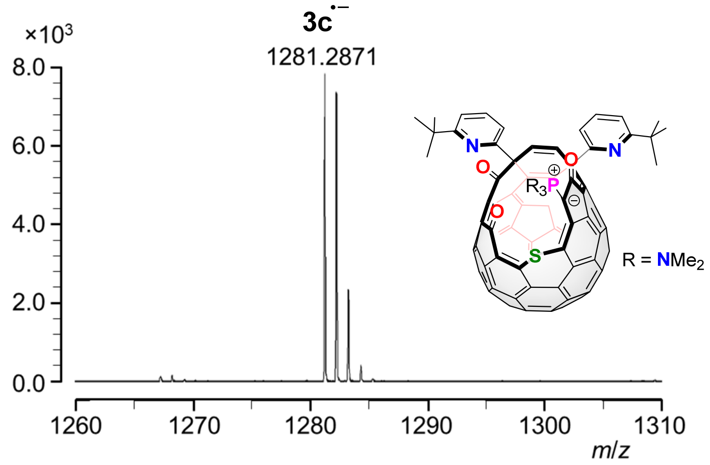

Supplementary Fig. 15. APCI mass spectrum (negative ion mode) of **3c**.

4.4. Synthesis of 3d by the Reaction with PPh_3

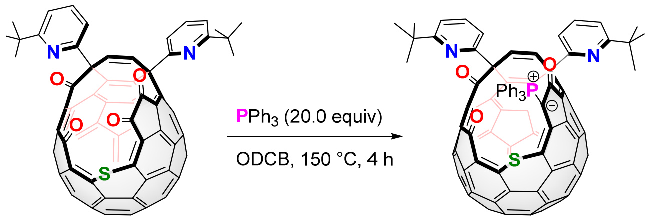

1 3d

Compound **1** (9.97 mg, 8.78 μmol) and PPh_3 (46.2 mg, 176 mmol, 20.0 equiv) were placed into a Schlenk tube and degassed through three vacuum-Ar cycles. *o*-Dichlorobenzene (0.30 mL, 29 mM) was added to the tube. The resulting solution was heated at 150 °C for 4 hours. The chromatographic purification using silica gel (CS_2/AcOEt (50:1)) gave **3d** (8.79 mg, 6.36 μmol) in 72% isolated yield as black powder.

H_2O@**3d** (H_2O: 65%): IR (KBr) ν 1687, 1700, 1734 (C=O); ^{1}H NMR (500 MHz, $CDCl_3$) δ 8.11 (br s, 6H), 7.77 (br s, 6H), 7.77–7.68 (br m, 3H), 7.54 (t, J = 7.8 Hz, 1H), 7.44 (d, J = 7.8 Hz, 1H), 7.36–7.41 (m, 2H), 7.21 (d, J = 7.9 Hz, 1H), 7.12 (d, J = 7.8 Hz, 1H), 7.05 (d, J = 7.8 Hz, 1H), 6.50 (d, J = 10.4 Hz, 1H) 1.12 (m, 18H), –11.14 (s, 1.29H); ^{13}C NMR (126 MHz, $CDCl_3$) δ 192.89, 189.77 (d, $^2J_{CP}$ = 9.6 Hz), 183.54, 168.00, 167.87, 164.14, 163.71, 153.33, 151.98, 151.66, 150.79, 150.53, 150.21, 150.03, 149.94, 149.82, 149.69, 149.43, 148.88, 148.27, 147.46, 145.58, 145.47, 144.92, 144.54, 144.46, 144.01, 143.86, 143.78, 143.68, 143.62, 143.52, 143.10, 142.66, 142.57, 141.18, 140.63, 140.57, 139.93, 138.93, 138.30, 137.80, 137.11, 136.91, 136.71, 136.20, 135.99, 135.73, 135.61, 135.51, 134.91, 133.78, 133.63, 133.37, 133.33, 132.89, 132.61, 132.25, 132.19, 131.86, 131.15, 130.39, 129.42, 129.33, 128.68, 128.49, 128.43, 128.39, 126.25, 124.38, 121.97, 120.25, 120.01, 116.82, 116.60, 116.55, 72.46 (d, $^1J_{CP}$ = 122.4 Hz), 58.32, 54.41, 37.59, 37.50, 29.88, 29.84 (The sum of carbon signals must be 84 in theory. Observed 83. One sp^2 signal is overlapped in the aromatic region.); ^{31}P NMR (202 MHz, $CDCl_3$) δ 14.29 (H_2O@**3d**), 14.24 (**3d**); HRMS (APCI, negative ion mode) calcd for $C_{88}H_{44}N_5O_3PS$ (**3d**$^{\bullet-}$) 1381.2614, found 1381.2600.

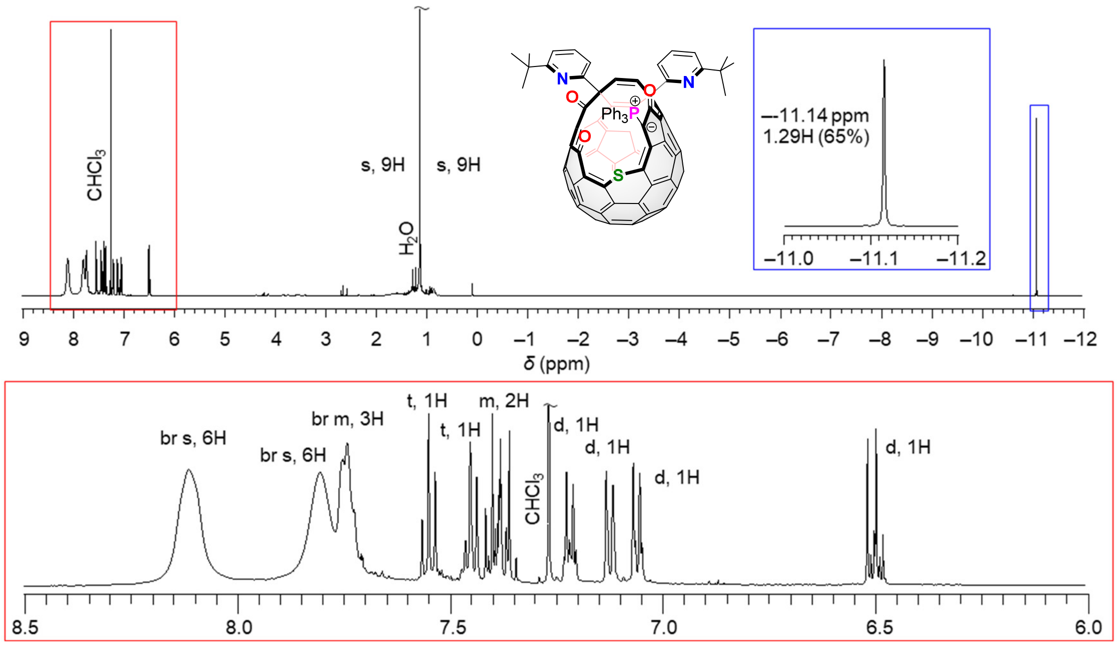

Supplementary Fig. 16. ^{1}H NMR spectra (500 MHz, $CDCl_3$) of **3d**.

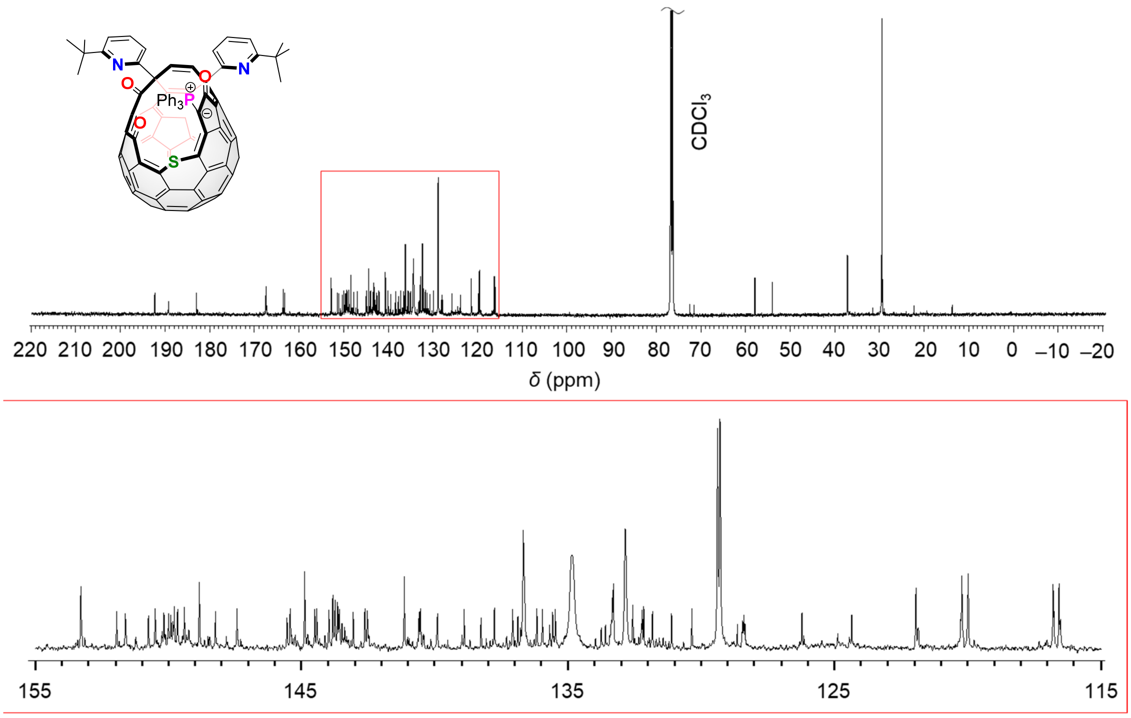

Supplementary Fig. 17. ^{13}C NMR spectra (126 MHz, $CDCl_3$) of **3d**.

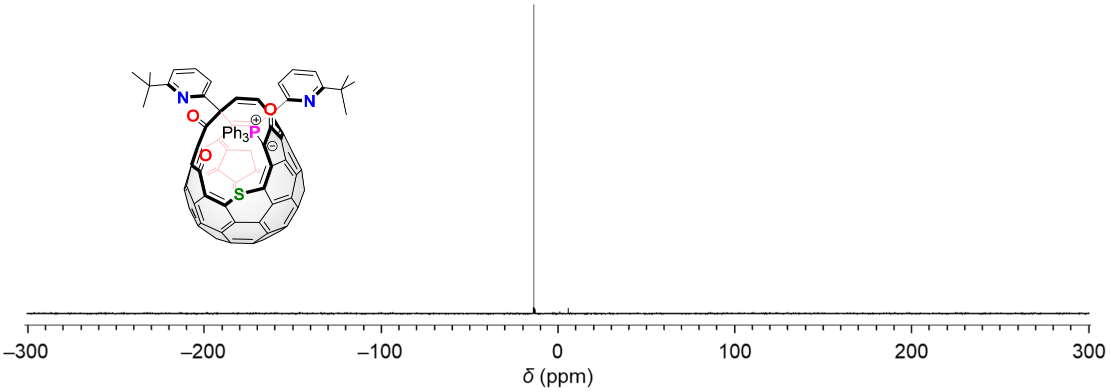

Supplementary Fig. 18. ^{31}P NMR spectrum (202 MHz, $CDCl_3$) of **3d**.

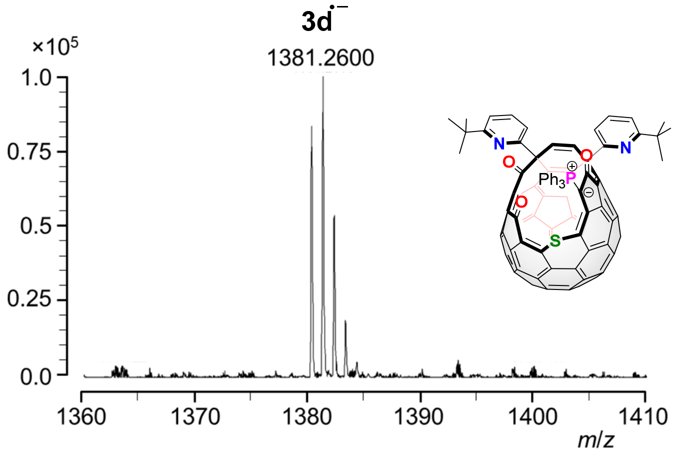

Supplementary Fig. 19. APCI mass spectrum (negative ion mode) of **3d**.

4.5. Synthesis of 3e by the Reaction with dppf

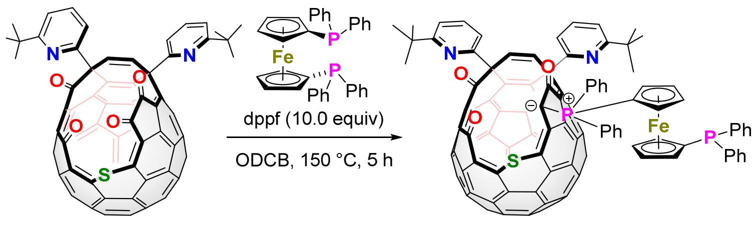

1 **3e**

Compound **1** (10.0 mg, 8.82 μmol) and dppf (48.9 mg, 88.1 μmol, 10.0 equiv) were placed into a Schlenk tube and degassed through three vacuum-Ar cycles. *o*-Dichlorobenzene (0.25 mL, 35 mM) was added to the tube. The resulting solution was heated at 150 °C for 5 hours. The chromatographic purification using silica gel (toluene to toluene/AcOEt (80:1)) gave **3e** (7.24 g, 4.33 mmol) in 49% isolated yield as black powder.

H_2O@**3e** (H_2O: 40%): ^{1}H NMR (500 MHz, CD_2Cl_2) δ 8.33 (br s, 1H), 8.06 (br s, 2H), 7.91 (br s, 5H), 7.79 (br s, 1H), 7.55 (t, J = 7.8 Hz, 1H), 7.44–7.48 (m, 1H), 7.26–7.41 (m, 14H), 7.16 (d, J = 7.9 Hz, 1H), 7.10 (d, J = 7.8 Hz, 1H), 6.46–6.49 (m, 1H), 5.21–5.04 (br s, 1H), 3.99–4.38 (br m, 7H), 1.13 (s, 9H), 1.13 (s, 9H), –11.12 (s, 0.79H); ^{13}C NMR (126 MHz, CD_2Cl_2) δ 193.45, 193.32, 189.20–188.94 (observed as multiplet probably due to conformational isomers and/or encapsulated species), 183.73, 183.50, 168.47, 168.43, 164.55, 164.48, 164.44, 164.38, 164.34, 153.69, 153.63, 153.57, 153.53, 153.47, 152.33, 152.25, 152.01, 151.63, 151.59, 151.12, 150.94, 150.86, 150.80, 150.68, 150.56, 150.42, 150.34, 150.20, 150.06, 149.90, 149.84, 149.77, 149.70, 149.59, 149.27, 149.20, 149.08, 148.94, 148.79, 148.56, 148.15, 148.11, 147.69, 147.65, 147.54, 145.90, 145.86, 145.76, 145.68, 145.64, 145.43, 145.33, 145.30, 145.21, 145.02, 144.92, 144.72, 144.68, 144.57, 144.42, 144.26, 144.17, 144.08, 144.00, 143.97, 143.93, 143.86, 143.68, 143.61, 143.52, 143.19, 143.16, 143.12, 142.95, 142.90, 141.46, 141.32, 141.21, 141.02, 140.96, 140.88, 140.59, 140.22, 139.83, 139.39, 139.18, 138.87, 138.80, 138.73, 138.68, 138.49, 138.40, 138.23, 138.18, 137.78, 137.54, 137.44, 137.34, 137.25, 137.16, 137.11, 137.02, 136.87, 136.65, 136.55, 136.17, 136.13, 136.01, 135.88, 135.82, 135.47, 135.29, 135.21, 135.14, 134.51, 133.82, 133.75, 133.65, 132.98, 132.89, 132.68, 132.64, 132.56, 132.35, 132.17, 131.83, 131.77, 131.69, 131.62, 131.55, 131.39, 131.32, 130.85, 130.56, 130.01, 129.92, 129.45, 129.34, 129.22, 129.14, 128.82, 128.70, 128.67, 128.65, 128.62, 128.49, 128.44, 125.85, 125.73, 125.47, 125.03, 124.96, 122.40, 122.34, 122.29, 120.60, 120.36, 120.30, 117.42, 117.38, 117.17, 117.13, 79.31, 79.24, 77.04, 76.49, 76.32, 75.50, 75.33, 75.09, 75.03, 75.00, 74.95, 74.26, 74.18, 74.07, 73.53, 72.75, 72.72, 58.68, 58.62, 54.85, 54.83, 37.87, 37.81, 30.15, 29.99 (The carbon signals of H_2O@**3e** and **3e** were

partly distinguishable. The sum of carbon signals for each molecule must be 96 in theory. Observed 187 out of 192.); ^{31}P NMR (202 MHz, $CDCl_3$) δ 15.34 (*P*=C, br s, 1P), –18.08 (s, 1P); HRMS (APCI, negative ion mode) calcd for $C_{116}H_{54}FeN_2O_3PS$ (**3e**$^{\bullet-}$) 1673.2720, found 1673.2685.

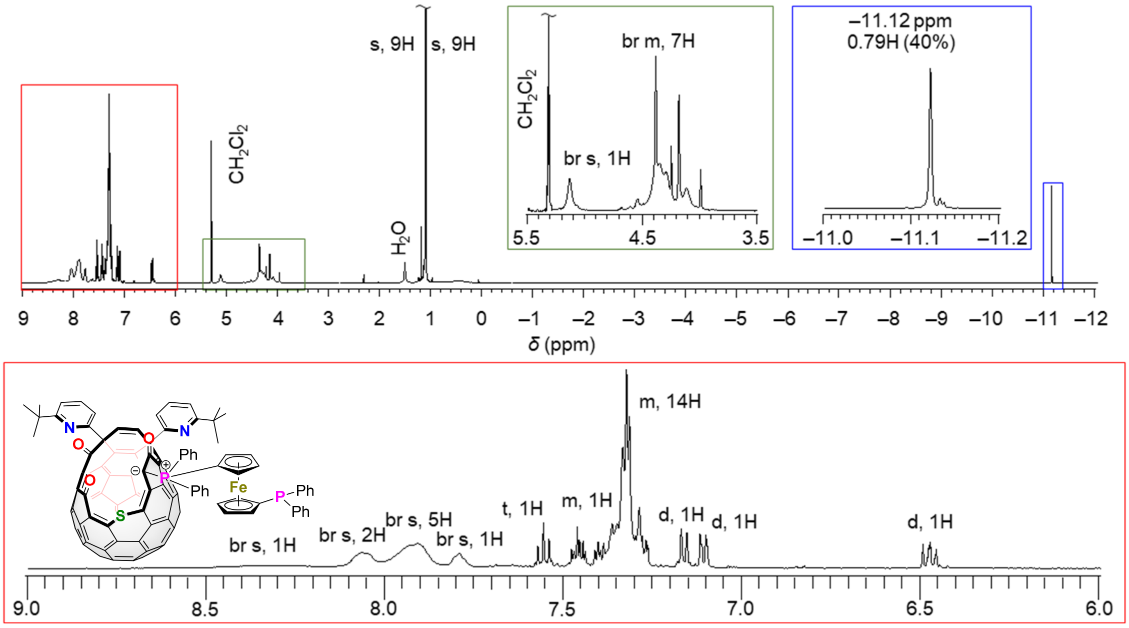

Supplementary Fig. 20. ^{1}H NMR spectra (500 MHz, CD_2Cl_2) of **3e**.

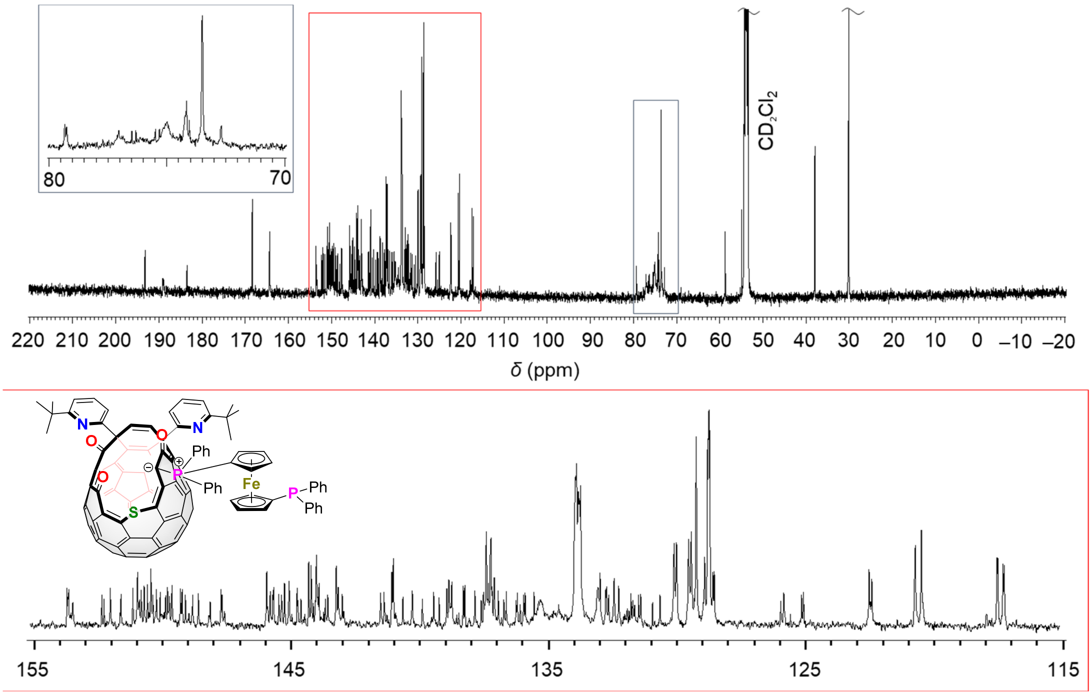

Supplementary Fig. 21. ^{13}C NMR spectra (126 MHz, CD_2Cl_2) of **3e**.

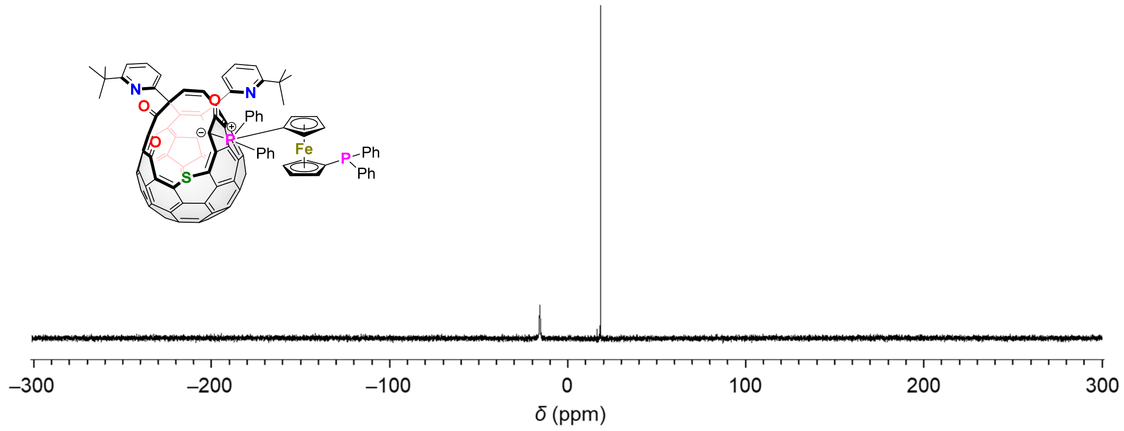

Supplementary Fig. 22. ^{31}P NMR spectrum (202 MHz, $CDCl_3$) of **3e**.

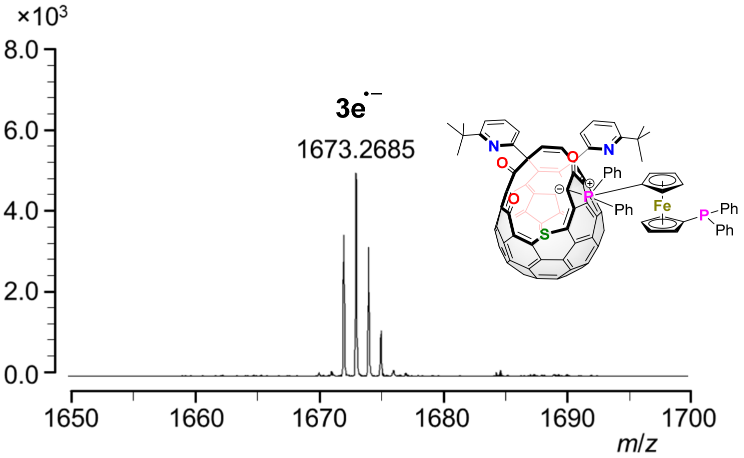

Supplementary Fig. 23. APCI mass spectrum (negative ion mode) of **3e**.

5. Synthesis of Methylene Derivative (4)

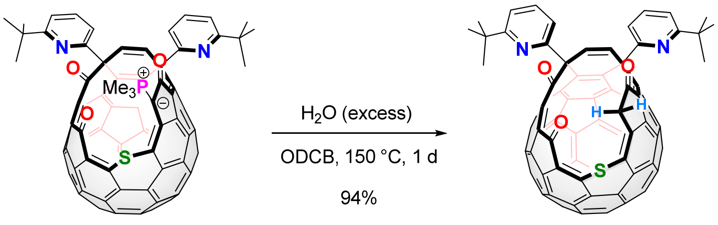

3a **4**

β-Oxo-phosphorus ylide **3a** (4.79 mg, 4.01 μmol) was placed into a Schlenk tube and degassed through three vacuum-Ar cycles. *o*-Dichlorobenzene (0.50 mL, 8.0 mM) and distilled water (7.2 μL, 0.40 mmol, 1.0×10^2 equiv) were added to the tube. The resulting solution was stirred at 150 °C for 1 day. The chromatographic purification using silica gel (CS_2/AcOEt (80:1)) gave **4** (4.22 mg, 3.76 μmol) in 94% isolated yield as brown powder.

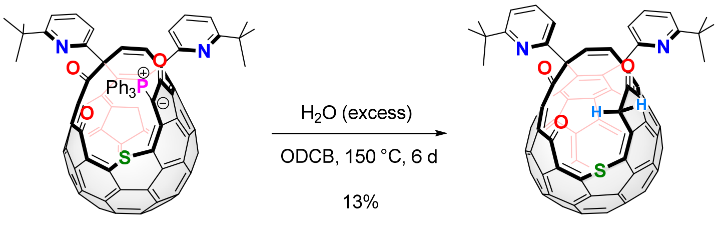

3d **4**

β-Oxo-phosphorus ylide **3d** (5.02 mg, 3.63 μmol) was placed into a Schlenk tube and degassed through three vacuum-Ar cycles. *o*-Dichlorobenzene (0.50 mL, 7.3 mM) and distilled water (3.27 μL, 0.18 mmol, 50 equiv) were added to the tube. The resulting solution was stirred at 150 °C for 6 days. The chromatographic purification using silica gel (CS_2/AcOEt (50:1)) gave a mixture of **4**, **3d**, and $O{=}PPh_3$ (1.37 mg in total). From the ^1H NMR analysis, the ratio was estimated to be 38:47:15. Thus, the yield of **4** was calculated to be 13%.

H_2O@**4** (H_2O: 72%): ^{1}H NMR (500 MHz, $CDCl_3$) δ 7.58 (t, J = 7.9 Hz, 1H), 7.47 (t, J = 7.9 Hz, 1H), 7.19 (d, J = 7.9 Hz, 1H), 7.16 (d, J = 7.9 Hz, 1H), 7.08 (d, J = 7.9 Hz, 1H), 7.02 (d, J = 10.3 Hz, 1H), 6.54 (d, J = 10.3 Hz, 1H), 4.08 (d, J = 21.8 Hz, 1H), 4.00 (d, J = 21.8 Hz, 1H), 1.18 (s, 9H), 1.08 (s, 9H), –11.47 (s, 1.43H) (One sp^2 signal is overlapped with the solvent peak.); ^{13}C NMR (126 MHz, $CDCl_3$) δ 197.36, 191.52, 184.57, 168.31, 163.60, 162.63, 156.19, 152.46, 151.52, 151.05, 150.48, 150.16, 150.08, 149.78, 149.64, 149.51, 149.48, 149.28, 148.95, 148.90, 146.99, 146.00, 144.91, 144.87, 144.83, 144.81, 144.52, 144.41, 143.96, 143.94, 143.42, 143.38, 143.19, 143.05, 141.95, 141.56, 141.47, 141.11, 140.53, 139.80, 139.72, 139.47, 139.29, 138.99, 138.71, 138.05, 137.79, 137.66, 137.16, 136.96, 136.81, 136.43, 136.37, 136.07, 135.52, 133.97, 132.99, 132.13, 132.09, 131.88, 130.95, 130.48, 129.97, 124.88, 124.58, 119.86, 119.70, 117.19, 117.00, 58.82, 54.14, 43.06, 37.60, 29.86, 29.80 (The sum of carbon signals must be 78 in theory. Observed 75. Two peaks are overlapped in the aromatic region and one peak is overlapped in the aliphatic region.); HRMS (APCI, negative ion mode) calcd for $C_{82}H_{28}N_2O_3S$ (**4**$^{\bullet-}$) 1120.1826, found 1120.1833.

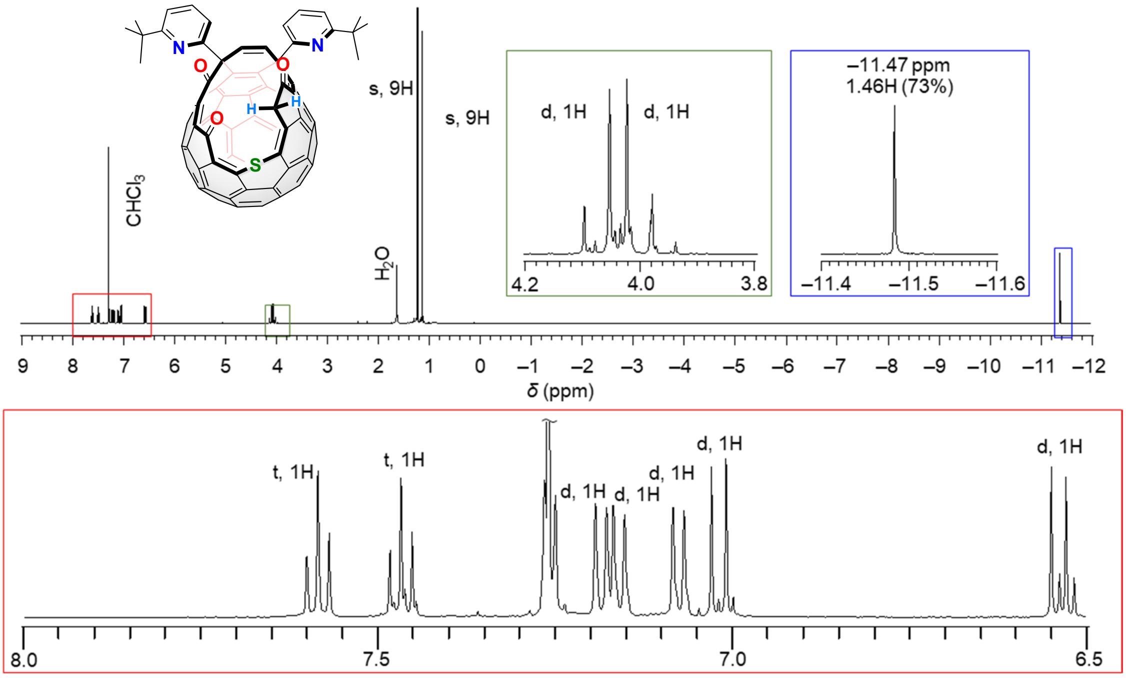

Supplementary Fig. 24. ^{1}H NMR spectra (500 MHz, $CDCl_3$) of **4**.

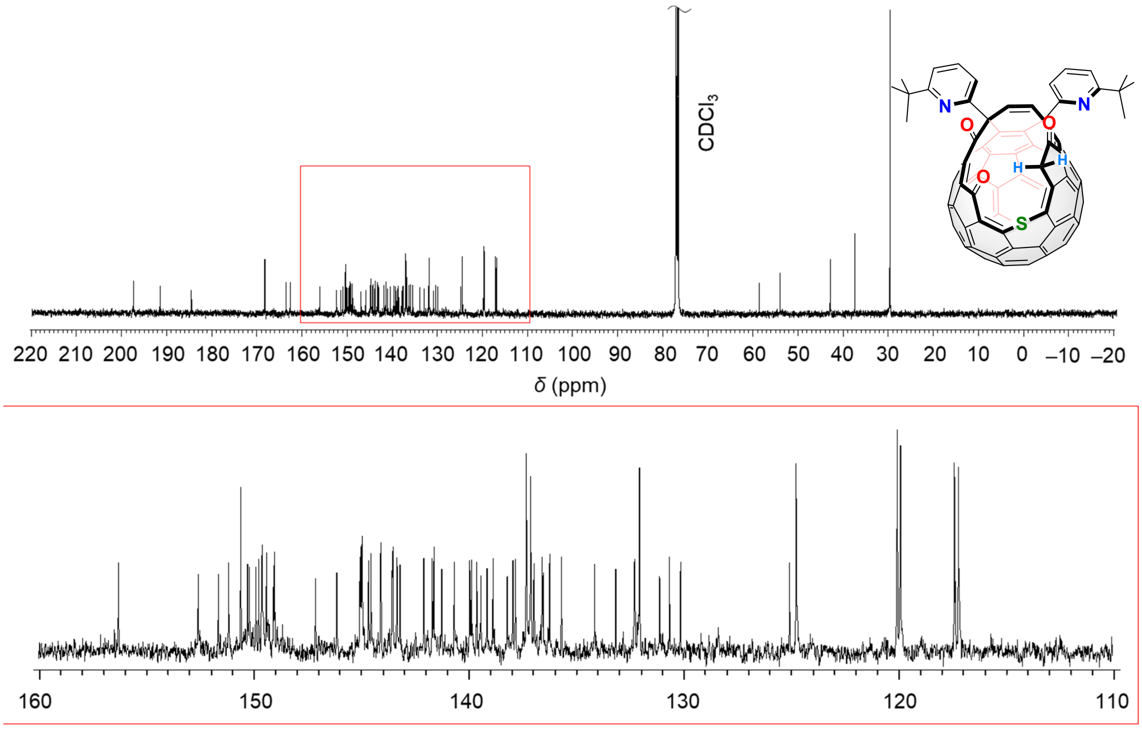

Supplementary Fig. 25. ^{13}C NMR spectra (126 MHz, $CDCl_3$) of **4**.

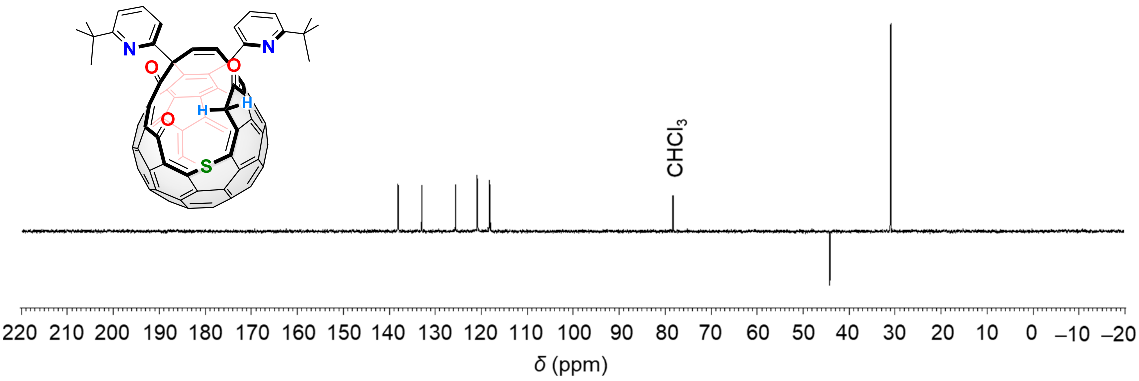

Supplementary Fig. 26. ^{13}C DEPT 135 spectrum (126 MHz, $CDCl_3$) of **4**.

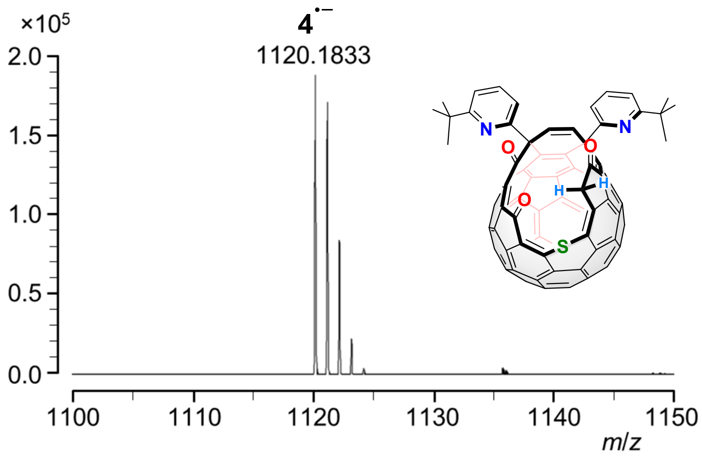

Supplementary Fig. 27. APCI mass spectrum (negative ion mode) of **4**.

6. UV-Vis-NIR Absorption Spectra

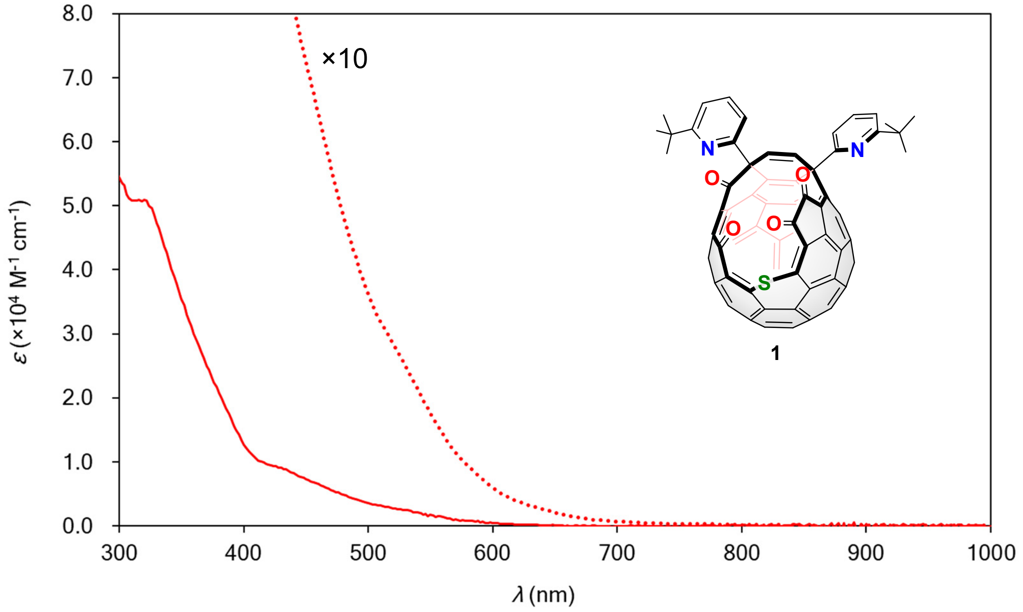

Supplementary Fig. 28. UV-Vis-NIR absorption spectrum of **1** (50 μM in toluene).

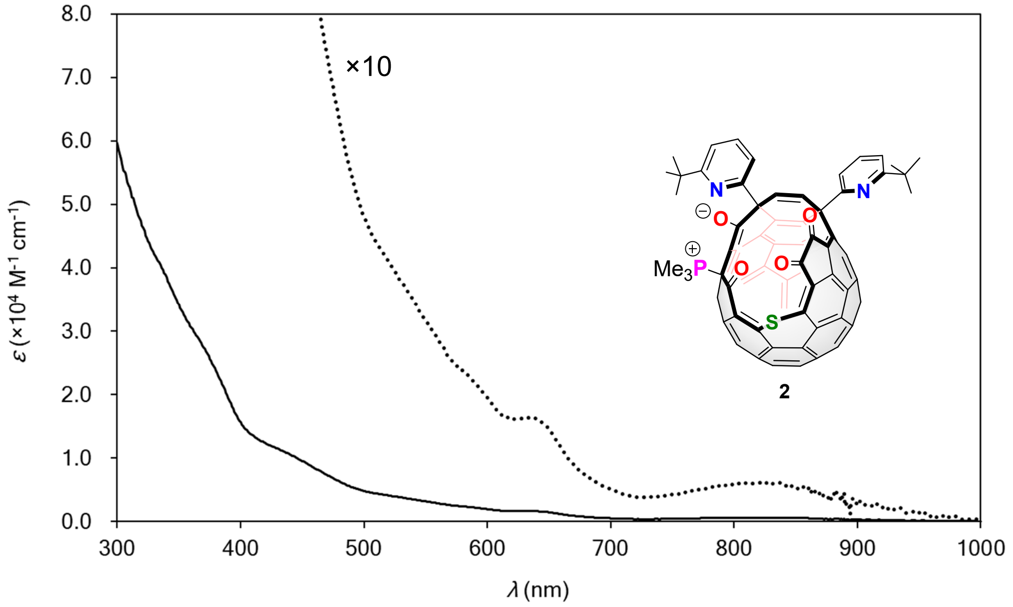

Supplementary Fig. 29. UV-Vis-NIR absorption spectrum of **2** (50 μM in toluene).

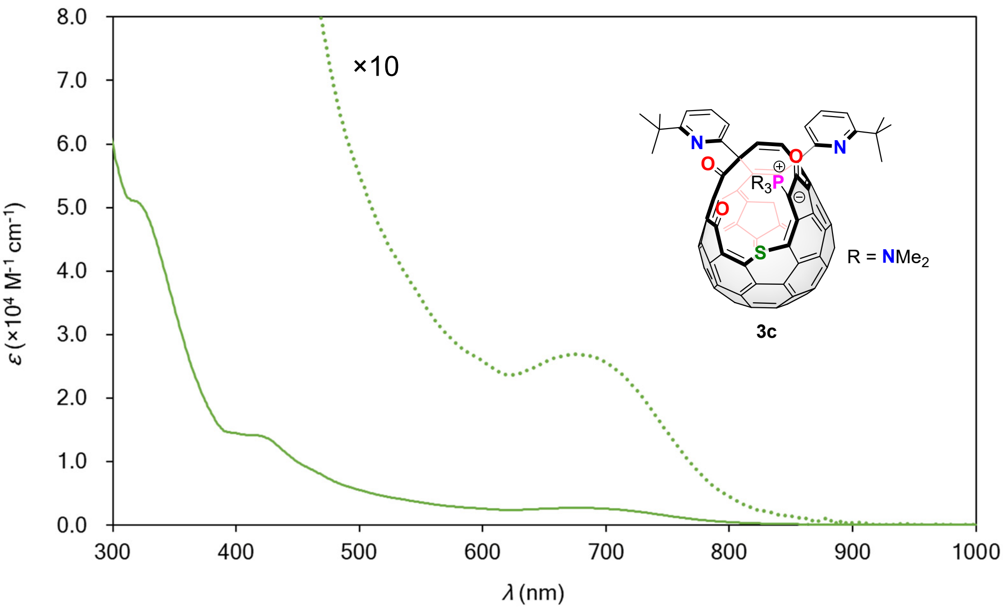

Supplementary Fig. 30. UV-Vis-NIR absorption spectrum of **3c** (50 μM in toluene).

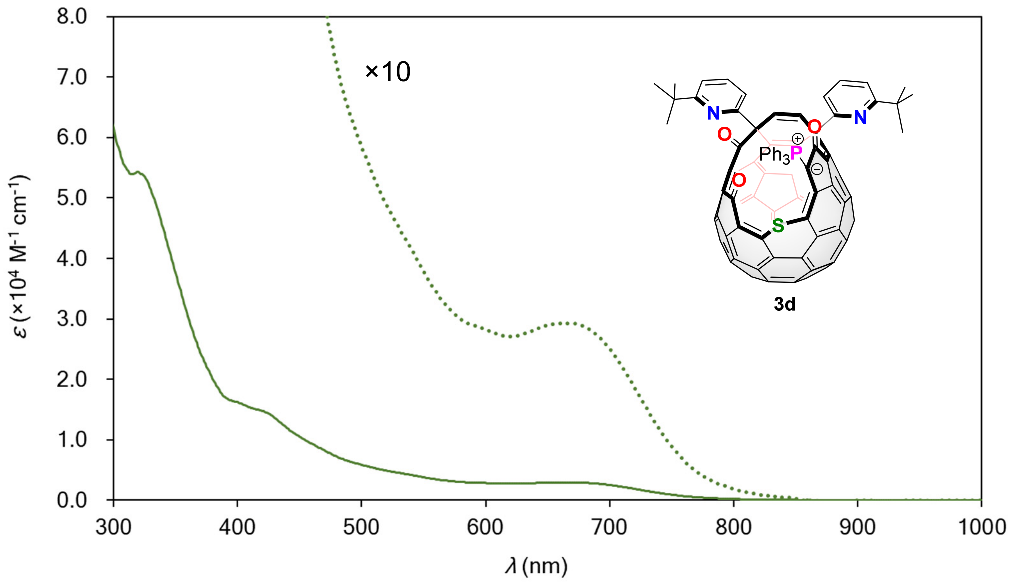

Supplementary Fig. 31. UV-Vis-NIR absorption spectrum of **3d** (50 μM in toluene).

7. Cyclic Voltammograms

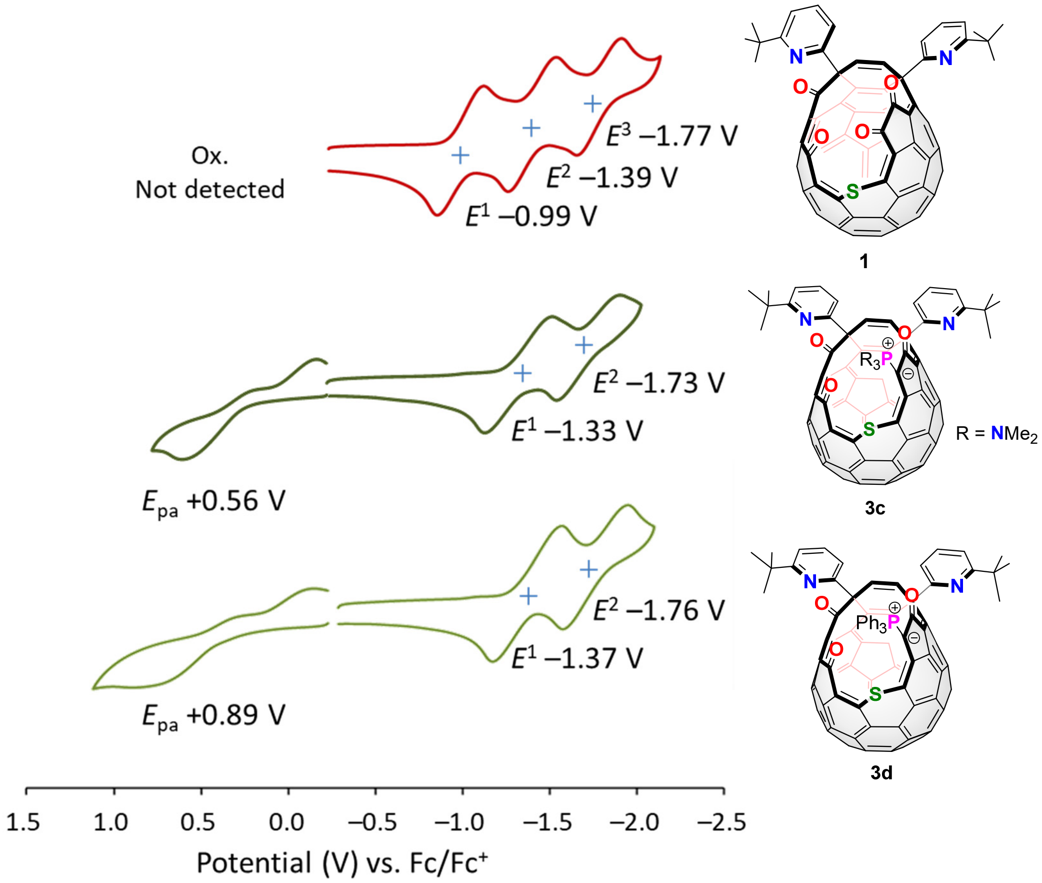

Supplementary Fig. 32. Cyclic voltammograms of **1**, **3c**, and **3d** (1 mM in ODCB, 0.10 M n-$Bu_4N{\cdot}BF_4$, 100 mV s^{-1}).

8. IR spectra

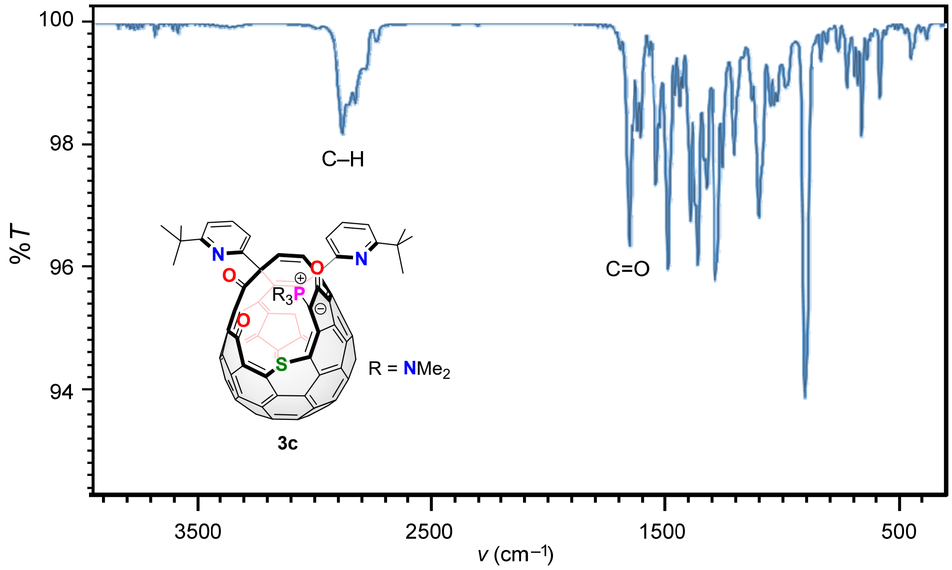

Supplementary Fig. 33. IR spectra (KBr) of **3c**.

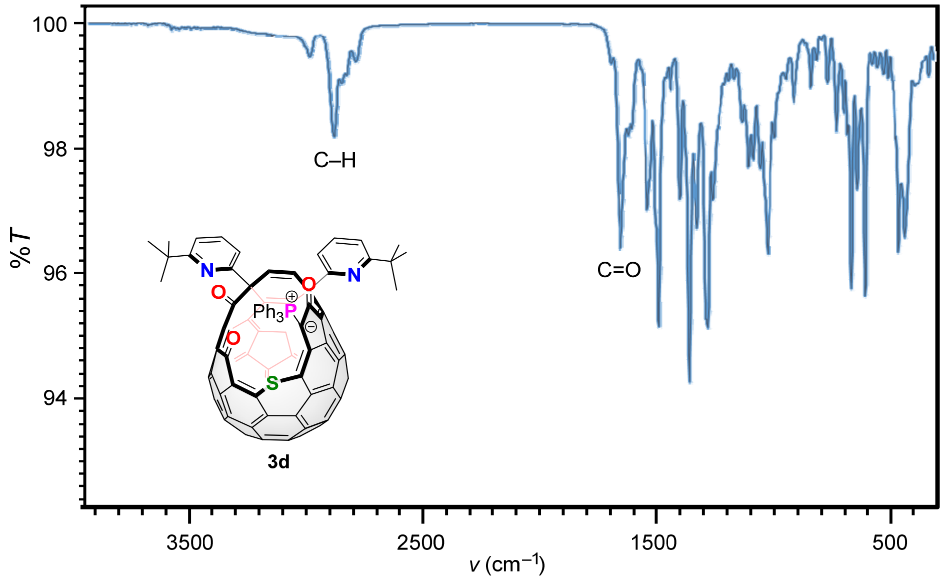

Supplementary Fig. 34. IR spectra (KBr) of **3d**.

9. Single Crystal X-Ray Structures

9.1. Crystal Structure of $[(H_2O)_{0.60(4)}(N_2)_{0.24(3)}@2]$ $\bullet[(H_2O)_{0.61(4)}(N_2)_{0.21(3)}@2]\bullet(C_6H_5CH_3)_{6.11}\bullet(CHCl_3)_{1.07}$

Single crystals of **2** were obtained by liquid-liquid diffusion of a chloroform solution of **1** into PMe_3 in toluene at 5 °C as shown in Supplementary Fig. 35. Intensity data were collected at 100 K on a Bruker Single Crystal CCD X-ray Diffractometer (SMART APEX II) with Mo Kα radiation (λ = 0.71073 Å) and graphite monochromator. A total of 131753 reflections were measured at the maximum 2θ angle of 49.0°, of which 23918 were independent reflections (R_{int} = 0.0715). The structure was solved by direct methods (SHELXT-2014/5[2]) and refined by the full-matrix least-squares on F^2 (SHELXL-2018/3[2]). The two independent molecules of **2** was confirmed in an asymmetric unit. The water and nitrogen molecules were disordered inside each cage of **2**, which was solved using appropriate models. Thus, [(H5–O9–H330) and (N5–N6)] and [(H10–O10–H331) and (N7–N8)] were placed and their occupancies were refined to be [0.60(4) and 0.24(3)] and [0.61(4) and 0.21(3)], respectively. This crystal contains several disordered solvent molecules consisting of toluene and chloroform, all of which were solved using appropriate models. Thus, two sets of toluene molecule, (C183–C184–C185–C186–C187–C188–C211) and (C171–C172–C173–C174–C175–C176–C229) were placed and their occupancies were refined to be 0.549(12) and 0.451(12), respectively. Two sets of toluene molecule, (C189–C196–C197–C235–C137–C240–C233) and (C199–C200–C201–C202–C203–C204–C269) were placed and their occupancies were refined to be 0.641(10) and 0.359(10), respectively. Two toluene molecules and two chloroform molecules were disordered at the similar positions, i.e., [(C213–C214–C215–C216–C217–C218–C278) and (C271–C272–C273–C274–C275–C276–C277)] and [(C220–Cl4–Cl5–Cl6) and (C270–Cl7–Cl8–Cl9)], which were refined to be 0.846(6) and 0.154(6), respectively. Two non-disordered toluene molecules, i.e., (C252–C253–C254–C255–C256–C257–C258) and (C177–C178–C179–C180–C181–C182–C228) were placed and their occupancies were refined to be 0.30 and 0.403(9), respectively. All non-hydrogen atoms except for an encapsulated H_2O molecule and a part of disordered solvent molecules were refined anisotropically. All hydrogen atoms were placed using AFIX instructions. A part of disordered molecules was refined using DFIX and SIMU instructions. The crystal data are as follows: $C_{213.87}H_{122.39}Cl_{3.22}N_{4.90}O_{9.20}P_2S_2$; FW = 3148.19, crystal size 0.30 × 0.10 × 0.03 mm^3, monoclinic, $P2/n$, a = 24.337(8) Å, b = 18.173(6) Å, c = 35.233(12) Å, β = 101.168(5)°, V = 15288(9) Å^3, Z = 4, D_c = 1.368 g cm^{-3}. The refinement converged to R_1 = 0.1124, wR_2 = 0.2578 ($I > 2\sigma(I)$), GOF = 1.097.

The data was deposited at the Cambridge Crystallographic Data Centre (CCDC 1988945).

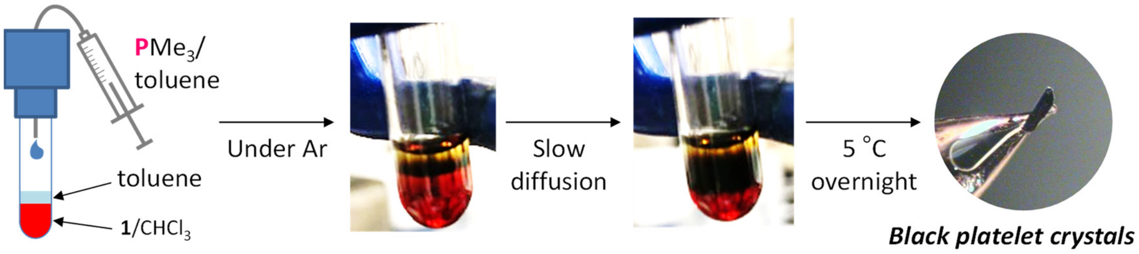

Supplementary Fig. 35. Procedure to prepare single crystals of **2**.

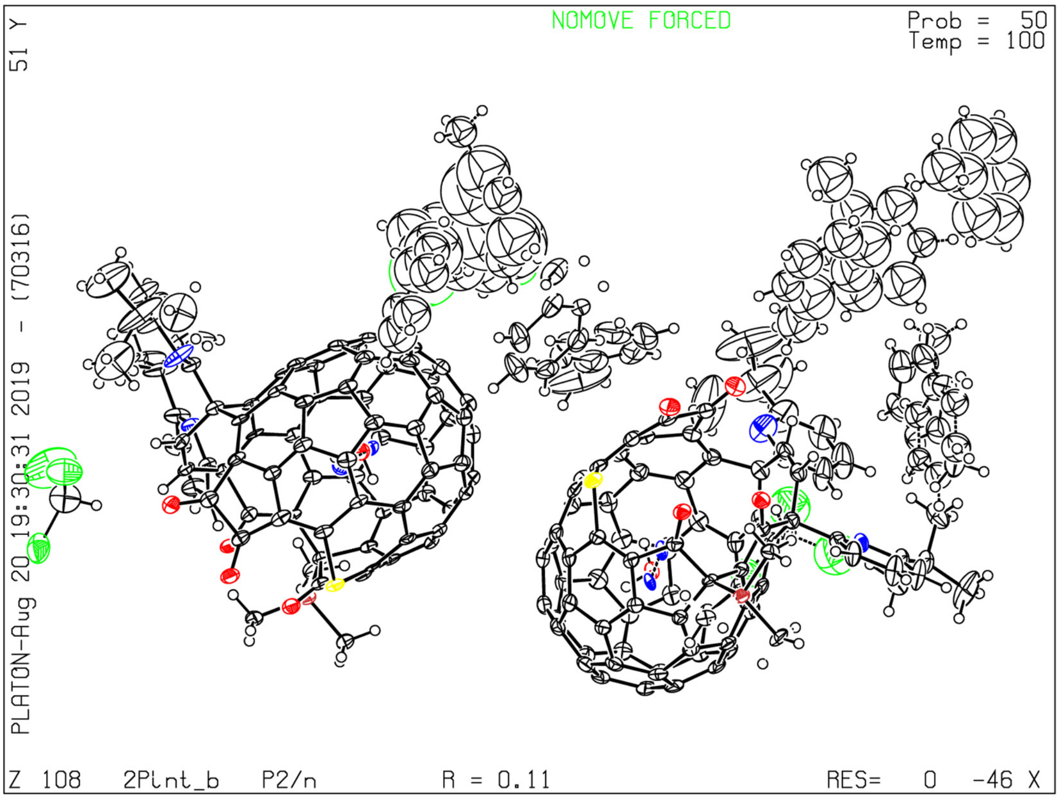

Supplementary Fig. 36. Single crystal X-ray structure of $[(H_2O)_{0.60(4)}(N_2)_{0.24(3)}@\mathbf{2}]\bullet[(H_2O)_{0.61(4)}(N_2)_{0.21(3)}@\mathbf{2}]\bullet(C_6H_5CH_3)_{6.11}\bullet(CHCl_3)_{1.07}$.

9.2. Crystal Structure of (3d)•$(3CH_2Cl_2)_{0.4}$•$(hexane)_{0.6}$

Single crystals of **3d** were obtained from a CH_2Cl_2/hexane solution. Intensity data were collected at 100 K on a Bruker Single Crystal CCD X-ray Diffractometer (SMART APEX II) with Mo Kα radiation (λ = 0.71073 Å) and graphite monochromator. A total of 34420 reflections were measured at the maximum 2θ angle of 50.1°, of which 12662 were independent reflections (R_{int} = 0.0192). The structure was solved by direct methods (SHELXT-2014/5[2]) and refined by the full-matrix least-squares on F^2 (SHELXL-2018/3[2]). One of *t*-butyl groups was disordered, which was solved using appropriate models. Thus, two sets of *t*-butyl group, i.e., (C80–C81–C82) and (C83–C84–C85) were placed and their occupancies were refined to be 0.629(9) and 0.371(9), respectively. A molecule of hexane and three molecules of dichloromethane were disordered. Thus, (C104–C105–C106–C107–C108–C109) and [(C110–Cl4–Cl5), (C111–Cl2–Cl6), and (C112–Cl1–Cl3)] were placed and their occupancies were refined to be 0.60 and 0.40, respectively. All non-hydrogen atoms except for a part of disordered solvent molecules were refined anisotropically. All hydrogen atoms except for an encapsulated H_2O molecule were placed using AFIX instructions. The disordered molecules were refined using DFIX, ISOR, and SIMU instructions. The crystal data are as follows: $C_{104.80}H_{53.80}Cl_{2.40}N_2O_4PS$; FW = 1553.01, crystal size 0.23 × 0.17 × 0.13 mm^3, monoclinic, $P2_1/c$, a = 17.480(7) Å, b = 22.173(9) Å, c = 18.633(7) Å, β = 97.269(5)°, V = 7164(5) Å^3, Z = 4, D_c = 1.440 g cm^{-3}. The refinement converged to R_1 = 0.0615, wR_2 = 0.1715 ($I > 2\sigma(I)$), GOF = 1.066. The data was deposited at the Cambridge Crystallographic Data Centre (CCDC 1988944).

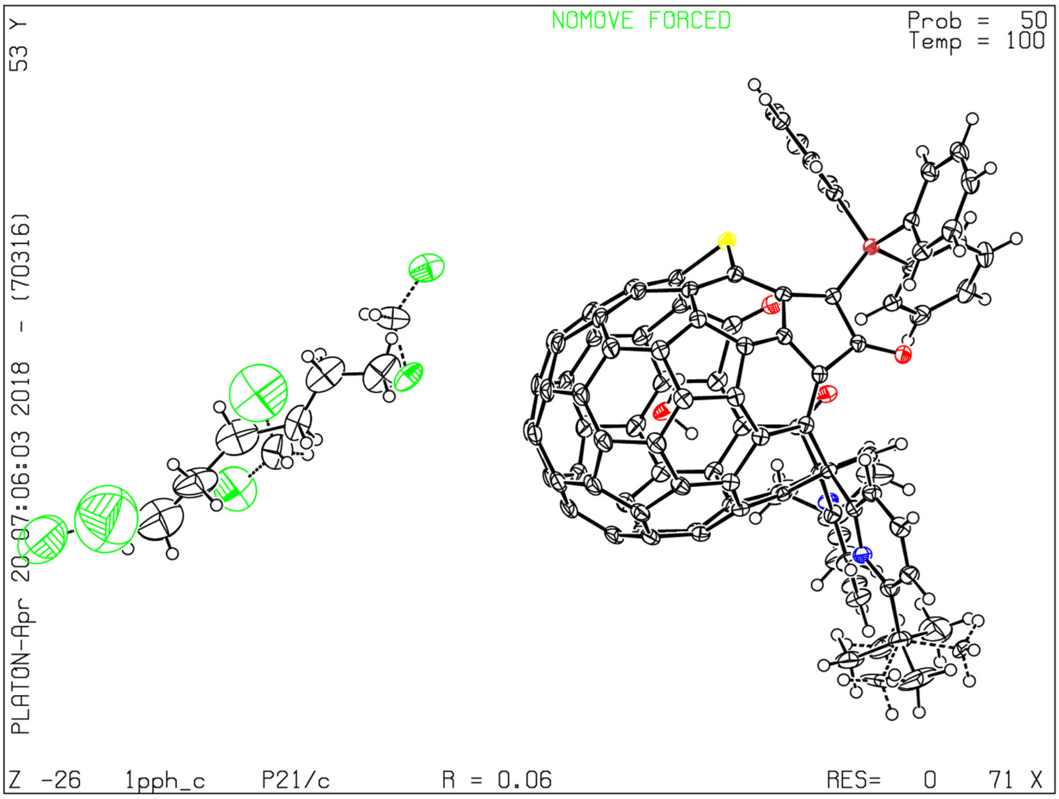

Supplementary Fig. 37. Single crystal X-ray structure of (**3d**)•(3CH_2Cl_2)$_{0.4}$•(hexane)$_{0.6}$.

Supplementary Note 1

10. Formation Mechanism of 1,5-Betaines (2')

The possible pathways for the formation of **2'** via addition of PMe_3 to electron deficient olefins on **1'** were examined by theoretical calculations, except for the carbon atoms which are sterically inaccessible for PMe_3 to attack nucleophilically, as shown in Supplementary Fig. 38. Thus, the conjugate addition to the **A** position is suggested to be kinetically and thermodynamically favorable compared with the others (**B–E**) (Supplementary Fig. 39). This results are consistent with our experimental observation that PM_3 underwent conjugate addition to **1** at room temperature to give **2**.

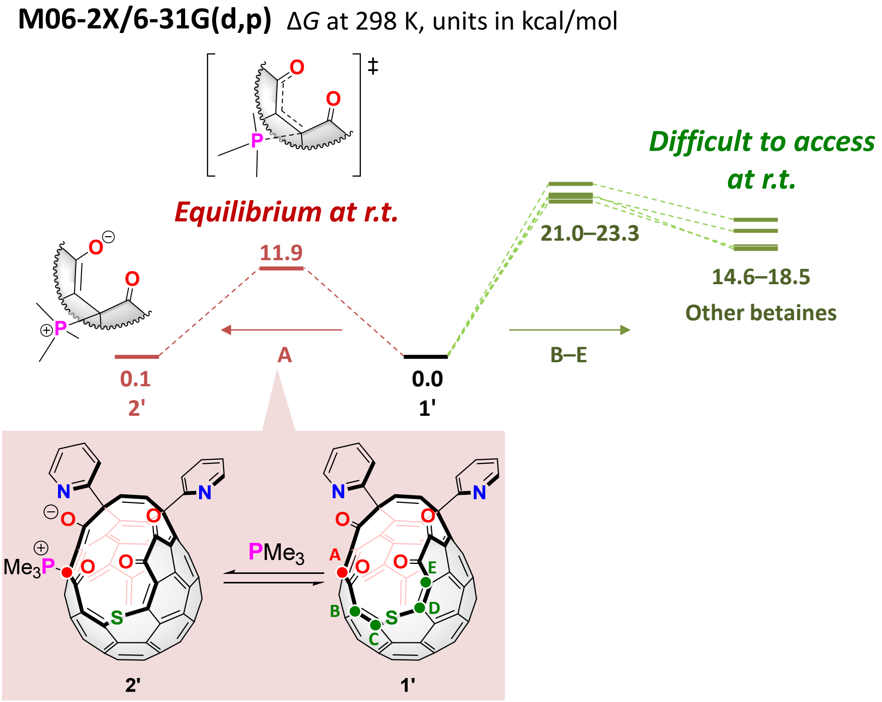

Supplementary Fig. 38. Formation mechanism of **2'** from **1'** (M06-2X/6-31G(d,p)).

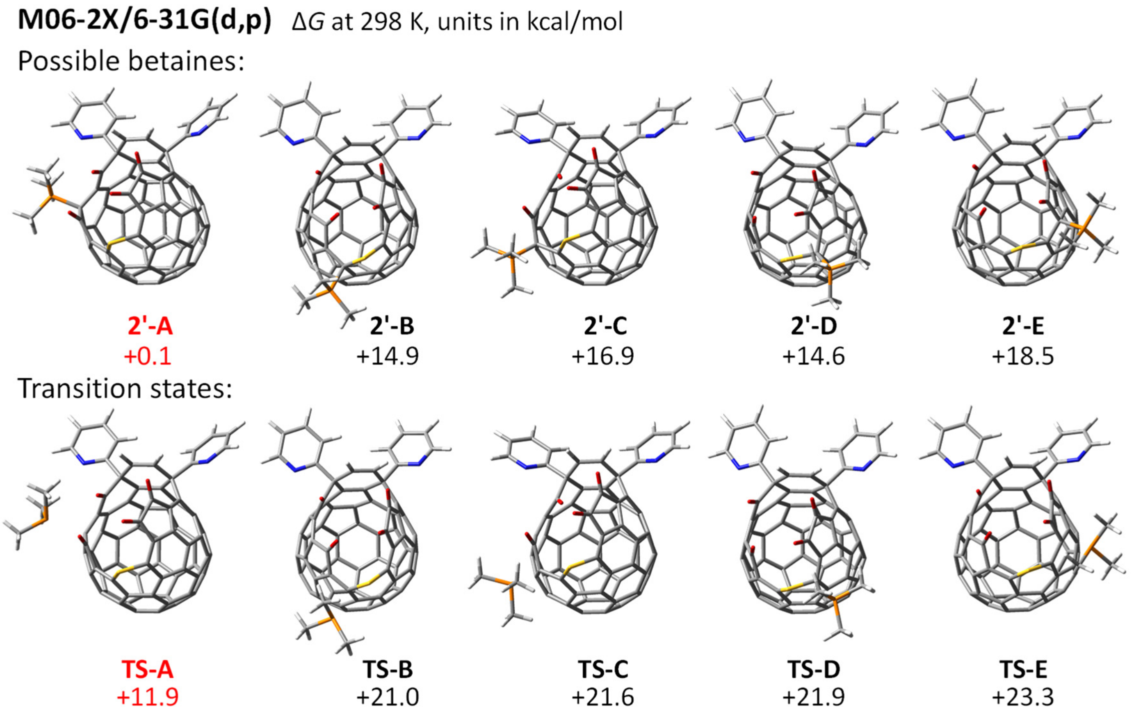

Supplementary Fig. 39. Optimized structures of 1,5-betaines and transition states for the reaction of **1'** and PMe_3 (M06-2X/6-31G(d,p)).

Supplementary Table 1. Optimized structure of **1'** (M06-2X/6-31G(d,p))

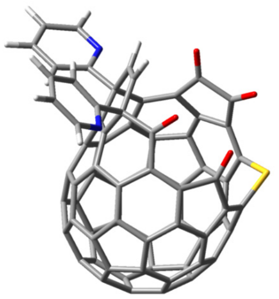

Standard orientation:

Center Number	Atomic Number	Atomic Type	Coordinates (Angstroms) X	Y	Z
1	6	0	3.819525	0.475660	1.389576
2	6	0	3.830525	-0.816832	1.081510
3	6	0	3.009973	-1.443476	-0.014520
4	6	0	1.844986	-2.279323	0.544858
5	6	0	1.502497	-2.345314	1.885992
6	6	0	2.021974	-1.793803	3.200563
7	6	0	0.749538	-1.500959	4.084212
8	6	0	-0.403543	-2.046995	3.319901
9	6	0	-1.732815	-1.675766	3.267334
10	6	0	-2.843306	0.656561	3.004249
11	6	0	-2.249844	1.884162	2.775981
12	6	0	-0.778404	2.244769	3.032317
13	6	0	-0.359955	2.952092	1.718321
14	6	0	0.787757	2.768893	0.958453
15	6	0	2.059787	2.248638	1.562140
16	6	0	3.063513	1.520834	0.618008
17	16	0	-2.259833	-0.360698	4.332598
18	7	0	3.614796	3.847400	0.105621
19	8	0	3.133206	-1.675803	3.635771
20	8	0	2.321652	2.399236	2.727754
21	7	0	4.068727	-3.597151	-0.403078
22	6	0	2.329696	0.942274	-0.566874
23	6	0	1.510745	-0.888860	-1.958895
24	8	0	0.792368	-0.967698	5.157027
25	6	0	1.452311	1.853429	-1.263790
26	6	0	0.909332	-2.787974	-0.462080
27	8	0	-0.104506	1.921347	3.966043
28	6	0	-0.276536	-2.429727	-2.614992
29	6	0	4.926997	-4.395467	-1.040261
30	6	0	-1.433882	-3.147507	-2.118613

31	6	0	3.948602	-2.341406	-0.832220
32	6	0	0.841264	-2.176031	-1.797116
33	6	0	0.996738	0.044595	-2.853094
34	6	0	2.345087	-0.391815	-0.890009
35	6	0	-1.415561	-3.620713	-0.830403
36	6	0	-0.154250	-0.228649	-3.671654
37	6	0	-0.648619	-3.369664	1.321444
38	6	0	-0.784119	-1.447951	-3.552432
39	6	0	0.958623	1.442889	-2.492774
40	6	0	0.697999	2.842723	-0.490759
41	6	0	-0.225873	-3.489620	-0.038755
42	6	0	5.291407	2.211420	-0.409176
43	6	0	4.043416	2.585905	0.091438
44	6	0	-3.657471	-2.724788	-0.414457
45	6	0	-0.454607	3.378070	-1.077782
46	6	0	-0.224306	2.018505	-3.084922
47	6	0	6.127715	3.203402	-0.904817
48	6	0	0.165441	-2.759071	2.224402
49	6	0	-2.237114	-1.500214	-3.563762
50	6	0	5.700882	-3.985228	-2.120988
51	6	0	-4.247901	-1.686044	0.411593
52	6	0	-2.980360	-0.330310	-3.663526
53	6	0	-2.061021	-3.130959	1.357067
54	6	0	5.691981	4.524668	-0.885156
55	6	0	4.685712	-1.828109	-1.901084
56	6	0	-2.590125	-2.128986	2.186966
57	6	0	4.428056	4.791907	-0.371174
58	6	0	-0.922620	0.989911	-3.818029
59	6	0	-4.810227	-0.722359	-0.510891
60	6	0	-4.338717	1.013377	1.113541
61	6	0	-2.554007	-3.399477	0.039711
62	6	0	-1.616275	3.642624	-0.277342
63	6	0	-2.637411	-2.538306	-2.643179
64	6	0	-4.129665	-0.127631	-2.801084
65	6	0	-3.696241	-1.311822	1.654913
66	6	0	5.575346	-2.669962	-2.556291
67	6	0	-1.567333	3.336774	1.067213
68	6	0	-3.776720	0.110550	2.029659
69	6	0	-4.474793	-1.101353	-1.872873
70	6	0	-3.733714	-2.336337	-1.806592
71	6	0	-2.694569	2.683131	1.690940
72	6	0	-2.309386	0.948411	-3.809542
73	6	0	-3.052661	1.944344	-3.062405
74	6	0	-0.924128	2.978883	-2.386085
75	6	0	-4.556366	1.636596	-1.146705
76	6	0	-2.374131	2.949054	-2.376289
77	6	0	-2.804102	3.349470	-1.057087
78	6	0	-3.800083	2.339864	0.947244
79	6	0	-4.855103	0.607713	-0.166724
80	6	0	-3.876571	2.703142	-0.453415
81	6	0	-4.172401	1.275699	-2.431834
82	1	0	4.388591	0.837856	2.240635
83	1	0	4.406909	-1.512548	1.681665
84	1	0	4.997791	-5.413507	-0.664899
85	1	0	5.590279	1.168329	-0.395619
86	1	0	7.107361	2.949346	-1.297369
87	1	0	6.381023	-4.678433	-2.602608
88	1	0	6.314284	5.330935	-1.256117
89	1	0	4.553487	-0.795538	-2.209085
90	1	0	4.047060	5.809446	-0.336410
91	1	0	6.160598	-2.305932	-3.394899

The total electronic energy was calculated to be –3633.172092 Hartree.

Supplementary Table 2. Optimized structure of **2'-A** (M06-2X/6-31G(d,p))

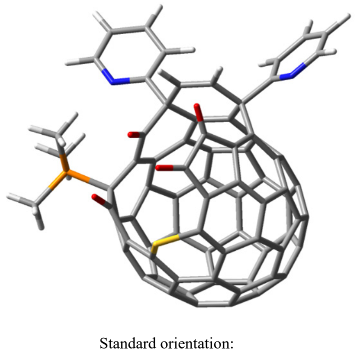

Standard orientation:

Center Number	Atomic Number	Atomic Type	Coordinates (Angstroms) X	Y	Z
1	6	0	3.780056	0.879458	1.105563
2	6	0	3.919717	-0.430153	1.284337
3	6	0	3.195815	-1.485304	0.490414
4	6	0	2.092732	-2.175774	1.311161
5	6	0	1.707598	-1.791234	2.585533
6	6	0	2.125788	-0.758199	3.613718
7	6	0	0.799511	-0.294499	4.330099
8	6	0	-0.269918	-1.188227	3.810770
9	6	0	-1.624414	-0.992142	3.623670
10	6	0	-2.933333	0.967978	2.532827
11	6	0	-2.452384	2.087423	1.879733
12	6	0	-1.032635	2.661961	1.993710
13	6	0	-0.682464	3.056615	0.536460
14	6	0	0.558792	2.552501	-0.123585
15	6	0	1.849329	2.409546	0.632441
16	6	0	2.952420	1.497782	0.014889
17	16	0	-2.304808	0.561544	4.138015
18	7	0	3.258734	3.524263	-1.304228
19	8	0	3.205293	-0.385940	3.980209
20	8	0	2.049552	2.994236	1.665998
21	7	0	4.389708	-3.573130	0.839640
22	6	0	2.319448	0.464167	-0.883877
23	6	0	1.727906	-1.815944	-1.526130
24	8	0	0.752790	0.587501	5.140668
25	6	0	1.389488	0.972541	-1.864178
26	6	0	1.246525	-3.102795	0.552903
27	8	0	-0.371282	2.767414	2.984612
28	6	0	0.115013	-3.658868	-1.586529
29	6	0	5.316986	-4.497200	0.546051
30	6	0	-0.989114	-4.261829	-0.866191
31	6	0	4.236922	-2.521492	0.044782

32	6	0	1.173739	-3.019879	-0.913043
33	6	0	1.163295	-1.320794	-2.696927
34	6	0	2.470505	-0.888703	-0.705944
35	6	0	-0.976189	-4.239236	0.506082
36	6	0	0.075089	-1.982539	-3.365689
37	6	0	-0.317375	-3.157505	2.423651
38	6	0	-0.444494	-3.134076	-2.816058
39	6	0	0.982554	0.102051	-2.863961
40	6	0	0.517743	2.091490	-1.501803
41	6	0	0.165987	-3.715540	1.199205
42	6	0	5.130560	2.033197	-1.202722
43	6	0	3.834534	2.405406	-0.862925
44	6	0	-3.304622	-3.478841	0.565115
45	6	0	-0.654777	2.265162	-2.247359
46	6	0	-0.224539	0.306112	-3.627340
47	6	0	5.848069	2.885530	-2.037236
48	6	0	0.402092	-2.186414	3.047396
49	6	0	-1.884471	-3.330731	-2.810454
50	6	0	6.134226	-4.369232	-0.577729
51	6	0	-4.018580	-2.276964	0.958900
52	6	0	-2.728300	-2.355656	-3.328636
53	6	0	-1.746003	-3.062611	2.367904
54	6	0	5.253187	4.053028	-2.492575
55	6	0	5.016276	-2.308418	-1.093723
56	6	0	-2.395072	-1.886020	2.777619
57	6	0	3.939215	4.346751	-2.104661
58	6	0	-0.795717	-0.980697	-3.942797
59	6	0	-4.632661	-1.769623	-0.250119
60	6	0	-4.384564	0.471437	0.639461
61	6	0	-2.162366	-3.834418	1.235625
62	6	0	-1.865779	2.683595	-1.600145
63	6	0	-2.222433	-4.003160	-1.577977
64	6	0	-3.923055	-1.971955	-2.599771
65	6	0	-3.549588	-1.428276	1.983166
66	6	0	5.979420	-3.256895	-1.401593
67	6	0	-1.841179	2.889370	-0.235914
68	6	0	-3.774760	0.017853	1.819432
69	6	0	-4.211512	-2.576127	-1.382814
70	6	0	-3.363702	-3.624910	-0.873501
71	6	0	-2.927327	2.394938	0.578365
72	6	0	-2.172355	-1.154405	-3.924667
73	6	0	-3.031895	-0.035764	-3.588153
74	6	0	-1.035649	1.378824	-3.325090
75	6	0	-4.573013	0.216168	-1.694198
76	6	0	-2.475822	1.210157	-3.308683
77	6	0	-2.990939	2.013275	-2.224203
78	6	0	-3.965768	1.696680	0.006299
79	6	0	-4.812965	-0.415867	-0.409461
80	6	0	-4.021559	1.522861	-1.430766
81	6	0	-4.109226	-0.541680	-2.762154
82	1	0	4.289158	1.576236	1.764564
83	1	0	4.541420	-0.804136	2.090796
84	1	0	5.431070	-5.393746	1.210937
85	1	0	5.557304	1.113211	-0.817163
86	1	0	6.865071	2.639938	-2.327020
87	1	0	6.879602	-5.119388	-0.811177
88	1	0	5.799018	4.731774	-3.139199
89	1	0	4.859315	-1.430858	-1.713090
90	1	0	3.455180	5.292644	-2.465048
91	1	0	6.606500	-3.136688	-2.279686
92	15	0	-0.790229	4.739047	0.378007
93	6	0	-2.106117	5.816032	1.123267
94	6	0	-0.778308	5.462348	-1.331613
95	6	0	0.698058	5.614843	1.058923
96	1	0	-2.151759	5.646791	2.202106
97	1	0	-3.079241	5.551940	0.701529
98	1	0	-1.917440	6.879008	0.939546
99	1	0	0.063684	5.054575	-1.896763
100	1	0	-1.697444	5.184154	-1.853533
101	1	0	-0.697629	6.554351	-1.314419
102	1	0	0.653247	6.695067	0.884426
103	1	0	1.603109	5.218725	0.591303
104	1	0	0.770368	5.433434	2.134313

The total electronic energy was calculated to be –4094.1877273 Hartree

Supplementary Table 3. Optimized structure of **2'-B** (M06-2X/6-31G(d,p))

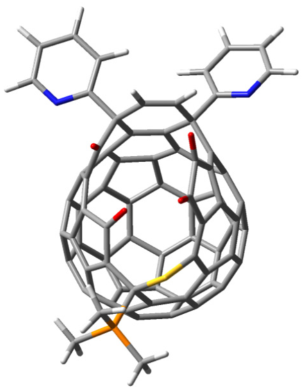

Standard orientation:

Center Number	Atomic Number	Atomic Type	Coordinates (Angstroms) X	Y	Z
1	6	0	3.570924	-2.418242	-0.417516
2	6	0	3.816356	-1.570260	-1.411114
3	6	0	3.452297	-0.109085	-1.400148
4	6	0	2.266894	0.201697	-2.330419
5	6	0	1.524682	-0.757505	-3.000698
6	6	0	1.538144	-2.264733	-3.165898
7	6	0	0.026793	-2.707696	-3.248559
8	6	0	-0.758434	-1.445576	-3.299586
9	6	0	-2.033129	-1.135306	-2.867150
10	6	0	-3.477428	-1.727008	-0.633485
11	6	0	-2.750141	-2.205532	0.531538
12	6	0	-1.486684	-3.071756	0.641554
13	6	0	-0.746986	-2.453297	1.855208
14	6	0	0.595499	-2.121974	1.979458
15	6	0	1.656644	-2.798339	1.158484

16	6	0	2.982425	-2.018663	0.905692
17	16	0	-3.004152	-2.446772	-2.175343
18	7	0	3.370909	-2.837241	3.168331
19	8	0	2.434846	-3.042804	-3.335465
20	8	0	1.513530	-3.913757	0.727726
21	7	0	4.758629	0.955970	-3.151008
22	6	0	2.729862	-0.533959	0.998747
23	6	0	2.601011	1.729298	0.091893
24	8	0	-0.331721	-3.850900	-3.289536
25	6	0	2.028140	-0.091406	2.180268
26	6	0	1.761000	1.575695	-2.249234
27	8	0	-1.119030	-3.916634	-0.120947
28	6	0	1.290929	3.572658	-0.849993
29	6	0	5.834229	1.595016	-3.635415
30	6	0	0.119752	3.877874	-1.647947
31	6	0	4.689508	0.677189	-1.855405
32	6	0	2.056977	2.413349	-1.077951
33	6	0	2.300110	2.203086	1.364571
34	6	0	2.991635	0.344359	-0.023392
35	6	0	-0.222301	3.032007	-2.673286
36	6	0	1.507311	3.384310	1.577926
37	6	0	-0.211868	0.891440	-3.509417
38	6	0	1.006417	4.058035	0.485544
39	6	0	1.998668	1.272836	2.427032
40	6	0	0.964762	-0.933517	2.731066
41	6	0	0.632598	1.926823	-3.000330
42	6	0	5.319262	-2.248089	1.905740
43	6	0	3.944439	-2.403008	2.045548
44	6	0	-2.561970	3.015995	-1.945976
45	6	0	0.010435	-0.316858	3.549484
46	6	0	1.012100	1.886051	3.282668
47	6	0	6.118945	-2.574569	2.997354
48	6	0	0.201654	-0.402612	-3.445086
49	6	0	-0.352481	4.571488	0.530277
50	6	0	6.890716	1.965305	-2.802433
51	6	0	-3.492025	2.051901	-1.384878
52	6	0	-1.146102	4.360818	1.651489
53	6	0	-1.579176	1.218231	-3.232346
54	6	0	5.524870	-3.032297	4.164328
55	6	0	5.703210	1.001604	-0.951994
56	6	0	-2.448043	0.247111	-2.707446
57	6	0	4.129929	-3.152170	4.219493
58	6	0	0.699705	3.195721	2.764202
59	6	0	-3.832040	2.552096	-0.069426
60	6	0	-4.091290	0.257387	0.667023
61	6	0	-1.595583	2.589891	-2.820790
62	6	0	-1.367191	-0.713411	3.476041
63	6	0	-0.911754	4.425424	-0.793073
64	6	0	-2.524900	3.937286	1.491977
65	6	0	-3.406691	0.673576	-1.671727
66	6	0	6.819802	1.658546	-1.445642
67	6	0	-1.717987	-1.696069	2.573047
68	6	0	-3.754389	-0.264928	-0.591395
69	6	0	-3.040914	3.736244	0.218192
70	6	0	-2.232348	4.007019	-0.944146
71	6	0	-2.909036	-1.534100	1.771550
72	6	0	-0.604411	3.668085	2.806488
73	6	0	-1.649418	2.838643	3.375169
74	6	0	0.027842	1.099606	3.841871
75	6	0	-3.643216	1.902518	2.291650
76	6	0	-1.337375	1.585426	3.896435
77	6	0	-2.205860	0.456856	3.654562
78	6	0	-3.674505	-0.396123	1.882226
79	6	0	-4.126735	1.669398	0.942777
80	6	0	-3.338792	0.614132	2.864654
81	6	0	-2.833634	3.000002	2.556352
82	1	0	3.806599	-3.472123	-0.530476
83	1	0	4.250845	-1.931185	-2.337238
84	1	0	5.880916	1.844485	-4.728333
85	1	0	5.740232	-1.892754	0.971041
86	1	0	7.198129	-2.474263	2.935018
87	1	0	7.755549	2.483027	-3.198471
88	1	0	6.132588	-3.296542	5.023166
89	1	0	5.606014	0.750080	0.099506
90	1	0	3.644446	-3.527615	5.158765
91	1	0	7.631865	1.935517	-0.780386
92	15	0	-4.938250	-2.381467	-0.539902
93	6	0	-5.352450	-4.171167	-0.271046
94	6	0	-5.922033	-2.101077	-2.088987
95	6	0	-6.125345	-1.639873	0.679321
96	1	0	-4.983968	-4.490456	0.707165
97	1	0	-4.856899	-4.780939	-1.030771
98	1	0	-6.431243	-4.353198	-0.319420
99	1	0	-5.932909	-1.034795	-2.328879
100	1	0	-5.449964	-2.626671	-2.922959
101	1	0	-6.954510	-2.451977	-1.988327
102	1	0	-7.139536	-2.034092	0.555436
103	1	0	-6.150489	-0.554387	0.553675
104	1	0	-5.786088	-1.851041	1.696627

The total electronic energy was calculated to be –4094.1633566 Hartree.

Supplementary Table 4. Optimized structure of **2'-C** (M06-2X/6-31G(d,p))

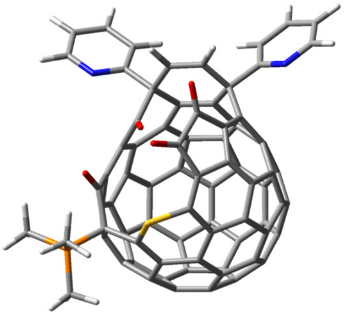

Standard orientation:

Center Number	Atomic Number	Atomic Type	Coordinates (Angstroms) X	Y	Z
1	6	0	3.222847	-2.590491	-0.589517
2	6	0	3.665452	-1.632757	-1.397845
3	6	0	3.465937	-0.157880	-1.168445
4	6	0	2.428629	0.445174	-2.131505
5	6	0	1.661114	-0.292771	-3.018398
6	6	0	1.525642	-1.743668	-3.437630
7	6	0	-0.007781	-1.972571	-3.726501
8	6	0	-0.638793	-0.629289	-3.628789
9	6	0	-1.910363	-0.233030	-3.262451
10	6	0	-3.311579	-0.908030	-1.343251
11	6	0	-3.159945	-1.812158	-0.187868
12	6	0	-1.960166	-2.758379	-0.103404
13	6	0	-1.290978	-2.440473	1.258112
14	6	0	0.058805	-2.306103	1.553933
15	6	0	1.120555	-2.971654	0.724942
16	6	0	2.543742	-2.336021	0.726134
17	16	0	-3.088781	-1.504356	-2.893005
18	7	0	2.594810	-3.547969	2.840158
19	8	0	2.343894	-2.591970	-3.659145
20	8	0	0.902093	-3.976717	0.098593
21	7	0	5.061904	0.998833	-2.590155
22	6	0	2.448203	-0.865349	1.049850
23	6	0	2.668004	1.511520	0.536528
24	8	0	-0.483902	-3.039781	-3.993896
25	6	0	1.677148	-0.531831	2.223945
26	6	0	2.071818	1.841468	-1.860508
27	8	0	-1.608524	-3.510344	-0.964387
28	6	0	1.678016	3.632620	-0.186173
29	6	0	6.247169	1.565486	-2.862181
30	6	0	0.639975	4.207293	-1.019099
31	6	0	4.824027	0.527770	-1.372535
32	6	0	2.331317	2.436971	-0.541718
33	6	0	2.286682	1.810257	1.840398
34	6	0	2.913361	0.124570	0.220137
35	6	0	0.318408	3.586283	-2.200096
36	6	0	1.610934	3.033084	2.183034
37	6	0	0.181862	1.622877	-3.386535
38	6	0	1.307435	3.930589	1.182797
39	6	0	1.772271	0.768086	2.697320
40	6	0	0.474420	-1.309436	2.527390
41	6	0	1.076095	2.448184	-2.635851
42	6	0	4.720285	-3.016509	1.872057
43	6	0	3.329746	-3.016249	1.862774
44	6	0	-2.073081	3.751003	-1.693204
45	6	0	-0.488106	-0.715467	3.352958
46	6	0	0.773522	1.356826	3.556008
47	6	0	5.357450	-3.611809	2.957111
48	6	0	0.440643	0.293319	-3.509090
49	6	0	0.016637	4.598347	1.196141
50	6	0	7.242742	1.661073	-1.889126
51	6	0	-3.158650	2.835435	-1.388925
52	6	0	-0.910909	4.313640	2.191095
53	6	0	-1.162751	2.071856	-3.177912
54	6	0	4.594883	-4.170983	3.972042
55	6	0	5.764713	0.572622	-0.341960
56	6	0	-2.184758	1.147436	-2.905021
57	6	0	3.197404	-4.120347	3.883947
58	6	0	0.665038	2.761377	3.244401
59	6	0	-3.580285	3.158152	-0.041968
60	6	0	-4.168785	0.826894	0.264883
61	6	0	-1.071551	3.351065	-2.540410
62	6	0	-1.885258	-0.917357	3.092752
63	6	0	-0.410349	4.737564	-0.176330
64	6	0	-2.302980	4.099232	1.841700
65	6	0	-3.195838	1.521051	-1.899177
66	6	0	6.993448	1.153077	-0.616411
67	6	0	-2.243679	-1.690713	2.007613
68	6	0	-3.758780	0.473808	-1.030168
69	6	0	-2.698603	4.171228	0.512221
70	6	0	-1.745246	4.519451	-0.511487
71	6	0	-3.316797	-1.253194	1.144928
72	6	0	-0.575907	3.382357	3.253082
73	6	0	-1.760999	2.611971	3.577849
74	6	0	-0.345781	0.622348	3.884143
75	6	0	-3.718379	2.121240	2.180200
76	6	0	-1.646927	1.262285	3.901847
77	6	0	-2.603810	0.306119	3.395326
78	6	0	-3.959318	-0.059773	1.381775
79	6	0	-4.077393	2.169704	0.774372
80	6	0	-3.621263	0.729826	2.548280
81	6	0	-2.825578	3.050792	2.698660
82	1	0	3.351416	-3.634068	-0.860410
83	1	0	4.154111	-1.893276	-2.330633
84	1	0	6.437930	1.978327	-3.887878
85	1	0	5.275616	-2.572729	1.052418
86	1	0	6.441539	-3.640436	3.007270
87	1	0	8.196883	2.121552	-2.113608
88	1	0	5.074179	-4.643853	4.822583
89	1	0	5.528254	0.170785	0.638335
90	1	0	2.575618	-4.576368	4.699000
91	1	0	7.755168	1.215069	0.154735
92	15	0	-4.255966	-3.293638	-0.462246
93	6	0	-3.789412	-5.077607	-0.678243
94	6	0	-5.473553	-3.156882	-1.856839
95	6	0	-5.459537	-3.493179	0.936775
96	1	0	-3.180978	-5.403290	0.169320
97	1	0	-3.189865	-5.193232	-1.584746
98	1	0	-4.669792	-5.724813	-0.751211
99	1	0	-5.990889	-2.195793	-1.799439
100	1	0	-4.942820	-3.194479	-2.811572

101	1	0	-6.216574	-3.961010	-1.832954
102	1	0	-6.199484	-4.274241	0.732428
103	1	0	-5.981568	-2.548562	1.109636
104	1	0	-4.919306	-3.749075	1.851798

The total electronic energy was calculated to be –4094.1590937 Hartree.

Supplementary Table 5. Optimized structure of **2'-D** (M06-2X/6-31G(d,p))

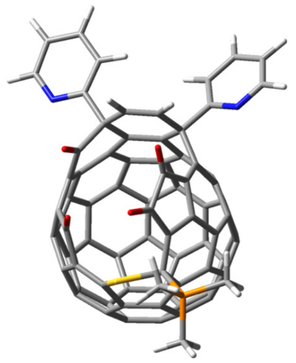

Standard orientation:

Center Number	Atomic Number	Atomic Type	Coordinates (Angstroms) X	Y	Z
1	6	0	-3.373993	-2.185111	-1.260922
2	6	0	-3.096930	-2.699328	-0.068097
3	6	0	-2.608817	-1.905363	1.113706
4	6	0	-1.126435	-2.177612	1.412537
5	6	0	-0.296417	-2.936617	0.617691
6	6	0	-0.387696	-3.787379	-0.641461
7	6	0	1.013683	-3.670376	-1.360887
8	6	0	1.859315	-2.974419	-0.439048
9	6	0	3.188001	-2.278862	-0.629592
10	6	0	3.015146	-0.045272	-2.279777
11	6	0	2.038263	0.498344	-3.096176
12	6	0	0.739868	-0.168517	-3.575911
13	6	0	-0.320364	0.936260	-3.334938
14	6	0	-1.569318	0.825371	-2.741840
15	6	0	-2.327026	-0.468056	-2.741232
16	6	0	-3.329911	-0.716494	-1.576676
17	16	0	3.369189	-1.757042	-2.408315
18	7	0	-4.725384	0.690145	-3.009918
19	8	0	-1.238329	-4.544321	-1.022792
20	8	0	-2.193308	-1.284119	-3.617984
21	7	0	-3.055229	-3.310979	3.059081
22	6	0	-2.949920	0.122735	-0.379609
23	6	0	-2.196281	0.442689	1.922851
24	8	0	1.224253	-4.178411	-2.450890
25	6	0	-2.712031	1.522032	-0.642703
26	6	0	-0.546506	-1.332764	2.471117
27	8	0	0.590236	-1.307878	-3.930421
28	6	0	-0.424234	0.891071	3.564090
29	6	0	-3.829166	-3.710538	4.070945
30	6	0	0.981856	0.706663	3.815673
31	6	0	-3.483053	-2.292921	2.313476
32	6	0	-1.164069	-0.065322	2.829753
33	6	0	-2.358030	1.814466	1.766472
34	6	0	-2.674954	-0.406032	0.857441
35	6	0	1.594190	-0.441571	3.370432
36	6	0	-1.576478	2.773816	2.505866
37	6	0	1.684211	-2.180454	1.855307
38	6	0	-0.618428	2.314785	3.383053
39	6	0	-2.605191	2.364725	0.454649
40	6	0	-2.048443	1.902175	-1.890703
41	6	0	0.822070	-1.476097	2.746512
42	6	0	-5.876587	-0.745389	-1.472498
43	6	0	-4.713237	-0.243068	-2.058627
44	6	0	3.473106	0.842269	2.458210
45	6	0	-1.400247	3.142748	-1.928224
46	6	0	-1.952639	3.649770	0.387215
47	6	0	-7.100883	-0.254953	-1.908134
48	6	0	1.133673	-2.762261	0.744806
49	6	0	0.667783	2.990080	3.448633
50	6	0	-5.052208	-3.127313	4.383227
51	6	0	4.003342	1.126284	1.152595
52	6	0	0.941664	4.062466	2.606389
53	6	0	2.876596	-1.371420	1.661715
54	6	0	-7.122548	0.713808	-2.906378
55	6	0	-4.702400	-1.647736	2.535918
56	6	0	3.438069	-1.153190	0.397797
57	6	0	-5.907520	1.151785	-3.421692
58	6	0	-1.309608	3.910765	1.654355
59	6	0	3.854214	2.555613	0.969275
60	6	0	3.534319	2.157663	-1.395291
61	6	0	2.861285	-0.369159	2.680447
62	6	0	-0.150145	3.281416	-2.619339
63	6	0	1.669398	1.978809	3.685524
64	6	0	2.218721	4.133541	1.922034
65	6	0	3.889579	0.194112	0.082743
66	6	0	-5.496962	-2.073465	3.591025
67	6	0	0.395115	2.161262	-3.212333
68	6	0	3.656018	0.767573	-1.261809
69	6	0	3.157313	3.125168	2.110025
70	6	0	2.893511	2.045966	3.026760
71	6	0	1.805341	1.894355	-3.061210
72	6	0	-0.075909	4.546985	1.693233
73	6	0	0.568424	4.935871	0.453862
74	6	0	-1.355338	4.037762	-0.793238
75	6	0	2.701760	4.163497	-0.483948
76	6	0	-0.064710	4.700502	-0.764584
77	6	0	0.692515	4.213217	-1.892815
78	6	0	2.606410	2.741158	-2.330279
79	6	0	3.635526	3.071374	-0.284791
80	6	0	2.050299	3.946710	-1.752919
81	6	0	1.986110	4.670981	0.594263
82	1	0	-3.651852	-2.834530	-2.085547
83	1	0	-3.144196	-3.772065	0.084309
84	1	0	-3.448137	-4.540876	4.661151
85	1	0	-5.808283	-1.505829	-0.701476
86	1	0	-8.025347	-0.626401	-1.477071
87	1	0	-5.636080	-3.490895	5.221220

88	1	0	-8.055174	1.120849	-3.280114
89	1	0	-5.008262	-0.826638	1.894920
90	1	0	-5.876660	1.907538	-4.202851
91	1	0	-6.447101	-1.589134	3.793586
92	15	0	4.523334	-3.549157	-0.441361
93	6	0	6.168238	-2.807932	-0.667226
94	6	0	4.231081	-4.874656	-1.647920
95	6	0	4.442868	-4.216630	1.238716
96	1	0	6.121915	-1.772899	-0.315963
97	1	0	6.436767	-2.810736	-1.723875
98	1	0	6.915420	-3.355277	-0.088671
99	1	0	4.217613	-5.841739	-1.141734
100	1	0	3.260236	-4.710125	-2.138201
101	1	0	5.025203	-4.868072	-2.396419
102	1	0	5.041556	-5.128609	1.296274
103	1	0	3.399222	-4.439263	1.476926
104	1	0	4.822087	-3.475786	1.944465

The total electronic energy was calculated to be –4094.1635411 Hartree.

Supplementary Table 6. Optimized structure of **2'-E** (M06-2X/6-31G(d,p))

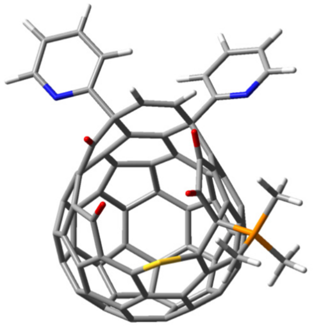

Standard orientation:

Center Number	Atomic Number	Atomic Type	Coordinates (Angstroms) X	Y	Z
1	6	0	3.642561	1.332513	-1.323370
2	6	0	3.433442	2.028709	-0.210660
3	6	0	2.748275	1.493501	1.018889
4	6	0	1.334566	2.074241	1.195909
5	6	0	0.700746	2.888606	0.271083
6	6	0	1.029564	3.526736	-1.064536
7	6	0	-0.308307	3.504697	-1.899306
8	6	0	-1.409329	3.132681	-0.934713
9	6	0	-2.602361	2.342542	-1.222307
10	6	0	-3.008554	0.186255	-2.573677
11	6	0	-2.128278	-0.629113	-3.260185
12	6	0	-0.707890	-0.253736	-3.707800
13	6	0	0.135208	-1.467157	-3.239402
14	6	0	1.350545	-1.475194	-2.568305
15	6	0	2.310944	-0.323119	-2.656936
16	6	0	3.313644	-0.124918	-1.480160
17	16	0	-2.963756	1.935919	-2.844841
18	7	0	4.418805	-1.914878	-2.711091
19	8	0	2.016440	4.076653	-1.466891
20	8	0	2.335737	0.412897	-3.609751
21	7	0	3.303664	2.996698	2.845990
22	6	0	2.747681	-0.726559	-0.217422
23	6	0	1.872904	-0.623313	2.059731
24	8	0	-0.385612	3.851191	-3.044336
25	6	0	2.267842	-2.083419	-0.331204
26	6	0	0.553440	1.478703	2.284746
27	8	0	-0.346317	0.787076	-4.172958
28	6	0	-0.022972	-0.541367	3.609047
29	6	0	4.048194	3.416323	3.880173
30	6	0	-1.390039	-0.080174	3.750374
31	6	0	3.617181	1.864596	2.228627
32	6	0	0.916884	0.164109	2.833310
33	6	0	1.784962	-2.011437	2.069976
34	6	0	2.539886	-0.010793	0.935504
35	6	0	-1.759684	1.093382	3.142145
36	6	0	0.811226	-2.714538	2.861700
37	6	0	-1.446062	2.560124	1.401009
38	6	0	-0.083053	-1.989297	3.618265
39	6	0	1.977418	-2.754590	0.846972
40	6	0	1.598807	-2.498215	-1.565561
41	6	0	-0.772899	1.888270	2.468610
42	6	0	5.808761	-0.613372	-1.258019
43	6	0	4.585054	-0.913510	-1.846359
44	6	0	-3.782550	0.046350	2.239357
45	6	0	0.752435	-3.611801	-1.499239
46	6	0	1.110496	-3.906976	0.890739
47	6	0	6.902066	-1.401259	-1.606761
48	6	0	-0.735641	2.979474	0.319733
49	6	0	-1.472594	-2.411689	3.673016
50	6	0	5.156203	2.688261	4.315350
51	6	0	-4.294796	-0.317841	0.930052
52	6	0	-1.899191	-3.518103	2.948312
53	6	0	-2.743348	1.979026	1.220823
54	6	0	6.732693	-2.439669	-2.510801
55	6	0	4.712755	1.078592	2.590434
56	6	0	-3.205078	1.660641	-0.067183
57	6	0	5.461342	-2.675151	-3.050697
58	6	0	0.381955	-3.891011	2.135972
59	6	0	-4.415467	-1.760589	0.941626
60	6	0	-3.939692	-1.756451	-1.435110
61	6	0	-2.983451	1.153639	2.366330
62	6	0	-0.468306	-3.635974	-2.254028
63	6	0	-2.283930	-1.218024	3.720703
64	6	0	-3.135439	-3.453447	2.191067
65	6	0	-3.958140	0.404175	-0.233864
66	6	0	5.489411	1.507661	3.655678
67	6	0	-0.784338	-2.534376	-3.022583
68	6	0	-3.802960	-0.360404	-1.482919
69	6	0	-3.881107	-2.281617	2.188142
70	6	0	-3.464568	-1.153616	2.983274
71	6	0	-2.137128	-2.027260	-3.017690
72	6	0	-0.946909	-4.289258	2.169974
73	6	0	-1.597329	-4.721880	0.948008

74	6	0	0.504812	-4.334807	-0.271188
75	6	0	-3.514395	-3.728316	-0.219582
76	6	0	-0.881777	-4.758557	-0.246262
77	6	0	-1.491038	-4.310346	-1.476851
78	6	0	-3.099709	-2.616277	-2.230492
79	6	0	-4.240025	-2.470920	-0.222507
80	6	0	-2.785219	-3.803296	-1.462010
81	6	0	-2.947724	-4.197303	0.958933
82	1	0	4.086060	1.812373	-2.190556
83	1	0	3.704112	3.078345	-0.168759
84	1	0	3.769408	4.364897	4.410616
85	1	0	5.895451	0.213219	-0.560613
86	1	0	7.879103	-1.203137	-1.176959
87	1	0	5.752339	3.030533	5.152183
88	1	0	7.574425	-3.060604	-2.798337
89	1	0	4.934488	0.161315	2.053964
90	1	0	5.319785	-3.509865	-3.786970
91	1	0	6.348915	0.927143	3.976578
92	15	0	-2.187282	4.776306	-0.529677
93	6	0	-3.549790	5.340801	-1.657411
94	6	0	-1.062985	6.243565	-0.700631
95	6	0	-2.973989	5.218554	1.092411
96	1	0	-4.404198	4.665076	-1.567947
97	1	0	-3.205168	5.309246	-2.694144
98	1	0	-3.878204	6.359200	-1.424435
99	1	0	-0.251451	6.170931	0.027900
100	1	0	-0.617069	6.249138	-1.698495
101	1	0	-1.594863	7.188154	-0.545290
102	1	0	-3.353917	6.245674	1.096981
103	1	0	-2.242625	5.108862	1.897255
104	1	0	-3.801741	4.534820	1.297191

The total electronic energy was calculated to be –4094.1563644 Hartree.

Supplementary Table 7. Optimized structure of **TS-A** (M06-2X/6-31G(d,p))

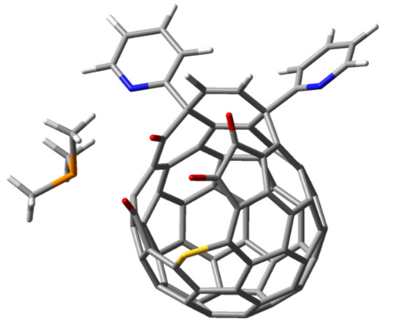

Standard orientation:

Center Number	Atomic Number	Atomic Type	Coordinates (Angstroms) X	Y	Z
1	6	0	-3.499207	1.531636	0.903116
2	6	0	-2.912777	2.705815	1.111266
3	6	0	-1.649303	3.157913	0.426566
4	6	0	-0.420073	3.024336	1.341705
5	6	0	-0.447555	2.427737	2.591282
6	6	0	-1.483750	1.809654	3.510124
7	6	0	-0.769413	0.592831	4.209801
8	6	0	0.662371	0.690640	3.820910
9	6	0	1.642180	-0.271146	3.669735
10	6	0	1.637944	-2.593294	2.482001
11	6	0	0.667028	-3.223524	1.727489
12	6	0	-0.828815	-3.125883	1.808610
13	6	0	-1.249632	-2.900403	0.355250
14	6	0	-1.779610	-1.666520	-0.293378
15	6	0	-2.543432	-0.774528	0.534301
16	6	0	-3.052968	0.538428	-0.134267
17	16	0	1.208903	-1.944041	4.072546
18	7	0	-4.829688	-0.985577	-0.939497
19	8	0	-2.584572	2.164662	3.826634
20	8	0	-2.987098	-1.071179	1.639885
21	7	0	-1.608574	5.520820	0.980581
22	6	0	-1.937917	1.074388	-0.992661
23	6	0	-0.116877	2.605789	-1.493921
24	8	0	-1.329323	-0.189082	4.927372
25	6	0	-1.413527	0.142151	-1.955200
26	6	0	0.868292	3.279901	0.689944
27	8	0	-1.523224	-3.291106	2.775448
28	6	0	2.272604	3.111761	-1.353384
29	6	0	-1.822901	6.806984	0.700976
30	6	0	3.456817	2.905617	-0.544515
31	6	0	-1.840164	4.624398	0.020260
32	6	0	0.993044	3.220333	-0.774675
33	6	0	0.131124	1.895886	-2.664931
34	6	0	-1.317520	2.276557	-0.765231
35	6	0	3.323253	2.843149	0.820701
36	6	0	1.443946	1.796380	-3.240525
37	6	0	1.996416	2.298839	2.616959
38	6	0	2.502514	2.395119	-2.592838
39	6	0	-0.538270	0.636930	-2.904391
40	6	0	-1.385081	-1.290713	-1.606601
41	6	0	2.044633	3.087747	1.424771
42	6	0	-4.785617	1.229567	-1.843475
43	6	0	-4.270991	0.223216	-1.019148
44	6	0	4.729353	0.840210	0.915133
45	6	0	-0.594785	-2.130264	-2.434264
46	6	0	0.347196	-0.211981	-3.664054
47	6	0	-5.900902	0.960571	-2.620465
48	6	0	0.790698	1.929878	3.129500
49	6	0	3.776265	1.696430	-2.516998
50	6	0	-2.277786	7.261547	-0.532943
51	6	0	4.556908	-0.563151	1.241114
52	6	0	3.907595	0.428204	-3.071884
53	6	0	3.084280	1.366334	2.609324
54	6	0	-6.481353	-0.303843	-2.547441
55	6	0	-2.305875	4.976921	-1.247958
56	6	0	2.865718	0.018259	2.944601
57	6	0	-5.910661	-1.232250	-1.689046
58	6	0	1.586680	0.486509	-3.850961
59	6	0	4.855090	-1.289630	0.023206
60	6	0	3.257821	-2.975709	0.711339
61	6	0	3.972204	1.781360	1.564789

62	6	0	0.112146	-3.212185	-1.770328
63	6	0	4.346160	1.985420	-1.224277
64	6	0	4.589769	-0.615928	-2.328911
65	6	0	3.588541	-1.003918	2.167294
66	6	0	-2.525539	6.320117	-1.526032
67	6	0	-0.151142	-3.544484	-0.459788
68	6	0	2.930985	-2.291536	1.893170
69	6	0	5.086318	-0.347708	-1.058054
70	6	0	4.985472	0.977707	-0.502850
71	6	0	0.963265	-3.694638	0.418956
72	6	0	2.803305	-0.184813	-3.785012
73	6	0	2.800792	-1.600167	-3.491842
74	6	0	0.332267	-1.568238	-3.380811
75	6	0	3.764291	-2.801857	-1.576703
76	6	0	1.596148	-2.282056	-3.320527
77	6	0	1.464162	-3.280445	-2.291189
78	6	0	2.269915	-3.704257	-0.036238
79	6	0	4.221110	-2.480166	-0.237300
80	6	0	2.531680	-3.542589	-1.437849
81	6	0	3.910225	-1.869979	-2.593484
82	15	0	-3.209862	-4.517585	0.090211
83	6	0	-2.798151	-6.224014	0.633501
84	6	0	-3.774163	-4.663070	-1.663126
85	6	0	-4.684778	-3.987837	1.040649
86	1	0	-4.355105	1.239798	1.503850
87	1	0	-3.285170	3.372454	1.882010
88	1	0	-1.619345	7.509291	1.505998
89	1	0	-4.307062	2.204069	-1.853376
90	1	0	-6.315431	1.724096	-3.271097
91	1	0	-2.429909	8.320883	-0.705796
92	1	0	-7.355202	-0.562375	-3.134338
93	1	0	-2.475501	4.213498	-1.999860
94	1	0	-6.339088	-2.228236	-1.589697
95	1	0	-2.881669	6.626678	-2.504611
96	1	0	-2.578522	-6.180170	1.702681
97	1	0	-1.904755	-6.541551	0.089840
98	1	0	-3.608347	-6.934230	0.454902
99	1	0	-4.068294	-3.657143	-1.975593
100	1	0	-2.933609	-5.003344	-2.271538
101	1	0	-4.616732	-5.350668	-1.768309
102	1	0	-5.470758	-4.747471	0.999849
103	1	0	-5.026726	-3.045393	0.602954
104	1	0	-4.349653	-3.807516	2.062734

The total electronic energy was calculated to be –4094.1656415 Hartree.

An imaginary frequency was found at 229.7464 cm^{-1}.

Supplementary Table 8. Optimized structure of **TS-B** (M06-2X/6-31G(d,p))

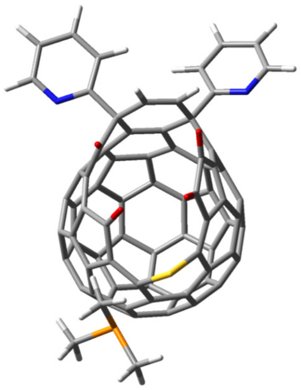

Standard orientation:

Center Number	Atomic Number	Atomic Type	Coordinates (Angstroms) X	Y	Z
1	6	0	3.597430	-2.513965	-0.296218
2	6	0	3.818683	-1.731964	-1.345757
3	6	0	3.471898	-0.268914	-1.403091
4	6	0	2.245857	-0.004949	-2.291503
5	6	0	1.435846	-0.991125	-2.838732
6	6	0	1.375679	-2.509652	-2.905272
7	6	0	-0.151673	-2.891076	-3.069172
8	6	0	-0.880592	-1.592971	-3.107083
9	6	0	-2.151934	-1.191937	-2.758619
10	6	0	-3.489051	-1.557645	-0.607803
11	6	0	-2.782433	-2.068554	0.555934
12	6	0	-1.464267	-2.703149	0.578315
13	6	0	-0.728747	-2.221422	1.812540
14	6	0	0.622513	-1.982967	2.003673
15	6	0	1.674596	-2.751401	1.268043
16	6	0	3.030257	-2.030834	1.008139
17	16	0	-3.317722	-2.398212	-2.220593
18	7	0	3.429266	-2.793887	3.300476
19	8	0	2.236330	-3.341409	-2.975912
20	8	0	1.514285	-3.893431	0.920831
21	7	0	4.769763	0.532106	-3.298241
22	6	0	2.815514	-0.537198	1.032934
23	6	0	2.698361	1.672162	0.004259
24	8	0	-0.547582	-4.007747	-3.243446
25	6	0	2.122412	-0.022198	2.192172
26	6	0	1.794021	1.389479	-2.298213
27	8	0	-0.944429	-3.370307	-0.246961
28	6	0	1.414962	3.486971	-1.024630
29	6	0	5.851657	1.099211	-3.834981
30	6	0	0.245742	3.787677	-1.827569
31	6	0	4.695828	0.462617	-1.969085
32	6	0	2.146814	2.296103	-1.193645
33	6	0	2.419618	2.219679	1.253488
34	6	0	3.070753	0.277459	-0.040480
35	6	0	-0.140284	2.892449	-2.794648
36	6	0	1.649067	3.425696	1.408269
37	6	0	-0.224772	0.697300	-3.466265
38	6	0	1.159731	4.056036	0.284097
39	6	0	2.110644	1.353955	2.367734
40	6	0	1.026968	-0.810782	2.756773
41	6	0	0.665124	1.731814	-3.050029
42	6	0	5.363937	-2.243667	1.994679
43	6	0	3.984876	-2.391374	2.158415
44	6	0	-2.474717	3.019672	-2.065482
45	6	0	0.072122	-0.137540	3.529453

46	6	0	1.129655	2.025870	3.187818
47	6	0	6.193450	-2.540725	3.067699
48	6	0	0.128279	-0.605830	-3.291541
49	6	0	-0.182153	4.612735	0.311943
50	6	0	6.902957	1.614447	-3.084256
51	6	0	-3.449368	2.132688	-1.456730
52	6	0	-0.973220	4.477072	1.447562
53	6	0	-1.576655	1.108065	-3.236990
54	6	0	5.621700	-2.970468	4.261324
55	6	0	5.702443	0.938689	-1.126383
56	6	0	-2.496504	0.211502	-2.678801
57	6	0	4.236907	-3.077565	4.324250
58	6	0	0.846630	3.319802	2.607521
59	6	0	-3.746928	2.699129	-0.171197
60	6	0	-4.115129	0.445505	0.665500
61	6	0	-1.530470	2.504412	-2.915645
62	6	0	-1.314568	-0.491742	3.440542
63	6	0	-0.757696	4.419790	-0.998090
64	6	0	-2.365164	4.091338	1.317663
65	6	0	-3.441654	0.729012	-1.671756
66	6	0	6.823187	1.526664	-1.698243
67	6	0	-1.679112	-1.460730	2.534565
68	6	0	-3.896935	-0.128645	-0.595016
69	6	0	-2.904395	3.863719	0.059687
70	6	0	-2.096227	4.048736	-1.119883
71	6	0	-2.889609	-1.326550	1.738644
72	6	0	-0.445716	3.822144	2.630521
73	6	0	-1.513812	3.055042	3.245534
74	6	0	0.122295	1.286994	3.769469
75	6	0	-3.536147	2.121807	2.208858
76	6	0	-1.236845	1.804760	3.802299
77	6	0	-2.133978	0.697777	3.614733
78	6	0	-3.703715	-0.204155	1.906495
79	6	0	-4.051871	1.863799	0.882826
80	6	0	-3.265216	0.838488	2.810856
81	6	0	-2.690559	3.206813	2.426159
82	15	0	-5.494124	-2.513436	-0.249133
83	6	0	-5.316062	-4.297800	-0.518539
84	6	0	-6.737009	-1.831433	-1.377156
85	6	0	-6.136886	-2.331427	1.435028
86	1	0	3.805398	-3.577980	-0.353331
87	1	0	4.207411	-2.151604	-2.267528
88	1	0	5.876353	1.142339	-4.921348
89	1	0	5.761350	-1.908447	1.042009
90	1	0	7.270308	-2.440098	2.973659
91	1	0	7.756170	2.069443	-3.574333
92	1	0	6.229993	-3.218335	5.123734
93	1	0	5.597196	0.852385	-0.049499
94	1	0	3.748171	-3.407379	5.237881
95	1	0	7.620859	1.912554	-1.071210
96	1	0	-4.401804	-4.619540	-0.010626
97	1	0	-5.227513	-4.519532	-1.582978
98	1	0	-6.177689	-4.821636	-0.099520
99	1	0	-6.852972	-0.767003	-1.160796
100	1	0	-6.390620	-1.952256	-2.404875
101	1	0	-7.690839	-2.344538	-1.236405
102	1	0	-7.049313	-2.930852	1.504162
103	1	0	-6.353926	-1.286632	1.654375
104	1	0	-5.393180	-2.698691	2.143912

The total electronic energy was calculated to be –4094.1497537 Hartree.

An imaginary frequency was found at 533.1371 cm^{-1}.

Supplementary Table 9. Optimized structure of **TS-C** (M06-2X/6-31G(d,p))

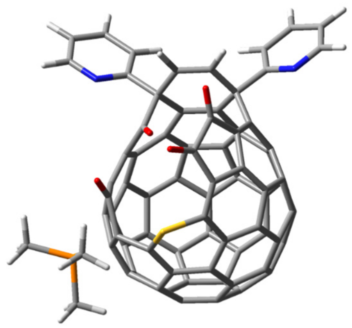

Standard orientation:

Center Number	Atomic Number	Atomic Type	Coordinates (Angstroms) X	Y	Z
1	6	0	-3.123923	2.916993	-0.358832
2	6	0	-3.813493	2.035620	-1.074237
3	6	0	-3.725803	0.542324	-0.889425
4	6	0	-2.806699	-0.125534	-1.922907
5	6	0	-2.010348	0.571806	-2.813984
6	6	0	-1.757313	2.017796	-3.203417
7	6	0	-0.218950	2.111177	-3.545796
8	6	0	0.280165	0.712580	-3.509637
9	6	0	1.520737	0.172889	-3.242502
10	6	0	3.462714	0.569123	-1.468438
11	6	0	3.511489	1.523550	-0.311894
12	6	0	2.297175	2.519523	-0.299243
13	6	0	1.627540	2.161864	1.091457
14	6	0	0.270124	2.117055	1.366754
15	6	0	-0.721200	2.642486	0.433821
16	6	0	-2.208752	2.536615	0.770161
17	16	0	2.904836	1.234010	-3.014321
18	7	0	-1.539666	4.520227	1.991751
19	8	0	-2.515069	2.927933	-3.392690
20	8	0	-0.528783	3.171571	-0.625917
21	7	0	-5.535795	-0.421989	-2.203653
22	6	0	-2.394377	1.097306	1.196008
23	6	0	-2.963214	-1.227469	0.733190
24	8	0	0.349594	3.133491	-3.814614
25	6	0	-1.547745	0.664865	2.278521
26	6	0	-2.566962	-1.555816	-1.701314
27	8	0	2.095258	3.443241	-1.038145
28	6	0	-2.224871	-3.414549	-0.086592
29	6	0	-6.787849	-0.862846	-2.343947
30	6	0	-1.304197	-4.063409	-0.997665
31	6	0	-5.147016	-0.023668	-0.993482
32	6	0	-2.793970	-2.159399	-0.378746
33	6	0	-2.489525	-1.574480	1.994596

34	6	0	-3.080774	0.181590	0.439739
35	6	0	-1.001092	-3.442559	-2.184552
36	6	0	-1.921477	-2.866227	2.279095
37	6	0	-0.743153	-1.460272	-3.314354
38	6	0	-1.782764	-3.771976	1.248630
39	6	0	-1.777977	-0.602179	2.792527
40	6	0	-0.225399	1.276656	2.442400
41	6	0	-1.672801	-2.225490	-2.543988
42	6	0	-3.474696	3.363769	2.814064
43	6	0	-2.406118	3.508805	1.928374
44	6	0	1.391461	-3.858954	-1.842614
45	6	0	0.705454	0.593756	3.240642
46	6	0	-0.788947	-1.304659	3.573350
47	6	0	-3.638610	4.312089	3.813616
48	6	0	-0.875776	-0.108614	-3.373745
49	6	0	-0.574875	-4.576292	1.161598
50	6	0	-7.703765	-0.923684	-1.299466
51	6	0	2.590693	-3.081559	-1.593498
52	6	0	0.449910	-4.411794	2.087006
53	6	0	0.560410	-2.048251	-3.202687
54	6	0	-2.734891	5.368054	3.892783
55	6	0	-5.993543	-0.032484	0.117232
56	6	0	1.685532	-1.243712	-2.953695
57	6	0	-1.706823	5.426102	2.959051
58	6	0	-0.870336	-2.711388	3.262993
59	6	0	3.092573	-3.524377	-0.301470
60	6	0	4.072753	-1.302254	0.004610
61	6	0	0.380183	-3.333181	-2.608318
62	6	0	2.102217	0.632372	2.941999
63	6	0	-0.257401	-4.717497	-0.240405
64	6	0	1.833248	-4.362979	1.656856
65	6	0	2.740808	-1.763808	-2.067990
66	6	0	-7.293210	-0.493682	-0.041297
67	6	0	2.535820	1.354149	1.837834
68	6	0	3.542690	-0.805666	-1.245419
69	6	0	2.128834	-4.416062	0.294135
70	6	0	1.066628	-4.627335	-0.663780
71	6	0	3.609322	0.891801	1.012452
72	6	0	0.291137	-3.466791	3.179171
73	6	0	1.575191	-2.845290	3.430546
74	6	0	0.428242	-0.707434	3.819129
75	6	0	3.493272	-2.568933	1.920260
76	6	0	1.642005	-1.497915	3.773735
77	6	0	2.678676	-0.659451	3.217433
78	6	0	3.994297	-0.426369	1.145304
79	6	0	3.756604	-2.605074	0.487761
80	6	0	3.590122	-1.191121	2.313077
81	6	0	2.525268	-3.396130	2.480694
82	1	0	-3.177332	3.975349	-0.597276
83	1	0	-4.428155	2.370147	-1.903596
84	1	0	-7.069420	-1.182855	-3.344344
85	1	0	-4.152433	2.521998	2.711683
86	1	0	-4.457684	4.230309	4.521053
87	1	0	-8.707388	-1.296185	-1.470329
88	1	0	-2.824231	6.130428	4.658265
89	1	0	-5.629683	0.311795	1.080167
90	1	0	-0.981349	6.235627	2.983526
91	1	0	-7.974441	-0.518539	0.803392
92	15	0	5.184824	2.590258	-0.561055
93	6	0	5.347202	3.458465	-2.186664
94	6	0	6.636746	1.470394	-0.440923
95	6	0	5.434227	3.898337	0.724354
96	1	0	4.433856	4.042704	-2.321881
97	1	0	5.417229	2.704666	-2.974120
98	1	0	6.228513	4.104026	-2.218674
99	1	0	6.703378	1.111157	0.587924
100	1	0	6.410044	0.623267	-1.095701
101	1	0	7.576822	1.938315	-0.740242
102	1	0	6.423562	4.359142	0.685886
103	1	0	5.290992	3.417275	1.695458
104	1	0	4.663198	4.657986	0.577811

The total electronic energy was calculated to be –4094.1494054 Hartree.

An imaginary frequency was found at 550.0241 cm^{-1}.

Supplementary Table 10. Optimized structure of **TS-D** (M06-2X/6-31G(d,p))

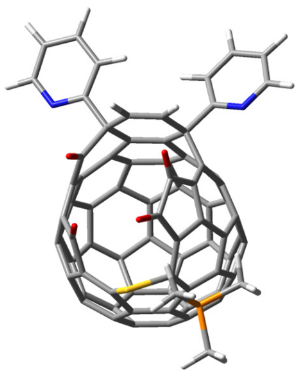

Standard orientation:

Center Number	Atomic Number	Atomic Type	Coordinates (Angstroms) X	Y	Z
1	6	0	-3.307004	-2.291564	-1.262593
2	6	0	-3.010811	-2.797196	-0.070695
3	6	0	-2.550136	-1.987966	1.111768
4	6	0	-1.058762	-2.207828	1.408989
5	6	0	-0.203035	-2.935850	0.612425
6	6	0	-0.265179	-3.787656	-0.647796
7	6	0	1.130527	-3.620045	-1.368238
8	6	0	1.951744	-2.895730	-0.446171
9	6	0	3.144964	-2.055953	-0.651641
10	6	0	3.001266	0.075014	-2.283916
11	6	0	2.004984	0.584694	-3.098763
12	6	0	0.730649	-0.127193	-3.578308
13	6	0	-0.367908	0.938942	-3.334957
14	6	0	-1.611615	0.783017	-2.740963
15	6	0	-2.322943	-0.536486	-2.741464
16	6	0	-3.315348	-0.821893	-1.576404
17	16	0	3.415720	-1.622942	-2.415059
18	7	0	-4.761130	0.536223	-3.006578
19	8	0	-1.088751	-4.573806	-1.029434

20	8	0	-2.161125	-1.346133	-3.619427
21	7	0	-2.944661	-3.411102	3.055619
22	6	0	-2.964320	0.028715	-0.378525
23	6	0	-2.220472	0.372179	1.923732
24	8	0	1.358029	-4.118850	-2.459101
25	6	0	-2.776472	1.435918	-0.639931
26	6	0	-0.508243	-1.344327	2.468221
27	8	0	0.621231	-1.270679	-3.934228
28	6	0	-0.464001	0.881004	3.564088
29	6	0	-3.703035	-3.839209	4.067592
30	6	0	0.947970	0.746282	3.814244
31	6	0	-3.409005	-2.407887	2.311746
32	6	0	-1.170079	-0.100074	2.829083
33	6	0	-2.430939	1.737556	1.769338
34	6	0	-2.669667	-0.491591	0.857580
35	6	0	1.600273	-0.378909	3.366942
36	6	0	-1.683271	2.723064	2.509370
37	6	0	1.750606	-2.111504	1.849399
38	6	0	-0.708758	2.297167	3.385134
39	6	0	-2.698634	2.280425	0.458466
40	6	0	-2.127898	1.841016	-1.887973
41	6	0	0.864801	-1.439363	2.742275
42	6	0	-5.859301	-0.941287	-1.470130
43	6	0	-4.715030	-0.397224	-2.056556
44	6	0	3.431636	0.972005	2.454877
45	6	0	-1.524170	3.103860	-1.924365
46	6	0	-2.092157	3.587907	0.392218
47	6	0	-7.100616	-0.494036	-1.904078
48	6	0	1.220079	-2.711019	0.738576
49	6	0	0.552736	3.017596	3.450546
50	6	0	-4.945727	-3.300169	4.381685
51	6	0	3.950302	1.276379	1.149203
52	6	0	0.787641	4.100136	2.609520
53	6	0	2.913356	-1.260408	1.655899
54	6	0	-7.157531	0.474660	-2.900997
55	6	0	-4.650283	-1.806676	2.536080
56	6	0	3.465612	-1.020724	0.391806
57	6	0	-5.959268	0.956161	-3.416738
58	6	0	-1.457671	3.869888	1.659169
59	6	0	3.750381	2.699755	0.967935
60	6	0	3.442717	2.293819	-1.396896
61	6	0	2.863388	-0.260665	2.675994
62	6	0	-0.280389	3.287717	-2.616340
63	6	0	1.589817	2.042198	3.685234
64	6	0	2.060765	4.217390	1.924191
65	6	0	3.868745	0.342167	0.078190
66	6	0	-5.428301	-2.261725	3.591277
67	6	0	0.303755	2.188402	-3.211300
68	6	0	3.613789	0.908749	-1.265391
69	6	0	3.034717	3.242715	2.110036
70	6	0	2.810192	2.153624	3.025536
71	6	0	1.722698	1.971512	-3.061719
72	6	0	-0.247294	4.549439	1.697871
73	6	0	0.381735	4.962580	0.458486
74	6	0	-1.510047	3.998408	-0.788211
75	6	0	2.540306	4.267640	-0.482152
76	6	0	-0.243726	4.706496	-0.759745
77	6	0	0.529315	4.247878	-1.889266
78	6	0	2.493858	2.845247	-2.330319
79	6	0	3.512417	3.209082	-0.285251
80	6	0	1.895825	4.029542	-1.750868
81	6	0	1.808052	4.747985	0.597342
82	1	0	-3.562371	-2.949348	-2.087860
83	1	0	-3.019843	-3.871135	0.080304
84	1	0	-3.292256	-4.656278	4.656358
85	1	0	-5.763371	-1.699840	-0.700192
86	1	0	-8.010934	-0.898627	-1.472742
87	1	0	-5.515586	-3.685347	5.219676
88	1	0	-8.104345	0.848838	-3.273402
89	1	0	-4.985659	-0.996106	1.896446
90	1	0	-5.955940	1.713561	-4.196903
91	1	0	-6.394850	-1.811687	3.795288
92	15	0	4.783415	-3.507505	-0.431478
93	6	0	6.432550	-2.736222	-0.653453
94	6	0	4.569090	-4.869098	-1.635309
95	6	0	4.759947	-4.207775	1.252033
96	1	0	6.349836	-1.703949	-0.300756
97	1	0	6.700074	-2.728102	-1.710329
98	1	0	7.199198	-3.257467	-0.076263
99	1	0	4.590399	-5.836716	-1.130416
100	1	0	3.592584	-4.738480	-2.124554
101	1	0	5.361815	-4.833349	-2.384463
102	1	0	5.390673	-5.098008	1.307860
103	1	0	3.725071	-4.467620	1.490818
104	1	0	5.113261	-3.454870	1.958463

The total electronic energy was calculated to be –4094.1490882 Hartree.

An imaginary frequency was found at 557.6421 cm^{-1}.

Supplementary Table 11. Optimized structure of **TS-E** (M06-2X/6-31G(d,p))

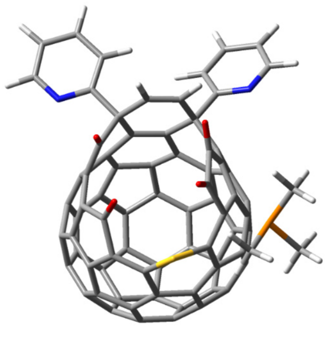

Standard orientation:

Center Number	Atomic Number	Atomic Type	Coordinates (Angstroms) X	Y	Z
1	6	0	3.131845	2.286291	-1.278882
2	6	0	2.652202	2.866994	-0.185310
3	6	0	2.147548	2.132098	1.027380
4	6	0	0.621418	2.245924	1.203155
5	6	0	-0.264199	2.870983	0.310128
6	6	0	-0.133645	3.609685	-0.985959
7	6	0	-1.516371	3.487780	-1.717036

8	6	0	-2.475339	2.824027	-0.750064
9	6	0	-3.260246	1.578002	-1.075108
10	6	0	-2.756228	-0.595971	-2.557363
11	6	0	-1.694521	-1.119721	-3.271121
12	6	0	-0.433074	-0.365057	-3.681341
13	6	0	0.700499	-1.338396	-3.284363
14	6	0	1.869935	-1.039325	-2.600900
15	6	0	2.488582	0.321733	-2.670083
16	6	0	3.305149	0.802738	-1.436462
17	16	0	-3.212881	1.088079	-2.769927
18	7	0	5.025410	-0.507799	-2.577969
19	8	0	0.702228	4.334508	-1.456375
20	8	0	2.388912	1.023061	-3.645008
21	7	0	2.306799	3.903541	2.687543
22	6	0	2.893361	0.037039	-0.202139
23	6	0	1.958927	-0.163364	2.045242
24	8	0	-1.732628	3.959710	-2.799167
25	6	0	2.848338	-1.400473	-0.341844
26	6	0	0.073816	1.452315	2.279683
27	8	0	-0.356591	0.769782	-4.062556
28	6	0	0.114480	-0.668325	3.572120
29	6	0	2.916138	4.532894	3.693322
30	6	0	-1.331982	-0.632828	3.720004
31	6	0	2.851728	2.772293	2.235114
32	6	0	0.809185	0.292185	2.817323
33	6	0	2.292867	-1.514506	2.042625
34	6	0	2.430347	0.639798	0.941451
35	6	0	-2.026476	0.402787	3.142794
36	6	0	1.556234	-2.486450	2.805593
37	6	0	-2.187439	1.973527	1.497295
38	6	0	0.479380	-2.068462	3.556908
39	6	0	2.726150	-2.147819	0.819416
40	6	0	2.367422	-1.969315	-1.603110
41	6	0	-1.314030	1.454942	2.492893
42	6	0	5.795512	1.156673	-1.032204
43	6	0	4.783597	0.475618	-1.712021
44	6	0	-3.673532	-1.143871	2.212513
45	6	0	1.851597	-3.270803	-1.569867
46	6	0	2.224019	-3.502042	0.825552
47	6	0	7.114054	0.798472	-1.279972
48	6	0	-1.669737	2.697938	0.466353
49	6	0	-0.732682	-2.873164	3.583656
50	6	0	4.087302	4.078232	4.290878
51	6	0	-3.993559	-1.582905	0.858279
52	6	0	-0.808814	-4.037707	2.829466
53	6	0	-3.331403	1.112872	1.325872
54	6	0	7.375913	-0.221810	-2.189290
55	6	0	4.034296	2.242078	2.754805
56	6	0	-3.607037	0.711922	-0.043398
57	6	0	6.296393	-0.842105	-2.808414
58	6	0	1.499741	-3.721995	2.054074
59	6	0	-3.684779	-2.994884	0.811723
60	6	0	-3.171748	-2.764964	-1.539341
61	6	0	-3.234194	0.158240	2.368319
62	6	0	0.694440	-3.609814	-2.350826
63	6	0	-1.855812	-1.970722	3.669812
64	6	0	-2.001510	-4.297529	2.043375
65	6	0	-3.856321	-0.744915	-0.266771
66	6	0	4.657464	2.907874	3.802951
67	6	0	0.098664	-2.615344	-3.100775
68	6	0	-3.412638	-1.379157	-1.518270
69	6	0	-3.049920	-3.387425	2.053794
70	6	0	-2.999211	-2.210065	2.894161
71	6	0	-1.339380	-2.482056	-3.091297
72	6	0	0.333255	-4.474727	2.049217
73	6	0	-0.147610	-5.041979	0.803785
74	6	0	1.787703	-4.058613	-0.358425
75	6	0	-2.246392	-4.589561	-0.384154
76	6	0	0.571431	-4.849370	-0.373964
77	6	0	-0.117480	-4.555804	-1.608030
78	6	0	-2.115809	-3.332605	-2.343380
79	6	0	-3.293745	-3.584057	-0.369713
80	6	0	-1.501637	-4.417962	-1.607320
81	6	0	-1.590281	-4.917675	0.795841
82	1	0	3.428660	2.887084	-2.133226
83	1	0	2.564835	3.946157	-0.136453
84	1	0	2.442458	5.449213	4.038850
85	1	0	5.540701	1.947890	-0.334361
86	1	0	7.926024	1.309874	-0.772442
87	1	0	4.536549	4.628291	5.109885
88	1	0	8.389765	-0.531588	-2.415848
89	1	0	4.446950	1.324609	2.348349
90	1	0	6.454712	-1.643897	-3.525348
91	1	0	5.573787	2.516259	4.233417
92	15	0	-3.869855	4.239626	-0.523340
93	6	0	-4.898974	4.387181	-2.041369
94	6	0	-3.155340	5.914605	-0.194939
95	6	0	-5.071388	3.956467	0.852808
96	1	0	-5.393030	3.422704	-2.183980
97	1	0	-4.214129	4.566361	-2.873403
98	1	0	-5.644935	5.182241	-1.976886
99	1	0	-2.498974	5.815350	0.674336
100	1	0	-2.561146	6.208359	-1.063434
101	1	0	-3.912847	6.675273	0.005724
102	1	0	-5.870661	4.703016	0.846340
103	1	0	-4.530436	3.988326	1.799605
104	1	0	-5.483863	2.954735	0.716527

The total electronic energy was calculated to be –4094.1463419 Hartree.

An imaginary frequency was found at 814.4291 cm^{-1}.

Supplementary Note 2

11. Formation Mechanism of β-Oxo-phosphorus Ylide (3a')

All values of the difference in the Gibbs energies indicated in Figures on this session were calibrated with reference to the Gibbs energy of **1'**.

11.1. Previously Proposed Intermediates (INTa and INTb)

The possible intermediates, i.e., carbene[3] and pentavalent phosphorus compounds[4,5] were computed on the basis of previously proposed mechanisms. However, none of them can rationally explain the experimental results since the calculated energy values at the stationary states are quite high (Supplementary Fig. 40).

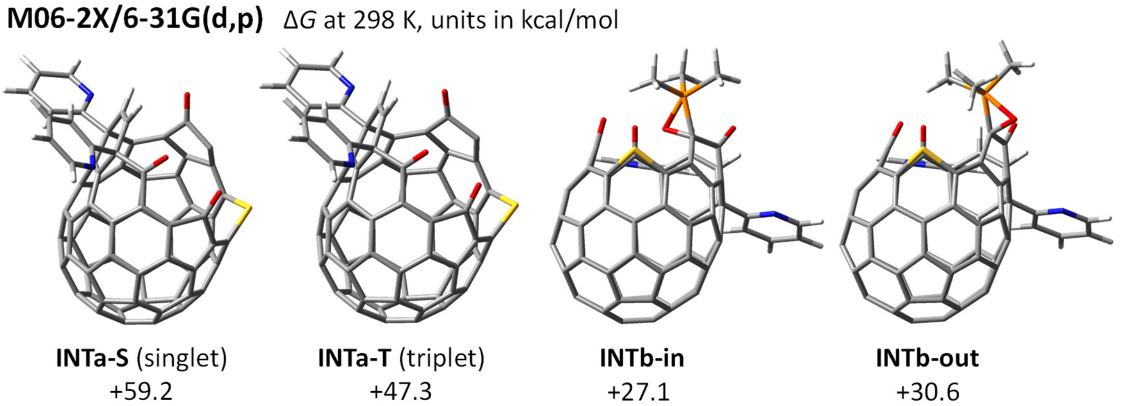

Supplementary Fig. 40. Optimized structures of betaines and transition states for the reaction of **1'** and PMe_3 (M06-2X/6-31G(d,p)).

Supplementary Table 12. Optimized structure of PMe_3 (M06-2X/6-31G(d,p))

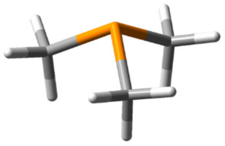

Standard orientation:

Center Number	Atomic Number	Atomic Type	Coordinates (Angstroms) X	Y	Z
1	15	0	0.000548	-0.001017	-0.614346
2	6	0	-1.280838	-1.019124	0.284030
3	6	0	-0.243883	1.618014	0.283188
4	6	0	1.523971	-0.597591	0.284491
5	1	0	-1.182879	-2.072296	-0.000944
6	1	0	-2.284009	-0.687012	-0.004783
7	1	0	-1.184365	-0.938824	1.374331
8	1	0	0.545482	2.321910	-0.002238
9	1	0	-1.204595	2.059031	-0.004086
10	1	0	-0.226384	1.493370	1.373435
11	1	0	2.385459	0.014238	-0.004447
12	1	0	1.739091	-1.633032	-0.001196
13	1	0	1.408482	-0.549929	1.374857

The total electronic energy was calculated to be –460.9904169 Hartree..

Supplementary Table 13. Optimized structure of **INTa-S** (M06-2X/6-31G(d,p))

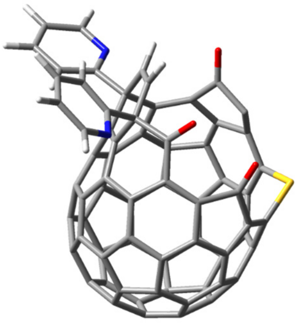

Standard orientation:

Center Number	Atomic Number	Atomic Type	Coordinates (Angstroms) X	Y	Z
1	6	0	3.812167	0.585928	1.477677
2	6	0	3.823672	-0.729255	1.288668
3	6	0	3.016844	-1.451670	0.242525
4	6	0	1.841573	-2.231178	0.858840
5	6	0	1.479949	-2.174260	2.195359
6	6	0	1.982418	-1.506269	3.461626
7	6	0	0.698486	-1.132079	4.296604
8	6	0	-0.445289	-1.743472	3.568661
9	6	0	-1.772571	-1.376334	3.463455
10	6	0	-2.872272	0.924233	2.973349
11	6	0	-2.271983	2.124965	2.642887
12	6	0	-0.803249	2.504931	2.886456
13	6	0	-0.364145	3.089024	1.519703
14	6	0	0.793651	2.835499	0.796213
15	6	0	2.055456	2.370208	1.462933
16	6	0	3.070265	1.557811	0.603443
17	16	0	-2.310685	0.031113	4.396979
18	7	0	3.635675	3.827158	-0.110518
19	8	0	3.087728	-1.351018	3.900206
20	8	0	2.301252	2.625826	2.613651
21	7	0	4.074564	-3.633547	0.066762
22	6	0	2.351564	0.875048	-0.534303
23	6	0	1.546934	-1.073799	-1.765573
24	6	0	1.486846	1.720467	-1.323752
25	6	0	0.918746	-2.827788	-0.110977
26	8	0	-0.143595	2.266699	3.855315
27	6	0	-0.235471	-2.664977	-2.304360
28	6	0	4.939380	-4.487983	-0.482758
29	6	0	-1.401858	-3.332655	-1.761407
30	6	0	3.964266	-2.421871	-0.476528
31	6	0	0.871397	-2.339776	-1.496989
32	6	0	1.048414	-0.224738	-2.748277
33	6	0	2.367548	-0.482938	-0.734470
34	6	0	-1.403175	-3.686684	-0.435358
35	6	0	-0.091688	-0.569412	-3.555026
36	6	0	-0.666008	-3.242122	1.695515
37	6	0	-0.726812	-1.771742	-3.334425
38	6	0	1.009373	1.200608	-2.517251
39	6	0	0.724625	2.777277	-0.654844
40	6	0	-0.224420	-3.486085	0.358082
41	6	0	5.314517	2.148298	-0.450211
42	6	0	4.060690	2.568889	-0.003716
43	6	0	-3.648065	-2.752854	-0.134885
44	6	0	-0.417960	3.258913	-1.304629
45	6	0	-0.163339	1.721930	-3.176238
46	6	0	6.160707	3.089647	-1.021985
47	6	0	0.137012	-2.553252	2.550774
48	6	0	-2.179651	-1.822377	-3.361817
49	6	0	5.729696	-4.179108	-1.585101
50	6	0	-4.247023	-1.642249	0.584671
51	6	0	-2.917921	-0.665160	-3.578267
52	6	0	-2.078055	-2.998791	1.688987
53	6	0	5.728679	4.407949	-1.128872
54	6	0	4.717951	-2.009224	-1.576962
55	6	0	-2.615858	-1.924558	2.416602
56	6	0	4.458411	4.722982	-0.659539
57	6	0	-0.854274	0.632059	-3.822676
58	6	0	-4.793367	-0.765577	-0.429642
59	6	0	-4.339714	1.110004	1.036740
60	6	0	-2.553153	-3.385262	0.394581
61	6	0	-1.590042	3.597171	-0.548342
62	6	0	-2.596024	-2.771702	-2.356451
63	6	0	-4.078703	-0.382898	-2.754435
64	6	0	-3.711895	-1.157350	1.796582
65	6	0	5.614252	-2.908709	-2.140018
66	6	0	-1.561039	3.414880	0.819026
67	6	0	-3.793428	0.293358	2.039173
68	6	0	-4.439829	-1.267519	-1.746537
69	6	0	-3.703446	-2.492589	-1.557538
70	6	0	-2.698930	2.822612	1.483383
71	6	0	-2.241139	0.593835	-3.830364
72	6	0	-2.991938	1.654881	-3.187685
73	6	0	-0.870112	2.743100	-2.577796
74	6	0	-4.523506	1.525291	-1.273723
75	6	0	-2.320191	2.716724	-2.586150
76	6	0	-2.767587	3.236264	-1.315159
77	6	0	-3.794830	2.414943	0.758203
78	6	0	-4.839143	0.590372	-0.208591
79	6	0	-3.850411	2.649364	-0.670652
80	6	0	-4.122475	1.048284	-2.515042
81	1	0	4.370213	1.023120	2.300338
82	1	0	4.389431	-1.368430	1.957844
83	1	0	5.001821	-5.467753	-0.015354
84	1	0	5.610057	1.110269	-0.337520
85	1	0	7.145047	2.799272	-1.375687
86	1	0	6.414514	-4.914411	-1.991840
87	1	0	6.358568	5.176061	-1.562674
88	1	0	4.593175	-1.008749	-1.979495
89	1	0	4.079996	5.740101	-0.722968
90	1	0	6.212393	-2.623498	-2.999776

The total electronic energy was calculated to be –3557.8481384 Hartree..

Supplementary Table 14. Optimized structure of **INTa-T** (M06-2X/6-31G(d,p))

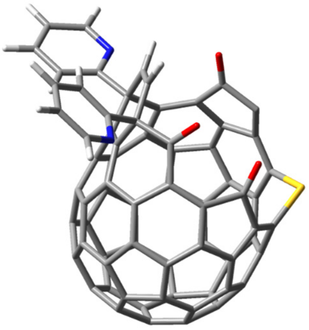

Standard orientation:

Center Number	Atomic Number	Atomic Type	Coordinates (Angstroms) X	Y	Z
1	6	0	3.812167	0.585928	1.477677
2	6	0	3.823672	-0.729255	1.288668
3	6	0	3.016844	-1.451670	0.242525
4	6	0	1.841573	-2.231178	0.858840
5	6	0	1.479949	-2.174260	2.195359
6	6	0	1.982418	-1.506269	3.461626
7	6	0	0.698486	-1.132079	4.296604
8	6	0	-0.445289	-1.743472	3.568661
9	6	0	-1.772571	-1.376334	3.463455
10	6	0	-2.872272	0.924233	2.973349
11	6	0	-2.271983	2.124965	2.642887
12	6	0	-0.803249	2.504931	2.886456
13	6	0	-0.364145	3.089024	1.519703
14	6	0	0.793651	2.835499	0.796213
15	6	0	2.055456	2.370208	1.462933
16	6	0	3.070265	1.557811	0.603443
17	16	0	-2.310685	0.031113	4.396979
18	7	0	3.635675	3.827158	-0.110518
19	8	0	3.087728	-1.351018	3.900206
20	8	0	2.301252	2.625826	2.613651
21	7	0	4.074564	-3.633547	0.066762
22	6	0	2.351564	0.875048	-0.534303
23	6	0	1.546934	-1.073799	-1.765573
24	6	0	1.486846	1.720467	-1.323752
25	6	0	0.918746	-2.827788	-0.110977
26	8	0	-0.143595	2.266699	3.855315
27	6	0	-0.235471	-2.664977	-2.304360
28	6	0	4.939380	-4.487983	-0.482758
29	6	0	-1.401858	-3.332655	-1.761407
30	6	0	3.964266	-2.421871	-0.476528
31	6	0	0.871397	-2.339776	-1.496989
32	6	0	1.048414	-0.224738	-2.748277
33	6	0	2.367548	-0.482938	-0.734470
34	6	0	-1.403175	-3.686684	-0.435358
35	6	0	-0.091688	-0.569412	-3.555026
36	6	0	-0.666008	-3.242122	1.695515
37	6	0	-0.726812	-1.771742	-3.334425
38	6	0	1.009373	1.200608	-2.517251
39	6	0	0.724625	2.777277	-0.654844
40	6	0	-0.224420	-3.486085	0.358082
41	6	0	5.314517	2.148298	-0.450211
42	6	0	4.060690	2.568889	-0.003716
43	6	0	-3.648065	-2.752854	-0.134885
44	6	0	-0.417960	3.258913	-1.304629
45	6	0	-0.163339	1.721930	-3.176238
46	6	0	6.160707	3.089647	-1.021985
47	6	0	0.137012	-2.553252	2.550774
48	6	0	-2.179651	-1.822377	-3.361817
49	6	0	5.729696	-4.179108	-1.585101
50	6	0	-4.247023	-1.642249	0.584671
51	6	0	-2.917921	-0.665160	-3.578267
52	6	0	-2.078055	-2.998791	1.688987
53	6	0	5.728679	4.407949	-1.128872
54	6	0	4.717951	-2.009224	-1.576962
55	6	0	-2.615858	-1.924558	2.416602
56	6	0	4.458411	4.722982	-0.659539
57	6	0	-0.854274	0.632059	-3.822676
58	6	0	-4.793367	-0.765577	-0.429642
59	6	0	-4.339714	1.110004	1.036740
60	6	0	-2.553153	-3.385262	0.394581
61	6	0	-1.590042	3.597171	-0.548342
62	6	0	-2.596024	-2.771702	-2.356451
63	6	0	-4.078703	-0.382898	-2.754435
64	6	0	-3.711895	-1.157350	1.796582
65	6	0	5.614252	-2.908709	-2.140018
66	6	0	-1.561039	3.414880	0.819026
67	6	0	-3.793428	0.293358	2.039173
68	6	0	-4.439829	-1.267519	-1.746537
69	6	0	-3.703446	-2.492589	-1.557538
70	6	0	-2.698930	2.822612	1.483383
71	6	0	-2.241139	0.593835	-3.830364
72	6	0	-2.991938	1.654881	-3.187685
73	6	0	-0.870112	2.743100	-2.577796
74	6	0	-4.523506	1.525291	-1.273723
75	6	0	-2.320191	2.716724	-2.586150
76	6	0	-2.767587	3.236264	-1.315159
77	6	0	-3.794830	2.414943	0.758203
78	6	0	-4.839143	0.590372	-0.208591
79	6	0	-3.850411	2.649364	-0.670652
80	6	0	-4.122475	1.048284	-2.515042
81	1	0	4.370213	1.023120	2.300338
82	1	0	4.389431	-1.368430	1.957844
83	1	0	5.001821	-5.467753	-0.015354
84	1	0	5.610057	1.110269	-0.337520
85	1	0	7.145047	2.799272	-1.375687
86	1	0	6.414514	-4.914411	-1.991840
87	1	0	6.358568	5.176061	-1.562674
88	1	0	4.593175	-1.008749	-1.979495
89	1	0	4.079996	5.740101	-0.722968
90	1	0	6.212393	-2.623498	-2.999776

The total electronic energy was calculated to be –3557.8673018 Hartree..

Supplementary Table 15. Optimized structure of **INTb-in** (M06-2X/6-31G(d,p))

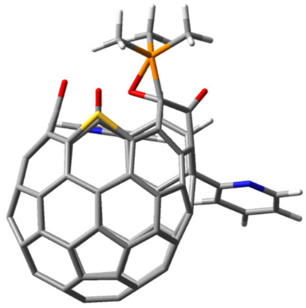

Standard orientation:

Center Number	Atomic Number	Atomic Type	Coordinates (Angstroms) X	Y	Z
1	6	0	-3.719406	-0.561731	0.732484
2	6	0	-3.733094	-0.731485	-0.586216
3	6	0	-2.631237	-1.375424	-1.387403
4	6	0	-1.836031	-0.345944	-2.212964
5	6	0	-2.018368	1.025945	-2.145651
6	6	0	-3.010280	2.009616	-1.576399
7	6	0	-2.193681	3.204012	-1.013804
8	6	0	-0.816155	2.979856	-1.563108
9	6	0	0.466598	3.391549	-1.238039
10	6	0	1.647999	2.796558	-1.862575
11	6	0	2.936393	2.638235	-1.158774
12	6	0	2.999417	2.841911	0.300482
13	6	0	1.828291	3.327751	1.020110
14	6	0	1.497402	2.781083	2.248434
15	6	0	0.100251	2.478390	2.814099
16	6	0	0.228958	0.984599	3.211273
17	6	0	-0.575743	-0.104494	2.902910
18	6	0	-2.011288	0.056147	2.484790
19	6	0	-2.652300	-1.097757	1.645014
20	6	0	-1.588635	-1.857143	0.886528
21	6	0	-1.596679	-2.025462	-0.477956
22	6	0	-0.473385	-2.651372	-1.137412
23	6	0	-0.011967	-2.117713	-2.415502
24	6	0	-0.621344	-0.852842	-2.851001
25	6	0	0.226232	0.051582	-3.508659
26	6	0	0.124647	1.442482	-3.201815
27	6	0	-0.927823	1.881184	-2.470051
28	6	0	1.428271	1.980504	-2.980794
29	6	0	2.357195	0.995911	-3.444221
30	6	0	1.614122	-0.207902	-3.766214
31	6	0	2.143645	-1.439932	-3.475808
32	6	0	1.308086	-2.417572	-2.802918
33	6	0	2.196959	-3.198366	-1.966761
34	6	0	1.747534	-3.683546	-0.757860
35	6	0	0.402446	-3.385003	-0.344529
36	6	0	0.414321	-3.192555	1.086697
37	6	0	-0.452931	-2.281759	1.671185
38	6	0	0.039132	-1.415873	2.746134
39	6	0	1.359305	-1.611266	3.170281
40	6	0	2.173277	-0.486039	3.528090
41	6	0	1.618415	0.772306	3.444088
42	6	0	2.381326	1.846511	2.850583
43	6	0	3.639764	1.611116	2.350884
44	6	0	3.950476	2.121603	1.040676
45	6	0	4.871088	1.193655	0.438941
46	6	0	4.843295	1.022131	-0.923593
47	6	0	3.894115	1.766375	-1.726397
48	6	0	3.594532	0.916145	-2.863687
49	6	0	4.212970	-0.379065	-2.677406
50	6	0	3.496589	-1.538600	-2.969920
51	6	0	3.543383	-2.657132	-2.058171
52	6	0	4.358678	-2.610000	-0.933366
53	6	0	3.887249	-3.147769	0.329996
54	6	0	2.606157	-3.674520	0.407411
55	6	0	1.769043	-3.377665	1.544777
56	6	0	2.238234	-2.594670	2.576217
57	6	0	3.580433	-2.048606	2.511309
58	6	0	3.533703	-0.725830	3.087679
59	6	0	4.254210	0.308525	2.502612
60	6	0	5.048373	0.057053	1.325801
61	6	0	5.136312	-1.224625	0.796996
62	6	0	5.119659	-1.409330	-0.642367
63	6	0	5.015515	-0.308135	-1.483047
64	6	0	4.380853	-2.304119	1.400841
65	6	0	-3.290275	-2.076428	2.649684
66	6	0	-4.290417	-2.958815	2.234968
67	6	0	-4.807184	-3.855647	3.160579
68	6	0	-4.311510	-3.843315	4.460494
69	6	0	-3.311936	-2.928815	4.772273
70	6	0	-3.285179	-2.408387	-2.315019
71	6	0	-3.528060	-3.712482	-1.876087
72	6	0	-4.188996	-4.586986	-2.728401
73	6	0	-4.587593	-4.131338	-3.981006
74	6	0	-4.307002	-2.811521	-4.317934
75	16	0	0.735586	4.438570	0.175298
76	7	0	-2.806227	-2.063248	3.891213
77	7	0	-3.672076	-1.961226	-3.508904
78	8	0	-0.833656	3.215655	2.894355
79	8	0	-4.205839	1.935187	-1.536122
80	8	0	-2.668811	1.009030	2.813243
81	8	0	-2.415914	3.286684	0.454365
82	15	0	-2.969158	4.729479	-0.306210
83	6	0	-2.101261	5.243101	1.203856
84	6	0	-3.577480	6.301265	-1.062865
85	6	0	-4.603967	4.130216	0.364715
86	1	0	-4.532824	-0.033331	1.220588
87	1	0	-4.554091	-0.339427	-1.173125
88	1	0	-4.651139	-2.926417	1.212126
89	1	0	-5.587484	-4.552899	2.871810
90	1	0	-4.687968	-4.523371	5.216093
91	1	0	-2.895047	-2.884421	5.775501
92	1	0	-3.195140	-4.026739	-0.891619
93	1	0	-4.387532	-5.608919	-2.420978
94	1	0	-5.103265	-4.778233	-4.681555
95	1	0	-4.604530	-2.413918	-5.285536
96	1	0	-2.825807	5.409206	2.002409
97	1	0	-1.375138	4.487765	1.491082
98	1	0	-1.564356	6.167667	0.982102
99	1	0	-2.703004	6.927288	-1.238163
100	1	0	-4.308159	6.806858	-0.421978
101	1	0	-4.033067	6.096341	-2.029891
102	1	0	-4.437976	3.336237	1.100302

103	1	0	-5.145401	4.956150	0.832928
104	1	0	-5.220092	3.725724	-0.445746

The total electronic energy was calculated to be –4094.1466508 Hartree..

Supplementary Table 16. Optimized structure of **INTb-out** (M06-2X/6-31G(d,p))

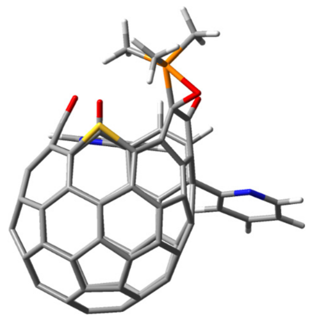

Standard orientation:

Center Number	Atomic Number	Atomic Type	Coordinates (Angstroms) X	Y	Z
1	6	0	3.817568	-0.154363	-0.640513
2	6	0	3.762364	-0.185540	0.686326
3	6	0	2.712507	-0.910208	1.484590
4	6	0	1.762278	0.069264	2.197827
5	6	0	1.785605	1.445487	2.043731
6	6	0	2.567641	2.501644	1.227520
7	6	0	1.594903	3.455128	0.864954
8	6	0	0.326147	3.218230	1.449993
9	6	0	-1.000037	3.502262	1.136039
10	6	0	-2.093153	2.765596	1.740022
11	6	0	-3.307088	2.394697	0.991331
12	6	0	-3.304749	2.508252	-0.481149
13	6	0	-2.162007	3.098242	-1.169072
14	6	0	-1.678603	2.515488	-2.329598
15	6	0	-0.213804	2.304258	-2.706475
16	6	0	-0.175147	0.831339	-3.185025
17	6	0	0.733578	-0.171956	-2.869253
18	6	0	2.150542	0.114971	-2.485417
19	6	0	2.871871	-0.892165	-1.545949
20	6	0	1.857637	-1.700697	-0.775489
21	6	0	1.817848	-1.761547	0.595014
22	6	0	0.746372	-2.472274	1.255332
23	6	0	0.181369	-1.923586	2.483693
24	6	0	0.611485	-0.573065	2.859385
25	6	0	-0.358747	0.232965	3.465377
26	6	0	-0.420405	1.616177	3.110647
27	6	0	0.575848	2.143132	2.375460
28	6	0	-1.790185	1.995447	2.878877
29	6	0	-2.590034	0.907694	3.328625
30	6	0	-1.708386	-0.186629	3.708561
31	6	0	-2.073343	-1.484251	3.462153
32	6	0	-1.105055	-2.370952	2.855584
33	6	0	-1.865184	-3.294769	2.037451
34	6	0	-1.321234	-3.785746	0.871369
35	6	0	-0.004080	-3.354446	0.483563
36	6	0	0.015744	-3.250869	-0.956482
37	6	0	0.808300	-2.293511	-1.569144
38	6	0	0.258582	-1.534263	-2.693326
39	6	0	-1.025552	-1.872064	-3.136019
40	6	0	-1.938228	-0.844339	-3.548174
41	6	0	-1.523546	0.470581	-3.469884
42	6	0	-2.420126	1.471338	-2.944498
43	6	0	-3.671446	1.117995	-2.498765
44	6	0	-4.112537	1.640582	-1.230632
45	6	0	-4.946227	0.633625	-0.630788
46	6	0	-4.960733	0.517952	0.738404
47	6	0	-4.158241	1.410516	1.552822
48	6	0	-3.800835	0.651858	2.731058
49	6	0	-4.235116	-0.723779	2.568140
50	6	0	-3.394098	-1.773599	2.928696
51	6	0	-3.270916	-2.920741	2.065772
52	6	0	-4.047486	-3.025754	0.915193
53	6	0	-3.468709	-3.555605	-0.305267
54	6	0	-2.134080	-3.935862	-0.317176
55	6	0	-1.296929	-3.600255	-1.443369
56	6	0	-1.813513	-2.920842	-2.526678
57	6	0	-3.207463	-2.518799	-2.525043
58	6	0	-3.281495	-1.218031	-3.149009
59	6	0	-4.131697	-0.250104	-2.627215
60	6	0	-4.945951	-0.547901	-1.474873
61	6	0	-4.909557	-1.810969	-0.897638
62	6	0	-4.931264	-1.939170	0.548156
63	6	0	-4.993118	-0.801069	1.345873
64	6	0	-4.014076	-2.818552	-1.430205
65	6	0	3.658430	-1.866634	-2.441664
66	6	0	4.693093	-2.632065	-1.900046
67	6	0	5.348955	-3.534543	-2.726456
68	6	0	4.952425	-3.641711	-4.056388
69	6	0	3.907680	-2.837298	-4.496479
70	6	0	3.454799	-1.772685	2.516915
71	6	0	3.800697	-3.097889	2.242320
72	6	0	4.526967	-3.806088	3.191598
73	6	0	4.886794	-3.169902	4.374675
74	6	0	4.504982	-1.843036	4.546104
75	16	0	-1.286567	4.406087	-0.367845
76	7	0	3.267819	-1.967273	-3.711302
77	7	0	3.807844	-1.152465	3.643081
78	8	0	0.703159	3.068711	-2.548262
79	8	0	3.779997	2.564231	0.927341
80	8	0	2.745110	1.071950	-2.921220
81	8	0	1.804059	4.769260	1.314093
82	15	0	2.461567	4.392413	-0.185372
83	6	0	1.049010	5.039990	-1.028019
84	6	0	3.778978	4.969986	0.899534
85	6	0	3.213410	4.589862	-1.770792
86	1	0	4.589803	0.423178	-1.140444
87	1	0	4.468053	0.393797	1.271075
88	1	0	4.967578	-2.507910	-0.857321
89	1	0	6.158550	-4.145214	-2.339106
90	1	0	5.437717	-4.331704	-4.737205
91	1	0	3.564041	-2.889361	-5.526717

92	1	0	3.494401	-3.560379	1.309556
93	1	0	4.803396	-4.840300	3.011336
94	1	0	5.448104	-3.684342	5.146398
95	1	0	4.771281	-1.305639	5.453413
96	1	0	1.339248	5.456997	-1.901976
97	1	0	0.401492	4.574652	-1.549314
98	1	0	0.463435	5.732725	-0.430228
99	1	0	3.385309	5.751629	1.552990
100	1	0	4.595541	5.374095	0.294791
101	1	0	4.115769	4.115548	1.489300
102	1	0	2.573876	4.520262	-2.423433
103	1	0	3.737246	5.318091	-2.280428
104	1	0	3.977143	3.938598	-2.034925

The total electronic energy was calculated to be –4094.1401351 Hartree..

11.2. Formation of 1,3-Betaines (INT1)

The nucleophilic addition of PMe_3 to a carbonyl oxygen atom on **1'** was examined. Even though the formation of **INT1-A** seems to be preferable, the *t*-butyl group on the pyridyl group prohibits its formation in the real system. Thus, in the reaction system, the formation of **INT1-B** and **INT1-C1** is considered to be competed and only **INT1-C1** undergoes further reaction to give **3a'** (Supplementary Fig. 41). Note that **INT1-C1** should be reversibly converted into **INT1-C2**.

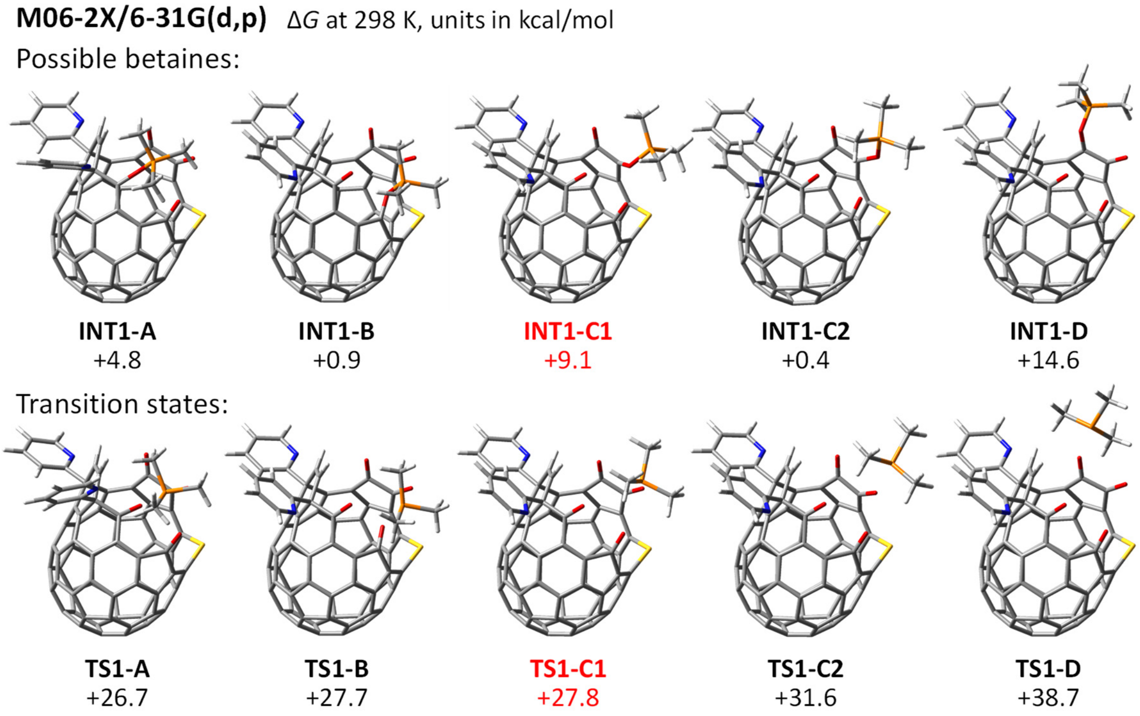

Supplementary Fig. 41. Optimized structures of 1,3-betaines and transition states for the reaction of **1'** and PMe_3 (M06-2X/6-31G(d,p)).

Supplementary Table 17. Optimized structure of **INT1-A** (M06-2X/6-31G(d,p))

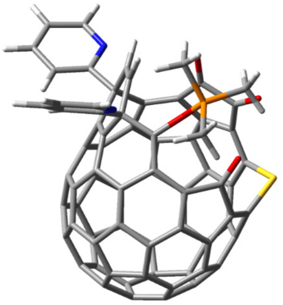

Standard orientation:

Center Number	Atomic Number	Atomic Type	X	Y	Z
1	6	0	-3.708492	0.632737	0.743864
2	6	0	-3.240897	1.732975	1.322960
3	6	0	-2.048912	2.515477	0.841885
4	6	0	-0.853753	2.381478	1.803471
5	6	0	-0.824011	1.557212	2.916117
6	6	0	-1.744024	0.527560	3.613715
7	6	0	-0.868068	-0.488209	4.046979
8	6	0	0.499520	-0.182580	3.836196
9	6	0	1.676468	-0.899833	3.640378
10	6	0	2.847368	-0.282085	3.048976
11	6	0	3.733513	-0.998189	2.114265
12	6	0	3.276111	-2.273381	1.526244
13	6	0	2.021135	-2.873998	1.963828
14	6	0	1.155899	-3.422435	1.030723
15	6	0	-0.364365	-3.280997	0.997775
16	6	0	-0.658939	-2.915992	-0.478705
17	6	0	-1.492675	-1.928594	-0.990588
18	6	0	-2.691920	-1.423520	-0.252919
19	6	0	-3.152281	0.037478	-0.518991
20	6	0	-2.008873	0.848181	-1.077008
21	6	0	-1.544286	2.003308	-0.499596
22	6	0	-0.374244	2.659511	-1.037752
23	6	0	0.587083	3.262198	-0.120267
24	6	0	0.396728	2.985103	1.306980
25	6	0	1.564420	2.846206	2.065575
26	6	0	1.611948	1.832406	3.071985
27	6	0	0.476134	1.190332	3.401180
28	6	0	2.862964	1.124242	2.986423
29	6	0	3.682186	1.862741	2.087127
30	6	0	2.887636	2.943274	1.521147
31	6	0	3.060873	3.315657	0.213840
32	6	0	1.888960	3.494788	-0.614579
33	6	0	2.283475	3.110408	-1.955170
34	6	0	1.365881	2.524814	-2.798846
35	6	0	0.028742	2.294505	-2.318926
36	6	0	-0.435725	1.055352	-2.896526
37	6	0	-1.308827	0.246943	-2.186456
38	6	0	-1.087083	-1.199858	-2.180920
39	6	0	-0.035778	-1.696331	-2.960252
40	6	0	0.773140	-2.776840	-2.473200
41	6	0	0.501189	-3.285367	-1.218390
42	6	0	1.588001	-3.572939	-0.314210
43	6	0	2.886753	-3.318986	-0.685806
44	6	0	3.741429	-2.640873	0.255211
45	6	0	4.645810	-1.827451	-0.512417
46	6	0	5.082061	-0.639469	0.022992
47	6	0	4.644007	-0.232226	1.344431
48	6	0	4.621319	1.213640	1.322094
49	6	0	4.880756	1.678541	-0.028491
50	6	0	4.121800	2.710758	-0.574339
51	6	0	3.649998	2.611547	-1.932023
52	6	0	4.015120	1.530489	-2.729469
53	6	0	3.044617	0.927972	-3.624089
54	6	0	1.750096	1.426821	-3.660638
55	6	0	0.627834	0.521513	-3.712674
56	6	0	0.825266	-0.842931	-3.749190
57	6	0	2.174281	-1.375381	-3.711864
58	6	0	2.142767	-2.572692	-2.904235
59	6	0	3.182166	-2.835547	-2.019615
60	6	0	4.293745	-1.922510	-1.917941
61	6	0	4.348918	-0.791960	-2.723347
62	6	0	4.815182	0.464070	-2.164584
63	6	0	5.207762	0.528425	-0.831470
64	6	0	3.260388	-0.507102	-3.636963
65	6	0	-4.249618	-0.020579	-1.597440
66	6	0	-5.114113	1.061904	-1.772486
67	6	0	-6.060701	0.993614	-2.785838
68	6	0	-6.111846	-0.145079	-3.584714
69	6	0	-5.203013	-1.165866	-3.331202
70	6	0	-2.489339	3.984389	0.747651
71	6	0	-2.999716	4.515122	-0.439384
72	6	0	-3.439874	5.832842	-0.443640
73	6	0	-3.361654	6.571457	0.732034
74	6	0	-2.849640	5.946086	1.864508
75	16	0	1.540166	-2.672394	3.651086
76	7	0	-4.286912	-1.111343	-2.361329
77	7	0	-2.423778	4.682485	1.881638
78	8	0	-1.126629	-3.324092	1.929084
79	8	0	-2.936530	0.532976	3.788783
80	8	0	-3.322013	-2.132144	0.495465
81	8	0	-1.235558	-1.503949	4.574945
82	15	0	-3.484774	-3.954835	0.481146
83	6	0	-2.601572	-5.120525	1.532126
84	6	0	-5.170459	-3.777304	1.091991
85	6	0	-3.460406	-4.595102	-1.185376
86	1	0	-4.554567	0.106600	1.176493
87	1	0	-3.682459	2.087301	2.247640
88	1	0	-5.036629	1.925625	-1.119789
89	1	0	-6.749474	1.816473	-2.949848
90	1	0	-6.835697	-0.241608	-4.385859
91	1	0	-5.205866	-2.072354	-3.931585
92	1	0	-3.037148	3.907504	-1.337805
93	1	0	-3.833603	6.276714	-1.352736
94	1	0	-3.687460	7.604562	0.774666
95	1	0	-2.776908	6.484996	2.806424
96	1	0	-3.072678	-6.104950	1.491100
97	1	0	-1.575421	-5.181830	1.160941
98	1	0	-2.591334	-4.755920	2.560815
99	1	0	-5.140579	-3.386810	2.111582
100	1	0	-5.662400	-4.753914	1.087381
101	1	0	-5.685953	-3.077365	0.431940
102	1	0	-2.423644	-4.672730	-1.519292

103	1	0	-3.932874	-5.582567	-1.174571
104	1	0	-4.003742	-3.907263	-1.833262

The total electronic energy was calculated to be –4094.1811046 Hartree..

Supplementary Table 18. Optimized structure of **INT1-B** (M06-2X/6-31G(d,p))

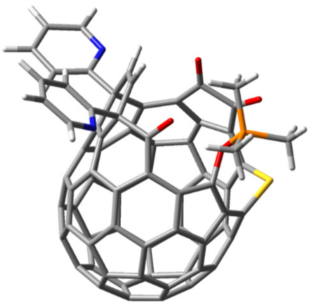

Standard orientation:

Center Number	Atomic Number	Atomic Type	Coordinates (Angstroms) X	Y	Z
1	6	0	3.808279	0.155360	-0.345694
2	6	0	3.494024	1.076301	-1.249924
3	6	0	2.338526	2.033360	-1.134023
4	6	0	1.226452	1.709186	-2.148523
5	6	0	1.222396	0.610196	-2.991426
6	6	0	2.105303	-0.627591	-3.277094
7	6	0	1.184272	-1.668501	-3.511641
8	6	0	-0.163917	-1.233205	-3.543845
9	6	0	-1.412871	-1.793858	-3.291285
10	6	0	-2.571955	-0.965115	-3.022798
11	6	0	-3.598032	-1.335901	-2.032128
12	6	0	-3.310284	-2.421366	-1.072927
13	6	0	-2.079945	-3.195835	-1.189758
14	6	0	-1.353491	-3.513379	-0.053302
15	6	0	0.124099	-3.520265	0.177375
16	6	0	0.357818	-2.717050	1.451947
17	6	0	1.227443	-1.678771	1.764717
18	6	0	2.527784	-1.474242	1.054277
19	6	0	3.091102	-0.028064	0.962174
20	6	0	1.979467	0.974557	1.149317
21	6	0	1.672941	1.948380	0.232107
22	6	0	0.522872	2.799035	0.439330
23	6	0	-0.295459	3.178796	-0.707524
24	6	0	-0.004398	2.502728	-1.975699
25	6	0	-1.107713	2.228920	-2.791780
26	6	0	-1.155764	0.979099	-3.483553
27	6	0	-0.056975	0.202481	-3.498617
28	6	0	-2.467580	0.400861	-3.347029
29	6	0	-3.294401	1.409887	-2.778283
30	6	0	-2.460155	2.554921	-2.443429
31	6	0	-2.715071	3.287724	-1.313939
32	6	0	-1.610190	3.619343	-0.441578
33	6	0	-2.155066	3.649835	0.901089
34	6	0	-1.372031	3.268493	1.967918
35	6	0	-0.023396	2.832001	1.718993
36	6	0	0.275501	1.777724	2.659213
37	6	0	1.132711	0.751310	2.296309
38	6	0	0.784205	-0.623016	2.659910
39	6	0	-0.372312	-0.816694	3.424137
40	6	0	-1.227829	-1.938766	3.163152
41	6	0	-0.891796	-2.793377	2.131733
42	6	0	-1.915186	-3.255277	1.225796
43	6	0	-3.213997	-2.828752	1.368962
44	6	0	-3.917708	-2.389809	0.190852
45	6	0	-4.810618	-1.340594	0.603974
46	6	0	-5.089840	-0.325280	-0.278892
47	6	0	-4.501436	-0.331170	-1.604669
48	6	0	-4.351978	1.059113	-1.973878
49	6	0	-4.688005	1.897929	-0.837658
50	6	0	-3.891588	2.993213	-0.512838
51	6	0	-3.553135	3.248820	0.864065
52	6	0	-4.082675	2.458147	1.880109
53	6	0	-3.253193	2.071597	3.006068
54	6	0	-1.927745	2.480745	3.047978
55	6	0	-0.899778	1.559281	3.467445
56	6	0	-1.220561	0.274138	3.851537
57	6	0	-2.602997	-0.164095	3.812017
58	6	0	-2.606679	-1.539021	3.368875
59	6	0	-3.582568	-1.974666	2.480141
60	6	0	-4.594834	-1.060323	2.012295
61	6	0	-4.620378	0.251457	2.468855
62	6	0	-4.921227	1.327280	1.541858
63	6	0	-5.186383	1.040303	0.206647
64	6	0	-3.596364	0.713289	3.384739
65	6	0	4.078613	0.150722	2.129834
66	6	0	5.017189	1.184082	2.098122
67	6	0	5.860027	1.344075	3.189727
68	6	0	5.738173	0.473152	4.268654
69	6	0	4.768093	-0.520315	4.203696
70	6	0	2.898057	3.440309	-1.394752
71	6	0	3.346130	4.249473	-0.347996
72	6	0	3.899914	5.486416	-0.653673
73	6	0	3.992742	5.870320	-1.987045
74	6	0	3.529753	4.985825	-2.956092
75	16	0	-1.434772	-3.503641	-2.804633
76	7	0	3.950404	-0.683209	3.160759
77	7	0	2.996122	3.796266	-2.675861
78	8	0	0.981249	-4.021497	-0.408064
79	8	0	3.304366	-0.744169	-3.314588
80	8	0	3.156225	-2.400909	0.600853
81	8	0	1.505211	-2.811798	-3.699776
82	15	0	2.804488	-3.865020	-0.422792
83	6	0	3.557879	-3.547296	-2.027438
84	6	0	4.158751	-4.137817	0.731137
85	6	0	2.527911	-5.643598	-0.435670
86	1	0	4.639174	-0.521384	-0.523073
87	1	0	4.045903	1.130389	-2.181622
88	1	0	5.075391	1.833995	1.230795
89	1	0	6.602108	2.136312	3.198689
90	1	0	6.376403	0.560376	5.140529
91	1	0	4.636692	-1.221271	5.024417

92	1	0	3.249179	3.916087	0.680214
93	1	0	4.249241	6.141788	0.138135
94	1	0	4.411873	6.828424	-2.272769
95	1	0	3.589427	5.243374	-4.011093
96	1	0	4.367516	-4.255437	-2.216487
97	1	0	2.776821	-3.662598	-2.783200
98	1	0	3.942095	-2.526218	-2.059390
99	1	0	4.511528	-3.104582	0.700786
100	1	0	4.997279	-4.817167	0.554647
101	1	0	3.680884	-4.348216	1.689620
102	1	0	1.771514	-5.737177	-1.217659
103	1	0	3.370137	-6.313276	-0.637207
104	1	0	2.080249	-5.857330	0.534902

The total electronic energy was calculated to be –4094.1897095 Hartree..

Supplementary Table 19. Optimized structure of **INT1-C1** (M06-2X/6-31G(d,p))

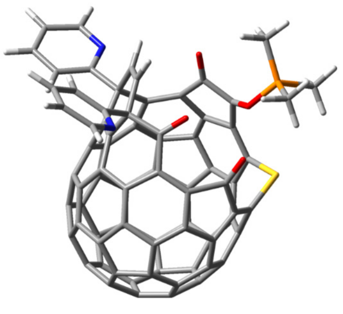

Standard orientation:

Center Number	Atomic Number	Atomic Type	Coordinates (Angstroms) X	Y	Z
1	6	0	3.328742	-1.938014	-1.105421
2	6	0	3.579547	-1.677747	0.172851
3	6	0	2.546180	-1.677021	1.267111
4	6	0	2.153598	-0.254619	1.715119
5	6	0	2.562159	0.908973	1.088959
6	6	0	3.388346	1.263072	-0.160192
7	6	0	2.821760	2.476052	-0.644168
8	6	0	1.607090	2.824748	0.037721
9	6	0	0.423951	3.541224	-0.153830
10	6	0	-0.660896	3.479884	0.805251
11	6	0	-2.076757	3.436983	0.390512
12	6	0	-2.405311	3.180429	-1.027287
13	6	0	-1.347243	3.122267	-2.030015
14	6	0	-1.371148	2.138462	-2.998397
15	6	0	-0.194493	1.342122	-3.562582
16	6	0	-0.673876	-0.127131	-3.402820
17	6	0	-0.051897	-1.236430	-2.842944
18	6	0	1.438603	-1.280172	-2.634029
19	6	0	1.985622	-2.347987	-1.638855
20	6	0	0.979922	-2.549918	-0.531455
21	6	0	1.242043	-2.291698	0.790294
22	6	0	0.176281	-2.353633	1.763368
23	6	0	0.115235	-1.336774	2.808886
24	6	0	1.035667	-0.202633	2.669393
25	6	0	0.538973	1.046708	3.060601
26	6	0	0.841462	2.198166	2.263326
27	6	0	1.747034	2.077033	1.271266
28	6	0	-0.354836	2.987018	2.087275
29	6	0	-1.329646	2.446680	2.970437
30	6	0	-0.781577	1.246524	3.584831
31	6	0	-1.591454	0.167633	3.831513
32	6	0	-1.128624	-1.147513	3.445184
33	6	0	-2.311297	-1.895009	3.065470
34	6	0	-2.235573	-2.835828	2.061056
35	6	0	-0.974772	-3.042430	1.399184
36	6	0	-1.248122	-3.301619	0.004276
37	6	0	-0.356758	-2.867706	-0.965811
38	6	0	-0.870612	-2.255295	-2.192249
39	6	0	-2.262453	-2.227495	-2.352369
40	6	0	-2.895359	-1.096165	-2.967422
41	6	0	-2.100556	-0.052241	-3.390093
42	6	0	-2.512894	1.304957	-3.120828
43	6	0	-3.664139	1.558966	-2.414341
44	6	0	-3.600938	2.514121	-1.337047
45	6	0	-4.533465	2.072442	-0.334309
46	6	0	-4.252193	2.325006	0.986524
47	6	0	-3.034698	3.026244	1.350798
48	6	0	-2.665453	2.521655	2.653469
49	6	0	-3.541216	1.420473	3.009499
50	6	0	-3.020690	0.266190	3.588309
51	6	0	-3.475243	-1.025734	3.139215
52	6	0	-4.489540	-1.128321	2.191371
53	6	0	-4.405135	-2.130293	1.144063
54	6	0	-3.299754	-2.967174	1.088996
55	6	0	-2.676000	-3.255001	-0.181276
56	6	0	-3.179968	-2.729962	-1.351424
57	6	0	-4.345369	-1.867394	-1.305467
58	6	0	-4.160259	-0.840924	-2.303749
59	6	0	-4.533598	0.468535	-2.024026
60	6	0	-5.101317	0.798893	-0.740050
61	6	0	-5.322465	-0.193024	0.206299
62	6	0	-5.043715	0.074744	1.605295
63	6	0	-4.552658	1.318716	1.989278
64	6	0	-4.933319	-1.559693	-0.080754
65	6	0	2.130073	-3.670062	-2.409749
66	6	0	2.754385	-4.759092	-1.795339
67	6	0	2.854553	-5.948490	-2.501255
68	6	0	2.334099	-6.011075	-3.792120
69	6	0	1.735166	-4.871827	-4.314209
70	6	0	3.143618	-2.455253	2.447410
71	6	0	2.917080	-3.823556	2.612943
72	6	0	3.548786	-4.481142	3.661184
73	6	0	4.386264	-3.756590	4.501894
74	6	0	4.555156	-2.399612	4.244930
75	16	0	0.068193	4.156413	-1.783823
76	7	0	1.629875	-3.721154	-3.642835
77	7	0	3.954434	-1.756955	3.243181
78	8	0	0.829229	1.770250	-4.018232
79	8	0	4.422525	0.763742	-0.598138

80	8	0	2.190096	-0.528413	-3.205536
81	8	0	3.614922	3.489962	-1.219651
82	15	0	4.093063	4.733981	-0.387415
83	6	0	5.058109	5.673240	-1.565754
84	6	0	2.658193	5.663131	0.180191
85	6	0	5.090966	4.270246	1.030236
86	1	0	4.120947	-1.835090	-1.840011
87	1	0	4.570536	-1.358062	0.474294
88	1	0	3.151284	-4.653497	-0.790241
89	1	0	3.331267	-6.815161	-2.053942
90	1	0	2.391127	-6.919216	-4.381404
91	1	0	1.318418	-4.875570	-5.318397
92	1	0	2.251094	-4.352928	1.939274
93	1	0	3.387100	-5.542866	3.819554
94	1	0	4.897349	-4.225231	5.335209
95	1	0	5.204875	-1.796090	4.874738
96	1	0	5.812862	5.000022	-1.980786
97	1	0	5.520543	6.539727	-1.089241
98	1	0	4.401204	5.972731	-2.384621
99	1	0	2.116847	6.044370	-0.688365
100	1	0	2.956149	6.486373	0.832925
101	1	0	2.011238	4.971938	0.728088
102	1	0	5.927487	3.662247	0.682332
103	1	0	5.435707	5.171638	1.544491
104	1	0	4.494335	3.641344	1.693645

The total electronic energy was calculated to be –4094.1726238 Hartree..

Supplementary Table 20. Optimized structure of **INT1-C2** (M06-2X/6-31G(d,p))

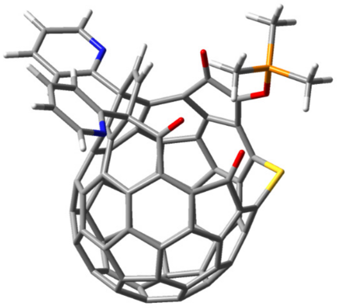

Standard orientation:

Center Number	Atomic Number	Atomic Type	Coordinates (Angstroms) X	Y	Z
1	6	0	-3.697979	-0.758567	0.460905
2	6	0	-3.555145	-0.947156	-0.845189
3	6	0	-2.350033	-1.569276	-1.499277
4	6	0	-1.537389	-0.542874	-2.308772
5	6	0	-1.812032	0.815594	-2.362667
6	6	0	-2.826724	1.812374	-1.750259
7	6	0	-2.058941	2.959957	-1.469057
8	6	0	-0.727533	2.881679	-1.953945
9	6	0	0.504512	3.434866	-1.618112
10	6	0	1.748987	2.839412	-2.067791
11	6	0	2.957061	2.796873	-1.224585
12	6	0	2.838787	3.098255	0.216359
13	6	0	1.567965	3.552132	0.768807
14	6	0	1.122305	3.049198	1.980510
15	6	0	-0.304295	2.627664	2.327865
16	6	0	-0.115234	1.248644	3.007675
17	6	0	-0.812827	0.064527	2.798024
18	6	0	-2.227030	0.043809	2.310027
19	6	0	-2.692288	-1.187538	1.490978
20	6	0	-1.506565	-1.884985	0.876569
21	6	0	-1.374225	-2.123232	-0.466355
22	6	0	-0.158773	-2.713765	-0.976332
23	6	0	0.384476	-2.237356	-2.243736
24	6	0	-0.247062	-1.046615	-2.821765
25	6	0	0.604685	-0.164906	-3.497275
26	6	0	0.400786	1.240832	-3.338047
27	6	0	-0.721180	1.671586	-2.733645
28	6	0	1.664222	1.889679	-3.102980
29	6	0	2.669604	0.916140	-3.363311
30	6	0	2.019991	-0.359982	-3.623911
31	6	0	2.587484	-1.525761	-3.181345
32	6	0	1.750443	-2.487923	-2.497475
33	6	0	2.604615	-3.144686	-1.529028
34	6	0	2.078103	-3.567398	-0.328659
35	6	0	0.683656	-3.335141	-0.058770
36	6	0	0.553864	-3.051050	1.350334
37	6	0	-0.428287	-2.178030	1.788642
38	6	0	-0.096243	-1.199566	2.825919
39	6	0	1.196606	-1.239359	3.362715
40	6	0	1.887407	-0.018482	3.665399
41	6	0	1.255161	1.178077	3.389885
42	6	0	1.994667	2.247529	2.764335
43	6	0	3.314039	2.073033	2.421347
44	6	0	3.736421	2.497711	1.111158
45	6	0	4.771079	1.590735	0.691380
46	6	0	4.895394	1.303726	-0.646504
47	6	0	4.002870	1.921458	-1.608766
48	6	0	3.863203	0.964628	-2.684690
49	6	0	4.519779	-0.275875	-2.314010
50	6	0	3.900611	-1.499041	-2.559442
51	6	0	3.922251	-2.527815	-1.550973
52	6	0	4.628328	-2.339261	-0.366390
53	6	0	4.072341	-2.802377	0.891365
54	6	0	2.826475	-3.413111	0.901525
55	6	0	1.872286	-3.091357	1.934988
56	6	0	2.192287	-2.197016	2.935075
57	6	0	3.491989	-1.552416	2.933436
58	6	0	3.298094	-0.190373	3.374286
59	6	0	3.997815	0.840058	2.757382
60	6	0	4.923748	0.548684	1.691183
61	6	0	5.145866	-0.763902	1.293974
62	6	0	5.283068	-1.071892	-0.118057
63	6	0	5.198103	-0.054583	-1.062933
64	6	0	4.407662	-1.839927	1.924570
65	6	0	-3.348055	-2.185644	2.462026
66	6	0	-3.974122	-3.321431	1.940324
67	6	0	-4.535423	-4.222653	2.832994

68	6	0	-4.449588	-3.964077	4.199766
69	6	0	-3.791108	-2.812687	4.611477
70	6	0	-2.868377	-2.722649	-2.372978
71	6	0	-3.138329	-3.990866	-1.860922
72	6	0	-3.559894	-4.987400	-2.735355
73	6	0	-3.695763	-4.686751	-4.084269
74	6	0	-3.390243	-3.392959	-4.499079
75	16	0	0.525473	4.558930	-0.241212
76	7	0	-3.244685	-1.934243	3.764503
77	7	0	-2.981913	-2.431555	-3.673445
78	8	0	-1.326833	3.188009	2.026611
79	8	0	-4.053456	1.690241	-1.541698
80	8	0	-2.997770	0.937144	2.568710
81	8	0	-2.563938	4.099098	-0.831841
82	15	0	-3.962628	4.265203	-0.107329
83	6	0	-3.699187	5.832228	0.738434
84	6	0	-5.295397	4.457024	-1.304279
85	6	0	-4.367768	3.033927	1.118628
86	1	0	-4.591662	-0.279255	0.849480
87	1	0	-4.322398	-0.590596	-1.523068
88	1	0	-3.989455	-3.483209	0.864539
89	1	0	-5.029264	-5.118936	2.470376
90	1	0	-4.874059	-4.642760	4.931027
91	1	0	-3.690438	-2.579355	5.668669
92	1	0	-3.023574	-4.188567	-0.801188
93	1	0	-3.780139	-5.983281	-2.363853
94	1	0	-4.025532	-5.429532	-4.801702
95	1	0	-3.478383	-3.115920	-5.547319
96	1	0	-4.597925	6.126694	1.284629
97	1	0	-2.868531	5.691314	1.434596
98	1	0	-3.435068	6.604016	0.013312
99	1	0	-5.011343	5.214601	-2.037912
100	1	0	-6.206149	4.770348	-0.786535
101	1	0	-5.436642	3.490197	-1.791016
102	1	0	-3.539666	2.946425	1.824832
103	1	0	-5.276378	3.359034	1.635593
104	1	0	-4.525724	2.078511	0.617198

The total electronic energy was calculated to be –4094.1892021 Hartree..

Supplementary Table 21. Optimized structure of **INT1-D** (M06-2X/6-31G(d,p))

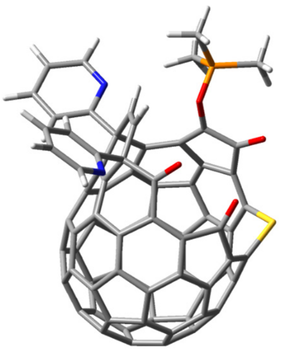

Standard orientation:

Center Number	Atomic Number	Atomic Type	Coordinates (Angstroms) X	Y	Z
1	6	0	-3.403875	0.980880	-0.990124
2	6	0	-3.544620	0.406845	0.199497
3	6	0	-2.554026	0.515369	1.327112
4	6	0	-1.832192	-0.820719	1.581734
5	6	0	-1.980312	-1.958598	0.806124
6	6	0	-2.740692	-2.394685	-0.468592
7	6	0	-1.833104	-3.231303	-1.149148
8	6	0	-0.648018	-3.492584	-0.417411
9	6	0	0.671802	-3.820134	-0.715387
10	6	0	1.735252	-3.621426	0.249996
11	6	0	3.080279	-3.160370	-0.137394
12	6	0	3.285884	-2.602457	-1.489266
13	6	0	2.202005	-2.626521	-2.464876
14	6	0	1.963143	-1.516382	-3.259002
15	6	0	0.602518	-0.921660	-3.615791
16	6	0	0.794711	0.593909	-3.358678
17	6	0	-0.037600	1.484715	-2.690983
18	6	0	-1.518590	1.293773	-2.603500
19	6	0	-2.259542	1.878711	-1.368155
20	6	0	-1.290772	2.079267	-0.229180
21	6	0	-1.449999	1.514371	1.011426
22	6	0	-0.419035	1.668668	2.012685
23	6	0	-0.106443	0.544799	2.889249
24	6	0	-0.730822	-0.740169	2.559009
25	6	0	0.045386	-1.879105	2.801057
26	6	0	0.010038	-2.947517	1.852291
27	6	0	-0.916004	-2.919104	0.876412
28	6	0	1.350163	-3.402694	1.586325
29	6	0	2.186240	-2.778129	2.553865
30	6	0	1.380755	-1.840350	3.322605
31	6	0	1.916064	-0.648927	3.736955
32	6	0	1.150541	0.560904	3.531943
33	6	0	2.119469	1.613863	3.301650
34	6	0	1.812043	2.655705	2.455044
35	6	0	0.528964	2.665886	1.803191
36	6	0	0.713609	3.220967	0.483099
37	6	0	-0.076899	2.782633	-0.567136
38	6	0	0.549694	2.526398	-1.864887
39	6	0	1.914407	2.810191	-1.991800
40	6	0	2.762379	1.940278	-2.755517
41	6	0	2.201466	0.816868	-3.330348
42	6	0	2.896360	-0.445259	-3.255790
43	6	0	4.097676	-0.539056	-2.594448
44	6	0	4.285343	-1.637364	-1.681036
45	6	0	5.123539	-1.156440	-0.615696
46	6	0	4.945562	-1.668375	0.647050
47	6	0	3.938919	-2.685224	0.885014
48	6	0	3.493201	-2.485238	2.246220
49	6	0	4.092569	-1.273858	2.776159
50	6	0	3.325122	-0.373247	3.510570
51	6	0	3.456612	1.041269	3.270569
52	6	0	4.403207	1.519742	2.368935
53	6	0	4.073866	2.625777	1.489465

54	6	0	2.804826	3.184840	1.543841
55	6	0	2.114227	3.529991	0.324946
56	6	0	2.710742	3.331174	-0.902516
57	6	0	4.037277	2.747788	-0.971104
58	6	0	4.063220	1.870064	-2.118160
59	6	0	4.716605	0.646038	-2.035426
60	6	0	5.376718	0.259716	-0.812891
61	6	0	5.390693	1.118960	0.278607
62	6	0	5.209691	0.582662	1.615338
63	6	0	5.028645	-0.784714	1.796887
64	6	0	4.698687	2.390116	0.200968
65	6	0	-2.793766	3.264978	-1.772431
66	6	0	-3.809243	3.866421	-1.026217
67	6	0	-4.233123	5.136950	-1.391808
68	6	0	-3.633015	5.759833	-2.482371
69	6	0	-2.627007	5.077116	-3.156239
70	6	0	-3.345045	0.935053	2.575596
71	6	0	-3.501234	2.280115	2.918567
72	6	0	-4.280707	2.598544	4.023632
73	6	0	-4.879764	1.569879	4.742560
74	6	0	-4.673032	0.263515	4.310798
75	16	0	1.080907	-3.990773	-2.437067
76	7	0	-2.209194	3.856171	-2.813321
77	7	0	-3.927208	-0.055273	3.252280
78	8	0	-0.404735	-1.514046	-3.906930
79	8	0	-3.852433	-2.126589	-0.849256
80	8	0	-2.140899	0.744282	-3.481017
81	8	0	-2.049761	-3.707751	-2.231545
82	15	0	-5.561452	-2.675416	-0.548520
83	6	0	-6.644364	-2.071189	-1.854524
84	6	0	-5.503102	-4.474669	-0.622475
85	6	0	-6.218986	-2.109088	1.011849
86	1	0	-4.150354	0.822959	-1.763209
87	1	0	-4.388044	-0.247859	0.388060
88	1	0	-4.248287	3.336773	-0.186726
89	1	0	-5.020990	5.634165	-0.834632
90	1	0	-3.933899	6.750436	-2.803752
91	1	0	-2.129624	5.526415	-4.012352
92	1	0	-3.010921	3.053038	2.335519
93	1	0	-4.414060	3.634122	4.320780
94	1	0	-5.491426	1.770048	5.614956
95	1	0	-5.128295	-0.570502	4.840016
96	1	0	-7.661768	-2.440481	-1.708823
97	1	0	-6.633245	-0.979272	-1.808528
98	1	0	-6.267117	-2.394635	-2.826281
99	1	0	-5.098684	-4.778451	-1.590518
100	1	0	-6.513817	-4.875237	-0.505029
101	1	0	-4.852120	-4.814677	0.184948
102	1	0	-6.222878	-1.017144	1.018569
103	1	0	-7.238871	-2.495014	1.108411
104	1	0	-5.582002	-2.478418	1.815553

The total electronic energy was calculated to be –4094.1635881 Hartree..

Supplementary Table 22. Optimized structure of **TS1-A** (M06-2X/6-31G(d,p))

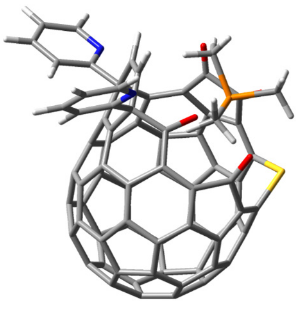

Standard orientation:

Center Number	Atomic Number	Atomic Type	Coordinates (Angstroms) X	Y	Z
1	6	0	3.819024	0.690177	-0.231308
2	6	0	3.408686	1.613621	-1.092922
3	6	0	2.110037	2.368854	-0.982886
4	6	0	1.029573	1.922020	-1.982519
5	6	0	1.124918	0.822073	-2.824545
6	6	0	2.123300	-0.279523	-3.085198
7	6	0	1.282785	-1.543493	-3.517271
8	6	0	-0.128144	-1.111834	-3.547967
9	6	0	-1.306126	-1.794338	-3.308961
10	6	0	-2.533054	-1.073546	-3.020611
11	6	0	-3.554889	-1.590916	-2.091568
12	6	0	-3.214230	-2.728445	-1.222084
13	6	0	-1.936405	-3.407511	-1.387847
14	6	0	-1.174044	-3.710986	-0.280258
15	6	0	0.341442	-3.515179	-0.131961
16	6	0	0.509829	-2.798461	1.152625
17	6	0	1.347686	-1.632586	1.461917
18	6	0	2.407960	-1.160386	0.745775
19	6	0	3.025504	0.219841	0.964114
20	6	0	1.852695	1.137280	1.210661
21	6	0	1.464312	2.148681	0.373125
22	6	0	0.230608	2.855971	0.620038
23	6	0	-0.624446	3.192046	-0.510659
24	6	0	-0.266253	2.585098	-1.803703
25	6	0	-1.331216	2.225047	-2.638259
26	6	0	-1.257087	1.004871	-3.377390
27	6	0	-0.089725	0.305991	-3.402770
28	6	0	-2.506571	0.308344	-3.268545
29	6	0	-3.437830	1.227555	-2.687540
30	6	0	-2.711477	2.420541	-2.298763
31	6	0	-3.036977	3.071236	-1.133587
32	6	0	-1.974908	3.480291	-0.236432
33	6	0	-2.523275	3.392760	1.103556
34	6	0	-1.708322	3.026083	2.152627
35	6	0	-0.320961	2.743897	1.892409
36	6	0	0.076016	1.658009	2.758328
37	6	0	0.997820	0.721466	2.295940
38	6	0	0.787003	-0.692635	2.503569
39	6	0	-0.327663	-1.053935	3.262506
40	6	0	-1.081976	-2.258115	2.991844
41	6	0	-0.694122	-2.980237	1.876350

42	6	0	-1.723199	-3.535827	1.015267
43	6	0	-3.046139	-3.224104	1.201548
44	6	0	-3.803076	-2.793198	0.050262
45	6	0	-4.754965	-1.823214	0.523094
46	6	0	-5.119835	-0.787967	-0.303950
47	6	0	-4.524156	-0.679390	-1.619222
48	6	0	-4.454715	0.741769	-1.904037
49	6	0	-4.879573	1.491008	-0.739143
50	6	0	-4.179716	2.637264	-0.359906
51	6	0	-3.873823	2.857273	1.032235
52	6	0	-4.321777	1.958380	1.994682
53	6	0	-3.457533	1.582833	3.098382
54	6	0	-2.180851	2.114120	3.173585
55	6	0	-1.062945	1.269133	3.530527
56	6	0	-1.256617	-0.075330	3.787903
57	6	0	-2.592290	-0.632326	3.740437
58	6	0	-2.479513	-1.974920	3.215231
59	6	0	-3.468337	-2.461962	2.360309
60	6	0	-4.539736	-1.600524	1.946316
61	6	0	-4.675509	-0.317988	2.470688
62	6	0	-5.061922	0.774982	1.597250
63	6	0	-5.318138	0.543825	0.250887
64	6	0	-3.673192	0.175686	3.387844
65	6	0	3.971986	0.220294	2.181359
66	6	0	4.157333	1.371440	2.950098
67	6	0	5.046420	1.333724	4.015325
68	6	0	5.734069	0.152487	4.276955
69	6	0	5.502169	-0.933492	3.442523
70	6	0	2.451350	3.843226	-1.247232
71	6	0	2.680848	4.750802	-0.213556
72	6	0	3.056277	6.048486	-0.541870
73	6	0	3.195852	6.391226	-1.881430
74	6	0	2.957202	5.407807	-2.837124
75	16	0	-1.223453	-3.537414	-3.010889
76	7	0	4.643395	-0.909559	2.418675
77	7	0	2.595956	4.161150	-2.535402
78	8	0	1.175098	-3.862117	-0.956606
79	8	0	3.327012	-0.319581	-3.076045
80	8	0	2.811542	-1.727753	-0.268628
81	8	0	1.789655	-2.600449	-3.788885
82	15	0	4.129823	-3.218919	-0.460884
83	6	0	4.058109	-4.025792	-2.055895
84	6	0	5.804143	-2.547085	-0.368490
85	6	0	3.897953	-4.313318	0.942874
86	1	0	4.785735	0.221439	-0.386686
87	1	0	4.021067	1.866833	-1.952436
88	1	0	3.600464	2.271709	2.711954
89	1	0	5.199559	2.211764	4.634882
90	1	0	6.433995	0.072879	5.100659
91	1	0	6.027556	-1.872873	3.602130
92	1	0	2.557988	4.446388	0.820150
93	1	0	3.234168	6.781196	0.239018
94	1	0	3.483350	7.391738	-2.183698
95	1	0	3.061272	5.630459	-3.896616
96	1	0	4.943495	-4.660967	-2.147216
97	1	0	3.136415	-4.593179	-2.161041
98	1	0	4.070337	-3.241922	-2.819760
99	1	0	5.972475	-1.906102	-1.237495
100	1	0	6.511809	-3.380813	-0.396407
101	1	0	5.927232	-1.986593	0.558994
102	1	0	2.915607	-4.780338	0.868586
103	1	0	4.688194	-5.068265	0.944885
104	1	0	3.961381	-3.696635	1.844626

The total electronic energy was calculated to be –4094.1442412 Hartree..

An imaginary frequency was found at 551.7125 cm^{-1}.

Supplementary Table 23. Optimized structure of **TS1-B** (M06-2X/6-31G(d,p))

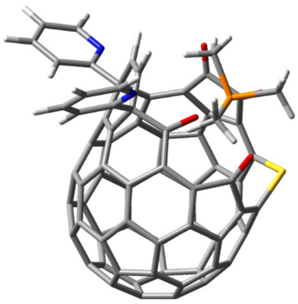

Standard orientation:

Center Number	Atomic Number	Atomic Type	Coordinates (Angstroms) X	Y	Z
1	6	0	-3.890910	0.057505	-0.146124
2	6	0	-3.647786	-0.672282	-1.229409
3	6	0	-2.542142	-1.687369	-1.349733
4	6	0	-1.420332	-1.221848	-2.296724
5	6	0	-1.352338	0.032157	-2.894646
6	6	0	-2.160868	1.309148	-2.901848
7	6	0	-1.128779	2.485949	-3.098589
8	6	0	0.185488	1.859064	-3.240128
9	6	0	1.444380	2.275433	-2.855162
10	6	0	2.543357	1.333558	-2.773463
11	6	0	3.621306	1.458373	-1.775525
12	6	0	3.433627	2.377732	-0.627651
13	6	0	2.261083	3.221956	-0.573086
14	6	0	1.471175	3.247458	0.588750
15	6	0	0.040632	3.164190	0.817602
16	6	0	-0.203282	2.316323	1.923957
17	6	0	-1.178659	1.290951	2.150601
18	6	0	-2.470444	1.296201	1.489468
19	6	0	-3.137412	-0.068743	1.150025
20	6	0	-2.089629	-1.152821	1.091553
21	6	0	-1.857619	-1.932679	-0.014033
22	6	0	-0.755306	-2.865001	-0.020150
23	6	0	0.018900	-3.045685	-1.242900
24	6	0	-0.250025	-2.103522	-2.340312
25	6	0	0.848245	-1.736967	-3.130104
26	6	0	0.965884	-0.385243	-3.574153
27	6	0	-0.078445	0.466811	-3.406626
28	6	0	2.308295	0.072329	-3.349037
29	6	0	3.085954	-1.085228	-3.042054

30	6	0	2.183182	-2.212923	-2.907371
31	6	0	2.410554	-3.165651	-1.946840
32	6	0	1.305527	-3.605060	-1.119362
33	6	0	1.880049	-3.939976	0.168677
34	6	0	1.145479	-3.730985	1.315007
35	6	0	-0.179285	-3.181580	1.207263
36	6	0	-0.393012	-2.323130	2.350991
37	6	0	-1.198903	-1.201535	2.224207
38	6	0	-0.752813	0.071433	2.800324
39	6	0	0.424421	0.041273	3.566238
40	6	0	1.349202	1.138835	3.462022
41	6	0	1.067112	2.191878	2.622862
42	6	0	2.084738	2.753942	1.804761
43	6	0	3.368981	2.253777	1.830294
44	6	0	4.055655	2.035118	0.589108
45	6	0	4.861638	0.857182	0.757278
46	6	0	5.074191	0.021670	-0.312690
47	6	0	4.447442	0.329621	-1.581294
48	6	0	4.164041	-0.955140	-2.201914
49	6	0	4.483246	-2.021684	-1.279093
50	6	0	3.616235	-3.108149	-1.147210
51	6	0	3.299746	-3.627074	0.158854
52	6	0	3.893151	-3.068043	1.287344
53	6	0	3.121260	-2.869711	2.498313
54	6	0	1.772222	-3.198999	2.509127
55	6	0	0.809313	-2.337094	3.147099
56	6	0	1.215214	-1.151673	3.729299
57	6	0	2.624083	-0.796724	3.729052
58	6	0	2.705384	0.635246	3.553376
59	6	0	3.697021	1.183034	2.748226
60	6	0	4.645861	0.315994	2.089816
61	6	0	4.590402	-1.056541	2.279583
62	6	0	4.803130	-1.947346	1.150417
63	6	0	5.063809	-1.418223	-0.106987
64	6	0	3.553505	-1.630289	3.115752
65	6	0	-4.125210	-0.426895	2.275070
66	6	0	-5.059689	-1.446284	2.071707
67	6	0	-5.912204	-1.782003	3.113474
68	6	0	-5.804887	-1.092796	4.318298
69	6	0	-4.840962	-0.096840	4.419650
70	6	0	-3.180258	-2.966382	-1.912001
71	6	0	-3.610734	-3.998817	-1.076989
72	6	0	-4.247095	-5.093297	-1.650213
73	6	0	-4.434263	-5.118127	-3.027701
74	6	0	-3.977693	-4.033531	-3.770223
75	16	0	1.633227	3.867466	-2.107516
76	7	0	-4.014082	0.235238	3.424714
77	7	0	-3.366424	-2.977158	-3.232982
78	8	0	-0.821809	3.370375	-0.008171
79	8	0	-3.339546	1.547210	-2.859697
80	8	0	-3.076706	2.321697	1.189856
81	8	0	-1.461014	3.648139	-3.141309
82	15	0	-2.067093	4.927115	-0.129809
83	6	0	-1.232285	6.255713	-1.015100
84	6	0	-3.655580	4.605541	-0.900987
85	6	0	-2.309175	5.481626	1.566111
86	1	0	-4.685693	0.797421	-0.158118
87	1	0	-4.237637	-0.527105	-2.128200
88	1	0	-5.106815	-1.951064	1.111616
89	1	0	-6.648635	-2.569686	2.988875
90	1	0	-6.449549	-1.320674	5.159595
91	1	0	-4.721313	0.464282	5.343416
92	1	0	-3.439568	-3.945034	-0.006954
93	1	0	-4.587463	-5.915974	-1.029157
94	1	0	-4.920282	-5.953551	-3.518609
95	1	0	-4.107909	-4.009827	-4.849690
96	1	0	-1.816469	7.175439	-0.925881
97	1	0	-0.243445	6.404462	-0.573892
98	1	0	-1.122511	5.966143	-2.061595
99	1	0	-3.506479	4.160195	-1.886441
100	1	0	-4.164487	5.568340	-1.007147
101	1	0	-4.221875	3.930949	-0.260867
102	1	0	-1.349576	5.756871	2.005721
103	1	0	-2.974158	6.349616	1.543253
104	1	0	-2.761783	4.664315	2.129179

The total electronic energy was calculated to be –4094.144264 Hartree..

An imaginary frequency was found at 673.2618 cm^{-1}.

Supplementary Table 24. Optimized structure of **TS1-C1** (M06-2X/6-31G(d,p))

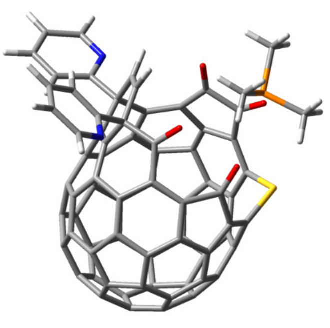

Standard orientation:

Center Number	Atomic Number	Atomic Type	Coordinates (Angstroms) X	Y	Z
1	6	0	-3.636111	-0.956240	0.432352
2	6	0	-3.470963	-1.171501	-0.867998
3	6	0	-2.227039	-1.744477	-1.491633
4	6	0	-1.464326	-0.689516	-2.314028
5	6	0	-1.804146	0.651135	-2.387898
6	6	0	-2.885284	1.569051	-1.797079
7	6	0	-2.134451	2.841562	-1.538298
8	6	0	-0.825318	2.781600	-2.029886
9	6	0	0.375961	3.408287	-1.710202
10	6	0	1.649275	2.862784	-2.138538
11	6	0	2.852335	2.898776	-1.288189
12	6	0	2.708831	3.227201	0.144515
13	6	0	1.413173	3.632747	0.677423
14	6	0	0.981008	3.133925	1.895828
15	6	0	-0.425584	2.648284	2.239255
16	6	0	-0.174444	1.294772	2.949838
17	6	0	-0.810430	0.072907	2.763652

18	6	0	-2.220970	-0.036313	2.277968
19	6	0	-2.624805	-1.311623	1.485666
20	6	0	-1.399110	-1.969313	0.901611
21	6	0	-1.238926	-2.223184	-0.437589
22	6	0	0.012048	-2.757165	-0.926611
23	6	0	0.536419	-2.282344	-2.202831
24	6	0	-0.151322	-1.139581	-2.812057
25	6	0	0.660818	-0.230724	-3.499566
26	6	0	0.386235	1.166253	-3.373194
27	6	0	-0.756799	1.552140	-2.777152
28	6	0	1.615257	1.882315	-3.148208
29	6	0	2.669185	0.954476	-3.379706
30	6	0	2.084749	-0.358213	-3.613111
31	6	0	2.707625	-1.484492	-3.142735
32	6	0	1.915207	-2.470367	-2.441400
33	6	0	2.794260	-3.060534	-1.451681
34	6	0	2.282470	-3.484301	-0.245372
35	6	0	0.876120	-3.316456	0.010196
36	6	0	0.720389	-3.006509	1.411813
37	6	0	-0.310392	-2.178022	1.825493
38	6	0	-0.032976	-1.153146	2.833016
39	6	0	1.255097	-1.114773	3.379351
40	6	0	1.881911	0.145878	3.656714
41	6	0	1.193619	1.302380	3.347096
42	6	0	1.884664	2.393434	2.704090
43	6	0	3.214317	2.276659	2.375885
44	6	0	3.627173	2.691788	1.059435
45	6	0	4.707916	1.827047	0.668246
46	6	0	4.855574	1.514818	-0.661788
47	6	0	3.941980	2.065695	-1.644550
48	6	0	3.855610	1.077803	-2.697237
49	6	0	4.569737	-0.119904	-2.293452
50	6	0	4.013399	-1.377519	-2.513289
51	6	0	4.079889	-2.380019	-1.480662
52	6	0	4.767251	-2.129593	-0.296288
53	6	0	4.225940	-2.590671	0.968479
54	6	0	3.012094	-3.263177	0.985338
55	6	0	2.035285	-2.966076	2.004818
56	6	0	2.301412	-2.031715	2.983678
57	6	0	3.567277	-1.323010	2.975921
58	6	0	3.301769	0.037760	3.381658
59	6	0	3.954732	1.087061	2.745573
60	6	0	4.902968	0.817110	1.693167
61	6	0	5.193018	-0.491736	1.328941
62	6	0	5.356489	-0.825777	-0.074241
63	6	0	5.227257	0.163578	-1.043740
64	6	0	4.503988	-1.588026	1.980187
65	6	0	-3.243932	-2.296676	2.494091
66	6	0	-4.037236	-3.352711	2.041077
67	6	0	-4.539826	-4.248668	2.975015
68	6	0	-4.235499	-4.061452	4.320294
69	6	0	-3.433034	-2.980764	4.667301
70	6	0	-2.675988	-2.899426	-2.399980
71	6	0	-2.740130	-4.212446	-1.927667
72	6	0	-3.214245	-5.202977	-2.778671
73	6	0	-3.611716	-4.851206	-4.064032
74	6	0	-3.521168	-3.512882	-4.433397
75	16	0	0.335604	4.567953	-0.363275
76	7	0	-2.942054	-2.113552	3.778622
77	7	0	-3.067815	-2.552941	-3.626350
78	8	0	-1.473768	3.147474	1.919315
79	8	0	-4.031678	1.435003	-1.565684
80	8	0	-3.045294	0.811208	2.525842
81	8	0	-2.661611	3.801630	-0.988937
82	15	0	-4.389270	4.176310	-0.053625
83	6	0	-4.226982	5.781592	0.743750
84	6	0	-5.721844	4.262882	-1.270213
85	6	0	-4.764108	2.973879	1.219906
86	1	0	-4.554081	-0.505019	0.797817
87	1	0	-4.239153	-0.865073	-1.569099
88	1	0	-4.249345	-3.452495	0.981320
89	1	0	-5.160834	-5.080835	2.658389
90	1	0	-4.607920	-4.735412	5.083308
91	1	0	-3.168704	-2.797541	5.705929
92	1	0	-2.413453	-4.446980	-0.919680
93	1	0	-3.267921	-6.234261	-2.443895
94	1	0	-3.982436	-5.590803	-4.764630
95	1	0	-3.827578	-3.193284	-5.426821
96	1	0	-5.145325	6.047229	1.271763
97	1	0	-3.397108	5.708914	1.451247
98	1	0	-3.996096	6.540895	-0.005512
99	1	0	-5.460943	5.007841	-2.025138
100	1	0	-6.652723	4.552764	-0.775008
101	1	0	-5.813336	3.275521	-1.726352
102	1	0	-3.932643	2.943217	1.927102
103	1	0	-5.683644	3.286211	1.725208
104	1	0	-4.885270	1.996031	0.753831

The total electronic energy was calculated to be –4094.141941 Hartree..

An imaginary frequency was found at 1142.3092 cm^{-1}.

Supplementary Table 25. Optimized structure of **TS1-C2** (M06-2X/6-31G(d,p))

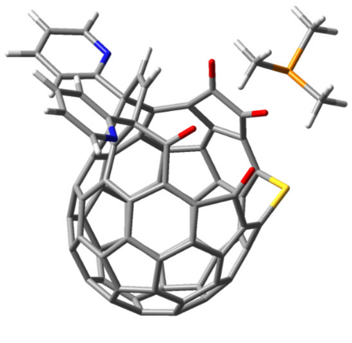

Standard orientation:

Center Number	Atomic Number	Atomic Type	X	Y	Z
1	6	0	3.578541	-1.202810	-0.518673
2	6	0	3.438326	-1.522716	0.762922
3	6	0	2.163027	-2.025071	1.385825
4	6	0	1.482784	-0.949560	2.250837
5	6	0	1.896760	0.367380	2.314804
6	6	0	2.953761	1.219849	1.617443
7	6	0	2.287261	2.479384	1.275457
8	6	0	1.038466	2.577569	2.008039
9	6	0	-0.125809	3.290656	1.755397
10	6	0	-1.414726	2.825156	2.228756
11	6	0	-2.646038	2.979844	1.431097
12	6	0	-2.536651	3.376194	0.013765
13	6	0	-1.242800	3.743112	-0.547855
14	6	0	-0.877853	3.283127	-1.799008
15	6	0	0.503743	2.777486	-2.232322
16	6	0	0.169682	1.415915	-2.903857
17	6	0	0.744742	0.162246	-2.732636
18	6	0	2.128326	0.004174	-2.170690
19	6	0	2.504019	-1.376173	-1.554769
20	6	0	1.265346	-2.027514	-0.988872
21	6	0	1.124899	-2.370901	0.332329
22	6	0	-0.146716	-2.846759	0.825906
23	6	0	-0.615983	-2.391777	2.132572
24	6	0	0.157650	-1.317259	2.767027
25	6	0	-0.573795	-0.364925	3.486584
26	6	0	-0.211969	1.016359	3.378849
27	6	0	0.939889	1.350313	2.747566
28	6	0	-1.397063	1.808350	3.199526
29	6	0	-2.506084	0.944020	3.451276
30	6	0	-2.000555	-0.406010	3.631178
31	6	0	-2.706288	-1.475979	3.140808
32	6	0	-1.997766	-2.491843	2.390317
33	6	0	-2.933021	-2.985889	1.399265
34	6	0	-2.473225	-3.387195	0.163933
35	6	0	-1.064923	-3.295128	-0.116885
36	6	0	-0.917932	-2.917962	-1.503458
37	6	0	0.144995	-2.115822	-1.892337
38	6	0	-0.091766	-1.025880	-2.840716
39	6	0	-1.382739	-0.909336	-3.371257
40	6	0	-1.953586	0.386824	-3.601642
41	6	0	-1.204047	1.499529	-3.281402
42	6	0	-1.833501	2.605854	-2.598769
43	6	0	-3.157615	2.538160	-2.233842
44	6	0	-3.509634	2.923352	-0.890086
45	6	0	-4.618516	2.096269	-0.495627
46	6	0	-4.743463	1.740925	0.826096
47	6	0	-3.767520	2.199576	1.794831
48	6	0	-3.697045	1.162520	2.804736
49	6	0	-4.499778	0.029189	2.388192
50	6	0	-4.013664	-1.266062	2.548753
51	6	0	-4.170458	-2.226707	1.484242
52	6	0	-4.868018	-1.888555	0.329205
53	6	0	-4.384466	-2.328805	-0.966578
54	6	0	-3.211899	-3.067005	-1.038979
55	6	0	-2.238637	-2.779683	-2.065273
56	6	0	-2.468632	-1.788180	-2.995002
57	6	0	-3.696747	-1.019295	-2.936019
58	6	0	-3.372537	0.339438	-3.301472
59	6	0	-3.961731	1.397216	-2.618814
60	6	0	-4.894105	1.136148	-1.549607
61	6	0	-5.243437	-0.167957	-1.224166
62	6	0	-5.388381	-0.545089	0.170748
63	6	0	-5.176461	0.397982	1.170515
64	6	0	-4.627707	-1.273490	-1.931319
65	6	0	3.030771	-2.266727	-2.693184
66	6	0	3.697175	-3.455843	-2.388257
67	6	0	4.129890	-4.257388	-3.435069
68	6	0	3.884865	-3.846790	-4.742797
69	6	0	3.212643	-2.646647	-4.939662
70	6	0	2.528239	-3.243299	2.244501
71	6	0	2.497422	-4.537974	1.721848
72	6	0	2.912803	-5.589859	2.529021
73	6	0	3.347030	-5.315207	3.821084
74	6	0	3.348856	-3.989394	4.243239
75	16	0	-0.045130	4.528377	0.488520
76	7	0	2.789550	-1.867539	-3.940487
77	7	0	2.952730	-2.971032	3.478515
78	8	0	1.566650	3.295902	-2.043002
79	8	0	4.156689	1.057718	1.459515
80	8	0	2.934175	0.901708	-2.193079
81	8	0	2.850011	3.345927	0.522733
82	15	0	4.670565	4.014328	0.531657
83	6	0	5.350928	2.800285	-0.636223
84	6	0	4.182814	5.434106	-0.502089
85	6	0	6.166874	4.637377	1.444868
86	1	0	4.512727	-0.777301	-0.872467
87	1	0	4.252994	-1.347509	1.457842
88	1	0	3.869539	-3.725457	-1.350780
89	1	0	4.651545	-5.188140	-3.235519
90	1	0	4.205403	-4.440172	-5.591489
91	1	0	3.001528	-2.287650	-5.943915
92	1	0	2.146053	-4.711297	0.709551
93	1	0	2.894820	-6.608720	2.154956
94	1	0	3.676529	-6.104140	4.487579
95	1	0	3.683641	-3.729489	5.244755
96	1	0	6.102942	3.275869	-1.273096
97	1	0	5.789979	1.971603	-0.079988
98	1	0	4.529977	2.413676	-1.245735
99	1	0	3.334842	5.120390	-1.115492
100	1	0	5.009704	5.752343	-1.142981

101	1	0	3.876337	6.269318	0.131575
102	1	0	6.583962	3.820487	2.036766
103	1	0	6.925242	5.001834	0.741977
104	1	0	5.876237	5.448818	2.114875

The total electronic energy was calculated to be –4094.1348925 Hartree..

An imaginary frequency was found at 1611.7894 cm^{-1}.

Supplementary Table 26. Optimized structure of **TS1-D** (M06-2X/6-31G(d,p))

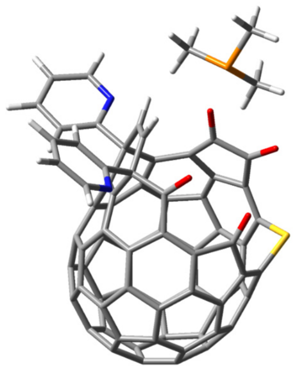

Standard orientation:

Center Number	Atomic Number	Atomic Type	Coordinates (Angstroms) X	Y	Z
1	6	0	-3.377789	1.250544	-1.027796
2	6	0	-3.564924	0.724901	0.176327
3	6	0	-2.570141	0.738714	1.305789
4	6	0	-1.913682	-0.635963	1.555961
5	6	0	-2.088611	-1.753782	0.722899
6	6	0	-2.756516	-2.157414	-0.485295
7	6	0	-2.065831	-3.184920	-1.209284
8	6	0	-0.834092	-3.451852	-0.373118
9	6	0	0.464841	-3.777397	-0.688427
10	6	0	1.532103	-3.627200	0.292308
11	6	0	2.906736	-3.258027	-0.075368
12	6	0	3.169301	-2.750296	-1.434234
13	6	0	2.097009	-2.734375	-2.422252
14	6	0	1.926875	-1.624179	-3.229747
15	6	0	0.592960	-0.969908	-3.619259
16	6	0	0.856074	0.537786	-3.356909
17	6	0	0.054916	1.467855	-2.706626
18	6	0	-1.431517	1.299001	-2.604106
19	6	0	-2.147966	2.014793	-1.420353
20	6	0	-1.196134	2.187854	-0.263655
21	6	0	-1.411786	1.670585	0.991318
22	6	0	-0.374870	1.768246	1.992208
23	6	0	-0.138321	0.638675	2.883839
24	6	0	-0.830028	-0.615191	2.543984
25	6	0	-0.113772	-1.790130	2.826881
26	6	0	-0.185405	-2.851406	1.866209
27	6	0	-1.082340	-2.802206	0.861508
28	6	0	1.126645	-3.365333	1.610702
29	6	0	1.982481	-2.793825	2.605744
30	6	0	1.212359	-1.810160	3.352519
31	6	0	1.811738	-0.647615	3.776737
32	6	0	1.114773	0.593344	3.538517
33	6	0	2.140800	1.594044	3.308675
34	6	0	1.900595	2.639214	2.445083
35	6	0	0.627913	2.710516	1.777890
36	6	0	0.857196	3.229919	0.451392
37	6	0	0.058645	2.809524	-0.601066
38	6	0	0.682533	2.495747	-1.888918
39	6	0	2.061144	2.713257	-2.003214
40	6	0	2.877023	1.795999	-2.745575
41	6	0	2.270855	0.694909	-3.317062
42	6	0	2.907984	-0.598243	-3.222875
43	6	0	4.097012	-0.739796	-2.545075
44	6	0	4.217285	-1.835335	-1.616913
45	6	0	5.057350	-1.381161	-0.539782
46	6	0	4.832546	-1.866520	0.727122
47	6	0	3.769518	-2.822121	0.955895
48	6	0	3.299927	-2.570847	2.305469
49	6	0	3.967769	-1.397607	2.842287
50	6	0	3.231270	-0.451392	3.550747
51	6	0	3.445934	0.953098	3.300861
52	6	0	4.426611	1.365573	2.404264
53	6	0	4.168669	2.476963	1.506704
54	6	0	2.930269	3.101453	1.537325
55	6	0	2.272735	3.464019	0.305218
56	6	0	2.870530	3.210827	-0.911307
57	6	0	4.167005	2.563092	-0.954269
58	6	0	4.165690	1.672500	-2.091022
59	6	0	4.760251	0.420541	-1.986027
60	6	0	5.383299	0.017853	-0.748628
61	6	0	5.423559	0.888131	0.333840
62	6	0	5.197967	0.379788	1.674428
63	6	0	4.939202	-0.973005	1.869301
64	6	0	4.796976	2.191372	0.231126
65	6	0	-2.562359	3.418554	-1.896286
66	6	0	-3.403015	4.194297	-1.093123
67	6	0	-3.739311	5.468801	-1.524189
68	6	0	-3.228261	5.924794	-2.736981
69	6	0	-2.395386	5.078172	-3.458174
70	6	0	-3.370209	1.157170	2.550880
71	6	0	-3.208676	2.387859	3.187194
72	6	0	-4.003030	2.684461	4.290132
73	6	0	-4.938998	1.752316	4.718157
74	6	0	-5.045064	0.562905	4.002926
75	16	0	0.864558	-4.006876	-2.404435
76	7	0	-2.062579	3.849393	-3.052483
77	7	0	-4.290176	0.265761	2.946486
78	8	0	-0.425538	-1.514663	-3.929543
79	8	0	-3.963220	-1.655358	-1.050753
80	8	0	-2.069216	0.663807	-3.404725
81	8	0	-2.410130	-3.760789	-2.241720
82	15	0	-5.528809	-2.777405	-0.513780
83	6	0	-6.946611	-1.825542	-1.085562
84	6	0	-5.537613	-4.383460	-1.314167
85	6	0	-5.603216	-2.959994	1.277122

86	1	0	-4.134947	1.145517	-1.798785
87	1	0	-4.497737	0.221185	0.406971
88	1	0	-3.771847	3.792461	-0.154243
89	1	0	-4.388038	6.099469	-0.924287
90	1	0	-3.465208	6.913109	-3.114255
91	1	0	-1.970555	5.396355	-4.407009
92	1	0	-2.469381	3.095151	2.830205
93	1	0	-3.886627	3.631918	4.807022
94	1	0	-5.575316	1.936848	5.576112
95	1	0	-5.774829	-0.189565	4.295616
96	1	0	-7.874080	-2.377036	-0.917891
97	1	0	-6.981799	-0.875797	-0.546997
98	1	0	-6.823967	-1.627054	-2.152674
99	1	0	-5.466398	-4.235841	-2.393417
100	1	0	-6.455823	-4.913467	-1.045847
101	1	0	-4.654075	-4.946760	-1.010508
102	1	0	-5.342241	-2.006549	1.757618
103	1	0	-6.611297	-3.269112	1.566707
104	1	0	-4.878923	-3.712364	1.594152

The total electronic energy was calculated to be –4094.1222801 Hartree..

An imaginary frequency was found at 1882.7421 cm^{-1}.

11.3. Phospha-Brook Rearrangement

As an alternative pathway to form **INT1-C1** from **1'**, the nucleophilic addition of PMe_3 to the carbon atom on **1'** followed by phospha-Brook rearrangement should be considered in addition to a pathway via direct nucleophilic addition to the oxygen atom (Supplementary Fig. 42). The results suggest that the alternative pathway is not preferable since the second step in the alternative pathway requires slightly higher energy than that of the direct addition pathway.

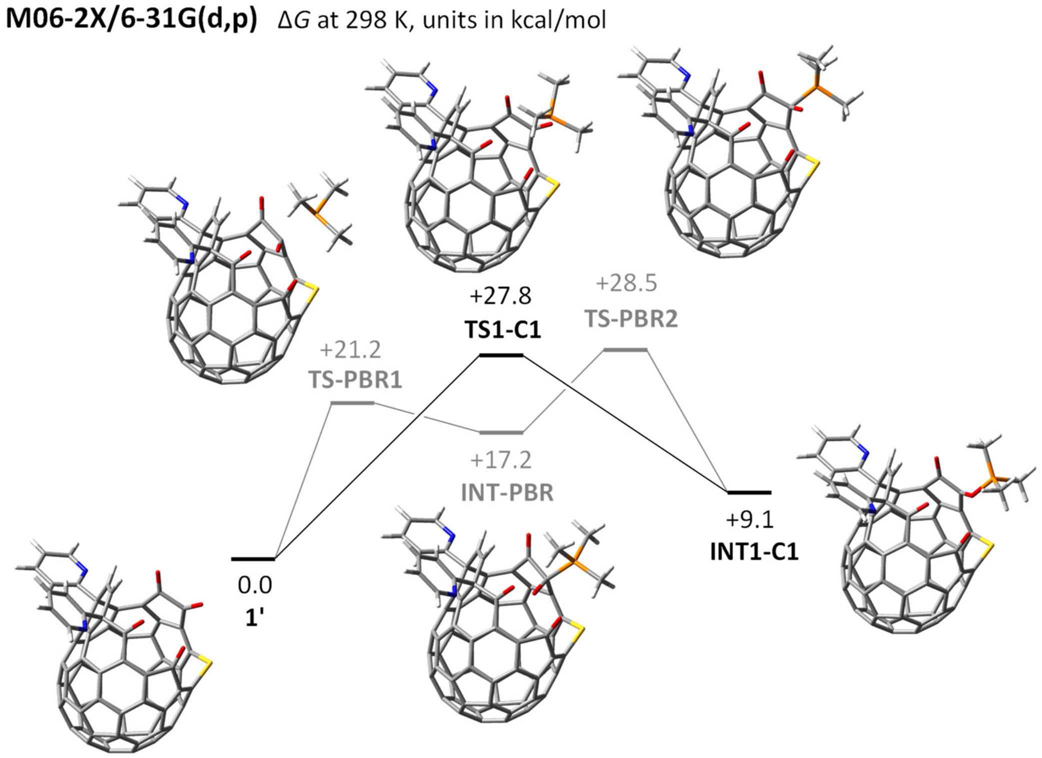

Supplementary Fig. 42. Reaction pathway for the formation of **INT-C1** from **1'** (M06-2X/6-31G(d,p)).

Supplementary Table 27. Optimized structure of **INT-PBR** (M06-2X/6-31G(d,p))

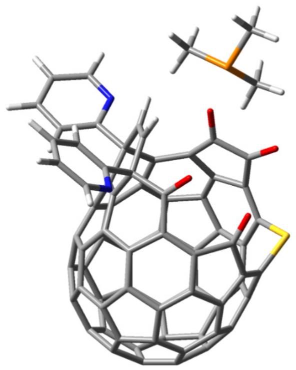

Standard orientation:

Center Number	Atomic Number	Atomic Type	Coordinates (Angstroms) X	Y	Z
1	6	0	3.663844	-0.606739	-1.027652
2	6	0	3.803769	-0.398246	0.277590
3	6	0	2.819067	-0.840466	1.328256
4	6	0	2.027348	0.350680	1.901362
5	6	0	2.125420	1.640604	1.411828
6	6	0	2.964395	2.384695	0.402359
7	6	0	1.972911	3.267931	-0.429181
8	6	0	0.727388	3.333196	0.450267
9	6	0	-0.596691	3.572773	0.149513
10	6	0	-1.672497	3.150109	1.037563
11	6	0	-3.001156	2.746152	0.536744
12	6	0	-3.193489	2.521305	-0.906919
13	6	0	-2.117164	2.819009	-1.843460
14	6	0	-1.845515	1.952041	-2.882109
15	6	0	-0.472500	1.552733	-3.454105
16	6	0	-0.547680	0.001421	-3.423637
17	6	0	0.342033	-0.932597	-2.913002
18	6	0	1.794409	-0.611105	-2.706963
19	6	0	2.573320	-1.440783	-1.638123
20	6	0	1.624684	-1.991707	-0.600526
21	6	0	1.758722	-1.761736	0.747168
22	6	0	0.735507	-2.209884	1.664699
23	6	0	0.350082	-1.348341	2.779074
24	6	0	0.911690	0.008489	2.779414
25	6	0	0.072222	1.036433	3.231055
26	6	0	0.065521	2.287936	2.537396
27	6	0	1.023720	2.545135	1.604917
28	6	0	-1.289316	2.688901	2.305538
29	6	0	-2.112795	1.841987	3.114761
30	6	0	-1.270440	0.810021	3.685638
31	6	0	-1.744768	-0.471846	3.808794
32	6	0	-0.911387	-1.571229	3.362938
33	6	0	-1.816708	-2.591059	2.874385
34	6	0	-1.440924	-3.387109	1.814206
35	6	0	-0.156157	-3.171564	1.203215
36	6	0	-0.299462	-3.401743	-0.215397
37	6	0	0.457167	-2.665647	-1.115642
38	6	0	-0.176239	-2.164572	-2.335619
39	6	0	-1.513159	-2.515830	-2.557560
40	6	0	-2.420743	-1.566026	-3.135471
41	6	0	-1.938233	-0.314920	-3.456167
42	6	0	-2.714415	0.854253	-3.116230
43	6	0	-3.914036	0.727589	-2.455843
44	6	0	-4.150459	1.581110	-1.318886
45	6	0	-4.955354	0.828366	-0.393957
46	6	0	-4.805785	1.054736	0.952865
47	6	0	-3.845020	2.033613	1.417199
48	6	0	-3.389029	1.553928	2.708816
49	6	0	-3.938072	0.238439	2.957154
50	6	0	-3.127160	-0.761203	3.491348
51	6	0	-3.181382	-2.095708	2.942218
52	6	0	-4.089102	-2.401643	1.935296
53	6	0	-3.692530	-3.260148	0.834491
54	6	0	-2.392674	-3.743579	0.784243
55	6	0	-1.674483	-3.756263	-0.467228
56	6	0	-2.276568	-3.316415	-1.626417
57	6	0	-3.638969	-2.821564	-1.587610
58	6	0	-3.722489	-1.717967	-2.514425
59	6	0	-4.455107	-0.585723	-2.175527
60	6	0	-5.130970	-0.519761	-0.903141
61	6	0	-5.094161	-1.598652	-0.029446
62	6	0	-4.947082	-1.363727	1.394977
63	6	0	-4.843552	-0.065021	1.877473
64	6	0	-4.325539	-2.777056	-0.376437
65	6	0	3.201681	-2.645062	-2.369125
66	6	0	4.274859	-3.333756	-1.799945
67	6	0	4.777748	-4.441419	-2.469356
68	6	0	4.197838	-4.821760	-3.675680
69	6	0	3.130535	-4.071974	-4.155412
70	6	0	3.617508	-1.533881	2.440155
71	6	0	3.890186	-2.902181	2.390424
72	6	0	4.680362	-3.457941	3.388842
73	6	0	5.171782	-2.633137	4.394676
74	6	0	4.851258	-1.279997	4.347549
75	16	0	-1.005325	4.147032	-1.481042
76	7	0	2.636266	-3.006974	-3.519862
77	7	0	4.093683	-0.733609	3.395423
78	8	0	0.407358	2.270590	-3.817304
79	8	0	4.166255	2.436821	0.291015
80	8	0	2.361357	0.235217	-3.347156
81	8	0	1.939425	3.111601	-1.669646
82	15	0	2.731351	4.751564	-0.317369
83	6	0	4.302995	4.599318	-1.225283
84	6	0	3.212882	5.349335	1.346411
85	6	0	1.596435	6.030720	-0.952173
86	1	0	4.382302	-0.190124	-1.727521
87	1	0	4.636103	0.188529	0.649695
88	1	0	4.696795	-2.996427	-0.859019
89	1	0	5.613076	-4.999195	-2.057700
90	1	0	4.563246	-5.676929	-4.232628
91	1	0	2.646957	-4.332284	-5.093549
92	1	0	3.485898	-3.510944	1.588024
93	1	0	4.906153	-4.519660	3.382483
94	1	0	5.786739	-3.024610	5.196968
95	1	0	5.220024	-0.599048	5.111043
96	1	0	4.161771	3.774884	-1.927985
97	1	0	5.106240	4.354114	-0.529157
98	1	0	4.539763	5.519182	-1.766421
99	1	0	3.737656	6.307680	1.300048
100	1	0	2.315979	5.455110	1.961354

101	1	0	3.863113	4.599446	1.802909
102	1	0	2.125064	6.934766	-1.268446
103	1	0	0.853632	6.288666	-0.193026
104	1	0	1.099660	5.585502	-1.819035

The total electronic energy was calculated to be –4094.1608809 Hartree..

Supplementary Table 28. Optimized structure of **TS-PBR1** (M06-2X/6-31G(d,p))

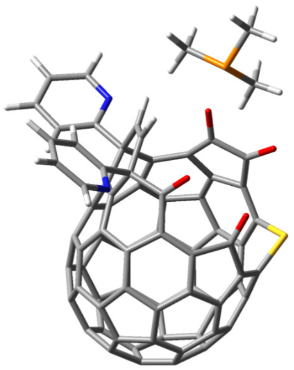

Standard orientation:

Center Number	Atomic Number	Atomic Type	Coordinates (Angstroms) X	Y	Z
1	6	0	3.547240	-1.108888	-0.917247
2	6	0	3.650741	-1.061830	0.407056
3	6	0	2.556960	-1.447912	1.368139
4	6	0	1.930905	-0.211693	2.041874
5	6	0	2.252434	1.089805	1.701452
6	6	0	3.247064	1.791211	0.809646
7	6	0	2.438971	2.900334	0.026036
8	6	0	1.184392	3.074092	0.885454
9	6	0	-0.069475	3.551582	0.567875
10	6	0	-1.241787	3.216085	1.366475
11	6	0	-2.589560	3.083258	0.778937
12	6	0	-2.740212	3.042851	-0.686459
13	6	0	-1.584250	3.260614	-1.547008
14	6	0	-1.397280	2.473840	-2.665154
15	6	0	-0.075176	1.922215	-3.230437
16	6	0	-0.391082	0.408042	-3.374090
17	6	0	0.315974	-0.703721	-2.939601
18	6	0	1.788135	-0.641017	-2.649740
19	6	0	2.373445	-1.690551	-1.653057
20	6	0	1.299086	-2.187748	-0.715729
21	6	0	1.397930	-2.123066	0.652912
22	6	0	0.272050	-2.495024	1.479781
23	6	0	-0.031890	-1.703288	2.668651
24	6	0	0.732245	-0.460796	2.838068
25	6	0	0.040034	0.635405	3.371494
26	6	0	0.262694	1.937050	2.820488
27	6	0	1.295631	2.133459	1.955457
28	6	0	-1.000060	2.570744	2.588115
29	6	0	-1.985068	1.786682	3.270192
30	6	0	-1.342985	0.580063	3.752080
31	6	0	-2.015727	-0.615553	3.716597
32	6	0	-1.340877	-1.781518	3.180819
33	6	0	-2.366955	-2.587744	2.551926
34	6	0	-2.065161	-3.319265	1.423791
35	6	0	-0.732937	-3.249145	0.884665
36	6	0	-0.837325	-3.305201	-0.554758
37	6	0	0.069232	-2.609840	-1.341774
38	6	0	-0.415656	-1.890441	-2.519897
39	6	0	-1.777699	-1.999126	-2.824889
40	6	0	-2.496499	-0.862041	-3.325071
41	6	0	-1.810333	0.322550	-3.488921
42	6	0	-2.412521	1.558893	-3.048686
43	6	0	-3.649508	1.557337	-2.447764
44	6	0	-3.808839	2.315101	-1.232251
45	6	0	-4.766857	1.608532	-0.424208
46	6	0	-4.653203	1.667291	0.943700
47	6	0	-3.577524	2.426983	1.545912
48	6	0	-3.268032	1.749443	2.791485
49	6	0	-4.026103	0.519753	2.874074
50	6	0	-3.408160	-0.646316	3.322021
51	6	0	-3.640147	-1.890889	2.627321
52	6	0	-4.531525	-1.942126	1.562304
53	6	0	-4.216811	-2.734063	0.387629
54	6	0	-3.006625	-3.410705	0.328799
55	6	0	-2.235933	-3.407915	-0.891105
56	6	0	-2.702511	-2.759923	-2.014539
57	6	0	-3.972051	-2.060912	-1.967951
58	6	0	-3.836142	-0.868052	-2.769513
59	6	0	-4.401014	0.325198	-2.332899
60	6	0	-5.122766	0.365797	-1.084887
61	6	0	-5.298217	-0.789852	-0.334958
62	6	0	-5.189668	-0.730341	1.110891
63	6	0	-4.911164	0.478133	1.737136
64	6	0	-4.704555	-2.033321	-0.783478
65	6	0	2.844379	-2.897248	-2.490669
66	6	0	3.767427	-3.803484	-1.964654
67	6	0	4.126373	-4.901709	-2.734711
68	6	0	3.557106	-5.057585	-3.994795
69	6	0	2.644733	-4.101581	-4.425013
70	6	0	3.180430	-2.371246	2.423311
71	6	0	3.240166	-3.752893	2.232114
72	6	0	3.882604	-4.528004	3.189487
73	6	0	4.443641	-3.900882	4.296489
74	6	0	4.339311	-2.516449	4.388167
75	16	0	-0.300247	4.349559	-1.002297
76	7	0	2.289420	-3.043105	-3.693007
77	7	0	3.725217	-1.760537	3.476855
78	8	0	0.922639	2.524345	-3.481808
79	8	0	4.446609	1.662310	0.745930
80	8	0	2.511299	0.165839	-3.172764
81	8	0	2.260438	2.631357	-1.182306
82	15	0	3.802990	4.744684	-0.140775
83	6	0	5.233169	4.241316	-1.150111
84	6	0	4.535202	5.416303	1.398758
85	6	0	2.826976	6.126144	-0.823575
86	1	0	4.356417	-0.741865	-1.541823
87	1	0	4.543725	-0.657046	0.869954

88	1	0	4.187764	-3.637134	-0.978344
89	1	0	4.843089	-5.625165	-2.358841
90	1	0	3.813744	-5.897664	-4.629957
91	1	0	2.175360	-4.182779	-5.402154
92	1	0	2.788126	-4.202899	1.354024
93	1	0	3.941324	-5.605685	3.073629
94	1	0	4.948682	-4.466434	5.071202
95	1	0	4.769473	-1.985878	5.234368
96	1	0	4.894706	3.386263	-1.739818
97	1	0	6.052584	3.935657	-0.498152
98	1	0	5.557385	5.049814	-1.810605
99	1	0	5.197452	6.263646	1.200274
100	1	0	3.731599	5.731755	2.068452
101	1	0	5.100896	4.617389	1.883784
102	1	0	3.457715	6.892648	-1.283376
103	1	0	2.213072	6.579405	-0.041096
104	1	0	2.182785	5.686235	-1.590356

The total electronic energy was calculated to be –4094.1537701 Hartree..

An imaginary frequency was found at 39.9153 cm^{-1}.

Supplementary Table 29. Optimized structure of **TS-PBR2** (M06-2X/6-31G(d,p))

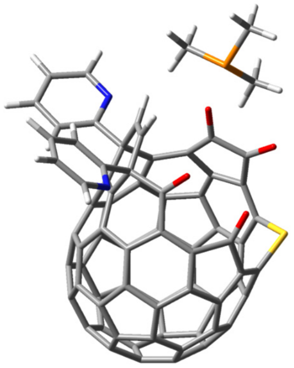

Standard orientation:

Center Number	Atomic Number	Atomic Type	Coordinates (Angstroms) X	Y	Z
1	6	0	-3.622582	-0.955825	0.898122
2	6	0	-3.712952	-1.041155	-0.427131
3	6	0	-2.590331	-1.477776	-1.341525
4	6	0	-1.932819	-0.310452	-2.079072
5	6	0	-2.232647	1.007363	-1.839183
6	6	0	-3.185774	1.727739	-0.929065
7	6	0	-2.438150	2.946949	-0.357815
8	6	0	-1.129395	2.971505	-1.090353
9	6	0	0.130049	3.462349	-0.801530
10	6	0	1.303243	3.068819	-1.578781
11	6	0	2.650754	2.965529	-0.982239
12	6	0	2.807635	3.043087	0.483156
13	6	0	1.665281	3.372269	1.327999
14	6	0	1.461170	2.682716	2.503831
15	6	0	0.122152	2.223105	3.091683
16	6	0	0.375427	0.707959	3.319223
17	6	0	-0.366439	-0.391546	2.921867
18	6	0	-1.789860	-0.226022	2.466980
19	6	0	-2.428337	-1.417809	1.690589
20	6	0	-1.374537	-2.049684	0.811894
21	6	0	-1.456464	-2.108067	-0.555978
22	6	0	-0.333051	-2.581536	-1.330768
23	6	0	0.003961	-1.886896	-2.568194
24	6	0	-0.723527	-0.639098	-2.827685
25	6	0	-0.002977	0.397385	-3.430444
26	6	0	-0.203954	1.739030	-2.970109
27	6	0	-1.238923	1.998487	-2.128646
28	6	0	1.067549	2.362329	-2.766867
29	6	0	2.041051	1.509261	-3.383687
30	6	0	1.380502	0.283589	-3.790443
31	6	0	2.024612	-0.922656	-3.664413
32	6	0	1.315344	-2.033613	-3.054573
33	6	0	2.312699	-2.814794	-2.352118
34	6	0	1.979928	-3.453282	-1.174689
35	6	0	0.643267	-3.309140	-0.661097
36	6	0	0.728611	-3.240555	0.778782
37	6	0	-0.161831	-2.445398	1.487462
38	6	0	0.326179	-1.640993	2.610733
39	6	0	1.677228	-1.782212	2.950454
40	6	0	2.432796	-0.640630	3.381144
41	6	0	1.788937	0.573786	3.461264
42	6	0	2.439476	1.755301	2.948810
43	6	0	3.673578	1.669279	2.353880
44	6	0	3.856670	2.330204	1.085858
45	6	0	4.796983	1.538145	0.337936
46	6	0	4.688623	1.497783	-1.030808
47	6	0	3.631083	2.235669	-1.694087
48	6	0	3.319068	1.479626	-2.892142
49	6	0	4.049151	0.227495	-2.878400
50	6	0	3.411112	-0.955361	-3.249723
51	6	0	3.602630	-2.151548	-2.461586
52	6	0	4.481347	-2.146847	-1.384721
53	6	0	4.133881	-2.842288	-0.157332
54	6	0	2.905273	-3.482060	-0.061408
55	6	0	2.118868	-3.359324	1.143974
56	6	0	2.588597	-2.637356	2.220441
57	6	0	3.881303	-1.983333	2.136732
58	6	0	3.778286	-0.730918	2.848081
59	6	0	4.385311	0.408860	2.332309
60	6	0	5.114984	0.337351	1.090784
61	6	0	5.261345	-0.875962	0.430263
62	6	0	5.165064	-0.920544	-1.016737
63	6	0	4.922142	0.246576	-1.732332
64	6	0	4.628311	-2.066896	0.963789
65	6	0	-2.863420	-2.470081	2.733008
66	6	0	-3.791965	-3.457947	2.399856
67	6	0	-4.131468	-4.398856	3.363695
68	6	0	-3.540317	-4.321973	4.620808
69	6	0	-2.630002	-3.297678	4.854888
70	6	0	-3.183959	-2.457373	-2.363325
71	6	0	-3.294476	-3.820690	-2.080819
72	6	0	-3.920594	-4.641264	-3.010126
73	6	0	-4.415741	-4.077294	-4.181132

74	6	0	-4.263956	-2.706612	-4.363024
75	16	0	0.392446	4.416635	0.674204
76	7	0	-2.294176	-2.389511	3.935944
77	7	0	-3.664429	-1.907298	-3.478725
78	8	0	-0.866849	2.871221	3.256383
79	8	0	-4.361395	1.507080	-0.733863
80	8	0	-2.411169	0.787456	2.657026
81	8	0	-2.447559	2.894220	1.075319
82	15	0	-3.630854	4.272266	-0.199411
83	6	0	-5.042882	3.753019	0.860333
84	6	0	-4.632270	5.161931	-1.502341
85	6	0	-2.832046	5.668456	0.652096
86	1	0	-4.443562	-0.537223	1.472400
87	1	0	-4.621240	-0.717255	-0.930157
88	1	0	-4.236184	-3.472366	1.410259
89	1	0	-4.852437	-5.178504	3.138094
90	1	0	-3.780294	-5.032503	5.403440
91	1	0	-2.147500	-3.194480	5.823721
92	1	0	-2.893459	-4.221465	-1.155288
93	1	0	-4.018787	-5.706163	-2.823736
94	1	0	-4.908737	-4.680243	-4.935176
95	1	0	-4.641495	-2.224079	-5.261524
96	1	0	-4.646116	3.239837	1.737257
97	1	0	-5.673225	3.068220	0.297196
98	1	0	-5.637332	4.625339	1.148956
99	1	0	-5.393846	5.827236	-1.086156
100	1	0	-3.955600	5.730027	-2.149302
101	1	0	-5.119899	4.392693	-2.109059
102	1	0	-3.622549	6.253481	1.130871
103	1	0	-2.296977	6.323563	-0.038642
104	1	0	-2.143970	5.261973	1.393617

The total electronic energy was calculated to be –4094.1412361 Hartree..

An imaginary frequency was found at 80.4508 cm^{-1}.

11.4. Kukhtin-Ramirez Intermediates

By the reaction of PMe_3 with the dicarbonyl moiety on **1'**, the Kukhtin-Ramirez intermediates **INT1** would be formed. The computational studies suggest the four possible conformations, **C1** (1,3-betaine), **C2** (1,5-betaine), **E** (pentavalent phosphorus compound), and **D** (1,5-betaine), in which the interconversion should occur in this order (Supplementary Fig. 43). Among these four conformations, **INT1-C1** is the most probable conformation which proceeds further reaction to give **3a'**.

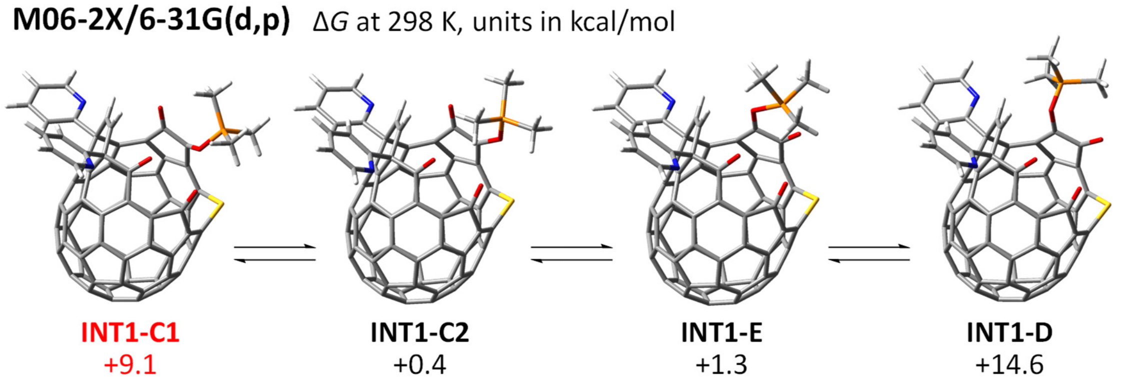

Supplementary Fig. 43. Equilibrium of the Kukhtin-Ramirez intermediates **INT1** (M06-2X/6-31G(d,p)).

Supplementary Table 30. Optimized structure of **INT1-E** (M06-2X/6-31G(d,p))

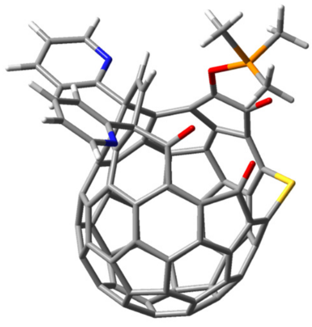

Standard orientation:

Center Number	Atomic Number	Atomic Type	Coordinates (Angstroms) X	Y	Z
1	6	0	-3.721211	-0.602672	0.448912
2	6	0	-3.579335	-0.838856	-0.848310
3	6	0	-2.376799	-1.489105	-1.474375
4	6	0	-1.519756	-0.489136	-2.262047
5	6	0	-1.727028	0.887165	-2.255092
6	6	0	-0.545985	2.950847	-2.007744
7	6	0	0.694019	3.435725	-1.657422
8	6	0	1.915149	2.777775	-2.106499
9	6	0	3.127544	2.715158	-1.276190
10	6	0	3.035051	3.073539	0.151947
11	6	0	1.787698	3.600093	0.688809
12	6	0	1.313766	3.138086	1.903103
13	6	0	-0.138487	2.795606	2.248162
14	6	0	-0.021384	1.408963	2.935740
15	6	0	-0.774773	0.255983	2.741349
16	6	0	-2.164744	0.306555	2.174606
17	6	0	-2.703841	-0.976747	1.487741
18	6	0	-1.554210	-1.757106	0.907804
19	6	0	-1.432650	-2.050909	-0.423993

20	6	0	-0.235890	-2.688365	-0.917516
21	6	0	0.324856	-2.254594	-2.193666
22	6	0	-0.258935	-1.044331	-2.786752
23	6	0	0.625711	-0.209505	-3.477672
24	6	0	0.473800	1.208189	-3.329137
25	6	0	-0.615637	1.708913	-2.714231
26	6	0	1.762411	1.803578	-3.104422
27	6	0	2.733786	0.786561	-3.368385
28	6	0	2.029445	-0.464033	-3.600217
29	6	0	2.554713	-1.645394	-3.141300
30	6	0	1.682718	-2.557680	-2.436853
31	6	0	2.510196	-3.226423	-1.451309
32	6	0	1.967478	-3.599175	-0.241438
33	6	0	0.582223	-3.311150	0.019719
34	6	0	0.461341	-2.976787	1.419168
35	6	0	-0.484600	-2.049051	1.826665
36	6	0	-0.116598	-1.041884	2.824159
37	6	0	1.170499	-1.124971	3.372568
38	6	0	1.915436	0.069786	3.647885
39	6	0	1.340176	1.285795	3.339823
40	6	0	2.138444	2.308026	2.705147
41	6	0	3.451786	2.066381	2.376496
42	6	0	3.898816	2.443986	1.059963
43	6	0	4.885537	1.474821	0.660506
44	6	0	4.993584	1.149584	-0.670637
45	6	0	4.126107	1.784234	-1.642657
46	6	0	3.923915	0.802125	-2.689047
47	6	0	4.540533	-0.455527	-2.298619
48	6	0	3.865884	-1.653180	-2.514777
49	6	0	3.850242	-2.662583	-1.485092
50	6	0	4.561919	-2.471245	-0.304769
51	6	0	3.987858	-2.880732	0.964106
52	6	0	2.718784	-3.439527	0.986626
53	6	0	1.775347	-3.049952	2.007438
54	6	0	2.126761	-2.138186	2.979981
55	6	0	3.453657	-1.552319	2.968015
56	6	0	3.319729	-0.172638	3.373606
57	6	0	4.072602	0.809032	2.739847
58	6	0	4.987111	0.449897	1.684303
59	6	0	5.147918	-0.880378	1.317988
60	6	0	5.272280	-1.228445	-0.085645
61	6	0	5.229526	-0.232020	-1.055277
62	6	0	4.360739	-1.906523	1.972280
63	6	0	-3.376903	-1.853341	2.554964
64	6	0	-4.004460	-3.037197	2.156365
65	6	0	-4.598476	-3.820654	3.134588
66	6	0	-4.542064	-3.400720	4.462496
67	6	0	-3.884021	-2.212014	4.750647
68	6	0	-2.903031	-2.635314	-2.351959
69	6	0	-3.132702	-3.917068	-1.854415
70	6	0	-3.556888	-4.907675	-2.734403
71	6	0	-3.735717	-4.586606	-4.073593
72	6	0	-3.470129	-3.279203	-4.473710
73	16	0	0.767592	4.620843	-0.338536
74	7	0	-3.307346	-1.445263	3.819288
75	7	0	-3.058937	-2.323248	-3.642962
76	8	0	-1.118579	3.416152	1.947179
77	8	0	-2.851325	1.298420	2.241669
78	6	0	-1.888273	3.075464	-1.425408
79	6	0	-2.555068	1.893008	-1.575388
80	8	0	-2.506347	4.017741	-0.773077
81	8	0	-3.794121	1.924933	-0.986430
82	15	0	-4.300318	3.416078	-0.372941
83	6	0	-4.010871	4.161530	1.263082
84	6	0	-4.878067	4.590116	-1.643398
85	6	0	-5.910620	2.593218	0.045974
86	1	0	-4.605525	-0.092543	0.815623
87	1	0	-4.352163	-0.513036	-1.540041
88	1	0	-4.002843	-3.319536	1.105731
89	1	0	-5.097493	-4.747327	2.868865
90	1	0	-4.994318	-3.981903	5.258182
91	1	0	-3.811440	-1.852077	5.773924
92	1	0	-2.987607	-4.128102	-0.800853
93	1	0	-3.746675	-5.914167	-2.374807
94	1	0	-4.069260	-5.323930	-4.794892
95	1	0	-3.594227	-2.986540	-5.513937
96	1	0	-3.510041	3.411574	1.881610
97	1	0	-4.969286	4.430306	1.711793
98	1	0	-3.345812	5.017938	1.170865
99	1	0	-4.322601	5.523386	-1.556643
100	1	0	-4.652698	4.147474	-2.618298
101	1	0	-5.952537	4.761518	-1.567648
102	1	0	-5.752641	1.893275	0.871465
103	1	0	-6.640705	3.341928	0.366180
104	1	0	-6.309532	2.045803	-0.810697

The total electronic energy was calculated to be –4094.1889039 Hartree..

11.5. Hydrolysis of INT1

The process on the hydrolysis of **INT1-C1** was computed (Supplementary Fig. 44). The results suggest the possible formation of protonated compounds **INT2** and pentavalent phosphorus compounds **INT2-^{V}P**. Note that protonated compound **INT2-(2H$_2$O)** should be the active species to form **3a'** via further transformation (vide infra).

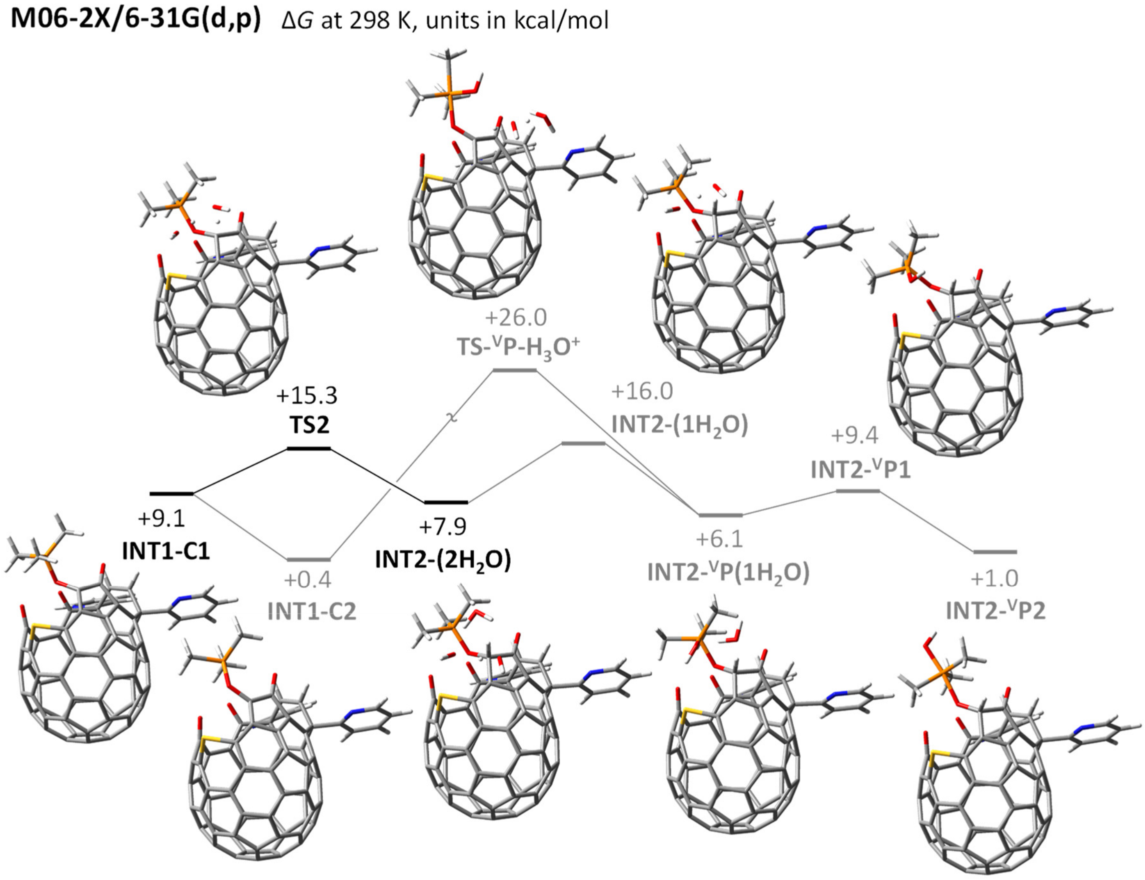

Supplementary Fig. 44. Hydrolysis of **INT1-C1** (M06-2X/6-31G(d,p)).

Supplementary Table 31. Optimized structure of **H_2O** (M06-2X/6-31G(d,p))

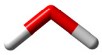

Standard orientation:

Center Number	Atomic Number	Atomic Type	Coordinates (Angstroms) X	Y	Z
1	8	0	0.000000	0.000000	0.117808
2	1	0	0.000000	0.760256	-0.471232
3	1	0	0.000000	-0.760256	-0.471232

The total electronic energy was calculated to be –76.3839203 Hartree..

Supplementary Table 32. Optimized structure of **INT2-(2H_2O)** (M06-2X/6-31G(d,p))

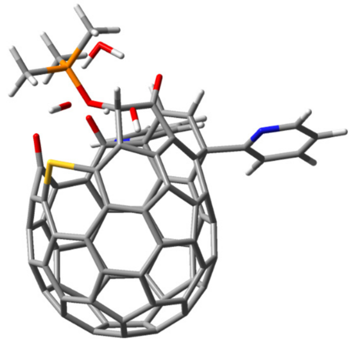

Standard orientation:

Center Number	Atomic Number	Atomic Type	Coordinates (Angstroms) X	Y	Z
1	6	0	-0.938373	3.766961	-0.382571
2	6	0	-0.944938	3.652861	0.941298
3	6	0	0.059589	2.866150	1.740396
4	6	0	-0.533155	1.559409	2.301600
5	6	0	-1.802863	1.079665	2.007774
6	6	0	-3.008260	1.527285	1.206680
7	6	0	-2.865843	-0.880921	1.144641
8	6	0	-2.551000	-2.121498	0.640556
9	6	0	-1.507267	-2.929124	1.263547
10	6	0	-0.658265	-3.865939	0.505841
11	6	0	-0.663327	-3.803379	-0.964637
12	6	0	-1.597215	-2.912405	-1.642176
13	6	0	-1.156878	-2.182652	-2.735184
14	6	0	-1.511211	-0.739701	-3.091159
15	6	0	-0.125724	-0.083941	-3.309672
16	6	0	0.400593	1.095090	-2.797499
17	6	0	-0.481471	2.197008	-2.288530
18	6	0	0.133504	3.204744	-1.273993
19	6	0	1.233231	2.533408	-0.488603
20	6	0	1.230395	2.421452	0.879135
21	6	0	2.250719	1.644699	1.543482
22	6	0	1.876078	0.806932	2.678092
23	6	0	0.435440	0.668543	2.943780
24	6	0	0.006011	-0.587978	3.391721
25	6	0	-1.221029	-1.123179	2.896163
26	6	0	-2.052189	-0.326561	2.172667
27	6	0	-1.022216	-2.484714	2.504187
28	6	0	0.245994	-2.879038	3.041990
29	6	0	0.888819	-1.701867	3.593550
30	6	0	2.244088	-1.533244	3.462064
31	6	0	2.745529	-0.248805	3.012358
32	6	0	3.945185	-0.530407	2.249960
33	6	0	4.271936	0.260330	1.169922
34	6	0	3.398280	1.347163	0.816030
35	6	0	3.386233	1.449255	-0.625013
36	6	0	2.233800	1.857842	-1.278922
37	6	0	1.825240	1.166613	-2.503393
38	6	0	2.675301	0.164764	-2.988115
39	6	0	2.127734	-1.028915	-3.568685
40	6	0	0.756414	-1.158121	-3.626867
41	6	0	0.141306	-2.414534	-3.265106
42	6	0	0.915120	-3.469130	-2.844624
43	6	0	0.499893	-4.173891	-1.657208
44	6	0	1.697817	-4.613272	-0.993054
45	6	0	1.701385	-4.700935	0.378610
46	6	0	0.512514	-4.353201	1.127945
47	6	0	0.984765	-3.836606	2.398600
48	6	0	2.427743	-3.725489	2.368033
49	6	0	3.048058	-2.590165	2.886292
50	6	0	4.129533	-1.970115	2.158068
51	6	0	4.602161	-2.542960	0.983274
52	6	0	4.961738	-1.701021	-0.143263
53	6	0	4.802463	-0.326628	-0.042314
54	6	0	4.250209	0.419185	-1.147644
55	6	0	3.900588	-0.215924	-2.319864
56	6	0	4.074681	-1.651570	-2.439940
57	6	0	2.961183	-2.158628	-3.205920
58	6	0	2.359676	-3.357818	-2.840896
59	6	0	2.853386	-4.094952	-1.703870
60	6	0	3.954637	-3.634775	-0.992997
61	6	0	3.967087	-3.735562	0.455356
62	6	0	2.880785	-4.289906	1.121306
63	6	0	4.578558	-2.380891	-1.365601
64	6	0	0.767072	4.346177	-2.090149
65	6	0	1.139518	5.531864	-1.453098
66	6	0	1.740497	6.527187	-2.210988

67	6	0	1.948536	6.306263	-3.569920
68	6	0	1.545398	5.089510	-4.106939
69	6	0	0.514202	3.767918	2.897250
70	6	0	1.635638	4.592094	2.786163
71	6	0	1.944191	5.437212	3.845598
72	6	0	1.125165	5.434286	4.969143
73	6	0	0.024166	4.583359	4.975442
74	16	0	-3.203428	-2.580454	-0.951141
75	7	0	0.967238	4.123759	-3.388015
76	7	0	-0.282287	3.767432	3.966120
77	8	0	-2.599646	-0.236634	-3.155349
78	8	0	-3.484839	2.618111	1.011931
79	8	0	-1.625833	2.324249	-2.650897
80	6	0	-3.722631	0.251898	0.675521
81	8	0	-3.814676	0.394273	-0.732286
82	15	0	-5.176970	0.683902	-1.508758
83	6	0	-4.747715	2.108773	-2.522637
84	6	0	-6.500659	1.169829	-0.398287
85	6	0	-5.676387	-0.638596	-2.613181
86	8	0	-5.743348	-3.647798	1.058319
87	1	0	-1.734760	4.307656	-0.884692
88	1	0	-1.748250	4.094367	1.520045
89	1	0	0.949494	5.657346	-0.391624
90	1	0	2.040728	7.462817	-1.749945
91	1	0	2.411054	7.057019	-4.200317
92	1	0	1.690471	4.874196	-5.162583
93	1	0	2.252608	4.560880	1.893801
94	1	0	2.812849	6.086205	3.794523
95	1	0	1.330525	6.072863	5.820687
96	1	0	-0.645896	4.553182	5.831428
97	1	0	-4.702882	0.170030	1.168988
98	1	0	-5.605755	2.413662	-3.126917
99	1	0	-4.439722	2.922667	-1.862169
100	1	0	-3.900337	1.839563	-3.157106
101	1	0	-6.811990	0.312969	0.202322
102	1	0	-6.135449	1.969099	0.252833
103	1	0	-7.324528	1.545950	-1.011896
104	1	0	-6.013299	-1.452142	-1.971983
105	1	0	-6.467346	-0.254499	-3.264128
106	1	0	-4.806561	-0.930890	-3.203262
107	1	0	-5.915578	-4.587118	1.187969
108	8	0	-5.403854	-2.494624	3.222909
109	1	0	-5.498528	-3.102877	2.370405
110	1	0	-4.635141	-2.625667	3.782827
111	8	0	-6.218011	-1.467714	1.703146
112	1	0	-5.875126	-1.561566	3.108892
113	1	0	-6.017206	-2.334852	1.343493

The total electronic energy was calculated to be –4323.3872386 Hartree..

Supplementary Table 33. Optimized structure of **INT2-(1H$_2$O)** (M06-2X/6-31G(d,p))

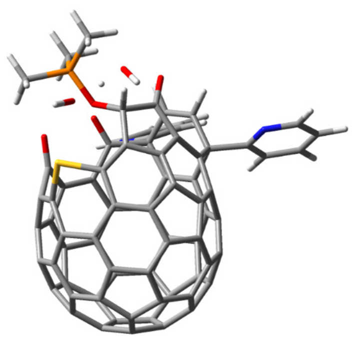

Standard orientation:

Center Number	Atomic Number	Atomic Type	Coordinates (Angstroms) X	Y	Z
1	6	0	3.040824	2.256560	0.553694
2	6	0	2.925890	2.372291	-0.765120
3	6	0	1.619890	2.464794	-1.508472
4	6	0	1.288191	1.167111	-2.269899
5	6	0	2.028433	-0.014544	-2.177872
6	6	0	3.296350	-0.488881	-1.498901
7	6	0	1.739655	-2.328330	-1.658002
8	6	0	0.762877	-3.203713	-1.242283
9	6	0	-0.581130	-3.128283	-1.809503
10	6	0	-1.792523	-3.491607	-1.051855
11	6	0	-1.695859	-3.673754	0.405234
12	6	0	-0.380332	-3.620113	1.049551
13	6	0	-0.262609	-2.953308	2.257661
14	6	0	0.900931	-2.062354	2.719965
15	6	0	0.195725	-0.760494	3.162618
16	6	0	0.462240	0.553542	2.838246
17	6	0	1.808700	0.986357	2.336078
18	6	0	1.882318	2.304180	1.510411
19	6	0	0.569753	2.541149	0.804367
20	6	0	0.451258	2.664871	-0.557348
21	6	0	-0.856211	2.751537	-1.164817
22	6	0	-1.103927	2.042425	-2.416129
23	6	0	-0.045753	1.127430	-2.871682
24	6	0	-0.472829	-0.053940	-3.492894
25	6	0	0.207265	-1.276333	-3.208272
26	6	0	1.378852	-1.243322	-2.519268
27	6	0	-0.752046	-2.300887	-2.931081
28	6	0	-2.023606	-1.786568	-3.345610
29	6	0	-1.854228	-0.389013	-3.693655
30	6	0	-2.831663	0.521225	-3.379145
31	6	0	-2.445028	1.766524	-2.744202
32	6	0	-3.542755	2.130018	-1.870988
33	6	0	-3.287257	2.782670	-0.684710
34	6	0	-1.923288	3.076687	-0.334175
35	6	0	-1.795800	2.926974	1.097056
36	6	0	-0.604225	2.471409	1.640662
37	6	0	-0.644761	1.493689	2.730004
38	6	0	-1.906267	1.124428	3.213431
39	6	0	-2.162507	-0.234459	3.600251
40	6	0	-1.141834	-1.152814	3.476717
41	6	0	-1.418449	-2.456335	2.917823
42	6	0	-2.685338	-2.772945	2.490217
43	6	0	-2.821342	-3.392442	1.195219
44	6	0	-4.067881	-2.934198	0.642772
45	6	0	-4.175596	-2.789390	-0.719760

46	6	0	-3.045098	-3.095746	-1.571005
47	6	0	-3.162903	-2.212176	-2.715339
48	6	0	-4.249271	-1.280828	-2.496219
49	6	0	-4.085180	0.065412	-2.817208
50	6	0	-4.549685	1.081037	-1.902184
51	6	0	-5.224946	0.722469	-0.741534
52	6	0	-4.963578	1.428201	0.499776
53	6	0	-4.015828	2.441115	0.518632
54	6	0	-3.083750	2.537070	1.616375
55	6	0	-3.139653	1.646290	2.666577
56	6	0	-4.135456	0.590814	2.662525
57	6	0	-3.520337	-0.584944	3.230839
58	6	0	-3.773268	-1.834330	2.675500
59	6	0	-4.653671	-1.953271	1.539379
60	6	0	-5.285325	-0.830444	1.019693
61	6	0	-5.411689	-0.679357	-0.418833
62	6	0	-4.901118	-1.655200	-1.266199
63	6	0	-5.017436	0.474260	1.590817
64	6	0	2.093929	3.457636	2.508215
65	6	0	2.488276	4.714391	2.044037
66	6	0	2.635058	5.741703	2.965638
67	6	0	2.384310	5.481482	4.310284
68	6	0	1.991942	4.197407	4.668853
69	6	0	1.751454	3.628760	-2.501440
70	6	0	1.358335	4.925712	-2.165819
71	6	0	1.575426	5.943170	-3.087531
72	6	0	2.178515	5.633805	-4.301540
73	6	0	2.543883	4.311386	-4.533717
74	16	0	1.079959	-4.193131	0.205482
75	7	0	1.845916	3.201075	3.791455
76	7	0	2.340143	3.326122	-3.658447
77	8	0	2.074381	-2.315861	2.697105
78	8	0	4.342333	0.067139	-1.263075
79	8	0	2.812916	0.367230	2.565098
80	6	0	3.124119	-2.002344	-1.190934
81	8	0	3.341114	-2.168972	0.199531
82	15	0	4.633924	-2.841187	0.846877
83	6	0	5.182451	-1.626355	2.045051
84	6	0	5.931682	-3.093791	-0.377876
85	6	0	4.271194	-4.359126	1.730479
86	8	0	3.395320	-4.724015	-1.072825
87	1	0	4.020985	2.135465	1.004693
88	1	0	3.809905	2.339760	-1.391651
89	1	0	2.677956	4.864086	0.985675
90	1	0	2.942588	6.730812	2.641281
91	1	0	2.490174	6.252840	5.064471
92	1	0	1.783855	3.950955	5.707002
93	1	0	0.886217	5.124938	-1.209056
94	1	0	1.275560	6.961585	-2.861135
95	1	0	2.362797	6.394092	-5.051909
96	1	0	3.023326	4.026416	-5.467267
97	1	0	3.839198	-2.566915	-1.806135
98	1	0	6.080578	-1.973965	2.571748
99	1	0	5.396876	-0.690690	1.510017
100	1	0	4.370901	-1.439620	2.744436
101	1	0	5.622074	-3.864638	-1.086573
102	1	0	6.101327	-2.144709	-0.899279
103	1	0	6.838721	-3.379674	0.154641
104	1	0	3.817438	-5.089350	0.890916
105	1	0	5.320985	-4.747269	2.202725
106	1	0	3.495231	-4.047009	2.565658
107	1	0	3.202223	-5.689319	-1.168318
108	8	0	4.643319	-3.937687	-3.057037
109	1	0	4.063551	-4.373376	-2.288117
110	1	0	4.152933	-4.051871	-3.875876

The total electronic energy was calculated to be –4246.9711455 Hartree..

Supplementary Table 34. Optimized structure of **INT2-^{V}P(1H_2O)** (M06-2X/6-31G(d,p))

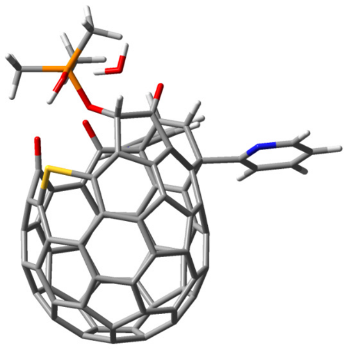

Standard orientation:

Center Number	Atomic Number	Atomic Type	Coordinates (Angstroms) X	Y	Z
1	6	0	2.691699	2.648306	0.562975
2	6	0	2.533960	2.789540	-0.748911
3	6	0	1.211382	2.731046	-1.467189
4	6	0	1.051730	1.429923	-2.273921
5	6	0	1.957891	0.379143	-2.250535
6	6	0	3.322095	0.087103	-1.663909
7	6	0	2.034867	-1.962014	-1.775795
8	6	0	1.196354	-2.974073	-1.364850
9	6	0	-0.158026	-3.097005	-1.904773
10	6	0	-1.284769	-3.656883	-1.135902
11	6	0	-1.133071	-3.863173	0.313931
12	6	0	0.159357	-3.629889	0.943456
13	6	0	0.213519	-2.982583	2.164274
14	6	0	1.243583	-1.943256	2.608705
15	6	0	0.363181	-0.786200	3.136273
16	6	0	0.425993	0.571487	2.854880
17	6	0	1.690341	1.213562	2.361905
18	6	0	1.557561	2.532757	1.542835
19	6	0	0.208507	2.592086	0.867426
20	6	0	0.045264	2.730081	-0.489345
21	6	0	-1.273354	2.646061	-1.073351

22	6	0	-1.444056	1.946360	-2.343479
23	6	0	-0.275324	1.210628	-2.847873
24	6	0	-0.538365	-0.001573	-3.500303
25	6	0	0.319233	-1.117773	-3.263084
26	6	0	1.490490	-0.932221	-2.600613
27	6	0	-0.474273	-2.279562	-2.999717
28	6	0	-1.815362	-1.946936	-3.377099
29	6	0	-1.859681	-0.530648	-3.686242
30	6	0	-2.951632	0.218225	-3.325912
31	6	0	-2.737118	1.488083	-2.658510
32	6	0	-3.857836	1.662282	-1.756870
33	6	0	-3.673681	2.310832	-0.555242
34	6	0	-2.359741	2.789194	-0.217414
35	6	0	-2.183853	2.623860	1.206463
36	6	0	-0.928433	2.335086	1.719774
37	6	0	-0.808876	1.338130	2.785509
38	6	0	-1.992907	0.771938	3.274375
39	6	0	-2.038455	-0.619541	3.624847
40	6	0	-0.896110	-1.372805	3.455998
41	6	0	-0.988811	-2.685469	2.859058
42	6	0	-2.202736	-3.174485	2.440405
43	6	0	-2.271386	-3.771453	1.130067
44	6	0	-3.583349	-3.488212	0.611788
45	6	0	-3.739762	-3.324823	-0.743633
46	6	0	-2.593302	-3.437420	-1.621885
47	6	0	-2.865191	-2.551439	-2.737734
48	6	0	-4.071510	-1.796203	-2.472953
49	6	0	-4.112430	-0.432338	-2.756213
50	6	0	-4.700924	0.478537	-1.803166
51	6	0	-5.292924	-0.006730	-0.643415
52	6	0	-5.111665	0.695721	0.614247
53	6	0	-4.320639	1.835203	0.649029
54	6	0	-3.391748	2.037497	1.734832
55	6	0	-3.298634	1.119532	2.759039
56	6	0	-4.128584	-0.069999	2.737274
57	6	0	-3.336052	-1.157116	3.261312
58	6	0	-3.413809	-2.414323	2.672601
59	6	0	-4.289496	-2.630290	1.547383
60	6	0	-5.089991	-1.599388	1.071402
61	6	0	-5.265283	-1.429557	-0.359306
62	6	0	-4.635390	-2.295760	-1.243994
63	6	0	-5.004744	-0.285283	1.676167
64	6	0	1.629851	3.698432	2.547488
65	6	0	1.935866	4.987925	2.107923
66	6	0	1.954800	6.016085	3.040783
67	6	0	1.668660	5.724819	4.371196
68	6	0	1.370063	4.408924	4.705621
69	6	0	1.145964	3.946006	-2.402988
70	6	0	0.684615	5.180911	-1.940942
71	6	0	0.712613	6.265732	-2.807259
72	6	0	1.201703	6.083898	-4.096883
73	6	0	1.646402	4.815936	-4.456085
74	16	0	1.655918	-3.952159	0.050322
75	7	0	1.346730	3.412669	3.817676
76	7	0	1.624330	3.765182	-3.633244
77	8	0	2.435069	-2.002894	2.504544
78	8	0	4.291528	0.787281	-1.535851
79	8	0	2.777025	0.751458	2.600533
80	6	0	3.399511	-1.449945	-1.371893
81	8	0	3.857028	-1.595739	-0.068467
82	15	0	5.046103	-2.695259	0.394511
83	6	0	5.485806	-1.430269	1.712373
84	6	0	6.685871	-2.581196	-0.438458
85	6	0	4.790065	-4.055141	1.598964
86	8	0	4.455784	-3.809227	-0.869902
87	1	0	3.688781	2.631275	0.992414
88	1	0	3.401763	2.887540	-1.390940
89	1	0	2.160839	5.163706	1.060869
90	1	0	2.193266	7.030005	2.735128
91	1	0	1.676254	6.495496	5.133441
92	1	0	1.139556	4.136594	5.732513
93	1	0	0.306089	5.278129	-0.927949
94	1	0	0.354917	7.237463	-2.481588
95	1	0	1.239167	6.900834	-4.808422
96	1	0	2.038930	4.630200	-5.453068
97	1	0	4.120922	-1.837348	-2.103386
98	1	0	6.307725	-1.801157	2.335194
99	1	0	5.797785	-0.497883	1.232258
100	1	0	4.624534	-1.206032	2.343800
101	1	0	6.954089	-3.505675	-0.949208
102	1	0	6.608811	-1.766924	-1.170143
103	1	0	7.461575	-2.303881	0.275664
104	1	0	5.683746	-4.113532	2.225872
105	1	0	3.951758	-3.760551	2.234273
106	1	0	4.593363	-5.005145	1.110001
107	1	0	3.775778	-4.421960	-0.571521
108	8	0	4.237014	-3.224136	-2.468244
109	1	0	4.322872	-4.067361	-2.057191
110	1	0	4.373145	-3.406629	-3.382316

The total electronic energy was calculated to be –4246.9867811 Hartree..

Supplementary Table 35. Optimized structure of **INT2-^{V}P1** (M06-2X/6-31G(d,p))

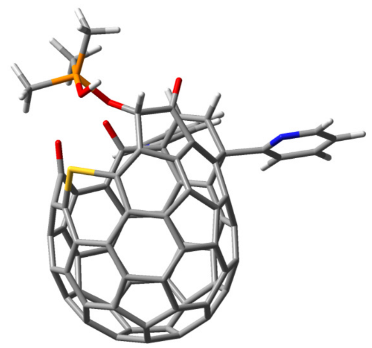

Standard orientation:

Center Number	Atomic Number	Atomic Type	X	Y	Z
1	6	0	3.378574	1.901036	0.391041
2	6	0	3.128204	2.246006	-0.866251
3	6	0	1.754662	2.524583	-1.418936
4	6	0	1.204530	1.380221	-2.287053
5	6	0	1.817034	0.138770	-2.448460
6	6	0	3.081537	-0.532886	-1.991583
7	6	0	1.333811	-2.201838	-2.238969
8	6	0	0.319209	-3.063019	-1.891433
9	6	0	-1.060330	-2.789511	-2.276877
10	6	0	-2.210340	-3.181901	-1.443180
11	6	0	-1.975553	-3.625662	-0.057302
12	6	0	-0.614671	-3.805778	0.429186
13	6	0	-0.267933	-3.352414	1.685342
14	6	0	1.031684	-2.681353	2.119568
15	6	0	0.535760	-1.385249	2.805806
16	6	0	0.897000	-0.053071	2.627472
17	6	0	2.155345	0.316104	1.892260
18	6	0	2.325247	1.788867	1.456779
19	6	0	0.981972	2.275936	0.983017
20	6	0	0.725093	2.654202	-0.305677
21	6	0	-0.626405	2.948016	-0.713400
22	6	0	-1.087971	2.469337	-2.010500
23	6	0	-0.186106	1.543980	-2.719668
24	6	0	-0.797710	0.513888	-3.447828
25	6	0	-0.221118	-0.792184	-3.424254
26	6	0	1.010720	-0.969579	-2.877637
27	6	0	-1.251954	-1.764433	-3.212126
28	6	0	-2.497132	-1.081159	-3.390346
29	6	0	-2.218899	0.334915	-3.535526
30	6	0	-3.054429	1.259552	-2.961157
31	6	0	-2.477112	2.352568	-2.200882
32	6	0	-3.427007	2.656329	-1.150033
33	6	0	-2.969236	3.076115	0.080259
34	6	0	-1.552877	3.201608	0.291264
35	6	0	-1.276198	2.792544	1.649452
36	6	0	-0.086728	2.145560	1.941232
37	6	0	-0.097793	0.991041	2.843777
38	6	0	-1.322629	0.660453	3.439401
39	6	0	-1.677263	-0.714782	3.643769
40	6	0	-0.785427	-1.690428	3.253170
41	6	0	-1.276459	-2.857359	2.554837
42	6	0	-2.609478	-2.978227	2.248511
43	6	0	-2.962217	-3.363921	0.904722
44	6	0	-4.202941	-2.704057	0.595110
45	6	0	-4.446868	-2.323565	-0.702554
46	6	0	-3.454925	-2.581470	-1.727169
47	6	0	-3.596902	-1.507136	-2.692725
48	6	0	-4.548575	-0.536016	-2.196810
49	6	0	-4.278857	0.824747	-2.323062
50	6	0	-4.530521	1.711287	-1.211794
51	6	0	-5.103291	1.222609	-0.044547
52	6	0	-4.628623	1.685361	1.247292
53	6	0	-3.584566	2.595931	1.300114
54	6	0	-2.525819	2.423300	2.265111
55	6	0	-2.549719	1.375065	3.159378
56	6	0	-3.646776	0.427214	3.121810
57	6	0	-3.100667	-0.876966	3.412212
58	6	0	-3.558636	-1.989209	2.717069
59	6	0	-4.571113	-1.838376	1.701826
60	6	0	-5.128461	-0.592192	1.447104
61	6	0	-5.398932	-0.192279	0.078559
62	6	0	-5.098723	-1.053557	-0.969224
63	6	0	-4.656138	0.569685	2.172719
64	6	0	2.758039	2.607221	2.679253
65	6	0	2.870895	3.994208	2.572075
66	6	0	3.273382	4.701444	3.696523
67	6	0	3.544990	4.002571	4.871133
68	6	0	3.403535	2.620308	4.868096
69	6	0	1.838426	3.852187	-2.188738
70	6	0	1.593088	5.084076	-1.587791
71	6	0	1.636229	6.227924	-2.380446
72	6	0	1.919035	6.096856	-3.733399
73	6	0	2.138418	4.817028	-4.238778
74	16	0	0.624954	-4.363531	-0.717444
75	7	0	3.017623	1.924909	3.792538
76	7	0	2.096303	3.714764	-3.491648
77	8	0	2.149348	-3.097871	1.962934
78	8	0	4.201646	-0.135386	-1.791451
79	8	0	2.981711	-0.511912	1.586435
80	6	0	2.783954	-2.071919	-1.871533
81	8	0	3.103484	-2.472671	-0.544740
82	15	0	4.475859	-3.140343	-0.083515
83	6	0	5.128842	-2.290750	1.345738
84	6	0	5.628999	-2.958658	-1.557543
85	6	0	3.988948	-4.739923	0.610599
86	8	0	4.378737	-5.684623	-2.035863
87	1	0	4.397532	1.692614	0.703102
88	1	0	3.943940	2.315046	-1.578509
89	1	0	2.644383	4.483777	1.627621
90	1	0	3.373209	5.781452	3.661308
91	1	0	3.859005	4.516875	5.772175
92	1	0	3.605631	2.039580	5.764179
93	1	0	1.378639	5.149034	-0.526299
94	1	0	1.453625	7.204577	-1.944075
95	1	0	1.967792	6.959719	-4.387325
96	1	0	2.357868	4.671188	-5.293608
97	1	0	3.415144	-2.597263	-2.599251
98	1	0	6.062097	-2.784308	1.631698
99	1	0	5.303197	-1.244417	1.088889
100	1	0	4.390043	-2.335754	2.147009

101	1	0	5.357289	-3.610275	-2.390879
102	1	0	5.705624	-1.921436	-1.896367
103	1	0	6.593154	-3.287206	-1.160093
104	1	0	4.875872	-5.257930	0.773802
105	1	0	3.214270	-4.762916	1.379821
106	1	0	3.627420	-5.251519	-0.284382
107	1	0	5.109421	-6.316180	-2.126074

The total electronic energy was calculated to be –4170.5791824 Hartree..

Supplementary Table 36. Optimized structure of **INT2-^{V}P2** (M06-2X/6-31G(d,p))

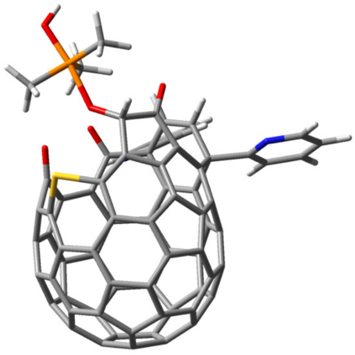

Standard orientation:

Center Number	Atomic Number	Atomic Type	Coordinates (Angstroms) X	Y	Z
1	6	0	3.527397	1.556491	0.404907
2	6	0	3.288490	1.988047	-0.827634
3	6	0	1.935571	2.410713	-1.338169
4	6	0	1.276796	1.364901	-2.253663
5	6	0	1.778632	0.086579	-2.491287
6	6	0	2.987940	-0.712876	-2.094705
7	6	0	1.100650	-2.212719	-2.394296
8	6	0	0.021606	-3.002509	-2.072228
9	6	0	-1.335205	-2.593315	-2.415926
10	6	0	-2.501831	-2.931204	-1.581444
11	6	0	-2.284943	-3.466611	-0.225344
12	6	0	-0.937230	-3.786703	0.224459
13	6	0	-0.534211	-3.431994	1.495463
14	6	0	0.824313	-2.897203	1.938507
15	6	0	0.451178	-1.602384	2.700715
16	6	0	0.922054	-0.297838	2.584819
17	6	0	2.196108	0.002544	1.845349
18	6	0	2.484494	1.476863	1.483743
19	6	0	1.180706	2.100205	1.062251
20	6	0	0.937683	2.567118	-0.199786
21	6	0	-0.389793	2.995339	-0.565221
22	6	0	-0.909985	2.627323	-1.876178
23	6	0	-0.101144	1.668352	-2.649945
24	6	0	-0.809303	0.733950	-3.418561
25	6	0	-0.345975	-0.615523	-3.474144
26	6	0	0.874317	-0.925227	-2.961643
27	6	0	-1.452707	-1.507220	-3.292729
28	6	0	-2.637577	-0.712725	-3.410856
29	6	0	-2.241738	0.680532	-3.487669
30	6	0	-2.986583	1.640390	-2.849826
31	6	0	-2.306730	2.638580	-2.045205
32	6	0	-3.211322	2.964864	-0.961644
33	6	0	-2.700942	3.278172	0.279623
34	6	0	-1.276027	3.272196	0.469163
35	6	0	-1.014861	2.769254	1.798600
36	6	0	0.119317	2.009503	2.033086
37	6	0	0.023354	0.813472	2.874468
38	6	0	-1.216125	0.556114	3.475917
39	6	0	-1.683702	-0.793221	3.615440
40	6	0	-0.884351	-1.818405	3.157333
41	6	0	-1.483619	-2.900734	2.409035
42	6	0	-2.826547	-2.892098	2.122933
43	6	0	-3.231088	-3.174286	0.768064
44	6	0	-4.415497	-2.396441	0.517445
45	6	0	-4.645560	-1.927928	-0.753685
46	6	0	-3.694771	-2.213537	-1.809493
47	6	0	-3.759065	-1.081003	-2.714967
48	6	0	-4.616830	-0.060898	-2.150773
49	6	0	-4.233909	1.277032	-2.211395
50	6	0	-4.392257	2.121008	-1.050863
51	6	0	-4.987012	1.620762	0.100379
52	6	0	-4.455207	1.972106	1.405002
53	6	0	-3.336581	2.787160	1.484664
54	6	0	-2.282029	2.474455	2.418528
55	6	0	-2.381832	1.385702	3.257418
56	6	0	-3.556211	0.537213	3.192084
57	6	0	-3.119076	-0.822061	3.403610
58	6	0	-3.680690	-1.852936	2.660693
59	6	0	-4.691762	-1.563142	1.674624
60	6	0	-5.144507	-0.262533	1.495929
61	6	0	-5.400424	0.231333	0.155554
62	6	0	-5.190603	-0.594997	-0.941227
63	6	0	-4.563899	0.814890	2.271570
64	6	0	3.003882	2.189270	2.738401
65	6	0	3.233056	3.565517	2.701250
66	6	0	3.711296	4.175159	3.852852
67	6	0	3.939917	3.394095	4.984034
68	6	0	3.680997	2.030851	4.911944
69	6	0	2.120726	3.765734	-2.039440
70	6	0	1.990471	4.980063	-1.370621
71	6	0	2.119133	6.156897	-2.103464
72	6	0	2.369335	6.074897	-3.466664
73	6	0	2.471079	4.809955	-4.042046
74	16	0	0.232887	-4.385049	-0.973591
75	7	0	3.221015	1.428976	3.809449
76	7	0	2.346304	3.676843	-3.352517
77	8	0	1.899875	-3.397621	1.738647
78	8	0	4.140767	-0.422617	-1.896103
79	8	0	2.944109	-0.874751	1.480836
80	6	0	2.561953	-2.225501	-2.048995
81	8	0	2.866045	-2.722113	-0.751310
82	15	0	4.183223	-3.526941	-0.352288
83	6	0	4.927751	-2.813087	1.106153

84	6	0	5.529644	-2.977601	-1.600581
85	6	0	3.355709	-4.818175	0.289725
86	8	0	4.266888	-4.803987	-1.534393
87	1	0	4.529432	1.246419	0.685758
88	1	0	4.096334	2.025934	-1.551136
89	1	0	3.034962	4.122221	1.788113
90	1	0	3.902377	5.243218	3.871856
91	1	0	4.310194	3.831175	5.904277
92	1	0	3.846260	1.388091	5.772526
93	1	0	1.798342	5.006084	-0.303202
94	1	0	2.027120	7.120763	-1.613455
95	1	0	2.481701	6.964267	-4.075701
96	1	0	2.661390	4.702677	-5.107112
97	1	0	3.134992	-2.762620	-2.815213
98	1	0	5.819681	-3.398239	1.347802
99	1	0	5.186872	-1.772985	0.900661
100	1	0	4.199945	-2.838309	1.918278
101	1	0	5.190815	-3.558496	-2.461183
102	1	0	5.689392	-1.933935	-1.886487
103	1	0	6.468116	-3.407134	-1.239644
104	1	0	4.197549	-5.417184	0.408441
105	1	0	2.593555	-4.816755	1.071695
106	1	0	2.938420	-5.248874	-0.623398
107	1	0	4.939574	-5.489248	-1.671548

The total electronic energy was calculated to be –4170.5936634 Hartree..

Supplementary Table 37. Optimized structure of **TS2** (M06-2X/6-31G(d,p))

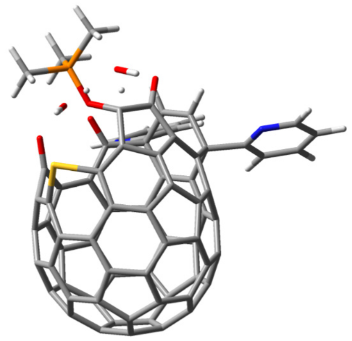

Standard orientation:

Center Number	Atomic Number	Atomic Type	Coordinates (Angstroms) X	Y	Z
1	6	0	-3.197877	2.092295	-0.495931
2	6	0	-3.076957	2.188063	0.823879
3	6	0	-1.771713	2.345417	1.557447
4	6	0	-1.353914	1.056833	2.291520
5	6	0	-2.020483	-0.157572	2.193771
6	6	0	-3.258905	-0.700096	1.510342
7	6	0	-1.592687	-2.441691	1.629312
8	6	0	-0.569191	-3.251327	1.194338
9	6	0	0.772513	-3.109600	1.750664
10	6	0	1.995571	-3.384251	0.975267
11	6	0	1.893443	-3.547103	-0.483943
12	6	0	0.579153	-3.563339	-1.114354
13	6	0	0.398376	-2.883754	-2.308752
14	6	0	-0.820055	-2.067118	-2.736430
15	6	0	-0.199577	-0.717706	-3.173963
16	6	0	-0.543010	0.582903	-2.827671
17	6	0	-1.907959	0.926484	-2.307887
18	6	0	-2.053238	2.224632	-1.461295
19	6	0	-0.750867	2.528140	-0.762267
20	6	0	-0.626776	2.634085	0.600314
21	6	0	0.678742	2.790086	1.197849
22	6	0	0.981111	2.076457	2.434319
23	6	0	-0.014682	1.090307	2.882745
24	6	0	0.490365	-0.072038	3.480898
25	6	0	-0.115737	-1.329625	3.182973
26	6	0	-1.292552	-1.358272	2.502414
27	6	0	0.902269	-2.289076	2.882860
28	6	0	2.143602	-1.703197	3.293856
29	6	0	1.891547	-0.324152	3.665172
30	6	0	2.808081	0.649342	3.357352
31	6	0	2.339657	1.878506	2.746453
32	6	0	3.404750	2.322998	1.870251
33	6	0	3.098565	2.978153	0.697419
34	6	0	1.715901	3.194208	0.363826
35	6	0	1.584002	3.060983	-1.068663
36	6	0	0.416940	2.543396	-1.609691
37	6	0	0.505924	1.587019	-2.714893
38	6	0	1.782645	1.302221	-3.215030
39	6	0	2.116718	-0.032659	-3.625212
40	6	0	1.154987	-1.013238	-3.506315
41	6	0	1.515333	-2.306049	-2.970969
42	6	0	2.803774	-2.551991	-2.562071
43	6	0	2.991167	-3.183346	-1.279366
44	6	0	4.213188	-2.658626	-0.731179
45	6	0	4.326564	-2.529647	0.632519
46	6	0	3.226316	-2.919020	1.489067
47	6	0	3.300743	-2.047771	2.646582
48	6	0	4.325219	-1.047684	2.432707
49	6	0	4.081846	0.280628	2.777237
50	6	0	4.474185	1.337367	1.875163
51	6	0	5.158736	1.039600	0.702817
52	6	0	4.842521	1.747917	-0.524186
53	6	0	3.834833	2.701353	-0.517812
54	6	0	2.888104	2.758352	-1.605669
55	6	0	2.987614	1.889417	-2.670914
56	6	0	4.045511	0.896353	-2.692976
57	6	0	3.496926	-0.305580	-3.274462
58	6	0	3.830671	-1.545922	-2.742226
59	6	0	4.728486	-1.629222	-1.616646
60	6	0	5.295964	-0.478360	-1.084348
61	6	0	5.427612	-0.342860	0.355217
62	6	0	4.986584	-1.361685	1.190878
63	6	0	4.943535	0.816629	-1.631280
64	6	0	-2.342887	3.378177	-2.438958
65	6	0	-2.802779	4.603275	-1.951066
66	6	0	-3.020263	5.633593	-2.855160
67	6	0	-2.772232	5.407669	-4.206538
68	6	0	-2.311632	4.153555	-4.589211
69	6	0	-1.964732	3.481082	2.572916

70	6	0	-1.650017	4.804760	2.259775
71	6	0	-1.921265	5.790663	3.201185
72	6	0	-2.498280	5.424284	4.411962
73	6	0	-2.784231	4.078631	4.621163
74	16	0	-0.830631	-4.239026	-0.263709
75	7	0	-2.097479	3.154698	-3.728851
76	7	0	-2.528332	3.123227	3.726681
77	8	0	-1.976493	-2.389329	-2.709561
78	8	0	-4.335850	-0.205383	1.285831
79	8	0	-2.875145	0.242026	-2.538398
80	6	0	-2.996839	-2.196274	1.175917
81	8	0	-3.214387	-2.345334	-0.217322
82	15	0	-4.468829	-3.104095	-0.844457
83	6	0	-5.105307	-1.894040	-2.016262
84	6	0	-5.746990	-3.423959	0.374318
85	6	0	-4.035235	-4.579564	-1.768123
86	8	0	-2.616174	-5.822956	1.499459
87	1	0	-4.173731	1.923182	-0.940701
88	1	0	-3.951368	2.091573	1.457347
89	1	0	-2.986949	4.726537	-0.888300
90	1	0	-3.379984	6.598497	-2.512137
91	1	0	-2.931578	6.182427	-4.947698
92	1	0	-2.103223	3.934417	-5.633403
93	1	0	-1.195508	5.048970	1.304936
94	1	0	-1.682978	6.828919	2.992355
95	1	0	-2.722369	6.158703	5.177138
96	1	0	-3.240780	3.748950	5.551475
97	1	0	-3.751102	-2.951533	1.911106
98	1	0	-5.979289	-2.295423	-2.535293
99	1	0	-5.372418	-0.988301	-1.466908
100	1	0	-4.311197	-1.646815	-2.724320
101	1	0	-5.405059	-4.186843	1.076430
102	1	0	-5.961467	-2.494122	0.908706
103	1	0	-6.641496	-3.750922	-0.163917
104	1	0	-3.739234	-5.320246	-1.026319
105	1	0	-4.911341	-4.884752	-2.347930
106	1	0	-3.207350	-4.329255	-2.433090
107	1	0	-2.545956	-6.533154	2.147134
108	8	0	-3.780859	-4.032132	2.736346
109	1	0	-3.175929	-4.954031	2.147566
110	1	0	-3.103766	-4.012060	3.418686

The total electronic energy was calculated to be –4246.9679852 Hartree..

An imaginary frequency was found at 1520.2560 cm⁻¹.

Supplementary Table 38. Optimized structure of **TS-^{V}P-H_3O^+** (M06-2X/6-31G(d,p))

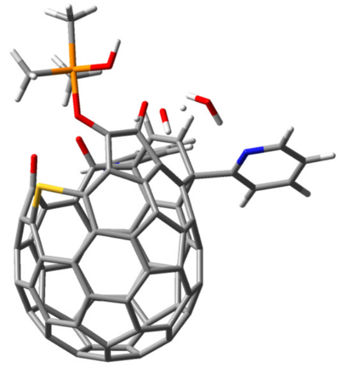

Standard orientation:

Center Number	Atomic Number	Atomic Type	Coordinates (Angstroms) X	Y	Z
1	6	0	-2.933311	1.974027	-0.753085
2	6	0	-2.908812	2.006146	0.575436
3	6	0	-1.671926	2.175101	1.415882
4	6	0	-1.325456	0.878956	2.175769
5	6	0	-2.012231	-0.321766	2.031519
6	6	0	-3.141198	-0.882452	1.228038
7	6	0	-1.539537	-2.541786	1.445229
8	6	0	-0.493299	-3.355390	1.062935
9	6	0	0.814793	-3.259801	1.705959
10	6	0	2.076738	-3.517845	0.993284
11	6	0	2.067634	-3.626529	-0.477778
12	6	0	0.805048	-3.592198	-1.204958
13	6	0	0.705550	-2.872251	-2.383219
14	6	0	-0.472040	-2.001224	-2.823976
15	6	0	0.209567	-0.685026	-3.266756
16	6	0	-0.132890	0.621429	-2.944845
17	6	0	-1.533261	1.024292	-2.604538
18	6	0	-1.731903	2.214976	-1.623566
19	6	0	-0.479077	2.453331	-0.814676
20	6	0	-0.455879	2.490647	0.558647
21	6	0	0.802233	2.624722	1.256144
22	6	0	1.008985	1.886920	2.499178
23	6	0	-0.019750	0.903930	2.862733
24	6	0	0.447334	-0.256635	3.493503
25	6	0	-0.143502	-1.502521	3.118213
26	6	0	-1.270632	-1.507896	2.382476
27	6	0	0.884369	-2.465763	2.861772
28	6	0	2.098886	-1.901783	3.366926
29	6	0	1.830194	-0.525489	3.755823
30	6	0	2.770963	0.446067	3.528732
31	6	0	2.346036	1.679823	2.906157
32	6	0	3.472120	2.138540	2.116562
33	6	0	3.254053	2.825945	0.943998
34	6	0	1.899625	3.055646	0.516280
35	6	0	1.876006	2.978896	-0.924461
36	6	0	0.748126	2.502795	-1.573804
37	6	0	0.917878	1.595312	-2.709659
38	6	0	2.224817	1.296171	-3.112242
39	6	0	2.564180	-0.035828	-3.526252
40	6	0	1.578690	-1.001810	-3.495573
41	6	0	1.876245	-2.308154	-2.957420
42	6	0	3.127809	-2.586810	-2.463016
43	6	0	3.219218	-3.253773	-1.188468
44	6	0	4.411016	-2.765169	-0.547159
45	6	0	4.434183	-2.673010	0.823597
46	6	0	3.277640	-3.075644	1.597014
47	6	0	3.289228	-2.240708	2.781812
48	6	0	4.337121	-1.242482	2.659472
49	6	0	4.079747	0.077068	3.019160

50	6	0	4.537393	1.149942	2.172130
51	6	0	5.303515	0.877544	1.042975
52	6	0	5.078151	1.620591	-0.183373
53	6	0	4.076965	2.581142	-0.221498
54	6	0	3.215098	2.681595	-1.373927
55	6	0	3.390461	1.846350	-2.457324
56	6	0	4.432508	0.838346	-2.427247
57	6	0	3.909095	-0.340662	-3.076918
58	6	0	4.183547	-1.597653	-2.550561
59	6	0	5.000384	-1.723045	-1.369102
60	6	0	5.547238	-0.594871	-0.769581
61	6	0	5.582246	-0.497234	0.678152
62	6	0	5.071988	-1.530707	1.456268
63	6	0	5.248887	0.718225	-1.305286
64	6	0	-1.959631	3.469613	-2.489241
65	6	0	-2.573008	4.600282	-1.946966
66	6	0	-2.706874	5.726181	-2.748802
67	6	0	-2.226249	5.685835	-4.054084
68	6	0	-1.625033	4.512405	-4.496004
69	6	0	-1.972814	3.309759	2.408723
70	6	0	-1.582214	4.627757	2.161875
71	6	0	-1.951993	5.611841	3.071367
72	6	0	-2.700080	5.251379	4.186009
73	6	0	-3.050599	3.912602	4.333098
74	16	0	-0.651880	-4.261174	-0.459104
75	7	0	-1.488798	3.425071	-3.734867
76	7	0	-2.701690	2.959298	3.469492
77	8	0	-1.644160	-2.246663	-2.729221
78	8	0	-4.341109	-0.272221	1.005721
79	8	0	-2.487911	0.480160	-3.104334
80	6	0	-2.828601	-2.162538	0.825402
81	8	0	-3.203755	-2.863112	-0.231568
82	15	0	-4.840225	-2.796779	-0.953832
83	6	0	-4.681428	-0.992466	-0.977087
84	6	0	-6.415014	-2.834504	-1.999356
85	6	0	-4.023124	-3.753769	-2.280422
86	1	0	-3.868436	1.801936	-1.278298
87	1	0	-3.822184	1.857873	1.139071
88	1	0	-2.933226	4.582299	-0.923434
89	1	0	-3.180984	6.622693	-2.361529
90	1	0	-2.312154	6.540409	-4.715621
91	1	0	-1.233198	4.437065	-5.507423
92	1	0	-0.996223	4.869348	1.281137
93	1	0	-1.657031	6.644279	2.911482
94	1	0	-3.007849	5.983881	4.923580
95	1	0	-3.640975	3.587660	5.186998
96	1	0	-4.468847	-0.682607	-2.002927
97	1	0	-5.627438	-0.549096	-0.658260
98	1	0	-3.874950	-0.634893	-0.344077
99	1	0	-6.737096	-3.862328	-2.204569
100	1	0	-7.242826	-2.294516	-1.526236
101	1	0	-6.221798	-2.355134	-2.963456
102	1	0	-4.655590	-3.839666	-3.163026
103	1	0	-3.082435	-3.248216	-2.510114
104	1	0	-3.779786	-4.742332	-1.886144
105	8	0	-5.643394	-3.483377	0.343805
106	1	0	-6.561827	-3.664825	0.105694
107	1	0	-5.172328	-0.746966	1.825045
108	8	0	-5.149220	-1.276221	2.951432
109	1	0	-4.922318	-2.498592	2.821717
110	1	0	-4.481624	-0.795645	3.406362
111	8	0	-4.459021	-3.532798	2.279795
112	1	0	-3.986264	-4.087489	2.873444
113	1	0	-3.570051	-3.001005	1.580204

The total electronic energy was calculated to be –4323.356009 Hartree..

An imaginary frequency was found at 4114.2953 cm^{-1}.

11.6. P–C Bond Formation from INT1 via a Concerted Pathway

The concerted pathway for the formation of **3a'** from **INT1-C1** was examined. The required energy was calculated to be ca. 55 kcal/mol (Supplementary Fig. 45). Thus, this pathway is conclusively excluded.

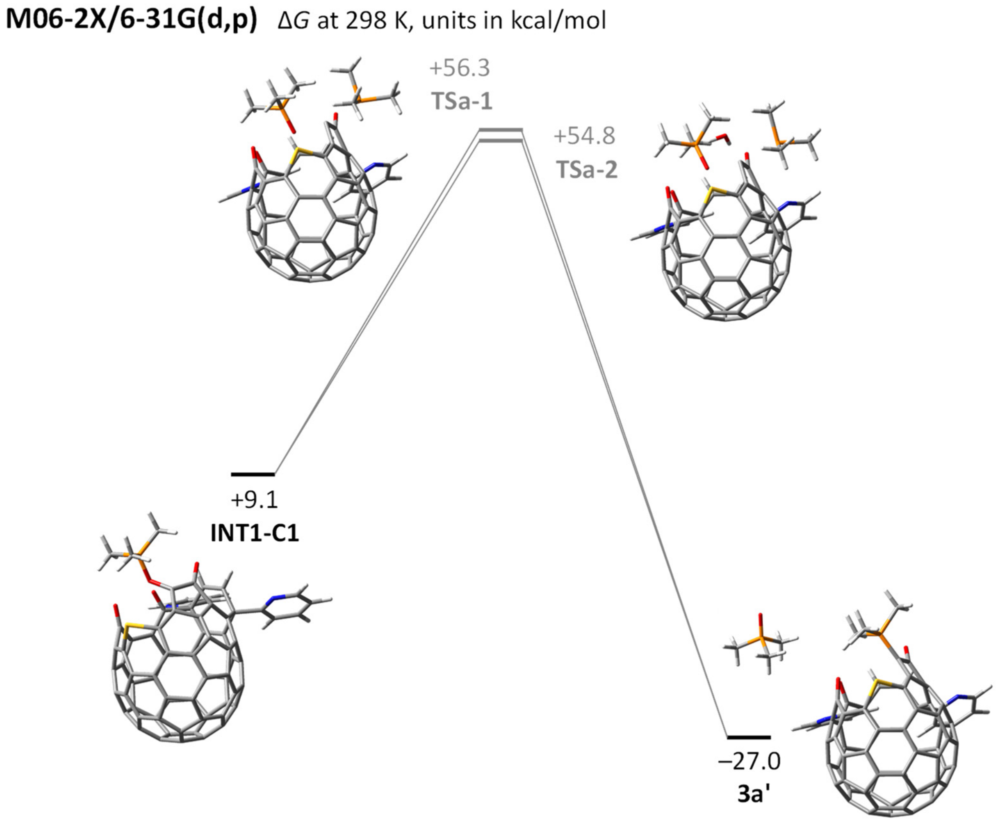

Supplementary Fig. 45. Formation of **3a'** from **INT1-C1** via a concerted pathway (M06-2X/6-31G(d,p)).

Supplementary Table 39. Optimized structure of **TSa-1** (M06-2X/6-31G(d,p))

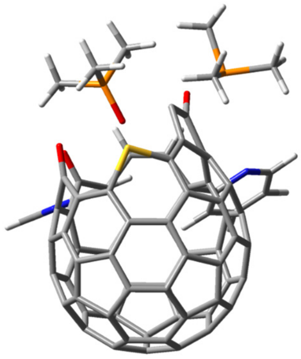

Standard orientation:

Center Number	Atomic Number	Atomic Type	Coordinates (Angstroms) X	Y	Z
1	6	0	-2.469771	2.634639	-0.538890
2	6	0	-2.517365	2.521415	0.783732
3	6	0	-1.312854	2.372825	1.676554
4	6	0	-1.229981	0.950608	2.255795
5	6	0	-2.103279	-0.076721	1.926566
6	6	0	-3.440233	-0.286884	1.218855
7	6	0	-2.062740	-2.271067	1.008214
8	6	0	-1.134716	-3.161019	0.496830
9	6	0	0.143729	-3.380897	1.179793
10	6	0	1.382923	-3.773438	0.486907
11	6	0	1.444061	-3.680773	-0.979456
12	6	0	0.245761	-3.312751	-1.728020
13	6	0	0.370042	-2.459800	-2.810252
14	6	0	-0.593467	-1.369387	-3.270897
15	6	0	0.321770	-0.126044	-3.377767
16	6	0	0.185094	1.143686	-2.837114
17	6	0	-1.146272	1.703531	-2.452167
18	6	0	-1.198401	2.776530	-1.329022
19	6	0	0.033962	2.699373	-0.460155
20	6	0	-0.011387	2.565951	0.906173
21	6	0	1.212324	2.379817	1.653763
22	6	0	1.222271	1.449395	2.778706
23	6	0	0.016561	0.632043	2.959708
24	6	0	0.224562	-0.680537	3.402607
25	6	0	-0.554834	-1.726008	2.828631
26	6	0	-1.616713	-1.412221	2.054676
27	6	0	0.292197	-2.812527	2.454243
28	6	0	1.554175	-2.567801	3.083314
29	6	0	1.517486	-1.239815	3.669707
30	6	0	2.627319	-0.434604	3.622108
31	6	0	2.471124	0.940016	3.187961
32	6	0	3.703244	1.287026	2.509906
33	6	0	3.673972	2.158594	1.443843
34	6	0	2.407567	2.690997	1.013369
35	6	0	2.448988	2.810472	-0.424938
36	6	0	1.292667	2.628062	-1.167511
37	6	0	1.369615	1.872599	-2.419304
38	6	0	2.628638	1.416523	-2.827404
39	6	0	2.764261	0.122233	-3.434853
40	6	0	1.630490	-0.645257	-3.596654
41	6	0	1.659050	-2.047368	-3.248013
42	6	0	2.807236	-2.612698	-2.752027
43	6	0	2.690042	-3.449032	-1.584967
44	6	0	3.907441	-3.285359	-0.835179
45	6	0	3.862273	-3.392963	0.533130
46	6	0	2.604727	-3.671265	1.193881
47	6	0	2.697924	-3.033435	2.492414
48	6	0	3.910006	-2.243668	2.560918
49	6	0	3.874096	-0.962202	3.108952
50	6	0	4.566861	0.117741	2.447850
51	6	0	5.332949	-0.130429	1.315303
52	6	0	5.316710	0.807288	0.207778
53	6	0	4.504133	1.930236	0.280147
54	6	0	3.739769	2.343819	-0.871292
55	6	0	3.830358	1.650826	-2.059541
56	6	0	4.680722	0.478830	-2.147720
57	6	0	4.005260	-0.480861	-2.988390
58	6	0	4.022222	-1.829307	-2.649650
59	6	0	4.725150	-2.265815	-1.469164
60	6	0	5.422923	-1.352712	-0.687899
61	6	0	5.384213	-1.469079	0.757941
62	6	0	4.654439	-2.491017	1.352697
63	6	0	5.394577	0.055230	-1.029934
64	6	0	-1.178300	4.150359	-2.029777
65	6	0	-1.643069	5.289599	-1.370115
66	6	0	-1.556756	6.511921	-2.023945
67	6	0	-1.013521	6.555518	-3.304189
68	6	0	-0.574073	5.364495	-3.871214
69	6	0	-1.447623	3.400806	2.807571
70	6	0	-0.926920	4.689939	2.674805
71	6	0	-1.137539	5.600485	3.702466
72	6	0	-1.860492	5.196557	4.819916
73	6	0	-2.345170	3.892806	4.850400
74	16	0	-1.360744	-3.811639	-1.140403
75	7	0	-0.649295	4.184781	-3.252408
76	7	0	-2.149950	3.008440	3.870784
77	8	0	-1.763503	-1.472989	-3.507035
78	8	0	-4.413455	0.427061	1.223818
79	8	0	-2.157428	1.378172	-3.022106
80	6	0	-3.388443	-1.706322	0.619002
81	8	0	-3.109446	-0.834788	-1.260010
82	15	0	-4.348966	-1.104080	-2.160198
83	6	0	-4.104212	-0.525446	-3.857342
84	6	0	-5.813645	-0.248748	-1.521186
85	6	0	-4.714251	-2.867588	-2.306065
86	1	0	-3.388121	2.672882	-1.117764
87	1	0	-3.473049	2.459309	1.292048
88	1	0	-2.065293	5.205329	-0.374099
89	1	0	-1.910913	7.417546	-1.541486
90	1	0	-0.929696	7.487108	-3.852156
91	1	0	-0.140298	5.352052	-4.868001
92	1	0	-0.362724	4.961724	1.788062
93	1	0	-0.740119	6.608241	3.633035
94	1	0	-2.047208	5.870317	5.648432
95	1	0	-2.918082	3.537369	5.703790
96	1	0	-5.008984	-0.784991	-4.415106
97	1	0	-3.969481	0.556587	-3.854830
98	1	0	-3.226133	-0.987019	-4.300574
99	1	0	-6.162953	-0.770664	-0.619661
100	1	0	-5.556171	0.786742	-1.292721

101	1	0	-6.591860	-0.280844	-2.289497
102	1	0	-3.869708	-3.357817	-2.790679
103	1	0	-4.867035	-3.271382	-1.300565
104	1	0	-5.625584	-2.997586	-2.897041
105	15	0	-4.523754	-3.083777	1.428501
106	6	0	-6.244187	-2.597766	1.043709
107	6	0	-4.562430	-3.285313	3.262772
108	6	0	-4.346890	-4.794849	0.808695
109	1	0	-6.316761	-2.629279	-0.044854
110	1	0	-6.412490	-1.577942	1.399404
111	1	0	-6.959318	-3.278548	1.515632
112	1	0	-3.625781	-3.723065	3.615358
113	1	0	-4.689773	-2.309574	3.742254
114	1	0	-5.391662	-3.936713	3.556110
115	1	0	-3.296912	-5.089509	0.855446
116	1	0	-4.642278	-4.757686	-0.240446
117	1	0	-4.973620	-5.482671	1.384066

The total electronic energy was calculated to be –4555.1116709 Hartree..

An imaginary frequency was found at 561.1827 cm^{-1}.

Supplementary Table 40. Optimized structure of **TSa-2** (M06-2X/6-31G(d,p))

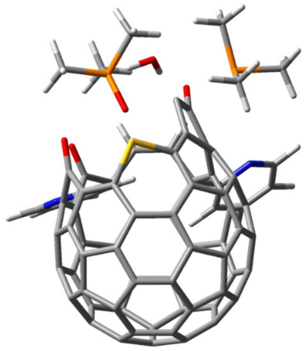

Standard orientation:

Center Number	Atomic Number	Atomic Type	Coordinates (Angstroms) X	Y	Z
1	6	0	-2.351679	2.780030	-0.415892
2	6	0	-2.570771	2.336860	0.817741
3	6	0	-1.487779	1.967940	1.798997
4	6	0	-1.424153	0.457312	2.068246
5	6	0	-2.201424	-0.491210	1.418885
6	6	0	-3.428840	-0.576825	0.521676
7	6	0	-1.942865	-2.444253	0.084611
8	6	0	-0.936045	-3.238593	-0.431155
9	6	0	0.243921	-3.577248	0.369598
10	6	0	1.575294	-3.817869	-0.213660
11	6	0	1.820878	-3.438849	-1.613810
12	6	0	0.712915	-2.952897	-2.428476
13	6	0	0.923255	-1.884309	-3.277037
14	6	0	-0.038385	-0.741194	-3.595764
15	6	0	0.839305	0.518630	-3.356328
16	6	0	0.592277	1.662097	-2.608790
17	6	0	-0.797283	2.118553	-2.290501
18	6	0	-0.995582	3.003208	-1.019946
19	6	0	0.110038	2.744713	-0.027213
20	6	0	-0.106320	2.333834	1.264855
21	6	0	1.019149	2.008251	2.111638
22	6	0	0.917910	0.866579	3.014175
23	6	0	-0.269154	0.016009	2.857974
24	6	0	-0.072464	-1.356448	3.054937
25	6	0	-0.728952	-2.273398	2.182506
26	6	0	-1.677278	-1.814590	1.336169
27	6	0	0.201051	-3.260543	1.732929
28	6	0	1.358238	-3.131184	2.563843
29	6	0	1.193946	-1.944653	3.384644
30	6	0	2.270064	-1.134380	3.646245
31	6	0	2.121299	0.297709	3.476290
32	6	0	3.416910	0.787173	3.051073
33	6	0	3.493568	1.855927	2.185416
34	6	0	2.274325	2.453981	1.708698
35	6	0	2.493533	2.859086	0.339805
36	6	0	1.446847	2.817122	-0.566800
37	6	0	1.694582	2.315088	-1.919145
38	6	0	3.009349	1.961272	-2.242572
39	6	0	3.263623	0.812717	-3.065186
40	6	0	2.185822	0.076362	-3.511035
41	6	0	2.234900	-1.366045	-3.452690
42	6	0	3.339031	-2.002879	-2.940406
43	6	0	3.118995	-3.063013	-1.990262
44	6	0	4.222832	-3.029975	-1.067600
45	6	0	4.009368	-3.409844	0.235246
46	6	0	2.689837	-3.833201	0.656619
47	6	0	2.586871	-3.458379	2.053517
48	6	0	3.747676	-2.681150	2.434529
49	6	0	3.592107	-1.534991	3.212125
50	6	0	4.322953	-0.337058	2.874526
51	6	0	5.236022	-0.345301	1.826850
52	6	0	5.325366	0.794341	0.932590
53	6	0	4.471509	1.872498	1.117994
54	6	0	3.843762	2.500238	-0.018927
55	6	0	4.100531	2.055159	-1.298187
56	6	0	4.997145	0.933748	-1.503726
57	6	0	4.465237	0.151193	-2.594498
58	6	0	4.497796	-1.236947	-2.529092
59	6	0	5.068217	-1.890323	-1.376642
60	6	0	5.625420	-1.141893	-0.347431
61	6	0	5.409759	-1.545092	1.029848
62	6	0	4.651341	-2.674941	1.311978
63	6	0	5.584206	0.305698	-0.408182
64	6	0	-0.879666	4.471780	-1.471910
65	6	0	-1.360026	5.495458	-0.652637
66	6	0	-1.198085	6.808569	-1.073328
67	6	0	-0.565065	7.053680	-2.288113
68	6	0	-0.119114	5.964239	-3.027963
69	6	0	-1.802043	2.713573	3.104399
70	6	0	-1.299282	3.992406	3.350687
71	6	0	-1.676560	4.639376	4.521136
72	6	0	-2.541020	3.992671	5.397802
73	6	0	-2.994421	2.722972	5.053639

74	16	0	-0.907494	-3.639468	-2.156350
75	7	0	-0.268064	4.697671	-2.633177
76	7	0	-2.640353	2.091313	3.933749
77	8	0	-1.185626	-0.822520	-3.922861
78	8	0	-4.430339	0.100897	0.549501
79	8	0	-1.731523	1.855390	-3.008434
80	6	0	-3.239542	-1.831278	-0.359010
81	8	0	-2.933519	-0.657999	-1.851346
82	15	0	-4.194101	-0.208916	-2.655063
83	6	0	-4.713560	1.503818	-2.328766
84	6	0	-5.719720	-1.099182	-2.246938
85	6	0	-3.951977	-0.232007	-4.443872
86	1	0	-3.189409	3.059971	-1.040878
87	1	0	-3.585938	2.213135	1.181877
88	1	0	-1.850502	5.252846	0.284967
89	1	0	-1.561489	7.629572	-0.463275
90	1	0	-0.418912	8.062761	-2.656176
91	1	0	0.381330	6.108427	-3.982203
92	1	0	-0.621746	4.458393	2.642047
93	1	0	-1.298701	5.631850	4.746030
94	1	0	-2.859637	4.455633	6.324830
95	1	0	-3.674654	2.183562	5.708574
96	1	0	-3.989369	-2.355642	-0.938473
97	1	0	-5.677841	1.647884	-2.824966
98	1	0	-4.843506	1.647712	-1.254893
99	1	0	-3.982708	2.198884	-2.737093
100	1	0	-5.592780	-2.184394	-2.245647
101	1	0	-6.045496	-0.764979	-1.257474
102	1	0	-6.468509	-0.805971	-2.988488
103	1	0	-3.090107	0.392551	-4.679178
104	1	0	-3.794460	-1.259743	-4.768236
105	1	0	-4.856861	0.178013	-4.902441
106	15	0	-4.601469	-3.016085	1.430189
107	6	0	-6.370008	-2.696252	1.092242
108	6	0	-4.390013	-2.357158	3.141057
109	6	0	-4.450284	-4.823403	1.650566
110	1	0	-6.568577	-3.111115	0.102588
111	1	0	-6.534068	-1.615168	1.084973
112	1	0	-7.009394	-3.157964	1.850696
113	1	0	-3.397338	-2.615280	3.518914
114	1	0	-4.483374	-1.266745	3.131012
115	1	0	-5.145421	-2.775724	3.813370
116	1	0	-3.400265	-5.084958	1.799353
117	1	0	-4.772152	-5.288437	0.719351
118	1	0	-5.053163	-5.159328	2.499987
119	8	0	-4.997098	-3.991261	-1.405405
120	1	0	-5.695478	-4.586876	-1.698932

The total electronic energy was calculated to be –4631.5177008 Hartree..

An imaginary frequency was found at 635.3131 cm^{-1}.

Supplementary Table 41. Optimized structure of **3a'** (M06-2X/6-31G(d,p))

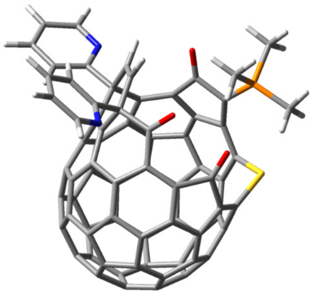

Center Number	Atomic Number	Atomic Type	Coordinates (Angstroms) X	Y	Z
1	6	0	-3.859272	0.006647	0.599017
2	6	0	-3.830663	-0.203744	-0.711204
3	6	0	-2.777943	-1.004276	-1.431747
4	6	0	-1.810636	-0.114835	-2.233591
5	6	0	-1.806006	1.270867	-2.202246
6	6	0	-2.621001	2.385776	-1.533265
7	6	0	-1.665043	3.408744	-1.134095
8	6	0	-0.366105	3.089302	-1.698181
9	6	0	0.971297	3.367035	-1.420248
10	6	0	2.069912	2.581097	-1.969709
11	6	0	3.298770	2.282596	-1.205157
12	6	0	3.330639	2.549236	0.246082
13	6	0	2.211382	3.224164	0.882138
14	6	0	1.731852	2.778549	2.098989
15	6	0	0.265164	2.651468	2.499826
16	6	0	0.181648	1.217013	3.078919
17	6	0	-0.746693	0.212178	2.827593
18	6	0	-2.130817	0.520304	2.334359
19	6	0	-2.885227	-0.598764	1.568946
20	6	0	-1.898295	-1.515527	0.892273
21	6	0	-1.880602	-1.753111	-0.456681
22	6	0	-0.821627	-2.545743	-1.035434
23	6	0	-0.243538	-2.130900	-2.310346
24	6	0	-0.659276	-0.815208	-2.815884
25	6	0	0.322276	-0.064874	-3.475856
26	6	0	0.398184	1.341879	-3.234998
27	6	0	-0.598966	1.946312	-2.550908
28	6	0	1.767223	1.724087	-3.037757
29	6	0	2.559805	0.592939	-3.396757
30	6	0	1.668411	-0.520179	-3.678125
31	6	0	2.018878	-1.793752	-3.309883
32	6	0	1.039830	-2.616509	-2.627677
33	6	0	1.788128	-3.462438	-1.720172
34	6	0	1.235981	-3.824077	-0.510654
35	6	0	-0.076128	-3.340795	-0.171610
36	6	0	-0.092752	-3.077549	1.248273
37	6	0	-0.859724	-2.035583	1.746118
38	6	0	-0.302100	-1.173269	2.790359
39	6	0	0.969928	-1.499163	3.278968
40	6	0	1.904579	-0.460299	3.603092
41	6	0	1.520669	0.850956	3.409969
42	6	0	2.447712	1.777585	2.804609
43	6	0	3.687948	1.355654	2.387505
44	6	0	4.130292	1.745349	1.072090
45	6	0	4.937659	0.668736	0.566393
46	6	0	4.938362	0.423804	-0.785832

47	6	0	4.137757	1.244175	-1.675482
48	6	0	3.764533	0.379811	-2.775483
49	6	0	4.186426	-0.977148	-2.487715
50	6	0	3.332770	-2.046291	-2.749014
51	6	0	3.196867	-3.105881	-1.780108
52	6	0	3.971936	-3.107167	-0.624721
53	6	0	3.385497	-3.506231	0.642043
54	6	0	2.045280	-3.861415	0.689311
55	6	0	1.211909	-3.397128	1.772408
56	6	0	1.737890	-2.620124	2.782214
57	6	0	3.139735	-2.250177	2.746501
58	6	0	3.241498	-0.899047	3.246045
59	6	0	4.116513	-0.004842	2.640993
60	6	0	4.919956	-0.426679	1.519812
61	6	0	4.856680	-1.736835	1.064183
62	6	0	4.872460	-2.002462	-0.363455
63	6	0	4.948864	-0.947021	-1.265967
64	6	0	3.943018	-2.671479	1.689323
65	6	0	-3.659832	-1.439361	2.598251
66	6	0	-4.503253	-2.457261	2.143268
67	6	0	-5.181195	-3.212449	3.088594
68	6	0	-4.993839	-2.930247	4.440566
69	6	0	-4.122063	-1.905491	4.784638
70	6	0	-3.520494	-2.011035	-2.322279
71	6	0	-3.902647	-3.271705	-1.866181
72	6	0	-4.517963	-4.144375	-2.757692
73	6	0	-4.729286	-3.730930	-4.066594
74	6	0	-4.300515	-2.455936	-4.427017
75	16	0	1.331715	4.435355	-0.052579
76	7	0	-3.460778	-1.168950	3.885795
77	7	0	-3.705949	-1.612618	-3.585138
78	8	0	-0.609034	3.454526	2.306803
79	8	0	-3.845003	2.456976	-1.427244
80	8	0	-2.652586	1.590666	2.530973
81	15	0	-2.445308	4.748179	-0.330291
82	6	0	-1.389739	6.076856	0.300794
83	6	0	-3.452218	4.221729	1.079449
84	6	0	-3.590451	5.570699	-1.486969
85	1	0	-4.628351	0.638161	1.033190
86	1	0	-4.570251	0.270625	-1.345653
87	1	0	-4.597021	-2.643112	1.075541
88	1	0	-5.845381	-4.012877	2.777769
89	1	0	-5.505775	-3.494365	5.211982
90	1	0	-3.939180	-1.659292	5.827683
91	1	0	-3.723117	-3.558917	-0.836222
92	1	0	-4.828118	-5.131885	-2.430558
93	1	0	-5.209948	-4.374474	-4.794515
94	1	0	-4.440494	-2.096110	-5.443919
95	1	0	-0.819471	5.701752	1.150297
96	1	0	-2.056270	6.884344	0.617222
97	1	0	-0.713078	6.449452	-0.469801
98	1	0	-4.120893	3.428250	0.744506
99	1	0	-4.030391	5.077353	1.439389
100	1	0	-2.796212	3.837959	1.862274
101	1	0	-4.263500	4.808062	-1.881422
102	1	0	-3.019126	6.016998	-2.303782
103	1	0	-4.162451	6.345933	-0.970496

The total electronic energy was calculated to be –4019.0066956 Hartree..

Supplementary Table 42. Optimized structure of O=PMe3 (M06-2X/6-31G(d,p))

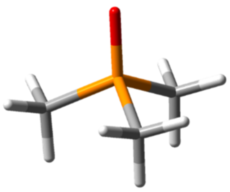

Standard orientation:

Center Number	Atomic Number	Atomic Type	Coordinates (Angstroms) X	Y	Z
1	8	0	0.000115	-0.002279	1.696840
2	15	0	0.000035	-0.000621	0.195974
3	6	0	-0.042396	1.668113	-0.562136
4	6	0	1.466301	-0.796142	-0.565080
5	6	0	-1.424056	-0.869419	-0.564987
6	1	0	-0.041227	1.615984	-1.656277
7	1	0	-0.942971	2.192808	-0.228396
8	1	0	0.830596	2.237622	-0.228630
9	1	0	1.418593	-0.771226	-1.659212
10	1	0	1.526281	-1.836728	-0.231581
11	1	0	2.370437	-0.276348	-0.233260
12	1	0	-1.431061	-1.911764	-0.231667
13	1	0	-2.353374	-0.396268	-0.232972
14	1	0	-1.377809	-0.841849	-1.659112

The total electronic energy was calculated to be –536.2181587 Hartree..

11.7. P–C Bond Formation from INT2-^{V}P in an S_N1 or S_N2 Fashion

The hydrolysis of **INT1-C1** is considered to give **INT2-^{V}P**. This pentavalent phosphorus compound would be a possible intermediate for the formation of **3a'**. Thus, we examined those pathways in an S_N1 or S_N2 fashion (Supplementary Fig. 46). The required energy to form a new P–C bond was estimated to be over 55 kcal/mol in both cases. Hence, these pathways are also not reliable.

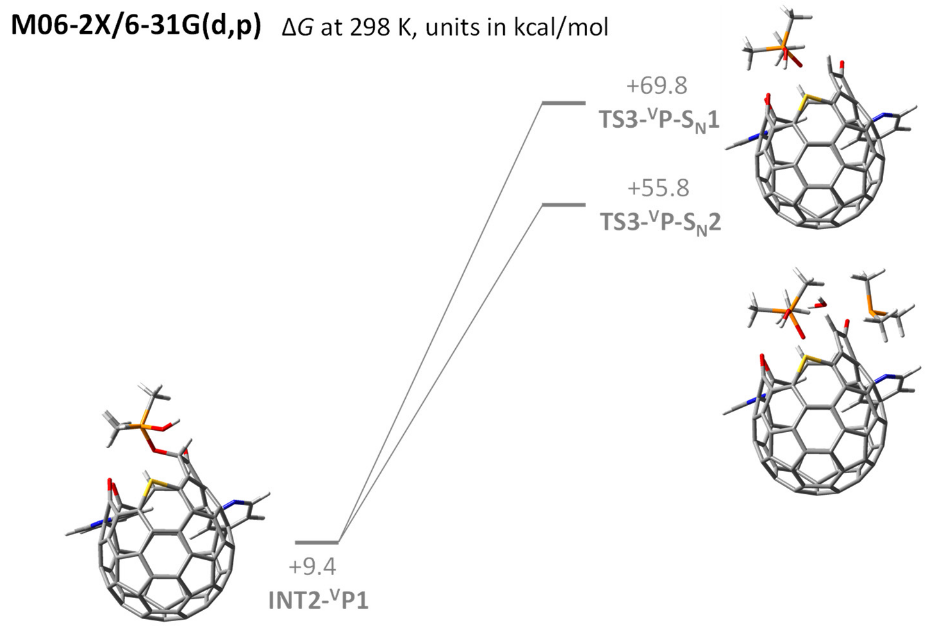

Supplementary Fig. 46. P–C bond formation from **INT2-^{V}P** in an S_N1 or S_N2 Fashion (M06-2X/6-31G(d,p)).

Supplementary Table 43. Optimized structure of **TS3-^{V}P-S$_{N}$1** (M06-2X/6-31G(d,p))

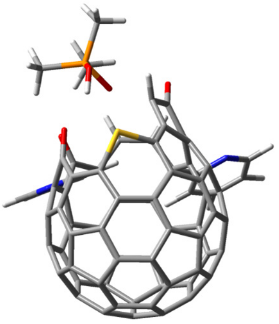

Standard orientation:

Center Number	Atomic Number	Atomic Type	X	Y	Z
1	6	0	3.390702	1.696982	0.422369
2	6	0	3.172981	2.032363	-0.844908
3	6	0	1.831039	2.407552	-1.416731
4	6	0	1.261336	1.300537	-2.321818
5	6	0	1.848098	0.055360	-2.498051
6	6	0	3.124588	-0.650480	-2.094529
7	6	0	1.326079	-2.264848	-2.260456
8	6	0	0.280463	-3.065948	-1.858995
9	6	0	-1.095207	-2.765833	-2.257035
10	6	0	-2.266391	-3.102178	-1.426750
11	6	0	-2.061074	-3.515029	-0.028543
12	6	0	-0.707821	-3.704179	0.475462
13	6	0	-0.383279	-3.246217	1.739297
14	6	0	0.921700	-2.572540	2.165843
15	6	0	0.432357	-1.299058	2.894541
16	6	0	0.837688	0.019258	2.739002
17	6	0	2.185138	0.365087	2.174518
18	6	0	2.349525	1.762610	1.504677
19	6	0	1.017495	2.252355	0.989419
20	6	0	0.790037	2.591507	-0.321976
21	6	0	-0.544307	2.924936	-0.763821
22	6	0	-0.998767	2.453085	-2.068723
23	6	0	-0.116917	1.505360	-2.765720
24	6	0	-0.749430	0.491720	-3.498351
25	6	0	-0.209913	-0.829411	-3.463962
26	6	0	1.016801	-1.036750	-2.918688
27	6	0	-1.264650	-1.767759	-3.227740
28	6	0	-2.491292	-1.054424	-3.422880
29	6	0	-2.175088	0.351266	-3.588574
30	6	0	-2.991211	1.309066	-3.041245
31	6	0	-2.388451	2.388749	-2.282993
32	6	0	-3.344559	2.737921	-1.251855
33	6	0	-2.896502	3.165578	-0.021085
34	6	0	-1.479534	3.240957	0.215380
35	6	0	-1.241993	2.864190	1.589177
36	6	0	-0.074827	2.198673	1.932354
37	6	0	-0.145003	1.083363	2.878485
38	6	0	-1.395187	0.789822	3.437383
39	6	0	-1.787592	-0.573730	3.656156
40	6	0	-0.909042	-1.572062	3.292357
41	6	0	-1.400908	-2.732988	2.586426
42	6	0	-2.731255	-2.834799	2.258050
43	6	0	-3.063209	-3.229919	0.912036
44	6	0	-4.286514	-2.554388	0.569590
45	6	0	-4.500438	-2.192851	-0.738788
46	6	0	-3.501093	-2.492548	-1.743710
47	6	0	-3.611096	-1.437829	-2.733393
48	6	0	-4.542835	-0.432590	-2.267504
49	6	0	-4.235498	0.918861	-2.412970
50	6	0	-4.477502	1.828985	-1.318701
51	6	0	-5.084307	1.377580	-0.152943
52	6	0	-4.619969	1.850829	1.138735
53	6	0	-3.549872	2.732307	1.195645
54	6	0	-2.517381	2.552749	2.187838
55	6	0	-2.594854	1.525020	3.103318
56	6	0	-3.714803	0.604148	3.058757
57	6	0	-3.206961	-0.706966	3.386812
58	6	0	-3.668822	-1.819655	2.692691
59	6	0	-4.657115	-1.662258	1.654382
60	6	0	-5.183981	-0.408747	1.369371
61	6	0	-5.420278	-0.026999	-0.010820
62	6	0	-5.121012	-0.913391	-1.037707
63	6	0	-4.698253	0.754550	2.083996
64	6	0	2.814536	2.738615	2.602188
65	6	0	3.422112	3.947500	2.257075
66	6	0	3.792985	4.814364	3.276241
67	6	0	3.545734	4.448520	4.595988
68	6	0	2.929743	3.225159	4.833742
69	6	0	2.022526	3.701851	-2.219382
70	6	0	1.951053	4.948810	-1.593840
71	6	0	2.202474	6.085716	-2.350383
72	6	0	2.519616	5.940621	-3.697145
73	6	0	2.574370	4.653860	-4.222274
74	16	0	0.569453	-4.294018	-0.602455
75	7	0	2.566867	2.384556	3.862545
76	7	0	2.334389	3.553315	-3.506138
77	8	0	2.040310	-2.927069	1.926961
78	8	0	4.254365	-0.247547	-2.006794
79	8	0	3.121599	-0.389258	2.247785
80	6	0	2.806083	-2.178383	-1.960934
81	8	0	3.388985	-2.659006	-0.415739
82	15	0	4.219147	-4.000886	-0.150803
83	6	0	5.051573	-3.031376	1.226663
84	6	0	5.782525	-4.238928	-1.096780
85	6	0	3.672870	-5.357942	0.956229
86	8	0	3.277599	-4.797616	-1.461798
87	1	0	4.376898	1.369160	0.736742
88	1	0	3.980839	1.978270	-1.565550
89	1	0	3.602019	4.184284	1.213332
90	1	0	4.271841	5.760618	3.044664
91	1	0	3.822423	5.092373	5.422978
92	1	0	2.716783	2.899775	5.848915
93	1	0	1.695015	5.017620	-0.540940
94	1	0	2.148326	7.070141	-1.896239
95	1	0	2.719949	6.799688	-4.327180
96	1	0	2.821158	4.494673	-5.269320
97	1	0	3.503535	-2.790451	-2.547900
98	1	0	5.776138	-3.664074	1.751747
99	1	0	5.575847	-2.170702	0.800563
100	1	0	4.316937	-2.652644	1.939156
101	1	0	5.765491	-5.146445	-1.699764
102	1	0	5.887461	-3.364968	-1.752158

103	1	0	6.641212	-4.246206	-0.425070
104	1	0	4.550423	-5.709832	1.505013
105	1	0	2.979385	-4.918067	1.676471
106	1	0	3.202935	-6.170318	0.408973
107	1	0	3.665891	-4.708506	-2.338251

The total electronic energy was calculated to be –4170.4820911 Hartree..

An imaginary frequency was found at 3184.3449 cm^{-1}.

Supplementary Table 44. Optimized structure of **TS3-^{V}P-S$_{N}$2** (M06-2X/6-31G(d,p))

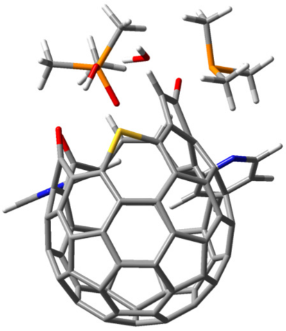

Standard orientation:

Center Number	Atomic Number	Atomic Type	Coordinates (Angstroms) X	Y	Z
1	6	0	-1.843532	2.977728	-0.755665
2	6	0	-1.977862	2.892947	0.563115
3	6	0	-0.856604	2.588906	1.522489
4	6	0	-1.008479	1.178477	2.116389
5	6	0	-2.000238	0.280404	1.747164
6	6	0	-3.313463	0.251021	0.968033
7	6	0	-2.223383	-1.911500	0.849429
8	6	0	-1.405457	-2.931907	0.397183
9	6	0	-0.209994	-3.320250	1.151678
10	6	0	0.996101	-3.895285	0.531637
11	6	0	1.148621	-3.835005	-0.929748
12	6	0	0.057002	-3.312417	-1.746439
13	6	0	0.359576	-2.502622	-2.826659
14	6	0	-0.412563	-1.293864	-3.348433
15	6	0	0.674882	-0.194705	-3.413964
16	6	0	0.691606	1.089813	-2.891534
17	6	0	-0.565184	1.838732	-2.585352
18	6	0	-0.524265	2.925584	-1.475137
19	6	0	0.635985	2.687923	-0.538687
20	6	0	0.498645	2.583511	0.824010
21	6	0	1.641343	2.237331	1.639734
22	6	0	1.458097	1.332483	2.770641
23	6	0	0.140267	0.697306	2.890791
24	6	0	0.135014	-0.624470	3.354658
25	6	0	-0.753354	-1.557617	2.746419
26	6	0	-1.716503	-1.108452	1.912362
27	6	0	-0.050852	-2.758965	2.427979
28	6	0	1.197426	-2.685959	3.124168
29	6	0	1.318810	-1.357251	3.697362
30	6	0	2.532974	-0.718422	3.705182
31	6	0	2.597796	0.657547	3.252449
32	6	0	3.901463	0.815732	2.641119
33	6	0	4.054057	1.665983	1.568382
34	6	0	2.901433	2.365867	1.064237
35	6	0	3.036776	2.455915	-0.370438
36	6	0	1.907906	2.427840	-1.174572
37	6	0	1.943678	1.649758	-2.414304
38	6	0	3.144997	1.013447	-2.748417
39	6	0	3.127327	-0.296267	-3.337406
40	6	0	1.906145	-0.897618	-3.555916
41	6	0	1.715860	-2.284022	-3.195422
42	6	0	2.743429	-2.998712	-2.632171
43	6	0	2.445662	-3.791750	-1.466991
44	6	0	3.631840	-3.790772	-0.652121
45	6	0	3.498251	-3.869625	0.712366
46	6	0	2.180174	-3.956467	1.304479
47	6	0	2.293290	-3.318219	2.601224
48	6	0	3.600064	-2.707375	2.730754
49	6	0	3.717653	-1.425417	3.266051
50	6	0	4.591762	-0.465040	2.636080
51	6	0	5.374491	-0.836907	1.549714
52	6	0	5.551622	0.076325	0.435792
53	6	0	4.904612	1.304154	0.454317
54	6	0	4.270033	1.804038	-0.740900
55	6	0	4.324768	1.086838	-1.916914
56	6	0	5.002988	-0.195142	-1.948751
57	6	0	4.243910	-1.062268	-2.818162
58	6	0	4.050342	-2.394066	-2.468611
59	6	0	4.619371	-2.907474	-1.247623
60	6	0	5.397059	-2.090547	-0.435926
61	6	0	5.264432	-2.177786	1.006396
62	6	0	4.365580	-3.076533	1.567626
63	6	0	5.588029	-0.698298	-0.789858
64	6	0	-0.270973	4.271628	-2.184275
65	6	0	-0.603509	5.475372	-1.560214
66	6	0	-0.308886	6.662796	-2.217660
67	6	0	0.303104	6.609025	-3.466101
68	6	0	0.598252	5.359058	-3.998677
69	6	0	-0.904200	3.643043	2.636326
70	6	0	-0.198769	4.843077	2.522705
71	6	0	-0.332499	5.790134	3.530048
72	6	0	-1.164687	5.510222	4.608763
73	6	0	-1.831093	4.289022	4.622374
74	16	0	-1.633454	-3.569232	-1.244998
75	7	0	0.322545	4.211688	-3.375913
76	7	0	-1.711437	3.370808	3.661945
77	8	0	-1.571007	-1.234128	-3.648295
78	8	0	-4.173888	1.095769	0.913502
79	8	0	-1.580234	1.651262	-3.207968
80	6	0	-3.432226	-1.170554	0.382943
81	8	0	-2.964235	-0.428986	-1.361335
82	15	0	-4.285713	-0.627521	-2.280663
83	6	0	-4.385129	1.188155	-2.902385
84	6	0	-5.917384	-0.555272	-1.494760
85	6	0	-4.128165	-1.326365	-3.939419
86	8	0	-4.629383	-2.456309	-1.732003
87	1	0	-2.714578	3.136839	-1.384860

88	1	0	-2.958595	2.974910	1.018108
89	1	0	-1.086401	5.467311	-0.588529
90	1	0	-0.555849	7.616876	-1.762586
91	1	0	0.548153	7.510689	-4.015746
92	1	0	1.078825	5.269744	-4.969744
93	1	0	0.445276	5.018295	1.666549
94	1	0	0.207592	6.730137	3.474994
95	1	0	-1.297813	6.216434	5.420426
96	1	0	-2.493874	4.031726	5.445392
97	1	0	-4.334732	-1.621773	-0.017242
98	1	0	-5.247826	1.165249	-3.574923
99	1	0	-4.576447	1.877556	-2.079538
100	1	0	-3.489190	1.508481	-3.426991
101	1	0	-6.176719	-1.556900	-1.125101
102	1	0	-5.891794	0.164394	-0.674910
103	1	0	-6.646701	-0.240713	-2.247156
104	1	0	-3.155745	-1.044511	-4.343626
105	1	0	-4.211249	-2.414813	-3.863258
106	1	0	-4.933910	-0.939188	-4.569934
107	1	0	-3.773241	-2.700866	-1.365285
108	15	0	-4.803181	-1.685758	2.459272
109	6	0	-6.430531	-0.856708	2.366699
110	6	0	-4.112266	-1.041726	4.044998
111	6	0	-5.166227	-3.439857	2.826069
112	1	0	-6.949789	-1.331746	1.532468
113	1	0	-6.276255	0.204437	2.154440
114	1	0	-6.983456	-0.971479	3.304083
115	1	0	-3.194161	-1.574847	4.302136
116	1	0	-3.876999	0.022524	3.943983
117	1	0	-4.836728	-1.170507	4.855285
118	1	0	-4.250372	-4.026066	2.731028
119	1	0	-5.858518	-3.763905	2.048215
120	1	0	-5.598214	-3.545888	3.825611
121	8	0	-6.971294	-3.538984	-2.000520
122	1	0	-6.986205	-4.083704	-2.793025
123	1	0	-6.030624	-3.234252	-1.885060

The total electronic energy was calculated to be –4707.9252352 Hartree..

An imaginary frequency was found at 935.3694 cm^{-1}.

11.8. P–C Bond Formation from INT2-(2H_2O)/OH^- in an S_N2 Fashion

The protonation of **INT1-C1** would give **INT2-(2H_2O)**. The nucleophilic addition of PMe_3 to **INT2-(2H_2O)** in an S_N2 fashion was examined (Supplementary Fig. 47). The activation barrier was estimated to be over 45 kcal/mol, suggestive of an undesirable pathway.

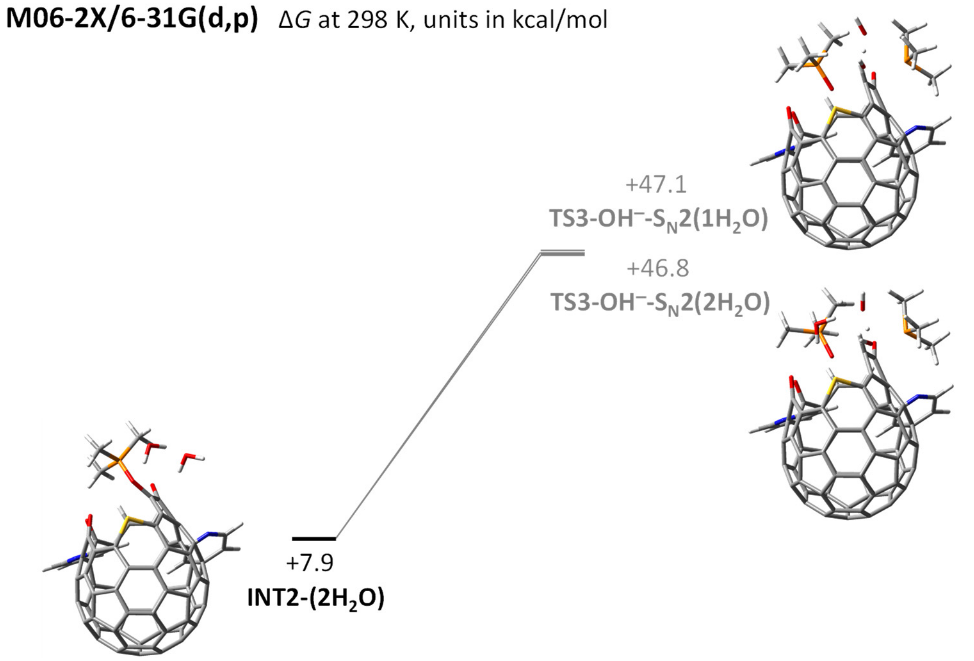

Supplementary Fig. 47. P–C bond formation from **INT2-(2H_2O)** in a S_N2 fashion (M06-2X/6-31G(d,p)).

Supplementary Table 45. Optimized structure of **TS3-OH$^-$-S_N2(1H_2O)** (M06-2X/6-31G(d,p))

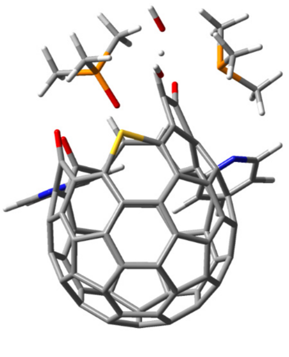

Standard orientation:

Center Number	Atomic Number	Atomic Type	Coordinates (Angstroms) X	Y	Z
1	6	0	-1.308444	3.446498	-0.868530
2	6	0	-1.425449	3.439501	0.455152
3	6	0	-0.377888	2.926948	1.408283
4	6	0	-0.785744	1.586195	2.044885
5	6	0	-1.921871	0.869064	1.697391
6	6	0	-3.138213	1.052458	0.815718
7	6	0	-2.548437	-1.295569	0.901086

8	6	0	-1.960512	-2.484661	0.531425
9	6	0	-0.860349	-3.055554	1.309047
10	6	0	0.207083	-3.878826	0.711563
11	6	0	0.343186	-3.925691	-0.753655
12	6	0	-0.647240	-3.262785	-1.590737
13	6	0	-0.231006	-2.545707	-2.697481
14	6	0	-0.791273	-1.211530	-3.192279
15	6	0	0.478691	-0.346753	-3.369947
16	6	0	0.740666	0.941150	-2.923721
17	6	0	-0.367545	1.904173	-2.610167
18	6	0	-0.059065	3.057513	-1.608454
19	6	0	1.056524	2.651664	-0.675506
20	6	0	0.933323	2.636735	0.692382
21	6	0	2.001053	2.110648	1.510954
22	6	0	1.666947	1.304559	2.681655
23	6	0	0.253744	0.929847	2.836880
24	6	0	0.003079	-0.347396	3.356594
25	6	0	-1.056912	-1.126445	2.802381
26	6	0	-1.940523	-0.543743	1.951012
27	6	0	-0.587572	-2.454217	2.546429
28	6	0	0.667259	-2.577229	3.226021
29	6	0	1.039125	-1.268588	3.728592
30	6	0	2.351253	-0.867979	3.699601
31	6	0	2.666911	0.450128	3.182945
32	6	0	3.967978	0.334721	2.555874
33	6	0	4.259172	1.091568	1.441949
34	6	0	3.250767	1.973082	0.917116
35	6	0	3.369983	1.970686	-0.522147
36	6	0	2.238800	2.118583	-1.310506
37	6	0	2.091279	1.284392	-2.504813
38	6	0	3.145361	0.417770	-2.820044
39	6	0	2.873727	-0.892023	-3.341367
40	6	0	1.557819	-1.267804	-3.508827
41	6	0	1.132987	-2.584136	-3.090729
42	6	0	2.026587	-3.450735	-2.508916
43	6	0	1.617394	-4.130810	-1.305535
44	6	0	2.798463	-4.301683	-0.501815
45	6	0	2.676432	-4.290318	0.866872
46	6	0	1.374328	-4.106891	1.474536
47	6	0	1.620456	-3.428356	2.732716
48	6	0	3.018668	-3.063590	2.820151
49	6	0	3.378479	-1.801828	3.289758
50	6	0	4.405484	-1.050970	2.607272
51	6	0	5.087113	-1.611555	1.533914
52	6	0	5.409289	-0.800493	0.373575
53	6	0	5.004984	0.526383	0.337694
54	6	0	4.449689	1.080376	-0.873696
55	6	0	4.339586	0.310093	-2.011621
56	6	0	4.768908	-1.075321	-1.987442
57	6	0	3.844154	-1.826524	-2.802959
58	6	0	3.423074	-3.085054	-2.388243
59	6	0	3.915853	-3.638457	-1.151110
60	6	0	4.843182	-2.939658	-0.388319
61	6	0	4.723108	-2.933292	1.058068
62	6	0	3.686106	-3.624214	1.672072
63	6	0	5.277172	-1.623697	-0.812323
64	6	0	0.437453	4.256847	-2.438165
65	6	0	0.417140	5.543596	-1.896392
66	6	0	0.907616	6.590967	-2.664603
67	6	0	1.399296	6.320508	-3.938106
68	6	0	1.380854	5.003563	-4.382963
69	6	0	-0.188724	3.993828	2.495532
70	6	0	0.698276	5.056615	2.308366
71	6	0	0.779656	6.033598	3.291732
72	6	0	-0.025769	5.920316	4.420386
73	6	0	-0.882845	4.828298	4.506572
74	16	0	-2.338082	-3.171821	-1.067267
75	7	0	0.913936	3.988287	-3.653088
76	7	0	-0.970135	3.882398	3.568865
77	8	0	-1.940922	-0.909391	-3.337418
78	8	0	-3.811673	2.023827	0.593590
79	8	0	-1.454585	1.816353	-3.122023
80	6	0	-3.651164	-0.337750	0.466842
81	8	0	-3.552449	-0.183306	-1.113161
82	15	0	-4.700267	-0.781368	-2.191135
83	6	0	-4.215651	0.674445	-3.275594
84	6	0	-6.476750	-0.474831	-1.809259
85	6	0	-4.445826	-2.244535	-3.268143
86	8	0	-5.002535	-2.193138	-1.025781
87	1	0	-2.141513	3.766990	-1.486722
88	1	0	-2.353755	3.753838	0.918108
89	1	0	0.015209	5.705659	-0.901395
90	1	0	0.902905	7.604890	-2.276962
91	1	0	1.787674	7.108003	-4.573741
92	1	0	1.755843	4.746751	-5.370503
93	1	0	1.312721	5.104048	1.414445
94	1	0	1.463883	6.868856	3.180166
95	1	0	0.006573	6.656902	5.215036
96	1	0	-1.531750	4.702292	5.370051
97	1	0	-4.664019	-0.464903	0.062426
98	1	0	-4.782967	0.654763	-4.213027
99	1	0	-4.433140	1.608707	-2.749250
100	1	0	-3.147321	0.659841	-3.497474
101	1	0	-7.023555	-1.398592	-1.621806
102	1	0	-6.499205	0.153687	-0.909926
103	1	0	-6.950863	0.090011	-2.612230
104	1	0	-4.989154	-2.067033	-4.200011
105	1	0	-3.379544	-2.282767	-3.502111
106	1	0	-4.773988	-3.165360	-2.794146
107	1	0	-5.668166	-2.819856	-1.327510
108	15	0	-4.665355	-0.755334	1.843302
109	6	0	-6.180502	-1.784909	1.846002
110	6	0	-5.321566	0.777941	2.626150
111	6	0	-3.781262	-1.483780	3.281607
112	1	0	-5.939686	-2.807444	1.544233
113	1	0	-6.907648	-1.387952	1.133074
114	1	0	-6.636819	-1.807897	2.841246
115	1	0	-4.505916	1.471938	2.844570
116	1	0	-6.014001	1.279414	1.945218
117	1	0	-5.847673	0.545235	3.557992
118	1	0	-2.903405	-0.882965	3.532803
119	1	0	-3.437637	-2.492167	3.037316
120	1	0	-4.436948	-1.535719	4.157231
121	8	0	-4.304973	-2.665877	0.968956
122	1	0	-4.176207	-3.543278	1.286900
123	1	0	-4.240271	-2.445021	-0.157781

The total electronic energy was calculated to be –4707.9330382 Hartree..

An imaginary frequency was found at 936.9880 cm^{-1}.

Supplementary Table 46. Optimized structure of **TS3-OH⁻-S_N2(2H_2O)** (M06-2X/6-31G(d,p))

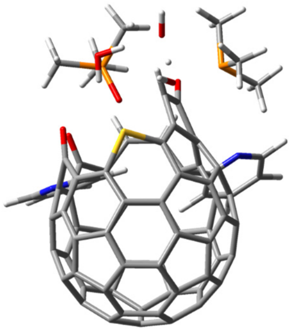

Standard orientation:

Center Number	Atomic Number	Atomic Type	Coordinates (Angstroms) X	Y	Z
1	6	0	-1.631025	3.214408	-0.456570
2	6	0	-1.741156	3.038768	0.855130
3	6	0	-0.612462	2.609176	1.755983
4	6	0	-0.828269	1.177107	2.261069
5	6	0	-1.865734	0.367318	1.831639
6	6	0	-3.221318	0.443299	1.135942
7	6	0	-2.184877	-1.711957	0.748279
8	6	0	-1.501129	-2.815339	0.287756
9	6	0	-0.337466	-3.339818	1.005121
10	6	0	0.810370	-3.965337	0.321492
11	6	0	0.930226	-3.845199	-1.142149
12	6	0	-0.145463	-3.228881	-1.905993
13	6	0	0.153844	-2.337400	-2.918915
14	6	0	-0.577083	-1.037164	-3.263378
15	6	0	0.571365	-0.009414	-3.353925
16	6	0	0.684134	1.247145	-2.773272
17	6	0	-0.515818	2.059012	-2.388921
18	6	0	-0.351372	3.093016	-1.230660
19	6	0	0.817745	2.728462	-0.349266
20	6	0	0.716829	2.559533	1.010612
21	6	0	1.857464	2.089305	1.765528
22	6	0	1.651546	1.129582	2.847527
23	6	0	0.299927	0.569394	2.969248
24	6	0	0.219854	-0.778824	3.345827
25	6	0	-0.733368	-1.613906	2.691279
26	6	0	-1.662351	-1.042301	1.889021
27	6	0	-0.115174	-2.841484	2.296287
28	6	0	1.149995	-2.885515	2.964060
29	6	0	1.364894	-1.602111	3.609187
30	6	0	2.617033	-1.040734	3.618758
31	6	0	2.758487	0.355120	3.248218
32	6	0	4.051111	0.469179	2.603960
33	6	0	4.223605	1.369777	1.576014
34	6	0	3.104187	2.169121	1.153476
35	6	0	3.199477	2.332838	-0.277775
36	6	0	2.045987	2.419151	-1.041666
37	6	0	1.986315	1.707979	-2.320110
38	6	0	3.134348	1.020816	-2.731965
39	6	0	3.017300	-0.249489	-3.389871
40	6	0	1.754624	-0.767084	-3.591820
41	6	0	1.505351	-2.162833	-3.317953
42	6	0	2.510573	-2.967575	-2.836407
43	6	0	2.211752	-3.821037	-1.714089
44	6	0	3.417167	-3.924511	-0.935974
45	6	0	3.315391	-4.073591	0.426332
46	6	0	2.010225	-4.119376	1.053264
47	6	0	2.191989	-3.553837	2.376835
48	6	0	3.535892	-3.030101	2.500930
49	6	0	3.743379	-1.789949	3.102530
50	6	0	4.658360	-0.848175	2.502070
51	6	0	5.385608	-1.201440	1.371594
52	6	0	5.585408	-0.237143	0.305288
53	6	0	5.014408	1.022390	0.414702
54	6	0	4.375357	1.628712	-0.727558
55	6	0	4.344663	0.978143	-1.942379
56	6	0	4.943929	-0.336193	-2.069927
57	6	0	4.105804	-1.106264	-2.958334
58	6	0	3.853502	-2.445231	-2.682691
59	6	0	4.432129	-3.060794	-1.513572
60	6	0	5.276689	-2.336219	-0.681950
61	6	0	5.180592	-2.499643	0.757270
62	6	0	4.247672	-3.376754	1.296470
63	6	0	5.536781	-0.938694	-0.963049
64	6	0	-0.020629	4.444291	-1.895672
65	6	0	-0.298288	5.648533	-1.247172
66	6	0	0.065601	6.833910	-1.874199
67	6	0	0.689601	6.777056	-3.115966
68	6	0	0.926869	5.525767	-3.674424
69	6	0	-0.553707	3.599742	2.926087
70	6	0	0.196512	4.773490	2.829174
71	6	0	0.164484	5.671106	3.888137
72	6	0	-0.615842	5.370184	4.999658
73	6	0	-1.333727	4.178848	4.993878
74	16	0	-1.818667	-3.412413	-1.356001
75	7	0	0.584690	4.381014	-3.081390
76	7	0	-1.310255	3.306767	3.983795
77	8	0	-1.757250	-0.871095	-3.403374
78	8	0	-4.103249	1.243859	1.317849
79	8	0	-1.559305	1.957373	-2.985699
80	6	0	-3.305542	-0.836021	0.268399
81	8	0	-2.815547	0.153279	-1.302458
82	15	0	-4.155074	0.336905	-2.283807
83	6	0	-4.355710	2.200544	-2.149009
84	6	0	-5.804785	-0.126427	-1.592975
85	6	0	-4.065895	0.244209	-4.124125
86	8	0	-4.120098	-1.652709	-2.266106
87	1	0	-2.497107	3.501396	-1.038118
88	1	0	-2.705219	3.152448	1.343724
89	1	0	-0.792601	5.645452	-0.281404
90	1	0	-0.138437	7.788934	-1.400201
91	1	0	0.985746	7.677174	-3.642260
92	1	0	1.412950	5.433489	-4.642550
93	1	0	0.792550	4.967700	1.942854
94	1	0	0.741646	6.589553	3.847110
95	1	0	-0.669747	6.039233	5.850935
96	1	0	-1.958450	3.907881	5.841747
97	1	0	-4.157223	-1.287764	-0.210652
98	1	0	-5.330230	2.487976	-2.558198

99	1	0	-4.310947	2.519555	-1.105247
100	1	0	-3.580866	2.705230	-2.725152
101	1	0	-6.005799	-1.195409	-1.619228
102	1	0	-5.897939	0.250937	-0.569438
103	1	0	-6.555772	0.384878	-2.202094
104	1	0	-3.178423	0.790471	-4.453567
105	1	0	-4.052819	-0.774856	-4.500621
106	1	0	-4.946162	0.775661	-4.498622
107	1	0	-3.194559	-1.814576	-2.033562
108	15	0	-4.863969	-1.974440	1.919733
109	6	0	-6.625821	-1.839231	1.386615
110	6	0	-4.905837	-0.646284	3.204830
111	6	0	-4.420859	-3.383726	3.031148
112	1	0	-6.733137	-2.382989	0.439743
113	1	0	-6.827118	-0.782466	1.192640
114	1	0	-7.344269	-2.214573	2.121560
115	1	0	-3.922089	-0.575567	3.677281
116	1	0	-5.112642	0.306823	2.708863
117	1	0	-5.661896	-0.819182	3.977697
118	1	0	-3.464967	-3.164702	3.514834
119	1	0	-4.294142	-4.282290	2.420730
120	1	0	-5.171769	-3.583608	3.802501
121	8	0	-4.851068	-3.432735	0.565735
122	1	0	-5.636604	-3.419361	-0.419330
123	1	0	-4.151969	-4.050198	0.862541
124	8	0	-5.640952	-3.218447	-1.666610
125	1	0	-5.546675	-4.062909	-2.068601
126	1	0	-5.144180	-2.363888	-2.447985

The total electronic energy was calculated to be –4784.3401762 Hartree..

An imaginary frequency was found at 902.5856 cm^{-1}.

11.9. P–C Bond Formation from INT2-($2H_2O$)/A$^-$ in an S_N2 Fashion

During the formation of **INT2**, a hydroxide anion (OH^-) co-exists as a counter anion. Since the activation barrier for **TS3-OH⁻** was suggested to be quite high, we considered that the hydroxide anion should be stabilized by the reaction with **INT1-C** to provide stable fullerenyl anions **A⁻** (Supplementary Fig. 48). Among eight possible anions, the anionic charges on **A1⁻**, **A3⁻**, and **A4⁻** are expected to be entirely delocalized by the effective π-conjugation along with the C_{60} cages. Thus, **TS3** was calculated by replacing OH^- with **A1⁻**, **A3⁻**, and **A4⁻**. Note that the calculations were performed on the basis of one of enantiomers, i.e., ^{f}C-**1'**. Since anions having the structural similarity with **A3⁻** and **A4⁻** formed by the reaction of dicarbonyl compounds and phosphines have been reported in some organic compounds[6,7], fullerenyl anions **A3⁻** and **A4⁻** should be most probable in our reaction system.

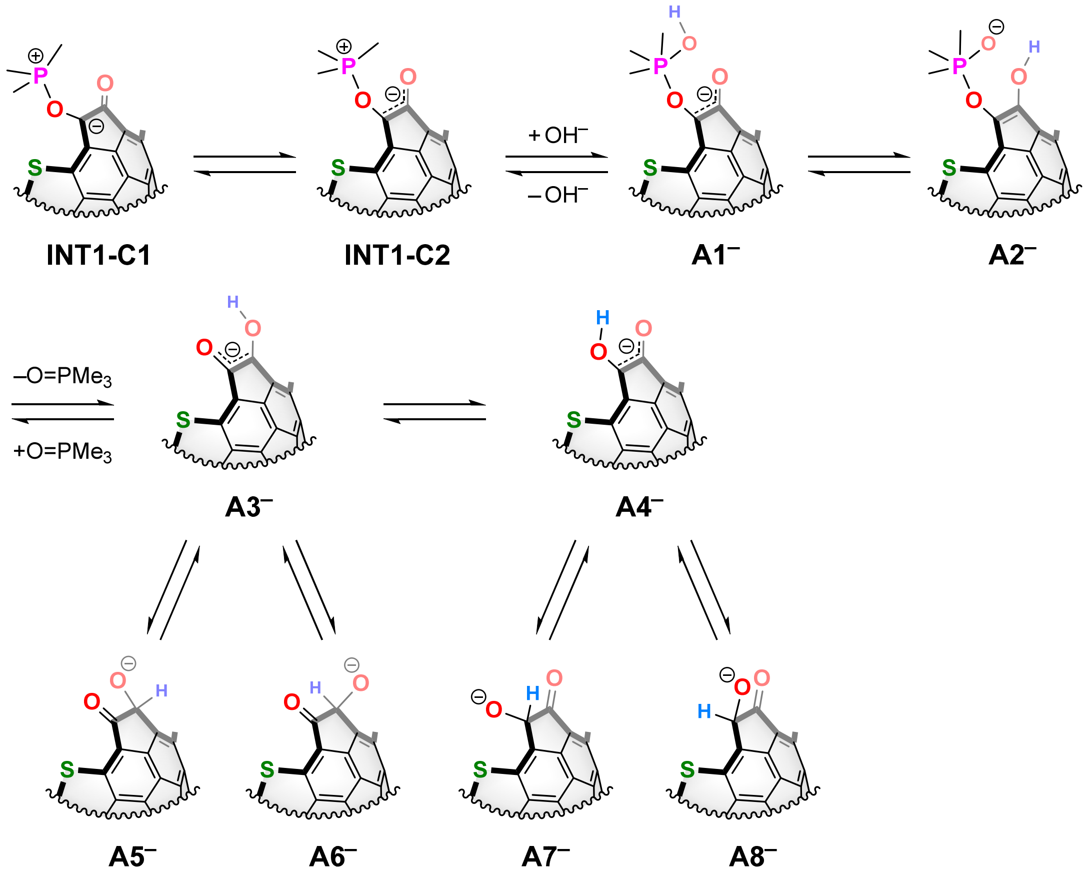

Supplementary Fig. 48. Possible formation of stable fullerenyl anions **A⁻**.

The results suggested that counter anion **A1⁻** increases the activation barrier ($\Delta G^{\ddagger}$ 55.6 kcal/mol) when compared with OH^{-} (46.8 kcal/mol) (Supplementary Fig. 49). This is probably due to the bulky pentavalent phosphorus substituent which prevents the close contact of the enolate moiety in **A1⁻** with the cationic center in **TS3-A1⁻-S_N2**. Contrastingly, the hydroxy enolate moiety of **A3⁻** and **A4⁻** in **TS3-A⁻-S_N2** is arranged in a similar manner with the hydrated hydroxide anion in **TS3-OH⁻-S_N2**. This leads to the significant decrease in the activation barrier in both cases (14.7 kcal/mol for **A3⁻** and 11.0 kcal/mol for **A4⁻**).

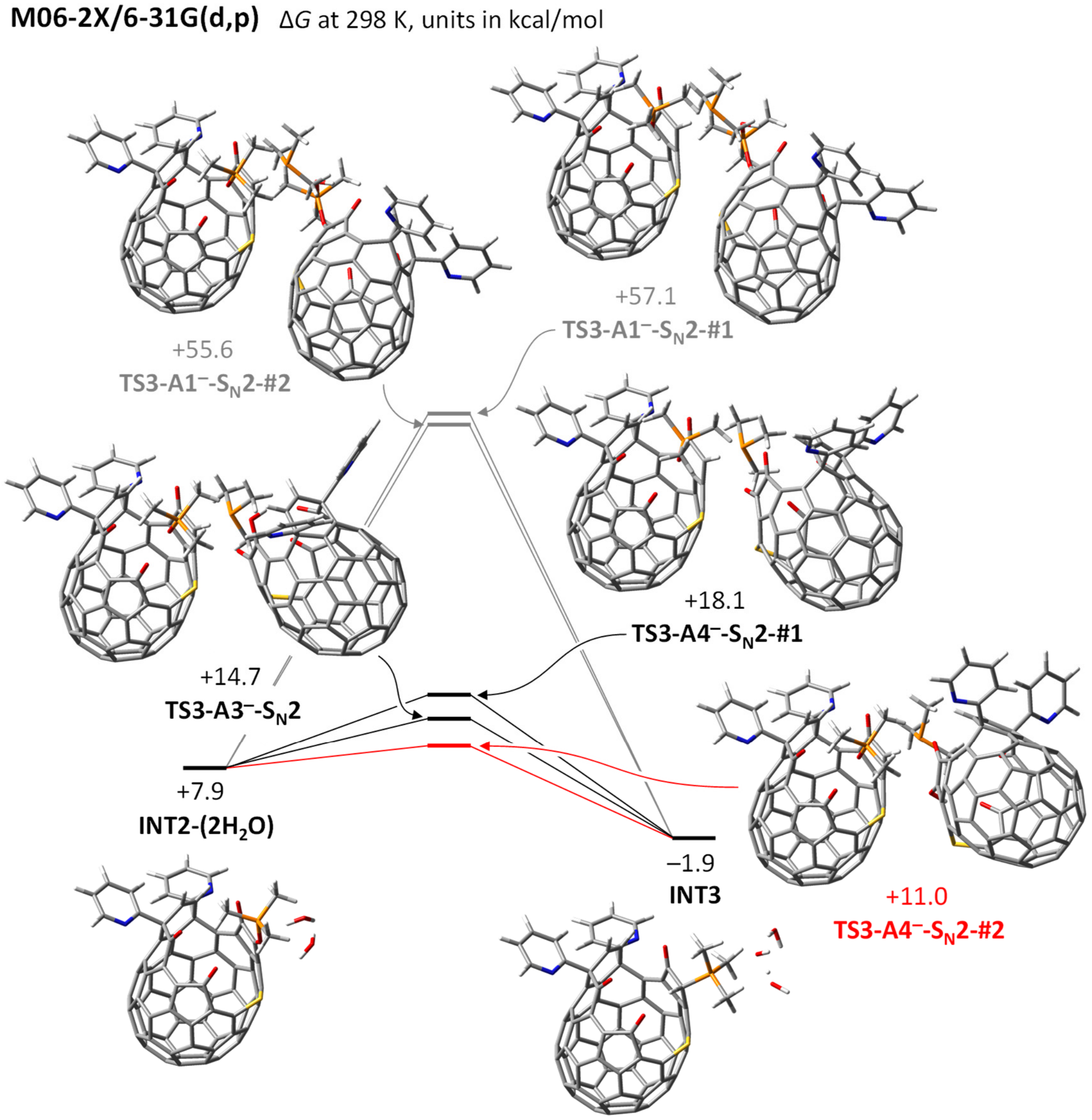

Supplementary Fig. 49. P–C bond formation from **INT2-(2H_2O)/A⁻** in an S_N2 fashion (M06-2X/6-31G(d,p)).

Supplementary Table 47. Optimized structure of **TS3-A1⁻-S$_N$2-#1** (M06-2X/6-31G(d,p))

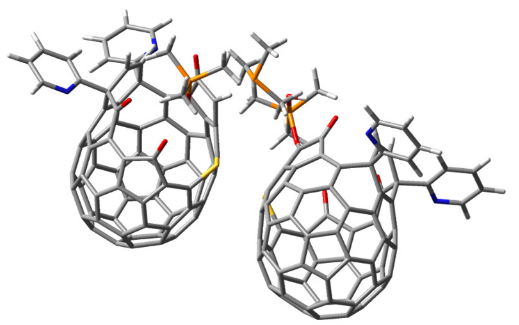

Standard orientation:

Center Number	Atomic Number	Atomic Type	X	Y	Z
1	6	0	8.224327	1.872118	-0.752636
2	6	0	7.594696	2.573671	0.184189
3	6	0	6.892317	1.973384	1.375045
4	6	0	5.365475	2.117776	1.294918
5	6	0	4.681116	2.650210	0.211550
6	6	0	4.947707	3.445853	-1.059204
7	6	0	2.996934	2.033343	-1.351951
8	6	0	2.173881	0.961143	-1.641180
9	6	0	1.520480	0.200627	-0.572125
10	6	0	1.199833	-1.233362	-0.679374
11	6	0	1.794587	-2.018532	-1.772150
12	6	0	2.583046	-1.339541	-2.794269
13	6	0	3.749002	-1.927903	-3.242089
14	6	0	5.060859	-1.238609	-3.612188
15	6	0	6.109647	-2.040518	-2.792550
16	6	0	7.116087	-1.603934	-1.941767
17	6	0	7.704798	-0.231653	-2.045715
18	6	0	8.354433	0.376570	-0.763418
19	6	0	7.753781	-0.247575	0.471256
20	6	0	7.143998	0.471572	1.469289
21	6	0	6.502677	-0.221980	2.563549
22	6	0	5.239504	0.291209	3.082469
23	6	0	4.618130	1.382929	2.321531
24	6	0	3.219384	1.367885	2.255295
25	6	0	2.589724	1.701856	1.020424
26	6	0	3.334166	2.223410	0.020477
27	6	0	1.578305	0.738572	0.719422
28	6	0	1.377050	-0.020556	1.915017
29	6	0	2.399336	0.364789	2.871572
30	6	0	2.978159	-0.581784	3.679019
31	6	0	4.422668	-0.604599	3.800533
32	6	0	4.786551	-1.993942	3.991089
33	6	0	5.975618	-2.465557	3.480041
34	6	0	6.825884	-1.562634	2.749795
35	6	0	7.443995	-2.313316	1.681881
36	6	0	7.728061	-1.690757	0.477317
37	6	0	7.457927	-2.401106	-0.773550
38	6	0	6.980992	-3.713242	-0.677488
39	6	0	5.982636	-4.184774	-1.594806
40	6	0	5.510449	-3.311769	-2.552695
41	6	0	4.091601	-3.238859	-2.813267
42	6	0	3.213990	-4.019502	-2.100883
43	6	0	2.038591	-3.384051	-1.562630
44	6	0	1.734434	-4.052611	-0.325144
45	6	0	1.152396	-3.334074	0.690835
46	6	0	0.856961	-1.928536	0.503418
47	6	0	0.973789	-1.326250	1.817201
48	6	0	1.485594	-2.303220	2.755450
49	6	0	2.475503	-1.939698	3.666748
50	6	0	3.594264	-2.821965	3.897253
51	6	0	3.648220	-4.062617	3.273351
52	6	0	4.909622	-4.561545	2.757204
53	6	0	6.047803	-3.775208	2.867151
54	6	0	6.963563	-3.671274	1.757514
55	6	0	6.733710	-4.365900	0.589098
56	6	0	5.550994	-5.196457	0.466283
57	6	0	5.074471	-5.069519	-0.890889
58	6	0	3.709429	-4.982298	-1.138527
59	6	0	2.774324	-5.025748	-0.041206
60	6	0	3.225536	-5.197200	1.261448
61	6	0	2.607193	-4.446721	2.338672
62	6	0	1.569034	-3.564690	2.063712
63	6	0	4.648713	-5.278616	1.523845
64	6	0	9.848313	-0.000124	-0.794239
65	6	0	10.759647	0.687566	0.009774
66	6	0	12.089846	0.290553	-0.011925
67	6	0	12.462347	-0.773015	-0.828350
68	6	0	11.477352	-1.394313	-1.587886
69	6	0	7.417692	2.710799	2.615622
70	6	0	8.539865	2.256376	3.310694
71	6	0	9.004163	3.006889	4.383994
72	6	0	8.338497	4.180405	4.721397
73	6	0	7.236327	4.551464	3.957419
74	16	0	2.104458	0.304095	-3.284743
75	7	0	10.195310	-1.022259	-1.574301
76	7	0	6.780803	3.840551	2.925108
77	8	0	5.212720	-0.299978	-4.337217
78	8	0	5.748524	4.334829	-1.236685
79	8	0	7.722242	0.373709	-3.090092
80	6	0	3.866886	3.019287	-2.076493
81	8	0	5.241231	2.090160	-3.049029
82	15	0	6.014303	2.916360	-4.124385
83	6	0	7.692811	3.406081	-3.621692
84	6	0	5.298708	4.538080	-4.504963
85	6	0	6.292938	2.012463	-5.662073
86	1	0	8.737115	2.387919	-1.553740
87	1	0	7.556004	3.656797	0.122469
88	1	0	10.420590	1.513784	0.626957
89	1	0	12.826321	0.804022	0.598181
90	1	0	13.489751	-1.115176	-0.877795
91	1	0	11.722538	-2.229477	-2.239191
92	1	0	9.025882	1.331096	3.017414
93	1	0	9.870507	2.678413	4.949597
94	1	0	8.661100	4.796369	5.553111
95	1	0	6.689419	5.463615	4.184329
96	1	0	3.577407	3.524726	-2.989547
97	1	0	8.068174	4.096589	-4.382691
98	1	0	7.649989	3.925689	-2.663062

99	1	0	8.336678	2.530410	-3.570452
100	1	0	4.225582	4.494410	-4.705716
101	1	0	5.487474	5.193986	-3.649956
102	1	0	5.832033	4.925282	-5.378007
103	1	0	6.818344	1.087675	-5.423255
104	1	0	5.332909	1.809733	-6.134745
105	1	0	6.909265	2.645660	-6.307383
106	15	0	2.581897	5.035783	-1.215694
107	6	0	3.217851	6.516391	-2.080299
108	6	0	2.868933	5.448694	0.560000
109	6	0	0.763227	5.101111	-1.377024
110	1	0	3.031366	6.349670	-3.142549
111	1	0	4.293015	6.593127	-1.896310
112	1	0	2.720961	7.425776	-1.729073
113	1	0	2.400035	4.694740	1.197571
114	1	0	3.943374	5.457746	0.768110
115	1	0	2.448311	6.429603	0.802357
116	1	0	0.325990	4.207000	-0.927044
117	1	0	0.534011	5.074394	-2.441805
118	1	0	0.366801	6.003109	-0.900499
119	6	0	-7.243994	3.268954	0.740411
120	6	0	-6.391205	3.633542	-0.209921
121	6	0	-6.020161	2.789203	-1.398453
122	6	0	-4.582239	2.253792	-1.273599
123	6	0	-3.779159	2.409320	-0.157444
124	6	0	-3.844427	3.058008	1.236855
125	6	0	-2.520341	1.130392	1.392013
126	6	0	-2.113078	-0.194979	1.659312
127	6	0	-1.866879	-1.134739	0.597274
128	6	0	-2.226167	-2.566722	0.687001
129	6	0	-3.137339	-3.007916	1.757235
130	6	0	-3.567583	-2.065197	2.786684
131	6	0	-4.887540	-2.061477	3.204175
132	6	0	-5.753791	-0.832290	3.498253
133	6	0	-7.066769	-1.144320	2.728036
134	6	0	-7.801735	-0.323972	1.884764
135	6	0	-7.763472	1.169908	1.999687
136	6	0	-8.049686	2.001186	0.711307
137	6	0	-7.774804	1.172586	-0.521540
138	6	0	-6.895887	1.550191	-1.506570
139	6	0	-6.590333	0.646394	-2.592366
140	6	0	-5.220263	0.571929	-3.090251
141	6	0	-4.203335	1.282269	-2.314837
142	6	0	-2.949717	0.664036	-2.232172
143	6	0	-2.240810	0.695470	-0.991387
144	6	0	-2.707397	1.470792	0.000705
145	6	0	-1.703086	-0.615869	-0.711912
146	6	0	-1.875261	-1.380907	-1.887774
147	6	0	-2.636664	-0.590464	-2.848464
148	6	0	-3.551438	-1.191609	-3.675082
149	6	0	-4.858421	-0.592342	-3.807507
150	6	0	-5.783160	-1.689030	-4.008264
151	6	0	-7.069081	-1.603924	-3.519472
152	6	0	-7.459390	-0.422316	-2.793989
153	6	0	-8.356810	-0.830938	-1.740135
154	6	0	-8.371396	-0.142210	-0.535042
155	6	0	-8.444494	-0.900254	0.713486
156	6	0	-8.565131	-2.292778	0.617436
157	6	0	-7.869262	-3.142643	1.541106
158	6	0	-7.069930	-2.551182	2.499630
159	6	0	-5.761673	-3.093551	2.772806
160	6	0	-5.298438	-4.181403	2.068857
161	6	0	-3.957448	-4.128549	1.544955
162	6	0	-3.963119	-4.863354	0.309306
163	6	0	-3.118867	-4.467205	-0.700568
164	6	0	-2.232653	-3.333258	-0.510816
165	6	0	-2.051685	-2.749794	-1.816515
166	6	0	-2.951427	-3.392928	-2.754178
167	6	0	-3.680812	-2.642476	-3.675484
168	6	0	-5.064497	-2.951675	-3.913720
169	6	0	-5.663321	-4.054812	-3.305482
170	6	0	-7.019294	-3.955191	-2.801058
171	6	0	-7.707849	-2.754475	-2.917878
172	6	0	-8.501850	-2.264894	-1.816245
173	6	0	-8.609280	-2.990863	-0.648333
174	6	0	-7.897833	-4.249614	-0.517549
175	6	0	-7.423906	-4.334265	0.844475
176	6	0	-6.154116	-4.839765	1.102731
177	6	0	-5.320381	-5.289079	0.014471
178	6	0	-5.790560	-5.253413	-1.292503
179	6	0	-4.899890	-4.845938	-2.364305
180	6	0	-3.583034	-4.496614	-2.075306
181	6	0	-7.107560	-4.713107	-1.566890
182	6	0	-9.550963	2.339295	0.709325
183	6	0	-10.028922	3.403315	-0.061418
184	6	0	-11.394793	3.646621	-0.082081
185	6	0	-12.237556	2.826454	0.663114
186	6	0	-11.664199	1.793083	1.394540
187	6	0	-6.164026	3.677721	-2.640916
188	6	0	-7.364833	3.729463	-3.356857
189	6	0	-7.466250	4.605551	-4.428759
190	6	0	-6.373613	5.404361	-4.750286
191	6	0	-5.228589	5.286884	-3.969394
192	16	0	-2.442867	-0.807196	3.298019
193	7	0	-10.353190	1.547368	1.420653
194	7	0	-5.117933	4.449356	-2.938027
195	8	0	-5.449409	0.163966	4.087130
196	8	0	-4.365862	4.138276	1.561746
197	8	0	-7.560710	1.727543	3.048767
198	6	0	-3.115759	2.180108	2.116502
199	8	0	-3.190222	2.238198	3.471839
200	15	0	-2.809410	3.514171	4.533943
201	6	0	-3.355194	2.263929	5.846638
202	6	0	-4.149686	4.722356	4.895883
203	6	0	-1.187487	3.815160	5.378689
204	8	0	-2.206386	4.462511	3.255688
205	1	0	-7.382287	3.895492	1.616115
206	1	0	-5.826083	4.552105	-0.102472
207	1	0	-9.329443	4.017577	-0.619304
208	1	0	-11.797179	4.465569	-0.670866
209	1	0	-13.310724	2.980556	0.679417
210	1	0	-12.283783	1.126521	1.990283
211	1	0	-8.191544	3.084895	-3.075521
212	1	0	-8.383760	4.661396	-5.007056
213	1	0	-6.403746	6.099593	-5.582055
214	1	0	-4.352228	5.896106	-4.181437
215	1	0	-3.357520	2.747127	6.831436
216	1	0	-4.351501	1.878975	5.620265
217	1	0	-2.670152	1.410315	5.873767
218	1	0	-3.736040	5.731704	4.918366
219	1	0	-4.834604	4.655149	4.043758
220	1	0	-4.689833	4.497413	5.815681
221	1	0	-1.255862	3.633855	6.451811
222	1	0	-0.481813	3.095953	4.949903
223	1	0	-0.820900	4.817540	5.163304
224	1	0	-2.903359	4.615192	2.593402

The total electronic energy was calculated to be –8725.7275745 Hartree..

An imaginary frequency was found at 951.0820 cm^{-1}.

Supplementary Table 48. Optimized structure of **TS3-A1^{-}-S$_{N}$2-#2** (M06-2X/6-31G(d,p))

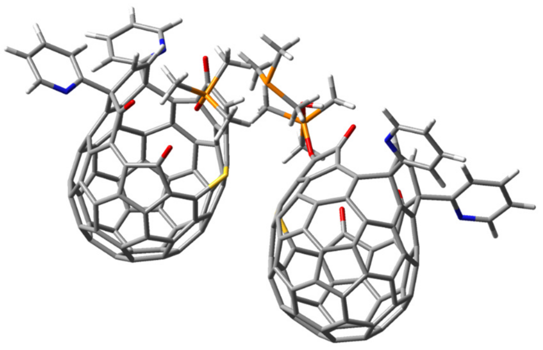

Standard orientation:

Center Number	Atomic Number	Atomic Type	Coordinates (Angstroms) X	Y	Z
1	6	0	7.326118	-2.618596	0.777356
2	6	0	6.637079	-3.074116	-0.264482
3	6	0	6.188960	-2.233132	-1.432468
4	6	0	4.665833	-2.019867	-1.453734
5	6	0	3.797306	-2.470389	-0.467407
6	6	0	3.808761	-3.379791	0.747920
7	6	0	2.202568	-1.582723	1.066672
8	6	0	1.670893	-0.357571	1.434167
9	6	0	1.287328	0.626607	0.423332
10	6	0	1.322570	2.083872	0.652003
11	6	0	1.999398	2.618085	1.838950
12	6	0	2.542350	1.703162	2.834632
13	6	0	3.774426	1.967319	3.399420
14	6	0	4.864193	0.972049	3.775415
15	6	0	6.115188	1.544013	3.069592
16	6	0	7.030602	0.929647	2.224718
17	6	0	7.230587	-0.553043	2.223856
18	6	0	7.807397	-1.203222	0.931003
19	6	0	7.467764	-0.360013	-0.274281
20	6	0	6.783916	-0.831597	-1.367504
21	6	0	6.403475	0.079468	-2.424366
22	6	0	5.097139	-0.072938	-3.054183
23	6	0	4.184057	-1.043271	-2.434732
24	6	0	2.826893	-0.691395	-2.431460
25	6	0	2.051396	-0.964533	-1.265994
26	6	0	2.574786	-1.749268	-0.301007
27	6	0	1.283083	0.190502	-0.911227
28	6	0	1.356268	1.072953	-2.034945
29	6	0	2.311996	0.529555	-2.981120
30	6	0	3.151731	1.369091	-3.669643
31	6	0	4.566186	1.048462	-3.721144
32	6	0	5.260826	2.318893	-3.763830
33	6	0	6.490111	2.447026	-3.155243
34	6	0	7.047307	1.311440	-2.469405
35	6	0	7.750651	1.803925	-1.308182
36	6	0	7.793222	1.040804	-0.152491
37	6	0	7.625443	1.700142	1.143310
38	6	0	7.486389	3.094226	1.141686
39	6	0	6.576161	3.730109	2.051549
40	6	0	5.852423	2.935518	2.915374
41	6	0	4.441219	3.187986	3.104233
42	6	0	3.820621	4.198693	2.414493
43	6	0	2.566529	3.903150	1.768347
44	6	0	2.508327	4.717379	0.586055
45	6	0	1.845548	4.232842	-0.517356
46	6	0	1.222357	2.931411	-0.471982
47	6	0	1.267366	2.423928	-1.834752
48	6	0	2.053986	3.316906	-2.653109
49	6	0	2.985994	2.800783	-3.555615
50	6	0	4.294781	3.400515	-3.650454
51	6	0	4.601355	4.538759	-2.913331
52	6	0	5.907012	4.675533	-2.295621
53	6	0	6.831376	3.647933	-2.423252
54	6	0	7.618285	3.240246	-1.285343
55	6	0	7.485913	3.880848	-0.071899
56	6	0	6.532034	4.964299	0.069732
57	6	0	5.951476	4.857633	1.387386
58	6	0	4.590710	5.083304	1.563807
59	6	0	3.764542	5.429576	0.434493
60	6	0	4.328447	5.584810	-0.826478
61	6	0	3.622478	5.087751	-1.993440
62	6	0	2.391903	4.461531	-1.844822
63	6	0	5.743433	5.338534	-1.015776
64	6	0	9.341315	-1.210665	1.080318
65	6	0	10.122113	-1.940835	0.181668
66	6	0	11.502125	-1.899548	0.323341
67	6	0	12.051271	-1.134701	1.349306
68	6	0	11.186743	-0.442490	2.188504
69	6	0	6.618890	-2.984410	-2.702109
70	6	0	7.792687	-2.670967	-3.387286
71	6	0	8.140900	-3.441545	-4.491162
72	6	0	7.314023	-4.493276	-4.865982
73	6	0	6.171447	-4.732111	-4.107165
74	16	0	1.729276	0.147340	3.135057
75	7	0	9.857194	-0.474159	2.061892
76	7	0	5.826107	-4.001586	-3.046483
77	8	0	4.750967	-0.009999	4.450058
78	8	0	4.396769	-4.417810	0.957314
79	8	0	6.987294	-1.230171	3.196476
80	6	0	2.788907	-2.771187	1.727704
81	8	0	4.194106	-2.204416	2.889714
82	15	0	4.654264	-3.217251	3.967396
83	6	0	5.152703	-4.846507	3.361893
84	6	0	3.324163	-3.596492	5.156841
85	6	0	6.034729	-2.646344	4.980162
86	1	0	7.632477	-3.303891	1.558875
87	1	0	6.349194	-4.119144	-0.315314
88	1	0	9.644359	-2.523338	-0.600301
89	1	0	12.141035	-2.456083	-0.355043
90	1	0	13.123387	-1.074264	1.497234
91	1	0	11.572141	0.168128	3.001090
92	1	0	8.409955	-1.836938	-3.070439
93	1	0	9.043164	-3.218433	-5.051621
94	1	0	7.543861	-5.115720	-5.723099
95	1	0	5.499850	-5.548187	-4.362807
96	1	0	2.346392	-3.288131	2.556736
97	1	0	5.308972	-5.500471	4.224413

98	1	0	4.361581	-5.251692	2.729963
99	1	0	6.080522	-4.738267	2.801855
100	1	0	2.964099	-2.689270	5.649741
101	1	0	2.502336	-4.098238	4.638676
102	1	0	3.720610	-4.269697	5.920765
103	1	0	6.916420	-2.552590	4.346679
104	1	0	5.806368	-1.668729	5.405341
105	1	0	6.200551	-3.381985	5.772224
106	15	0	1.221327	-4.455814	0.501956
107	6	0	1.225159	-6.080682	1.358719
108	6	0	1.591625	-4.863089	-1.242205
109	6	0	-0.497153	-3.860592	0.525109
110	1	0	0.887126	-5.957255	2.390978
111	1	0	2.243097	-6.480328	1.359975
112	1	0	0.560861	-6.787127	0.852678
113	1	0	0.849211	-5.560253	-1.638647
114	1	0	1.513760	-3.950183	-1.837621
115	1	0	2.606499	-5.265223	-1.313573
116	1	0	-0.582497	-3.083443	-0.239715
117	1	0	-0.743265	-3.437125	1.502540
118	1	0	-1.212543	-4.649592	0.273412
119	6	0	-6.576237	-3.480746	-0.601434
120	6	0	-5.782856	-3.672752	0.446098
121	6	0	-5.600922	-2.692252	1.573326
122	6	0	-4.220500	-2.018526	1.504616
123	6	0	-3.321620	-2.187520	0.467396
124	6	0	-3.197230	-2.969566	-0.852392
125	6	0	-2.087392	-0.921354	-1.112605
126	6	0	-1.781122	0.408067	-1.481607
127	6	0	-1.730076	1.459489	-0.497553
128	6	0	-2.192724	2.835767	-0.755442
129	6	0	-3.036859	3.090652	-1.936447
130	6	0	-3.283221	2.022339	-2.898036
131	6	0	-4.555584	1.838095	-3.407127
132	6	0	-5.267150	0.498689	-3.642008
133	6	0	-6.660959	0.734809	-2.998972
134	6	0	-7.376088	-0.080838	-2.133411
135	6	0	-7.170906	-1.565110	-2.092986
136	6	0	-7.496483	-2.303228	-0.759250
137	6	0	-7.413463	-1.346174	0.405957
138	6	0	-6.597740	-1.544582	1.492593
139	6	0	-6.475459	-0.519166	2.504267
140	6	0	-5.165989	-0.252910	3.093853
141	6	0	-4.023093	-0.921050	2.469981
142	6	0	-2.833554	-0.185560	2.418454
143	6	0	-2.035130	-0.251136	1.232729
144	6	0	-2.348181	-1.153193	0.288848
145	6	0	-1.616115	1.077142	0.859465
146	6	0	-1.941634	1.923916	1.948896
147	6	0	-2.692925	1.146990	2.927658
148	6	0	-3.728636	1.722708	3.617471
149	6	0	-4.979069	1.003714	3.713658
150	6	0	-6.019169	2.012073	3.736231
151	6	0	-7.242068	1.747787	3.159174
152	6	0	-7.452711	0.470906	2.527878
153	6	0	-8.290983	0.687170	1.372493
154	6	0	-8.130840	-0.103536	0.244090
155	6	0	-8.169531	0.526539	-1.076003
156	6	0	-8.437596	1.899932	-1.127089
157	6	0	-7.755565	2.733474	-2.075723
158	6	0	-6.824747	2.147898	-2.910140
159	6	0	-5.560693	2.804352	-3.140578
160	6	0	-5.268898	3.993727	-2.512611
161	6	0	-3.977876	4.130047	-1.887921
162	6	0	-4.159101	4.962998	-0.729274
163	6	0	-3.366576	4.749834	0.373448
164	6	0	-2.363174	3.700579	0.357297
165	6	0	-2.241827	3.250048	1.725422
166	6	0	-3.269768	3.883902	2.530851
167	6	0	-3.996427	3.144480	3.462633
168	6	0	-5.420693	3.328195	3.568918
169	6	0	-6.067519	4.300223	2.809634
170	6	0	-7.361558	4.016961	2.218038
171	6	0	-7.936095	2.765933	2.400317
172	6	0	-8.582713	2.098277	1.296084
173	6	0	-8.657855	2.699903	0.057804
174	6	0	-8.066725	4.009494	-0.142118
175	6	0	-7.491165	4.022917	-1.467019
176	6	0	-6.262549	4.638692	-1.678925
177	6	0	-5.571058	5.267234	-0.579506
178	6	0	-6.142625	5.297096	0.686069
179	6	0	-5.309073	5.080922	1.854759
180	6	0	-3.945121	4.848906	1.701337
181	6	0	-7.418827	4.647639	0.912887
182	6	0	-8.952915	-2.793972	-0.851420
183	6	0	-9.397820	-3.827442	-0.023340
184	6	0	-10.729717	-4.210582	-0.100136
185	6	0	-11.569176	-3.554015	-0.995137
186	6	0	-11.029840	-2.535503	-1.772518
187	6	0	-5.754574	-3.472426	2.885339
188	6	0	-7.005285	-3.617108	3.493313
189	6	0	-7.104208	-4.389791	4.642169
190	6	0	-5.957367	-4.996576	5.144344
191	6	0	-4.763509	-4.801839	4.458891
192	16	0	-2.004304	0.837852	-3.191427
193	7	0	-9.751891	-2.156572	-1.706027
194	7	0	-4.653555	-4.061555	3.354496
195	8	0	-4.810378	-0.504465	-4.107454
196	8	0	-3.510794	-4.150013	-1.081526
197	8	0	-6.799251	-2.187139	-3.055927
198	6	0	-2.512151	-2.090499	-1.768105
199	8	0	-2.444273	-2.300182	-3.108852
200	15	0	-1.672608	-3.539246	-3.966406
201	6	0	-2.226580	-2.558616	-5.474892
202	6	0	-2.657611	-5.038658	-4.366137
203	6	0	0.106086	-3.527495	-4.474709
204	8	0	-1.123909	-4.262874	-2.485551
205	1	0	-6.583664	-4.195772	-1.418333
206	1	0	-5.133293	-4.539963	0.478823
207	1	0	-8.701565	-4.312512	0.653090
208	1	0	-11.107528	-5.011021	0.528566
209	1	0	-12.615409	-3.821540	-1.090858
210	1	0	-11.649067	-1.994518	-2.484056
211	1	0	-7.874445	-3.123915	3.069800
212	1	0	-8.061304	-4.514145	5.139207
213	1	0	-5.983474	-5.605367	6.041061
214	1	0	-3.844638	-5.263019	4.815389
215	1	0	-1.972402	-3.113916	-6.385491
216	1	0	-3.303241	-2.377991	-5.442371
217	1	0	-1.730296	-1.583729	-5.503686
218	1	0	-2.053361	-5.933919	-4.213907
219	1	0	-3.474678	-5.051384	-3.636084
220	1	0	-3.074369	-5.011548	-5.372617
221	1	0	0.560367	-2.665289	-3.971600
222	1	0	0.614218	-4.428626	-4.131882
223	1	0	0.217473	-3.394456	-5.550789
224	1	0	-1.904799	-4.487269	-1.946228

The total electronic energy was calculated to be –8725.72804206 Hartree..

An imaginary frequency was found at 872.7248 cm^{-1}.

Supplementary Table 49. Optimized structure of **TS3-A3^{-}-S$_{N}$2** (M06-2X/6-31G(d,p))

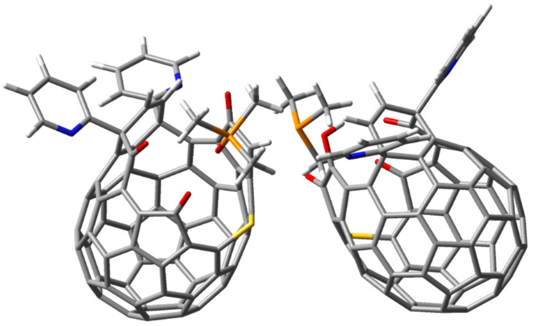

Standard orientation:

Center Number	Atomic Number	Atomic Type	Coordinates (Angstroms) X	Y	Z
1	6	0	-6.732761	3.213373	1.055813
2	6	0	-6.698306	3.329840	-0.268182
3	6	0	-7.072830	2.237401	-1.236995
4	6	0	-5.855011	1.676681	-1.990912
5	6	0	-4.541190	2.061300	-1.754112
6	6	0	-3.773835	3.125633	-0.990973
7	6	0	-2.468935	1.077388	-1.104336
8	6	0	-1.958990	-0.165387	-0.766397
9	6	0	-2.314285	-1.356078	-1.536794
10	6	0	-2.373247	-2.712010	-0.957878
11	6	0	-2.325090	-2.884360	0.498241
12	6	0	-2.116818	-1.725657	1.356875
13	6	0	-2.843395	-1.606660	2.525081
14	6	0	-3.422212	-0.340273	3.142081
15	6	0	-4.907097	-0.709456	3.363645
16	6	0	-6.065348	-0.038463	2.992878
17	6	0	-6.070075	1.431716	2.715564
18	6	0	-7.195918	1.989236	1.794410
19	6	0	-7.680649	0.913970	0.852128
20	6	0	-7.677518	1.039145	-0.515163
21	6	0	-8.053215	-0.087742	-1.340225
22	6	0	-7.313944	-0.341386	-2.571299
23	6	0	-6.112112	0.475186	-2.789965
24	6	0	-5.032053	-0.176402	-3.402043
25	6	0	-3.713870	0.087103	-2.925290
26	6	0	-3.520629	1.111595	-2.068518
27	6	0	-3.008790	-1.145101	-2.738636
28	6	0	-3.791889	-2.149884	-3.389794
29	6	0	-5.047513	-1.553428	-3.803955
30	6	0	-6.213441	-2.272061	-3.713310
31	6	0	-7.370937	-1.644666	-3.102793
32	6	0	-8.103522	-2.710611	-2.450549
33	6	0	-8.788685	-2.454775	-1.283059
34	6	0	-8.741224	-1.128862	-0.726198
35	6	0	-8.724660	-1.259055	0.712010
36	6	0	-8.031876	-0.337171	1.480086
37	6	0	-7.241707	-0.808394	2.618300
38	6	0	-7.279001	-2.179730	2.902662
39	6	0	-6.091061	-2.860109	3.334627
40	6	0	-4.927534	-2.130602	3.458688
41	6	0	-3.688630	-2.670433	2.944297
42	6	0	-3.672579	-3.891676	2.319236
43	6	0	-2.969890	-3.998248	1.065259
44	6	0	-3.670153	-4.977007	0.280522
45	6	0	-3.688676	-4.836114	-1.087456
46	6	0	-3.010021	-3.723447	-1.708363
47	6	0	-3.753079	-3.436913	-2.925940
48	6	0	-4.953574	-4.239288	-2.954984
49	6	0	-6.168286	-3.667756	-3.338895
50	6	0	-7.362594	-3.955468	-2.582303
51	6	0	-7.326271	-4.863425	-1.530048
52	6	0	-8.070102	-4.596800	-0.313235
53	6	0	-8.790562	-3.415690	-0.200887
54	6	0	-8.758381	-2.665616	1.030624
55	6	0	-8.041268	-3.123423	2.115291
56	6	0	-7.294756	-4.362832	2.013355
57	6	0	-6.069993	-4.190539	2.758281
58	6	0	-4.876618	-4.694897	2.253102
59	6	0	-4.865054	-5.393741	0.992430
60	6	0	-6.049721	-5.607770	0.297532
61	6	0	-6.066862	-5.474683	-1.147896
62	6	0	-4.903981	-5.135071	-1.826808
63	6	0	-7.292657	-5.073046	0.815081
64	6	0	-8.373496	2.374545	2.710955
65	6	0	-9.438456	3.117353	2.196683
66	6	0	-10.498986	3.419038	3.039579
67	6	0	-10.460673	2.970405	4.357306
68	6	0	-9.355963	2.235365	4.769829
69	6	0	-8.054130	2.861784	-2.241240
70	6	0	-9.436043	2.724992	-2.110371
71	6	0	-10.255474	3.374746	-3.026858
72	6	0	-9.670946	4.136735	-4.030869
73	6	0	-8.281675	4.220422	-4.066410
74	16	0	-1.112073	-0.373077	0.779888
75	7	0	-8.329543	1.938510	3.967926
76	7	0	-7.484397	3.603153	-3.193888
77	8	0	-2.846238	0.681312	3.380846
78	8	0	-4.028386	4.291072	-0.781199
79	8	0	-5.253882	2.176527	3.208282
80	6	0	-2.453701	2.456905	-0.565953
81	8	0	-3.022453	2.418005	1.256392
82	15	0	-2.689706	3.664259	2.113840
83	6	0	-4.029879	4.858964	2.328775
84	6	0	-1.350008	4.659200	1.376909
85	6	0	-2.121751	3.284874	3.784414
86	1	0	-6.474440	4.063640	1.675962
87	1	0	-6.376888	4.258982	-0.727455
88	1	0	-9.419040	3.445086	1.161670
89	1	0	-11.343037	3.996384	2.675723
90	1	0	-11.266492	3.183436	5.050299
91	1	0	-9.285378	1.863976	5.789032
92	1	0	-9.857054	2.114981	-1.318135
93	1	0	-11.334632	3.281512	-2.958329
94	1	0	-10.271249	4.655094	-4.769688
95	1	0	-7.783791	4.810211	-4.832332
96	1	0	-1.570983	2.979407	-0.252688
97	1	0	-3.622838	5.746757	2.820886
98	1	0	-4.429429	5.129578	1.350765
99	1	0	-4.800097	4.406307	2.951725
100	1	0	-0.442236	4.064031	1.245078
101	1	0	-1.680964	5.063982	0.416571
102	1	0	-1.122701	5.490502	2.048640

103	1	0	-2.942171	2.826325	4.335866
104	1	0	-1.291708	2.579082	3.745055
105	1	0	-1.809924	4.220702	4.256657
106	15	0	-1.666126	3.523020	-2.811537
107	6	0	-1.016164	5.217471	-2.527389
108	6	0	-2.900173	3.729868	-4.145542
109	6	0	-0.303293	2.558873	-3.533546
110	1	0	-0.175864	5.180955	-1.829083
111	1	0	-1.810278	5.836683	-2.100943
112	1	0	-0.675045	5.664087	-3.465796
113	1	0	-2.434855	4.164629	-5.033720
114	1	0	-3.268806	2.743084	-4.436024
115	1	0	-3.730430	4.341459	-3.780299
116	1	0	-0.743410	1.673927	-4.001888
117	1	0	0.400121	2.247242	-2.756806
118	1	0	0.228622	3.113015	-4.313084
119	6	0	4.693644	3.680815	-0.628210
120	6	0	4.625033	3.636599	0.697301
121	6	0	5.575576	2.871971	1.578724
122	6	0	4.898373	1.627699	2.176576
123	6	0	3.629603	1.193023	1.839400
124	6	0	2.455108	1.611245	0.936864
125	6	0	2.507735	-0.730504	1.024543
126	6	0	2.720015	-2.086928	0.689451
127	6	0	3.716818	-2.873532	1.370695
128	6	0	4.544712	-3.892678	0.699420
129	6	0	4.605148	-3.899975	-0.773184
130	6	0	3.748087	-3.003527	-1.540283
131	6	0	4.265347	-2.324619	-2.628219
132	6	0	3.999800	-0.862221	-3.010809
133	6	0	5.428041	-0.314420	-3.279036
134	6	0	6.012156	0.863674	-2.835499
135	6	0	5.193198	2.064146	-2.468127
136	6	0	5.799526	3.064271	-1.437658
137	6	0	6.828509	2.372553	-0.575517
138	6	0	6.762763	2.329506	0.795383
139	6	0	7.712956	1.536321	1.542303
140	6	0	7.252454	0.781059	2.704506
141	6	0	5.805172	0.729538	2.915781
142	6	0	5.298849	-0.476155	3.414329
143	6	0	4.067278	-0.976487	2.884553
144	6	0	3.344214	-0.190603	2.070768
145	6	0	4.198529	-2.385567	2.607825
146	6	0	5.417531	-2.801229	3.200373
147	6	0	6.106724	-1.622361	3.711511
148	6	0	7.472162	-1.530637	3.626978
149	6	0	8.052844	-0.303824	3.129492
150	6	0	9.260826	-0.688966	2.428381
151	6	0	9.669055	0.025877	1.323545
152	6	0	8.869341	1.137764	0.879160
153	6	0	8.921551	1.165208	-0.563378
154	6	0	7.822388	1.603799	-1.286984
155	6	0	7.425242	0.870783	-2.489515
156	6	0	8.229730	-0.205438	-2.883487
157	6	0	7.631612	-1.396374	-3.416201
158	6	0	6.255301	-1.452548	-3.506672
159	6	0	5.562407	-2.652045	-3.102987
160	6	0	6.262866	-3.726448	-2.603868
161	6	0	5.773191	-4.354646	-1.403393
162	6	0	6.923390	-4.815439	-0.672758
163	6	0	6.875644	-4.832214	0.700909
164	6	0	5.677389	-4.393273	1.393372
165	6	0	6.127959	-3.847795	2.653891
166	6	0	7.578483	-3.792483	2.660145
167	6	0	8.242623	-2.665025	3.140496
168	6	0	9.374323	-2.139970	2.421361
169	6	0	9.865823	-2.798525	1.296872
170	6	0	10.306477	-2.036029	0.143968
171	6	0	10.215224	-0.650555	0.166700
172	6	0	9.746372	0.063389	-0.996775
173	6	0	9.405779	-0.615164	-2.147649
174	6	0	9.503455	-2.062069	-2.184577
175	6	0	8.387652	-2.547893	-2.962897
176	6	0	7.710294	-3.692540	-2.557253
177	6	0	8.127031	-4.394271	-1.367364
178	6	0	9.228057	-3.957093	-0.642184
179	6	0	9.186463	-3.980348	0.808717
180	6	0	8.048108	-4.442354	1.463252
181	6	0	9.930677	-2.758528	-1.056544
182	6	0	6.527765	4.160095	-2.237279
183	6	0	6.800942	5.395417	-1.644766
184	6	0	7.501944	6.341611	-2.379593
185	6	0	7.905171	6.026294	-3.673731
186	6	0	7.587101	4.767536	-4.170631
187	6	0	6.030971	3.824300	2.692011
188	6	0	7.161410	4.630960	2.530023
189	6	0	7.493498	5.525833	3.537807
190	6	0	6.687429	5.591956	4.669832
191	6	0	5.577674	4.756513	4.729442
192	16	0	2.143882	-2.618992	-0.905240
193	7	0	6.915917	3.848715	-3.473158
194	7	0	5.246966	3.891461	3.769173
195	8	0	2.956924	-0.275915	-2.994323
196	8	0	1.992066	2.749601	0.752180
197	8	0	4.117075	2.282156	-2.964859
198	6	0	1.893916	0.384333	0.426679
199	8	0	1.016668	0.327682	-0.609322
200	1	0	3.916434	4.180520	-1.198189
201	1	0	3.784502	4.091011	1.209209
202	1	0	6.458629	5.595029	-0.634523
203	1	0	7.729638	7.312021	-1.949277
204	1	0	8.452310	6.734989	-4.285062
205	1	0	7.883326	4.477695	-5.175937
206	1	0	7.764587	4.545232	1.631766
207	1	0	8.369483	6.159887	3.442037
208	1	0	6.908542	6.271374	5.485252
209	1	0	4.918573	4.780718	5.594949
210	1	0	1.052106	2.635851	0.829870

The total electronic energy was calculated to be –8189.55404299 Hartree..

An imaginary frequency was found at 849.7101 cm^{-1}.

Supplementary Table 50. Optimized structure of **TS3-A4^{-}-S$_N$2-#1** (M06-2X/6-31G(d,p))

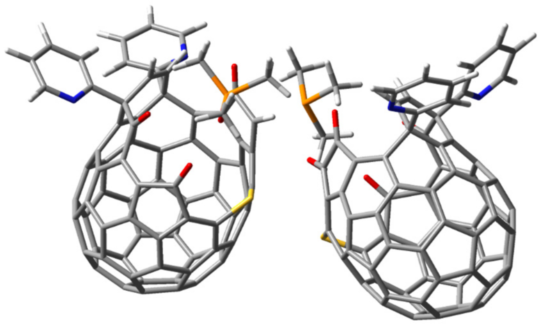

Standard orientation:

Center Number	Atomic Number	Atomic Type	X	Y	Z
1	6	0	6.278254	3.469130	-0.709712
2	6	0	5.994639	3.462464	0.587224
3	6	0	6.406245	2.378412	1.549180
4	6	0	5.212618	1.584358	2.093884
5	6	0	3.897214	1.780123	1.703389
6	6	0	3.065874	2.764701	0.877964
7	6	0	2.080568	0.545994	0.796250
8	6	0	1.829681	-0.729202	0.318659
9	6	0	2.281938	-1.902206	1.069927
10	6	0	2.650719	-3.180147	0.436128
11	6	0	2.841890	-3.226793	-1.020330
12	6	0	2.575973	-2.034948	-1.817088
13	6	0	3.431015	-1.715132	-2.853349
14	6	0	3.886762	-0.329510	-3.312733
15	6	0	5.429862	-0.475184	-3.386445
16	6	0	6.433091	0.325612	-2.862885
17	6	0	6.223062	1.777957	-2.567319
18	6	0	7.066527	2.406091	-1.417324
19	6	0	7.552387	1.338320	-0.468152
20	6	0	7.313371	1.352654	0.883413
21	6	0	7.749183	0.245441	1.704105
22	6	0	6.898796	-0.203173	2.800967
23	6	0	5.560009	0.395980	2.881530
24	6	0	4.534890	-0.448155	3.326746
25	6	0	3.256130	-0.358958	2.703145
26	6	0	3.001544	0.676631	1.873692
27	6	0	2.795193	-1.670019	2.355907
28	6	0	3.644510	-2.582512	3.056180
29	6	0	4.724899	-1.827820	3.664517
30	6	0	5.993093	-2.349530	3.701790
31	6	0	7.101336	-1.511616	3.284276
32	6	0	8.078606	-2.405824	2.698051
33	6	0	8.863640	-1.969540	1.653435
34	6	0	8.680040	-0.631510	1.155776
35	6	0	8.893980	-0.661580	-0.271578
36	6	0	8.186314	0.201955	-1.092564
37	6	0	7.644532	-0.299484	-2.354749
38	6	0	7.919235	-1.630593	-2.689354
39	6	0	6.906105	-2.439077	-3.306691
40	6	0	5.670607	-1.871290	-3.536340
41	6	0	4.473584	-2.611253	-3.212885
42	6	0	4.561024	-3.873763	-2.680515
43	6	0	3.727523	-4.179913	-1.547576
44	6	0	4.464366	-5.105629	-0.728263
45	6	0	4.278436	-5.080585	0.632260
46	6	0	3.347773	-4.135794	1.211165
47	6	0	3.878941	-3.821484	2.524110
48	6	0	5.180450	-4.434240	2.683681
49	6	0	6.223692	-3.707964	3.257346
50	6	0	7.539258	-3.756732	2.664635
51	6	0	7.784331	-4.582646	1.573903
52	6	0	8.632864	-4.124205	0.488884
53	6	0	9.166372	-2.843842	0.539499
54	6	0	9.188169	-2.023128	-0.647314
55	6	0	8.700962	-2.505446	-1.843739
56	6	0	8.141936	-3.842461	-1.907695
57	6	0	7.013027	-3.795993	-2.806238
58	6	0	5.854291	-4.499876	-2.496369
59	6	0	5.790919	-5.288688	-1.290775
60	6	0	6.893599	-5.377866	-0.449554
61	6	0	6.701211	-5.351436	0.989070
62	6	0	5.420387	-5.245472	1.516978
63	6	0	8.096217	-4.630344	-0.759778
64	6	0	8.314563	3.039879	-2.064407
65	6	0	9.031491	4.022190	-1.378266
66	6	0	10.173397	4.542652	-1.971831
67	6	0	10.560403	4.065093	-3.220352
68	6	0	9.781875	3.079548	-3.816656
69	6	0	7.113874	3.091593	2.712481
70	6	0	8.495518	3.289873	2.721767
71	6	0	9.054522	4.011137	3.770346
72	6	0	8.220459	4.509998	4.764357
73	6	0	6.854636	4.266737	4.656341
74	16	0	1.259105	-0.930812	-1.350520
75	7	0	8.681596	2.571960	-3.256023
76	7	0	6.304849	3.576937	3.656111
77	8	0	3.200544	0.586909	-3.652445
78	8	0	3.171203	3.964733	0.780589
79	8	0	5.468714	2.462611	-3.215312
80	6	0	1.879686	1.938822	0.347170
81	8	0	3.125216	2.205572	-1.592313
82	15	0	2.695162	3.388889	-2.443785
83	6	0	3.785788	4.842672	-2.364229
84	6	0	1.084917	4.107250	-1.973793
85	6	0	2.506081	3.104905	-4.224035
86	1	0	5.984540	4.314219	-1.318065
87	1	0	5.434324	4.280877	1.029551
88	1	0	8.687555	4.363658	-0.407173
89	1	0	10.752753	5.310438	-1.468720
90	1	0	11.443758	4.443720	-3.721830
91	1	0	10.047689	2.676436	-4.790655
92	1	0	9.112878	2.882556	1.927385
93	1	0	10.126225	4.178428	3.810517
94	1	0	8.613274	5.073844	5.602770
95	1	0	6.166147	4.644814	5.408398
96	1	0	1.100928	2.268048	-0.315319
97	1	0	3.304000	5.687026	-2.864405
98	1	0	3.969030	5.097435	-1.317773

99	1	0	4.719233	4.600211	-2.873554
100	1	0	0.269286	3.397340	-2.126115
101	1	0	1.112907	4.443332	-0.932988
102	1	0	0.886556	4.973178	-2.610997
103	1	0	3.466542	2.807471	-4.643336
104	1	0	1.787432	2.303761	-4.395972
105	1	0	2.150693	4.031984	-4.683251
106	15	0	0.539386	2.617141	2.173890
107	6	0	-0.703734	3.946497	1.955411
108	6	0	1.556592	3.141288	3.598857
109	6	0	-0.418121	1.179023	2.649565
110	1	0	-1.395860	3.667480	1.154150
111	1	0	-0.270743	4.934276	1.783260
112	1	0	-1.274933	3.976761	2.889407
113	1	0	0.883286	3.402564	4.420020
114	1	0	2.208812	2.325979	3.915642
115	1	0	2.169462	4.005109	3.330202
116	1	0	0.226514	0.404468	3.066310
117	1	0	-0.858739	0.788941	1.727505
118	1	0	-1.223358	1.438148	3.340066
119	6	0	-4.528860	3.657780	0.673300
120	6	0	-4.602118	3.683386	-0.653495
121	6	0	-5.623467	2.936956	-1.469318
122	6	0	-5.025220	1.721756	-2.183766
123	6	0	-3.728405	1.268070	-1.963641
124	6	0	-2.574242	1.576831	-1.156730
125	6	0	-2.544947	-0.729950	-1.467186
126	6	0	-2.774317	-2.042697	-1.119668
127	6	0	-3.832482	-2.823301	-1.763507
128	6	0	-4.607084	-3.855959	-1.061671
129	6	0	-4.515477	-3.955094	0.405187
130	6	0	-3.581117	-3.105121	1.133073
131	6	0	-3.980078	-2.512097	2.313631
132	6	0	-3.654757	-1.102839	2.782996
133	6	0	-5.032316	-0.535944	3.176320
134	6	0	-5.621377	0.672936	2.839643
135	6	0	-4.801235	1.859788	2.419857
136	6	0	-5.513408	2.950815	1.565115
137	6	0	-6.644034	2.326304	0.777186
138	6	0	-6.719373	2.362849	-0.593525
139	6	0	-7.751496	1.619021	-1.282796
140	6	0	-7.419120	0.932599	-2.527448
141	6	0	-5.995699	0.875863	-2.876324
142	6	0	-5.567083	-0.298898	-3.514395
143	6	0	-4.291117	-0.838536	-3.132180
144	6	0	-3.471818	-0.111246	-2.354915
145	6	0	-4.412591	-2.255510	-2.910475
146	6	0	-5.708858	-2.618292	-3.400736
147	6	0	-6.420756	-1.406792	-3.779742
148	6	0	-7.778364	-1.310087	-3.565929
149	6	0	-8.278627	-0.114514	-2.932763
150	6	0	-9.419350	-0.521635	-2.133615
151	6	0	-9.704615	0.132309	-0.954261
152	6	0	-8.845804	1.207557	-0.529010
153	6	0	-8.748777	1.151446	0.909939
154	6	0	-7.572732	1.533207	1.542547
155	6	0	-7.065649	0.723797	2.650896
156	6	0	-7.851430	-0.360659	3.068006
157	6	0	-7.230230	-1.590399	3.472264
158	6	0	-5.855210	-1.670785	3.430394
159	6	0	-5.227874	-2.855271	2.893178
160	6	0	-5.989537	-3.885741	2.399875
161	6	0	-5.625377	-4.437906	1.119413
162	6	0	-6.849909	-4.846593	0.482304
163	6	0	-6.937546	-4.777044	-0.887512
164	6	0	-5.808039	-4.307141	-1.662784
165	6	0	-6.373819	-3.671205	-2.831758
166	6	0	-7.823616	-3.622644	-2.707746
167	6	0	-8.504624	-2.458318	-3.051861
168	6	0	-9.556806	-1.967848	-2.195220
169	6	0	-9.938561	-2.687192	-1.066308
170	6	0	-10.252217	-1.988111	0.166873
171	6	0	-10.139912	-0.605393	0.212337
172	6	0	-9.543475	0.034731	1.361059
173	6	0	-9.102776	-0.712497	2.432656
174	6	0	-9.222411	-2.158735	2.398461
175	6	0	-8.046908	-2.705834	3.033827
176	6	0	-7.433813	-3.834045	2.497251
177	6	0	-7.974962	-4.458735	1.315795
178	6	0	-9.132753	-3.963006	0.728721
179	6	0	-9.237042	-3.904280	-0.716939
180	6	0	-8.175537	-4.334192	-1.509338
181	6	0	-9.769810	-2.782408	1.280124
182	6	0	-6.129400	3.963817	2.546596
183	6	0	-6.597648	5.190592	2.068043
184	6	0	-7.186873	6.069246	2.965208
185	6	0	-7.287967	5.697896	4.303609
186	6	0	-6.793600	4.454407	4.678301
187	6	0	-6.188332	3.936492	-2.492927
188	6	0	-7.456801	4.507600	-2.356145
189	6	0	-7.876755	5.437514	-3.299624
190	6	0	-7.019675	5.774440	-4.339712
191	6	0	-5.768486	5.165313	-4.376294
192	16	0	-2.040920	-2.639839	0.388825
193	7	0	-6.225429	3.599016	3.824072
194	7	0	-5.354266	4.270740	-3.479745
195	8	0	-2.586893	-0.560815	2.777684
196	8	0	-1.993803	2.756144	-0.733573
197	8	0	-3.643606	1.992807	2.744148
198	6	0	-1.851327	0.422693	-0.824236
199	8	0	-0.749231	0.472444	-0.185095
200	1	0	-3.717868	4.170134	1.181813
201	1	0	-3.848943	4.214626	-1.229025
202	1	0	-6.492397	5.431827	1.014822
203	1	0	-7.562981	7.029647	2.626724
204	1	0	-7.739727	6.353026	5.039832
205	1	0	-6.853124	4.123402	5.712322
206	1	0	-8.105554	4.214608	-1.538297
207	1	0	-8.862830	5.885646	-3.225752
208	1	0	-7.306453	6.488747	-5.103350
209	1	0	-5.063131	5.405274	-5.169403
210	1	0	-0.567447	1.411129	-0.098929

The total electronic energy was calculated to be –8189.55494455 Hartree..

An imaginary frequency was found at 906.1470 cm^{-1}.

Supplementary Table 51. Optimized structure of **TS3-A4^{-}-S$_{N}$2-#2** (M06-2X/6-31G(d,p))

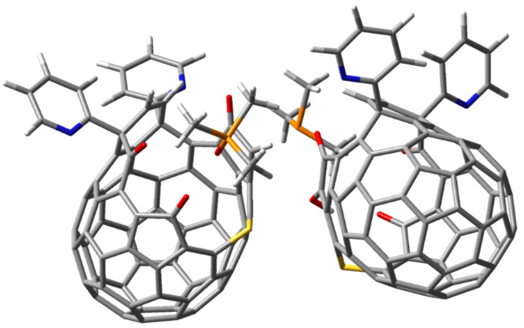

Standard orientation:

Center Number	Atomic Number	Atomic Type	Coordinates (Angstroms) X	Y	Z
1	6	0	-5.568845	3.497934	0.584086
2	6	0	-5.552783	3.405200	-0.741049
3	6	0	-6.241032	2.327186	-1.540047
4	6	0	-5.250252	1.376131	-2.244075
5	6	0	-3.865182	1.444103	-2.132235
6	6	0	-2.814235	2.377526	-1.548187
7	6	0	-2.012357	0.089335	-1.507994
8	6	0	-1.889295	-1.145409	-0.893544
9	6	0	-2.592031	-2.312181	-1.450293
10	6	0	-2.965573	-3.494710	-0.658545
11	6	0	-2.902381	-3.435673	0.807758
12	6	0	-2.389590	-2.238727	1.467254
13	6	0	-2.996831	-1.804921	2.634249
14	6	0	-3.217379	-0.367397	3.091786
15	6	0	-4.728328	-0.340372	3.425379
16	6	0	-5.711220	0.538648	2.995205
17	6	0	-5.387482	1.929702	2.544450
18	6	0	-6.336206	2.581746	1.492806
19	6	0	-7.097388	1.529480	0.723660
20	6	0	-7.105476	1.443130	-0.647817
21	6	0	-7.793641	0.350636	-1.296984
22	6	0	-7.211183	-0.249551	-2.491471
23	6	0	-5.859815	0.191335	-2.860992
24	6	0	-5.026291	-0.790757	-3.415352
25	6	0	-3.650339	-0.808232	-3.051730
26	6	0	-3.119439	0.244677	-2.395354
27	6	0	-3.268773	-2.132752	-2.668824
28	6	0	-4.314637	-2.991242	-3.134997
29	6	0	-5.409725	-2.158945	-3.601456
30	6	0	-6.708116	-2.533240	-3.369557
31	6	0	-7.630168	-1.552500	-2.831111
32	6	0	-8.572027	-2.294976	-2.019413
33	6	0	-9.107944	-1.713721	-0.892152
34	6	0	-8.698407	-0.381093	-0.534026
35	6	0	-8.658259	-0.306619	0.906498
36	6	0	-7.732613	0.513650	1.531464
37	6	0	-7.045628	0.026646	2.729199
38	6	0	-7.396802	-1.244504	3.200217
39	6	0	-6.385947	-2.129947	3.707442
40	6	0	-5.078480	-1.692907	3.703042
41	6	0	-4.032660	-2.572820	3.229219
42	6	0	-4.337179	-3.837582	2.792004
43	6	0	-3.753543	-4.272956	1.549473
44	6	0	-4.703537	-5.163000	0.936309
45	6	0	-4.758041	-5.234805	-0.433452
46	6	0	-3.861132	-4.428813	-1.232647
47	6	0	-4.575596	-4.154961	-2.462846
48	6	0	-5.938758	-4.628833	-2.347868
49	6	0	-6.990887	-3.828192	-2.789191
50	6	0	-8.176870	-3.694189	-1.977935
51	6	0	-8.308830	-4.423865	-0.802914
52	6	0	-8.901250	-3.811840	0.372286
53	6	0	-9.299764	-2.483152	0.318279
54	6	0	-9.026814	-1.601040	1.426867
55	6	0	-8.395268	-2.070080	2.558623
56	6	0	-7.975510	-3.457389	2.625776
57	6	0	-6.710922	-3.489301	3.321111
58	6	0	-5.697780	-4.328910	2.870428
59	6	0	-5.920892	-5.178730	1.727617
60	6	0	-7.157659	-5.195556	1.093729
61	6	0	-7.219340	-5.271283	-0.354279
62	6	0	-6.047255	-5.334690	-1.096794
63	6	0	-8.207911	-4.305859	1.546322
64	6	0	-7.378455	3.401651	2.280006
65	6	0	-8.124850	4.388659	1.632811
66	6	0	-9.082643	5.078628	2.363623
67	6	0	-9.264958	4.759497	3.705915
68	6	0	-8.476565	3.754714	4.254479
69	6	0	-7.080904	3.048052	-2.607081
70	6	0	-8.430011	3.344146	-2.405883
71	6	0	-9.105895	4.060572	-3.386684
72	6	0	-8.415608	4.458177	-4.526000
73	6	0	-7.068925	4.122311	-4.625919
74	16	0	-1.137586	-1.241547	0.711827
75	7	0	-7.552391	3.084327	3.562042
76	7	0	-6.407881	3.435471	-3.693100
77	8	0	-2.414369	0.514848	3.131990
78	8	0	-2.789391	3.586839	-1.522074
79	8	0	-4.456751	2.548165	2.997288
80	6	0	-1.590008	1.535134	-1.236248
81	8	0	-2.047951	1.866863	0.640410
82	15	0	-1.257449	2.973431	1.297780
83	6	0	-2.172382	4.546093	1.425777
84	6	0	0.222291	3.471484	0.349906
85	6	0	-0.604889	2.689747	2.965239
86	1	0	-5.048067	4.309889	1.076441
87	1	0	-5.003806	4.129852	-1.330793
88	1	0	-7.942225	4.605628	0.585117
89	1	0	-9.676623	5.856183	1.893558
90	1	0	-9.999441	5.274338	4.314598
91	1	0	-8.587041	3.471264	5.298228
92	1	0	-8.935145	3.012931	-1.504547
93	1	0	-10.157240	4.300689	-3.263148
94	1	0	-8.903470	5.013932	-5.318546
95	1	0	-6.491548	4.418403	-5.498619
96	1	0	-0.678099	1.783714	-0.787991
97	1	0	-1.504196	5.349882	1.747023
98	1	0	-2.566890	4.781316	0.433646

99	1	0	-2.990369	4.435772	2.139766
100	1	0	0.872393	2.603679	0.200534
101	1	0	-0.097744	3.878936	-0.614587
102	1	0	0.775565	4.242469	0.894255
103	1	0	-1.434018	2.516465	3.651309
104	1	0	0.040240	1.809384	2.956739
105	1	0	-0.028296	3.569267	3.267134
106	15	0	-0.609420	1.880451	-3.023294
107	6	0	0.535395	3.295887	-3.051055
108	6	0	-1.782560	2.302532	-4.354005
109	6	0	0.315528	0.413441	-3.494402
110	1	0	1.422640	3.162988	-2.431986
111	1	0	-0.001374	4.218378	-2.818373
112	1	0	0.898532	3.339423	-4.083378
113	1	0	-1.190835	2.570842	-5.232973
114	1	0	-2.421586	1.453690	-4.597697
115	1	0	-2.402808	3.150575	-4.054603
116	1	0	-0.366833	-0.412313	-3.709313
117	1	0	0.930554	0.146221	-2.626210
118	1	0	0.973343	0.602755	-4.346894
119	6	0	2.995288	3.022742	-0.093019
120	6	0	2.928152	2.698011	1.188274
121	6	0	4.073778	2.180657	1.992431
122	6	0	3.959113	0.668023	2.238762
123	6	0	3.034828	-0.175072	1.641455
124	6	0	1.935253	-0.110128	0.533828
125	6	0	2.874655	-2.270262	0.519482
126	6	0	3.602578	-3.402173	0.112436
127	6	0	4.815843	-3.786114	0.786866
128	6	0	6.027302	-4.232146	0.070647
129	6	0	6.132687	-3.964744	-1.376017
130	6	0	4.991650	-3.407925	-2.092224
131	6	0	5.187617	-2.377734	-2.995940
132	6	0	4.337186	-1.127840	-3.129916
133	6	0	5.373353	0.017728	-3.207748
134	6	0	5.381882	1.250576	-2.558362
135	6	0	4.117339	1.896177	-2.069274
136	6	0	4.242530	2.945943	-0.926923
137	6	0	5.455426	2.624532	-0.084215
138	6	0	5.387488	2.337674	1.256238
139	6	0	6.559236	1.875138	1.958831
140	6	0	6.411615	0.803657	2.938428
141	6	0	5.116515	0.114995	2.961656
142	6	0	5.149451	-1.255445	3.227626
143	6	0	4.268703	-2.122038	2.506839
144	6	0	3.331378	-1.579345	1.702887
145	6	0	4.995265	-3.307563	2.102648
146	6	0	6.258330	-3.250324	2.741278
147	6	0	6.357444	-1.990928	3.464083
148	6	0	7.556599	-1.329173	3.545304
149	6	0	7.580217	0.092316	3.286157
150	6	0	8.861301	0.368718	2.664779
151	6	0	8.968787	1.365588	1.718229
152	6	0	7.791322	2.111436	1.361130
153	6	0	7.858174	2.395690	-0.053589
154	6	0	6.691168	2.454571	-0.804209
155	6	0	6.655468	1.812057	-2.118710
156	6	0	7.851587	1.245422	-2.585884
157	6	0	7.831320	0.015670	-3.323419
158	6	0	6.615109	-0.600624	-3.536106
159	6	0	6.505683	-2.030754	-3.385201
160	6	0	7.590092	-2.777406	-2.989272
161	6	0	7.396811	-3.755879	-1.948498
162	6	0	8.613537	-3.798671	-1.183916
163	6	0	8.535403	-4.056390	0.164810
164	6	0	7.246884	-4.289882	0.793377
165	6	0	7.372743	-3.805268	2.145910
166	6	0	8.655569	-3.145781	2.302338
167	6	0	8.756089	-1.940515	2.992625
168	6	0	9.578259	-0.881865	2.467201
169	6	0	10.339036	-1.078463	1.316810
170	6	0	10.443720	-0.022206	0.327811
171	6	0	9.777445	1.177308	0.533760
172	6	0	9.077518	1.813462	-0.558239
173	6	0	9.079323	1.253520	-1.818032
174	6	0	9.785251	0.005495	-2.046448
175	6	0	9.002954	-0.768356	-2.979923
176	6	0	8.878182	-2.142243	-2.803936
177	6	0	9.530380	-2.793307	-1.694392
178	6	0	10.312769	-2.057928	-0.814248
179	6	0	10.246491	-2.337110	0.608814
180	6	0	9.399074	-3.334781	1.081697
181	6	0	10.440948	-0.625092	-0.991342
182	6	0	4.426782	4.315061	-1.604065
183	6	0	4.546971	5.464315	-0.817882
184	6	0	4.707979	6.685616	-1.454481
185	6	0	4.731070	6.723621	-2.848260
186	6	0	4.599098	5.525615	-3.536709
187	6	0	4.032393	2.936007	3.330666
188	6	0	4.926986	3.953386	3.663406
189	6	0	4.759096	4.616626	4.875181
190	6	0	3.708694	4.249558	5.706474
191	6	0	2.861177	3.231537	5.276405
192	16	0	3.378261	-3.904566	-1.582536
193	7	0	4.458354	4.341237	-2.934138
194	7	0	3.010380	2.590898	4.119357
195	8	0	3.136871	-1.044571	-3.050015
196	8	0	1.163649	0.821149	0.234674
197	8	0	3.044851	1.667789	-2.579941
198	6	0	1.982309	-1.385304	-0.116833
199	8	0	1.239000	-1.634865	-1.242938
200	1	0	2.079734	3.367415	-0.557490
201	1	0	1.982896	2.730195	1.719359
202	1	0	4.506890	5.378890	0.264034
203	1	0	4.807531	7.599081	-0.876463
204	1	0	4.844268	7.658269	-3.385578
205	1	0	4.576523	5.491395	-4.621687
206	1	0	5.742133	4.210072	2.996080
207	1	0	5.443878	5.407824	5.164577
208	1	0	3.544475	4.735349	6.661602
209	1	0	2.019920	2.916769	5.890102
210	1	0	1.863585	-1.822904	-1.962847

The total electronic energy was calculated to be –8189.5615528 Hartree..

An imaginary frequency was found at 783.7094 cm^{-1}.

Supplementary Table 52. Optimized structure of **INT3** (M06-2X/6-31G(d,p))

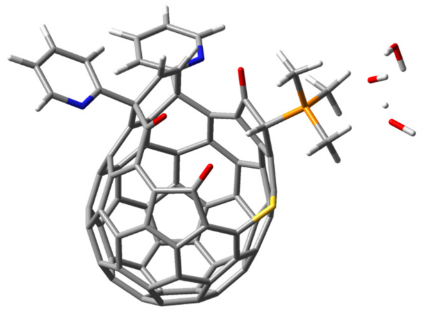

Standard orientation:

Center Number	Atomic Number	Atomic Type	X	Y	Z
1	6	0	2.288467	2.848243	0.704989
2	6	0	2.199444	2.958968	-0.616595
3	6	0	0.931166	2.773569	-1.406752
4	6	0	0.907902	1.434652	-2.160012
5	6	0	1.848845	0.432092	-2.008334
6	6	0	3.128796	0.250683	-1.223446
7	6	0	2.002087	-1.870208	-1.432849
8	6	0	1.225856	-2.974152	-1.175087
9	6	0	-0.043312	-3.203709	-1.835084
10	6	0	-1.179643	-3.827461	-1.136249
11	6	0	-1.106855	-4.016086	0.324930
12	6	0	0.132817	-3.719874	1.034104
13	6	0	0.080013	-3.041277	2.240208
14	6	0	1.015902	-1.922009	2.668578
15	6	0	0.052972	-0.805125	3.137997
16	6	0	0.049785	0.554744	2.837261
17	6	0	1.287609	1.266328	2.370717
18	6	0	1.112758	2.612799	1.613085
19	6	0	-0.195810	2.592933	0.862075
20	6	0	-0.287972	2.713557	-0.501319
21	6	0	-1.559207	2.523777	-1.160233
22	6	0	-1.599116	1.789077	-2.421878
23	6	0	-0.354648	1.118796	-2.827051
24	6	0	-0.488212	-0.124539	-3.457873
25	6	0	0.416524	-1.184977	-3.133954
26	6	0	1.497721	-0.909154	-2.350354
27	6	0	-0.322139	-2.392506	-2.943469
28	6	0	-1.653349	-2.142235	-3.406254
29	6	0	-1.761547	-0.733021	-3.730071
30	6	0	-2.920784	-0.050169	-3.460995
31	6	0	-2.836238	1.240753	-2.806723
32	6	0	-4.019052	1.352258	-1.976595
33	6	0	-3.956183	2.027977	-0.777032
34	6	0	-2.700737	2.599777	-0.369701
35	6	0	-2.595545	2.451336	1.063998
36	6	0	-1.354940	2.248630	1.648961
37	6	0	-1.223641	1.251287	2.714475
38	6	0	-2.395567	0.617647	3.146989
39	6	0	-2.376805	-0.770145	3.511962
40	6	0	-1.185142	-1.457758	3.416821
41	6	0	-1.174681	-2.788870	2.853934
42	6	0	-2.333785	-3.348712	2.372881
43	6	0	-2.294912	-3.969176	1.070851
44	6	0	-3.586332	-3.762003	0.471228
45	6	0	-3.666151	-3.617131	-0.893961
46	6	0	-2.464329	-3.676229	-1.701243
47	6	0	-2.708354	-2.808701	-2.837899
48	6	0	-3.970697	-2.121872	-2.656928
49	6	0	-4.075996	-0.765667	-2.959484
50	6	0	-4.779015	0.115887	-2.057707
51	6	0	-5.413492	-0.393419	-0.930619
52	6	0	-5.352756	0.330452	0.326421
53	6	0	-4.639966	1.518677	0.393317
54	6	0	-3.788872	1.786786	1.527481
55	6	0	-3.692971	0.884010	2.564488
56	6	0	-4.447715	-0.353397	2.513172
57	6	0	-3.621563	-1.386485	3.093117
58	6	0	-3.595687	-2.652602	2.520745
59	6	0	-4.391192	-2.931866	1.350202
60	6	0	-5.219093	-1.954110	0.814197
61	6	0	-5.316966	-1.808089	-0.626740
62	6	0	-4.583142	-2.643161	-1.460476
63	6	0	-5.246418	-0.631758	1.406525
64	6	0	1.054035	3.733967	2.665084
65	6	0	1.204938	5.065613	2.273795
66	6	0	1.117250	6.053203	3.245548
67	6	0	0.885572	5.679896	4.566108
68	6	0	0.749805	4.326412	4.852354
69	6	0	0.842979	3.941488	-2.400258
70	6	0	0.188579	5.129673	-2.070134
71	6	0	0.207223	6.172892	-2.987984
72	6	0	0.880092	5.997357	-4.192198
73	6	0	1.513317	4.779116	-4.418200
74	16	0	1.682449	-3.972361	0.205268
75	7	0	0.829937	3.367488	3.926503
76	7	0	1.502122	3.770921	-3.545322
77	8	0	2.216050	-1.898187	2.567772
78	8	0	4.090538	0.970552	-1.160184
79	8	0	2.397442	0.841672	2.591513
80	6	0	3.023140	-1.166011	-0.557855
81	15	0	4.688462	-1.732023	0.012858
82	6	0	4.561683	-3.352279	0.813296
83	6	0	5.153938	-0.531091	1.304401
84	6	0	5.822773	-1.558559	-1.383534
85	1	0	3.253636	2.939632	1.194332
86	1	0	3.091540	3.129808	-1.210458
87	1	0	1.394829	5.306340	1.232296
88	1	0	1.232090	7.098653	2.977415
89	1	0	0.812376	6.417652	5.356883
90	1	0	0.568128	3.991135	5.870404
91	1	0	-0.331966	5.221730	-1.122114
92	1	0	-0.298555	7.107559	-2.767073
93	1	0	0.918332	6.781812	-4.939367
94	1	0	2.056229	4.600622	-5.343385
95	1	0	2.542529	-0.980469	0.418312
96	1	0	5.024494	-4.109690	0.187837
97	1	0	3.511084	-3.576565	1.005057
98	1	0	5.097483	-3.247705	1.762121
99	1	0	5.259219	0.463058	0.874153
100	1	0	4.382700	-0.515773	2.079754
101	1	0	6.103862	-0.848869	1.742433
102	1	0	5.540139	-0.691942	-1.982767

103	1	0	5.749446	-2.467535	-1.973467
104	1	0	6.825948	-1.434582	-0.967433
105	8	0	7.648808	-2.750632	1.056307
106	1	0	7.853024	-3.600985	1.456323
107	8	0	9.150530	-0.824059	-0.134746
108	1	0	9.059971	-1.226068	-1.013142
109	1	0	8.795915	-1.567175	0.406811
110	8	0	8.008746	-3.008294	-1.466282
111	1	0	8.640006	-3.715076	-1.623942
112	1	0	7.829364	-3.013018	-0.460455

The total electronic energy was calculated to be –4248.1721918 Hartree..

11.10. Formation of 3a' by the Deprotonation of INT3

The final step for the formation of **3a'** by the reaction of **1'** with PMe_3 should be the deprotonation of **INT3**. In this step, a hydrogen atom on the carbon atom bearing a phosphonium substituent should be abstracted by a bulkiness anion such as OH^- which is considered to be formed at the second step in this sequential reaction mechanism. The required energy for this step was calculated to be $\Delta G^{\ddagger}$ +5.9 kcal/mol (Supplementary Fig. 50).

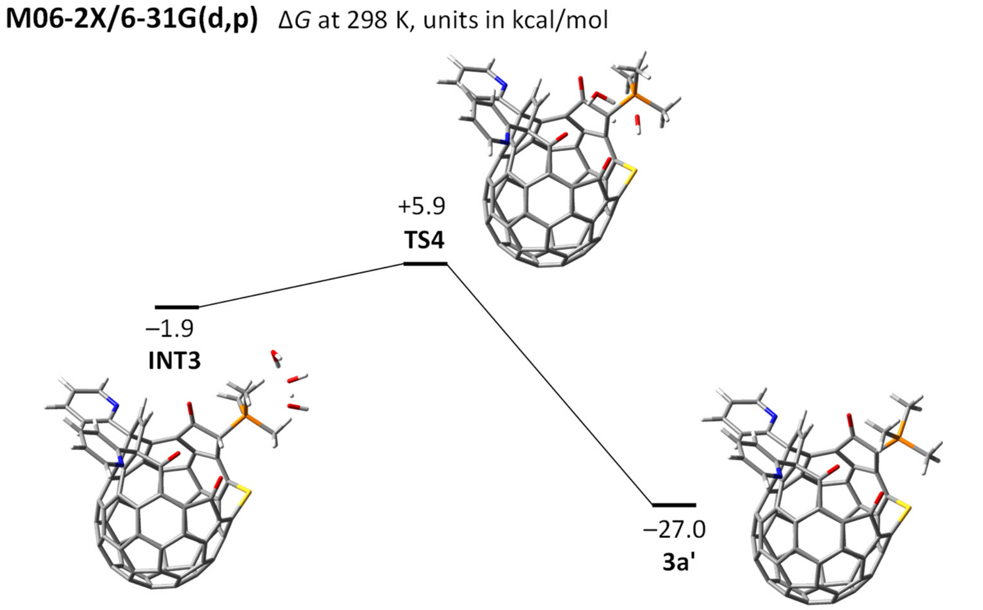

Supplementary Fig. 50. Formation of **3a'** by the deprotonation of **INT3** (M06-2X/6-31G(d,p)).

Supplementary Table 53. Optimized structure of **TS4** (M06-2X/6-31G(d,p))

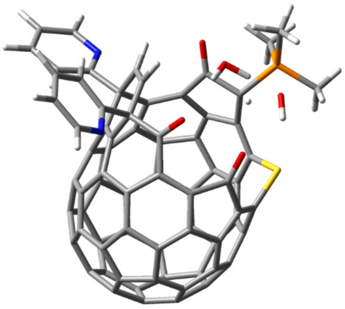

Standard orientation:

Center Number	Atomic Number	Atomic Type	Coordinates (Angstroms) X	Y	Z
1	6	0	3.370108	-1.079713	-0.685495
2	6	0	3.401056	-1.053108	0.643419
3	6	0	2.274726	-1.486299	1.545046
4	6	0	1.655977	-0.255880	2.235024
5	6	0	2.070640	1.042586	1.990012
6	6	0	3.178435	1.759047	1.229071
7	6	0	1.169363	3.055273	1.145033

8	6	0	-0.041764	3.548997	0.683487
9	6	0	-1.298231	3.249519	1.363442
10	6	0	-2.589667	3.142885	0.655559
11	6	0	-2.616416	3.078615	-0.818206
12	6	0	-1.383250	3.209251	-1.586796
13	6	0	-1.167489	2.420671	-2.699126
14	6	0	0.148250	1.791391	-3.186262
15	6	0	-0.250709	0.318639	-3.409278
16	6	0	0.370537	-0.828745	-2.946259
17	6	0	1.842925	-0.935032	-2.684563
18	6	0	2.306905	-1.796415	-1.473275
19	6	0	1.137963	-2.230350	-0.612299
20	6	0	1.139703	-2.133881	0.760178
21	6	0	-0.042575	-2.478944	1.517795
22	6	0	-0.399146	-1.687269	2.692432
23	6	0	0.393349	-0.476287	2.937633
24	6	0	-0.299114	0.627966	3.453794
25	6	0	0.013014	1.928051	2.952431
26	6	0	1.130568	2.099553	2.203971
27	6	0	-1.197811	2.610031	2.610063
28	6	0	-2.261978	1.856758	3.201473
29	6	0	-1.706387	0.622797	3.726945
30	6	0	-2.414506	-0.547456	3.624896
31	6	0	-1.739859	-1.729926	3.122565
32	6	0	-2.747757	-2.499300	2.424457
33	6	0	-2.394921	-3.240651	1.318730
34	6	0	-1.028845	-3.213186	0.866135
35	6	0	-1.039349	-3.293024	-0.573805
36	6	0	-0.054891	-2.653395	-1.312802
37	6	0	-0.442233	-1.960353	-2.541762
38	6	0	-1.788315	-2.003145	-2.923497
39	6	0	-2.417421	-0.834189	-3.469680
40	6	0	-1.661797	0.312977	-3.591245
41	6	0	-2.212137	1.578043	-3.166154
42	6	0	-3.481799	1.644376	-2.647441
43	6	0	-3.681804	2.410206	-1.443512
44	6	0	-4.738607	1.764231	-0.710883
45	6	0	-4.727397	1.825755	0.660731
46	6	0	-3.669254	2.541608	1.343235
47	6	0	-3.499111	1.864819	2.615372
48	6	0	-4.309316	0.664754	2.637005
49	6	0	-3.773784	-0.524704	3.127542
50	6	0	-3.999466	-1.759154	2.413779
51	6	0	-4.816254	-1.779935	1.289772
52	6	0	-4.453649	-2.590312	0.141297
53	6	0	-3.266627	-3.310087	0.166033
54	6	0	-2.418641	-3.352186	-1.000833
55	6	0	-2.793115	-2.707611	-2.160419
56	6	0	-4.028738	-1.948226	-2.196351
57	6	0	-3.784802	-0.767935	-2.991117
58	6	0	-4.309861	0.455745	-2.590460
59	6	0	-5.111618	0.538925	-1.394983
60	6	0	-5.397322	-0.604319	-0.658545
61	6	0	-5.388122	-0.543059	0.791391
62	6	0	-5.103411	0.655035	1.434998
63	6	0	-4.833804	-1.877374	-1.062226
64	6	0	2.893933	-3.088417	-2.092843
65	6	0	3.981038	-3.752364	-1.525643
66	6	0	4.412656	-4.935470	-2.116493
67	6	0	3.751139	-5.412635	-3.241326
68	6	0	2.672692	-4.678410	-3.726176
69	6	0	2.832518	-2.462444	2.587833
70	6	0	3.004689	-3.813064	2.270455
71	6	0	3.571939	-4.650539	3.220357
72	6	0	3.950157	-4.115465	4.448726
73	6	0	3.744156	-2.757678	4.662367
74	16	0	-0.077581	4.229296	-0.962585
75	7	0	2.248487	-3.546101	-3.168119
76	7	0	3.198738	-1.940719	3.757639
77	8	0	1.220948	2.309154	-3.297240
78	8	0	4.371385	1.591377	1.317068
79	8	0	2.650047	-0.481263	-3.458064
80	6	0	2.507673	2.917615	0.497184
81	15	0	3.639816	4.331057	0.461893
82	6	0	2.969026	5.656942	-0.550817
83	6	0	5.274756	3.839703	-0.177241
84	6	0	3.971094	4.884744	2.170620
85	1	0	4.146879	-0.586836	-1.262163
86	1	0	4.240995	-0.594757	1.154242
87	1	0	4.474746	-3.343236	-0.651882
88	1	0	5.260136	-5.473698	-1.703314
89	1	0	4.058687	-6.327809	-3.734194
90	1	0	2.123676	-5.013329	-4.602922
91	1	0	2.695284	-4.187124	1.298797
92	1	0	3.714051	-5.705419	3.007127
93	1	0	4.392859	-4.731981	5.222752
94	1	0	4.028190	-2.298023	5.606061
95	1	0	2.567525	2.752948	-0.841398
96	1	0	2.487271	6.399226	0.090591
97	1	0	2.263886	5.159613	-1.216805
98	1	0	3.792427	6.119059	-1.098722
99	1	0	5.670004	3.010963	0.416206
100	1	0	5.168591	3.526891	-1.214945
101	1	0	5.953502	4.693370	-0.109196
102	1	0	3.347849	5.744774	2.419897
103	1	0	5.023410	5.167062	2.249172
104	1	0	3.780112	4.083760	2.884156
105	8	0	2.979960	3.282886	-2.079408
106	1	0	2.197689	3.650138	-2.501465
107	8	0	4.754772	1.832566	-3.359910
108	1	0	5.204618	2.457273	-3.916577
109	1	0	3.956711	2.575100	-2.615503

The total electronic energy was calculated to be –4171.7567242 Hartree..

An imaginary frequency was found at 265.7970 cm^{-1}.

Supplementary Note 3

12. NBO Analyses of 2 and 3d

Prior to natural bond orbital (NBO) analyses, two different conformations of **2**, i.e., **2A** and **2B**, were calculated at the B3LYP/6-31G(d) level of theory. Conformation **2A** is suggested to be more stable by Δ*G* –4.3 kcal/mol (298 K) relative to **2B** (Supplementary Fig. 51). Thus, the geometry of **2A** was adopted for the further analyses.

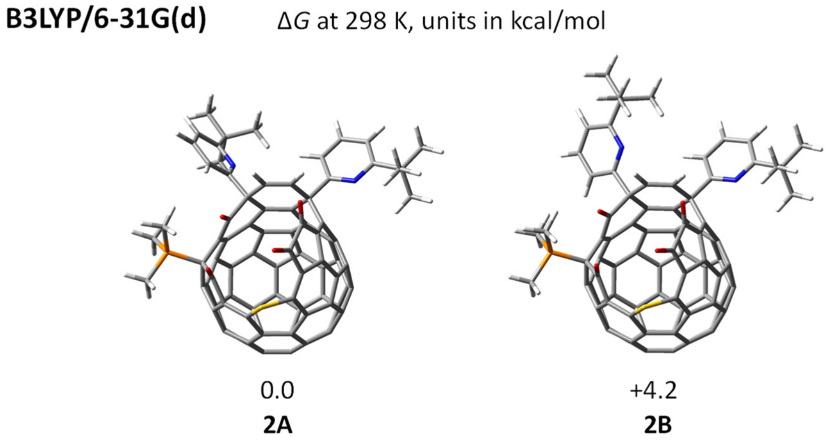

Supplementary Table 51. Optimized structures of **2A** and **2B** (B3LYP/6-31G(d)).

The structures of **2A** and **3d** were optimized at the M06-2X/6-31G(d,p) level of theory and the NBO analyses were performed at the same level of theory. The natural charges on the O and C(β) atoms in **2** are negatively charged while the C(γ) atom has positive charge, indicating the charge delocalization along with an O–C(γ)–C(β) moiety by the possible resonance structures (Supplementary Fig. 52a). The Wiberg bond indices support the delocalization of the anionic charge in **2** since the double bond character in the O–C(γ) bond is weakened while double bond character in the C(γ)–C(β) bond increases (Supplementary Fig. 52b). In the case of β-oxo-phosphorus ylide **3d**, natural charges suggest the $O^{\delta-}$–$C(\gamma)^{\delta+}$–$C(\beta)^{\delta-}$–P^{+} character with weakened double bond character in the O–C(γ) bond and strengthened double bond character in the C(γ)–C(β) bond (Supplementary Fig. 52a,b).

Compared with the bond order of the P–C bond in **2**, the corresponding index in **3d** was suggested to be increased by 19%, indicative of the slightly stronger double bond character in **3d** (though it is considered to be nearly pure single bond). This results are in line with the bond lengths observed in crystal structures: 1.838(6) Å for **2** and 1.738(3) Å in **3d** (Supplementary Fig. 52c).

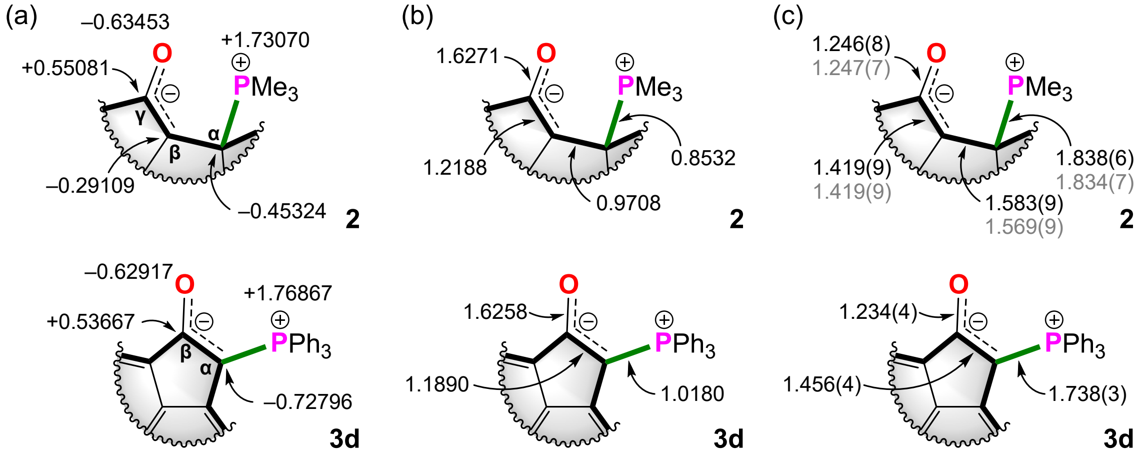

Supplementary Fig. 52. (a) Natural charges and (b) Wiberg bond order of **2** and **3d** (M06-2X/6-31G(d,p)). (c) Selected bond lengths (unit in Å) from single crystal X-ray structures of **2** and **3d**. Since the X-ray structure of **2** contains two independent molecules, two sets of bond lengths are shown.

Supplementary Table 54. Optimized structure of **2A** (B3LYP/6-31G(d))

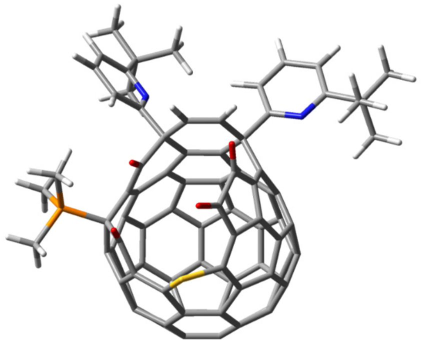

Standard orientation:

Center Number	Atomic Number	Atomic Type	Coordinates (Angstroms) X	Y	Z
1	6	0	2.527773	1.705324	1.729636
2	6	0	3.107492	0.511649	1.655083
3	6	0	2.825938	-0.505916	0.583449
4	6	0	1.981564	-1.691152	1.073424
5	6	0	1.410454	-1.764616	2.328833
6	6	0	1.421961	-0.962739	3.601528
7	6	0	-0.047828	-1.073745	4.191434
8	6	0	-0.733984	-2.086740	3.371369
9	6	0	-2.059705	-2.308116	3.067399
10	6	0	-2.446882	-3.153492	1.966886
11	6	0	-3.646129	-2.919519	1.158366
12	6	0	-4.341847	-1.622917	1.282788
13	6	0	-3.913673	-0.654617	2.280114
14	6	0	-3.821644	0.688133	1.954765
15	6	0	-2.850908	1.706070	2.476411
16	6	0	-2.447537	2.636420	1.220669
17	6	0	-0.999019	2.486594	0.600790
18	6	0	0.136654	2.420014	1.449205
19	6	0	1.526900	2.229274	0.762021
20	6	0	1.359662	1.308371	-0.408167
21	6	0	1.982443	0.103023	-0.535924
22	6	0	1.644083	-0.765620	-1.633050
23	6	0	1.487603	-2.189479	-1.378440
24	6	0	1.520429	-2.605665	0.021487
25	6	0	0.653353	-3.657548	0.372862
26	6	0	-0.052613	-3.581042	1.622520
27	6	0	0.255159	-2.614972	2.508465
28	6	0	-1.429104	-3.931164	1.396411
29	6	0	-1.498719	-4.502832	0.089423
30	6	0	-2.650671	-4.358612	-0.627109
31	6	0	-3.750001	-3.578906	-0.094076
32	6	0	-4.431889	-3.012222	-1.215165
33	6	0	-5.045905	-1.794832	-1.110734
34	6	0	-4.997380	-1.096180	0.152791
35	6	0	-5.001696	0.304959	-0.136859
36	6	0	-4.282348	1.141653	0.704114
37	6	0	-3.474922	2.199118	0.196584
38	6	0	-3.273681	2.277810	-1.163688
39	6	0	-1.949777	2.452895	-1.714967
40	6	0	-0.858240	2.365484	-0.823529
41	6	0	0.346729	1.717565	-1.339312
42	6	0	0.338132	1.096623	-2.586255
43	6	0	0.988750	-0.193375	-2.723477
44	6	0	0.215461	-0.952669	-3.652361
45	6	0	0.087186	-2.308604	-3.452603
46	6	0	0.729567	-2.931682	-2.308712
47	6	0	-0.146226	-4.009390	-1.884100
48	6	0	-0.208998	-4.320045	-0.545288
49	6	0	-1.349503	-3.972079	-2.680616
50	6	0	-2.591882	-4.157072	-2.060787
51	6	0	-3.719236	-3.338788	-2.436310
52	6	0	-3.585171	-2.383319	-3.447828
53	6	0	-4.230372	-1.092610	-3.310172
54	6	0	-4.970413	-0.816728	-2.172618
55	6	0	-4.920198	0.485191	-1.560617
56	6	0	-4.050753	1.451650	-2.064739
57	6	0	-3.232079	1.159567	-3.222277
58	6	0	-1.938094	1.767869	-2.989886
59	6	0	-0.799947	1.127628	-3.446563
60	6	0	-0.902528	-0.157113	-4.106630
61	6	0	-2.145475	-0.754306	-4.321644
62	6	0	-2.282437	-2.173835	-4.081114
63	6	0	-3.334152	-0.083497	-3.853159
64	6	0	2.047063	3.570036	0.194940
65	6	0	-1.194184	-2.943019	-3.688820
66	6	0	1.478842	4.798898	0.459567
67	6	0	2.010855	5.916531	-0.162609
68	6	0	3.106125	5.781360	-0.975312
69	6	0	3.653974	4.513824	-1.168651
70	6	0	4.875011	4.235833	-2.037320
71	6	0	5.934246	3.576008	-1.124739
72	6	0	5.449414	5.481822	-2.658072
73	6	0	4.487969	3.235427	-3.096758
74	6	0	4.176770	-1.012317	0.057673
75	6	0	4.882513	-0.282592	-0.877556
76	6	0	6.136423	-0.759523	-1.230406
77	6	0	6.621341	-1.889707	-0.662602
78	6	0	5.868261	-2.543379	0.299175
79	6	0	6.338447	-3.780750	1.086158
80	6	0	7.752551	-4.107839	0.854327
81	6	0	6.215187	-3.413295	2.707820
82	6	0	5.397976	-4.863273	0.860129
83	6	0	-1.757806	4.920630	3.033295
84	6	0	-2.477390	5.391000	0.249143
85	6	0	-4.490403	4.536406	2.167476
86	16	0	-3.260835	-1.237741	3.819796
87	15	0	-2.773873	4.383642	1.689710
88	8	0	-2.497027	1.866908	3.616477
89	8	0	0.125632	2.554820	2.687689
90	8	0	-0.409098	-0.461406	5.165063
91	8	0	2.307439	-0.448311	4.216214
92	7	0	3.118104	3.430796	-0.589927
93	7	0	4.642903	-2.109074	0.627938
94	1	0	2.770542	2.267158	2.457727
95	1	0	3.741284	0.286125	2.324541
96	1	0	0.740557	4.877499	1.054346
97	1	0	1.617723	6.770270	-0.025172
98	1	0	3.487400	6.540091	-1.400681
99	1	0	6.214589	4.211495	-0.434490
100	1	0	6.711303	3.313448	-1.661708

101	1	0	5.550828	2.781395	-0.697828
102	1	0	4.774328	5.900797	-3.231682
103	1	0	6.235956	5.247180	-3.195731
104	1	0	5.712740	6.107542	-1.951460
105	1	0	4.065597	2.457703	-2.673984
106	1	0	5.289466	2.945303	-3.580688
107	1	0	3.858529	3.649817	-3.722188
108	1	0	4.524655	0.510124	-1.263129
109	1	0	6.656830	-0.292551	-1.872994
110	1	0	7.468282	-2.232178	-0.920837
111	1	0	7.996503	-4.888843	1.391765
112	1	0	8.311876	-3.345106	1.114964
113	1	0	7.891190	-4.305177	-0.094459
114	1	0	5.293594	-3.148106	2.913943
115	1	0	6.823824	-2.676275	2.923870
116	1	0	6.454450	-4.201129	3.241491
117	1	0	5.389805	-5.097269	-0.090434
118	1	0	4.500246	-4.576949	1.133192
119	1	0	5.665425	-5.644497	1.386660
120	1	0	-2.019216	5.828239	3.297636
121	1	0	-0.819309	4.920351	2.752395
122	1	0	-1.872763	4.312807	3.791851
123	1	0	-2.476208	6.336541	0.505335
124	1	0	-3.183351	5.231795	-0.410770
125	1	0	-1.606402	5.157253	-0.139637
126	1	0	-4.651222	4.007345	2.975989
127	1	0	-5.062560	4.207662	1.441888
128	1	0	-4.699114	5.477958	2.346840

The total electronic energy was calculated to be –4409.880142 Hartree..

Supplementary Table 55. Optimized structure of **2B** (B3LYP/6-31G(d))

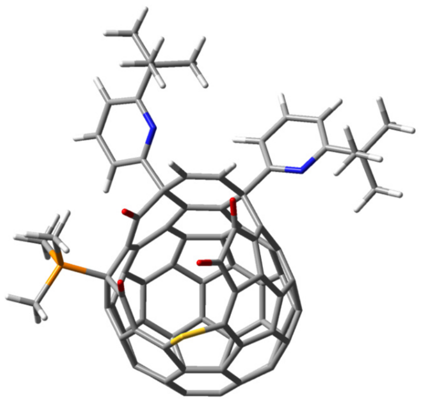

Standard orientation:

Center Number	Atomic Number	Atomic Type	Coordinates (Angstroms) X	Y	Z
1	6	0	2.772990	1.433356	1.249750
2	6	0	3.181981	0.166597	1.300601
3	6	0	2.692451	-0.939157	0.397178
4	6	0	1.747786	-1.922975	1.131789
5	6	0	1.317890	-1.747698	2.447785
6	6	0	1.592896	-0.802942	3.593290
7	6	0	0.199199	-0.576817	4.311117
8	6	0	-0.734412	-1.538764	3.678880
9	6	0	-2.110625	-1.516626	3.465234
10	6	0	-2.738869	-2.445002	2.542489
11	6	0	-3.948878	-2.110425	1.766978
12	6	0	-4.403111	-0.709354	1.704475
13	6	0	-3.738411	0.322849	2.483648
14	6	0	-3.508401	1.580283	1.924179
15	6	0	-2.412056	2.565847	2.190327
16	6	0	-1.964681	3.162843	0.745771
17	6	0	-0.649410	2.682454	0.084863
18	6	0	0.540595	2.586173	0.882809
19	6	0	1.837607	1.991640	0.204432
20	6	0	1.436300	0.945047	-0.802088
21	6	0	1.845507	-0.373272	-0.747440
22	6	0	1.279576	-1.338662	-1.661861
23	6	0	0.944348	-2.673114	-1.175043
24	6	0	1.046350	-2.887409	0.275823
25	6	0	0.076646	-3.733682	0.853674
26	6	0	-0.489123	-3.370486	2.117560
27	6	0	0.077140	-2.365252	2.844691
28	6	0	-1.916319	-3.487037	2.047040
29	6	0	-2.207213	-4.218903	0.851871
30	6	0	-3.397182	-3.992803	0.194166
31	6	0	-4.290497	-2.953518	0.675136
32	6	0	-4.993073	-2.465296	-0.495597
33	6	0	-5.406940	-1.149723	-0.552787
34	6	0	-5.107953	-0.279853	0.556249
35	6	0	-4.939933	1.043511	0.019535
36	6	0	-4.036182	1.898786	0.640198
37	6	0	-3.154399	2.742182	-0.102478
38	6	0	-3.069600	2.531523	-1.466367
39	6	0	-1.781671	2.393328	-2.126482
40	6	0	-0.637126	2.273208	-1.290737
41	6	0	0.419454	1.352514	-1.738546
42	6	0	0.193673	0.532675	-2.839334
43	6	0	0.631543	-0.843237	-2.799919
44	6	0	-0.323472	-1.618112	-3.546464
45	6	0	-0.640240	-2.893300	-3.111025
46	6	0	0.001756	-3.424284	-1.924136
47	6	0	-0.983978	-4.265658	-1.274123
48	6	0	-0.970349	-4.364708	0.102061
49	6	0	-2.246490	-4.154287	-1.979436
50	6	0	-3.441516	-4.034806	-1.253481
51	6	0	-4.454064	-3.103070	-1.686392
52	6	0	-4.272865	-2.355017	-2.854216
53	6	0	-4.702389	-0.968474	-2.905239
54	6	0	-5.300328	-0.387710	-1.786039
55	6	0	-4.986030	0.974099	-1.416167
56	6	0	-4.041473	1.698062	-2.151946
57	6	0	-3.385547	1.085401	-3.280282
58	6	0	-1.993717	1.505180	-3.241107
59	6	0	-1.009423	0.604424	-3.634554
60	6	0	-1.352967	-0.726780	-4.054901
61	6	0	-2.688367	-1.143818	-4.093670

62	6	0	-3.027988	-2.465777	-3.597324
63	6	0	-3.721035	-0.218794	-3.673814
64	6	0	2.584588	3.173026	-0.473597
65	6	0	-2.028013	-3.335128	-3.148390
66	6	0	1.857748	4.271450	-0.961914
67	6	0	2.564762	5.330007	-1.517605
68	6	0	3.958600	5.272031	-1.575637
69	6	0	4.610616	4.145864	-1.062736
70	6	0	6.136593	3.967578	-1.079080
71	6	0	6.624834	3.708185	0.363963
72	6	0	6.864291	5.202837	-1.638217
73	6	0	6.475628	2.740612	-1.956840
74	6	0	3.937277	-1.674121	-0.149286
75	6	0	4.762087	-1.043999	-1.090617
76	6	0	5.900869	-1.718236	-1.510477
77	6	0	6.185051	-2.981179	-0.985631
78	6	0	5.315035	-3.537501	-0.042739
79	6	0	5.521331	-4.915147	0.603623
80	6	0	6.811683	-5.601061	0.122325
81	6	0	5.585459	-4.734481	2.137481
82	6	0	4.311396	-5.808788	0.245934
83	6	0	-0.483491	5.698960	1.813361
84	6	0	-2.022260	5.792513	-0.695763
85	6	0	-3.423031	5.571642	1.891000
86	16	0	-3.002642	-0.085408	4.050976
87	15	0	-1.945791	5.031829	0.958492
88	8	0	-2.021295	2.968905	3.261419
89	8	0	0.630266	3.003945	2.046654
90	8	0	0.018120	0.219951	5.202724
91	8	0	2.625639	-0.359893	4.037270
92	7	0	3.916621	3.125763	-0.519958
93	7	0	4.211421	-2.875064	0.358463
94	1	0	3.142152	2.137813	1.987072
95	1	0	3.873525	-0.137850	2.077445
96	1	0	0.775628	4.284023	-0.887888
97	1	0	2.038183	6.201301	-1.899910
98	1	0	4.517957	6.094344	-2.005473
99	1	0	6.407338	4.564010	1.014569
100	1	0	7.708771	3.542351	0.372725
101	1	0	6.134693	2.824990	0.781630
102	1	0	6.584233	5.408411	-2.678157
103	1	0	7.946460	5.032710	-1.619113
104	1	0	6.660861	6.101525	-1.043841
105	1	0	5.963959	1.847047	-1.588989
106	1	0	7.556191	2.553308	-1.946531
107	1	0	6.171860	2.904540	-2.998051
108	1	0	4.512777	-0.057412	-1.468011
109	1	0	6.567490	-1.267679	-2.241492
110	1	0	7.071271	-3.513937	-1.309024
111	1	0	6.916095	-6.575744	0.612068
112	1	0	7.703911	-5.012639	0.367228
113	1	0	6.802521	-5.777154	-0.959905
114	1	0	4.684952	-4.235183	2.504548
115	1	0	6.452786	-4.129222	2.427491
116	1	0	5.672273	-5.709621	2.631788
117	1	0	4.252146	-5.978528	-0.836002
118	1	0	3.377327	-5.340967	0.568411
119	1	0	4.402117	-6.785119	0.737264
120	1	0	-0.631749	6.770317	1.988755
121	1	0	0.403466	5.532042	1.201800
122	1	0	-0.352708	5.158293	2.751837
123	1	0	-2.016622	6.883061	-0.606162
124	1	0	-2.926945	5.468970	-1.216086
125	1	0	-1.150834	5.468632	-1.271140
126	1	0	-3.348689	5.215055	2.922159
127	1	0	-4.321947	5.152902	1.430475
128	1	0	-3.491054	6.663685	1.892877

The total electronic energy was calculated to be –4409.8733493 Hartree..

Supplementary Table 56. Optimized structure of **2** (M06-2X/6-31G(d,p))

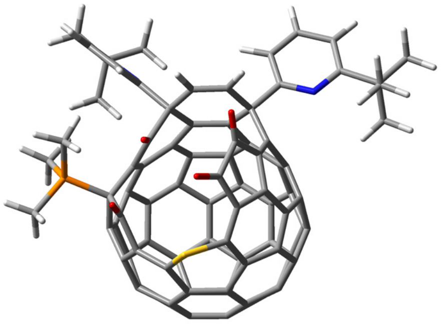

Standard orientation:

Center Number	Atomic Number	Atomic Type	Coordinates (Angstroms) X	Y	Z
1	6	0	2.226549	1.922894	1.958449
2	6	0	2.985886	0.832079	1.868627
3	6	0	2.911454	-0.186669	0.757269
4	6	0	2.247673	-1.509081	1.216142
5	6	0	1.690195	-1.697954	2.481418
6	6	0	1.589988	-0.934023	3.780165
7	6	0	0.142976	-1.236460	4.348924
8	6	0	-0.416779	-2.291424	3.471188
9	6	0	-1.718901	-2.615423	3.097556
10	6	0	-1.976365	-3.501919	1.976901
11	6	0	-3.171143	-3.384596	1.118866
12	6	0	-4.012322	-2.177530	1.211840
13	6	0	-3.740229	-1.157295	2.211573
14	6	0	-3.850806	0.193242	1.878598
15	6	0	-3.116196	1.381062	2.420423
16	6	0	-2.760293	2.326594	1.146612
17	6	0	-1.317220	2.357082	0.586147
18	6	0	-0.213381	2.455095	1.499401
19	6	0	1.247217	2.375888	0.902505
20	6	0	1.246942	1.455912	-0.290446
21	6	0	2.021405	0.315818	-0.384225
22	6	0	1.833612	-0.589755	-1.494672
23	6	0	1.871954	-2.029309	-1.257295
24	6	0	1.926219	-2.461886	0.146725
25	6	0	1.209502	-3.633498	0.469302

26	6	0	0.470988	-3.673533	1.695030	79	6	0	6.763569	-3.188764	0.709148
27	6	0	0.661047	-2.696866	2.627697	80	6	0	8.230347	-3.391212	0.291161
28	6	0	-0.849295	-4.168136	1.434903	81	6	0	6.659266	-3.273916	2.249065
29	6	0	-0.823344	-4.726707	0.117329	82	6	0	5.900332	-4.305116	0.077437
30	6	0	-1.975668	-4.727194	-0.638895	83	6	0	-2.400440	4.790960	3.034195
31	6	0	-3.168292	-4.080684	-0.120118	84	6	0	-3.094649	5.151366	0.195102
32	6	0	-3.895991	-3.606662	-1.281301	85	6	0	-5.083688	4.052414	2.076143
33	6	0	-4.673898	-2.470003	-1.190993	86	16	0	-3.033473	-1.617328	3.777910
34	6	0	-4.726517	-1.763573	0.063684	87	15	0	-3.308736	4.057867	1.637141
35	6	0	-4.917253	-0.372120	-0.244639	88	8	0	-2.941007	1.679222	3.579214
36	6	0	-4.353659	0.575921	0.602158	89	8	0	-0.336181	2.666812	2.714804
37	6	0	-3.707811	1.749741	0.106727	90	8	0	-0.329592	-0.693851	5.320956
38	6	0	-3.465201	1.817723	-1.252788	91	8	0	2.410905	-0.307638	4.407625
39	6	0	-2.149099	2.163423	-1.764625	92	7	0	2.188483	4.007347	-0.705487
40	6	0	-1.084310	2.219545	-0.823372	93	7	0	4.932130	-1.584157	0.614429
41	6	0	0.226489	1.724484	-1.272027	94	1	0	2.316725	2.559019	2.832174
42	6	0	0.333507	1.084175	-2.502230	95	1	0	3.677706	0.598670	2.669448
43	6	0	1.153368	-0.099583	-2.616086	96	1	0	1.109759	4.671330	2.415131
44	6	0	0.526438	-0.963562	-3.580613	97	1	0	1.868177	6.968054	1.748764
45	6	0	0.568656	-2.332883	-3.382545	98	1	0	2.827662	7.314394	-0.516474
46	6	0	1.250059	-2.867003	-2.219267	99	1	0	5.148323	4.561291	-1.954284
47	6	0	0.511348	-4.051321	-1.827097	100	1	0	4.732038	4.347455	-3.666143
48	6	0	0.452906	-4.386406	-0.489868	101	1	0	3.985847	3.307349	-2.432891
49	6	0	-0.672795	-4.171788	-2.656012	102	1	0	2.892772	7.493749	-2.787178
50	6	0	-1.899679	-4.521037	-2.071073	103	1	0	4.094973	6.756881	-3.850252
51	6	0	-3.107385	-3.846254	-2.479623	104	1	0	4.492112	7.050339	-2.152538
52	6	0	-3.069846	-2.881575	-3.491658	105	1	0	1.653436	3.963134	-3.290309
53	6	0	-3.884040	-1.683391	-3.386589	106	1	0	2.440398	4.965992	-4.529990
54	6	0	-4.706754	-1.501239	-2.274209	107	1	0	1.221858	5.675947	-3.454559
55	6	0	-4.835739	-0.194452	-1.669410	108	1	0	4.522292	1.484695	-0.719801
56	6	0	-4.095383	0.882887	-2.168303	109	1	0	6.893249	1.052179	-1.409987
57	6	0	-3.207351	0.688397	-3.287368	110	1	0	7.967147	-1.093913	-0.769676
58	6	0	-2.007849	1.464934	-3.016951	111	1	0	8.581309	-4.369389	0.638656
59	6	0	-0.775773	0.958879	-3.418069	112	1	0	8.889092	-2.631230	0.727935
60	6	0	-0.679983	-0.318161	-4.071375	113	1	0	8.352651	-3.366980	-0.798228
61	6	0	-1.826558	-1.076480	-4.334124	114	1	0	5.627279	-3.118736	2.574447
62	6	0	-1.796781	-2.507164	-4.086063	115	1	0	7.286025	-2.513015	2.729590
63	6	0	-3.113807	-0.565692	-3.909344	116	1	0	6.993262	-4.258235	2.598831
64	6	0	1.660280	3.819662	0.504666	117	1	0	5.973020	-4.288879	-1.016803
65	6	0	-0.620614	-3.129423	-3.653990	118	1	0	4.848908	-4.181033	0.350277
66	6	0	1.524629	4.866069	1.431983	119	1	0	6.238620	-5.288941	0.424578
67	6	0	1.950022	6.133725	1.056093	120	1	0	-2.876449	5.738470	3.310219
68	6	0	2.491375	6.329273	-0.215996	121	1	0	-1.361760	4.955745	2.746879
69	6	0	2.598570	5.235246	-1.080900	122	1	0	-2.412414	4.084674	3.865566
70	6	0	3.169103	5.331105	-2.504263	123	1	0	-3.409398	6.168512	0.447492
71	6	0	4.331154	4.322318	-2.645772	124	1	0	-3.683117	4.780629	-0.647591
72	6	0	3.689295	6.741648	-2.832612	125	1	0	-2.038664	5.155304	-0.088131
73	6	0	2.050569	4.959030	-3.504910	126	1	0	-5.224320	3.488026	3.002242
74	6	0	4.354078	-0.436460	0.262468	127	1	0	-5.658924	3.578393	1.276215
75	6	0	5.023032	0.555641	-0.466714	128	1	0	-5.440437	5.076423	2.222173
76	6	0	6.337464	0.311077	-0.841094						
77	6	0	6.942104	-0.895390	-0.480409						
78	6	0	6.208212	-1.830697	0.255846						

The total electronic energy was calculated to be –4408.5679684 Hartree..

Supplementary Table 57. Optimized structure of **3d** (M06-2X/6-31G(d,p))

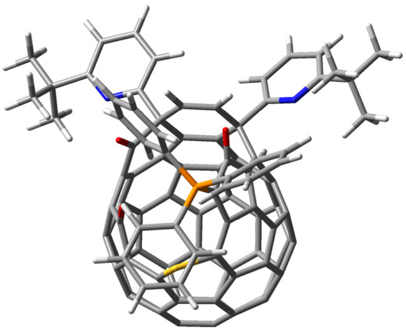

Standard orientation:

Center Number	Atomic Number	Atomic Type	Coordinates (Angstroms) X	Y	Z
1	6	0	0.709409	2.844048	1.130363
2	6	0	0.996711	2.947729	-0.161340
3	6	0	0.075950	2.594122	-1.295482
4	6	0	0.595600	1.336844	-2.033681
5	6	0	1.723085	0.624155	-1.640996
6	6	0	2.858988	0.733971	-0.605261
7	6	0	3.134521	-0.624013	-0.149166
8	6	0	2.368418	-1.557119	-0.971241
9	6	0	1.795398	-2.819009	-0.816212
10	6	0	0.809439	-3.347918	-1.755185
11	6	0	-0.291435	-4.246369	-1.350195
12	6	0	-0.598508	-4.420287	0.080351
13	6	0	0.251249	-3.798607	1.084943
14	6	0	-0.312777	-3.207267	2.201755
15	6	0	0.123453	-1.889844	2.838719
16	6	0	-1.212769	-1.127491	3.011680
17	6	0	-1.501451	0.187614	2.674007
18	6	0	-0.443171	1.237108	2.633968
19	6	0	-0.632322	2.428654	1.658376
20	6	0	-1.603665	2.077705	0.554208
21	6	0	-1.314325	2.199348	-0.786238
22	6	0	-2.257891	1.712281	-1.769035
23	6	0	-1.764274	1.021959	-2.957046
24	6	0	-0.329001	0.721819	-2.989822
25	6	0	0.031123	-0.483267	-3.604281
26	6	0	1.055284	-1.277795	-3.011309
27	6	0	1.814016	-0.747586	-2.026550
28	6	0	0.634372	-2.646080	-2.958685
29	6	0	-0.537457	-2.738644	-3.771324
30	6	0	-0.913152	-1.398268	-4.183974
31	6	0	-2.235104	-1.047993	-4.265482
32	6	0	-2.661624	0.198965	-3.658306
33	6	0	-4.018408	-0.018643	-3.201290
34	6	0	-4.467113	0.619220	-2.066318
35	6	0	-3.562607	1.478367	-1.345224
36	6	0	-3.854017	1.337716	0.060314
37	6	0	-2.832779	1.455647	0.990939
38	6	0	-2.799561	0.517695	2.116369
39	6	0	-3.835927	-0.418837	2.219774
40	6	0	-3.554484	-1.762139	2.643096
41	6	0	-2.247066	-2.101182	2.929386
42	6	0	-1.707317	-3.346214	2.435010
43	6	0	-2.483986	-4.184637	1.672723
44	6	0	-1.912275	-4.728161	0.465561
45	6	0	-2.987070	-4.870655	-0.477841
46	6	0	-2.713672	-4.724981	-1.816354
47	6	0	-1.361732	-4.441023	-2.256334
48	6	0	-1.500934	-3.668361	-3.473787
49	6	0	-2.896921	-3.339376	-3.678762
50	6	0	-3.261463	-2.050983	-4.065986
51	6	0	-4.387399	-1.405630	-3.435557
52	6	0	-5.162772	-2.090620	-2.506646
53	6	0	-5.648749	-1.406272	-1.323021
54	6	0	-5.309019	-0.075555	-1.117505
55	6	0	-4.925375	0.378542	0.197062
56	6	0	-4.918895	-0.492901	1.265774
57	6	0	-5.272442	-1.885027	1.063149
58	6	0	-4.408364	-2.675993	1.908086
59	6	0	-3.876994	-3.868331	1.428378
60	6	0	-4.200712	-4.318168	0.097047
61	6	0	-5.071761	-3.586383	-0.700124
62	6	0	-4.792136	-3.437462	-2.116916
63	6	0	-3.660411	-4.027273	-2.668605
64	6	0	-5.613661	-2.334873	-0.209901
65	6	0	-1.264648	3.567673	2.479557
66	6	0	-1.272006	4.875518	1.993336
67	6	0	-1.901488	5.841921	2.762293
68	6	0	-2.494933	5.481051	3.970422
69	6	0	-2.449289	4.146752	4.373971
70	6	0	-3.064257	3.619321	5.666803
71	6	0	-3.741557	4.726272	6.476517
72	6	0	-1.944470	2.980953	6.504142
73	6	0	-4.100797	2.544110	5.303160
74	6	0	-0.043468	3.812012	-2.246319
75	6	0	-0.988668	4.816364	-2.017583
76	6	0	-1.058382	5.868291	-2.921817
77	6	0	-0.206641	5.891362	-4.019419
78	6	0	0.700422	4.841893	-4.183238
79	6	0	1.653155	4.720858	-5.370514
80	6	0	3.076683	4.480090	-4.846099
81	6	0	1.209475	3.508865	-6.206920
82	6	0	1.640168	5.973683	-6.249007
83	6	0	4.668801	0.417153	2.125299
84	6	0	3.482617	0.789586	2.760676
85	6	0	3.526666	1.680111	3.829828
86	6	0	4.746589	2.198309	4.257085
87	6	0	5.927390	1.829608	3.617454
88	6	0	5.892899	0.939612	2.548934
89	6	0	4.783471	-2.441914	1.510536
90	6	0	4.452698	-2.656813	2.849168
91	6	0	4.533843	-3.941846	3.376558
92	6	0	4.945732	-5.003883	2.575035
93	6	0	5.280616	-4.787472	1.240311
94	6	0	5.201115	-3.506955	0.704458
95	6	0	6.112322	-0.582567	-0.218673
96	6	0	6.059189	0.133022	-1.416858
97	6	0	7.215375	0.308795	-2.171964
98	6	0	8.424584	-0.226398	-1.738365
99	6	0	8.482240	-0.941885	-0.544374
100	6	0	7.330163	-1.122846	0.213630
101	16	0	1.974262	-3.619561	0.762047
102	15	0	4.593113	-0.795091	0.779825

103	7	0	-1.840465	3.217724	3.622631
104	7	0	0.764641	3.839532	-3.300124
105	8	0	1.243877	-1.501118	3.029012
106	8	0	3.511906	1.735566	-0.325244
107	8	0	0.519291	1.189103	3.363796
108	1	0	1.467475	3.065369	1.876986
109	1	0	1.996979	3.245754	-0.453377
110	1	0	-0.807594	5.107613	1.040754
111	1	0	-1.934972	6.873845	2.426243
112	1	0	-2.986024	6.228725	4.580831
113	1	0	-4.559448	5.192656	5.917320
114	1	0	-3.030152	5.506336	6.766983
115	1	0	-4.163819	4.302929	7.392515
116	1	0	-2.362670	2.542244	7.415905
117	1	0	-1.199690	3.729533	6.793275
118	1	0	-1.441036	2.198010	5.931410
119	1	0	-4.912285	2.970092	4.704110
120	1	0	-4.533190	2.117778	6.214146
121	1	0	-3.632658	1.742115	4.726690
122	1	0	-1.640331	4.784059	-1.152816
123	1	0	-1.774461	6.670047	-2.768559
124	1	0	-0.249581	6.707861	-4.729444
125	1	0	3.418406	5.326682	-4.241770
126	1	0	3.766692	4.357598	-5.687109
127	1	0	3.111491	3.580120	-4.227022
128	1	0	1.887276	3.370911	-7.055831
129	1	0	0.195845	3.651257	-6.595386
130	1	0	1.222253	2.600494	-5.598634
131	1	0	2.358120	5.854283	-7.065646
132	1	0	1.922701	6.866601	-5.681495
133	1	0	0.656103	6.143960	-6.697959
134	1	0	2.529829	0.386044	2.432989
135	1	0	2.595279	1.959480	4.309934
136	1	0	4.778685	2.895935	5.088365
137	1	0	6.877616	2.240393	3.943063
138	1	0	6.813532	0.672718	2.040347
139	1	0	4.106302	-1.832922	3.464832
140	1	0	4.269654	-4.113119	4.414728
141	1	0	5.005216	-6.004557	2.991131
142	1	0	5.596917	-5.615476	0.614963
143	1	0	5.460374	-3.335485	-0.337050
144	1	0	5.121573	0.563014	-1.749632
145	1	0	7.167678	0.865416	-3.101933
146	1	0	9.323014	-0.089509	-2.331711
147	1	0	9.422025	-1.365557	-0.205676
148	1	0	7.376085	-1.694335	1.136889

The total electronic energy was calculated to be –4908.3708859 Hartree..

Supplementary Note 4

13. TD-DFT Calculations

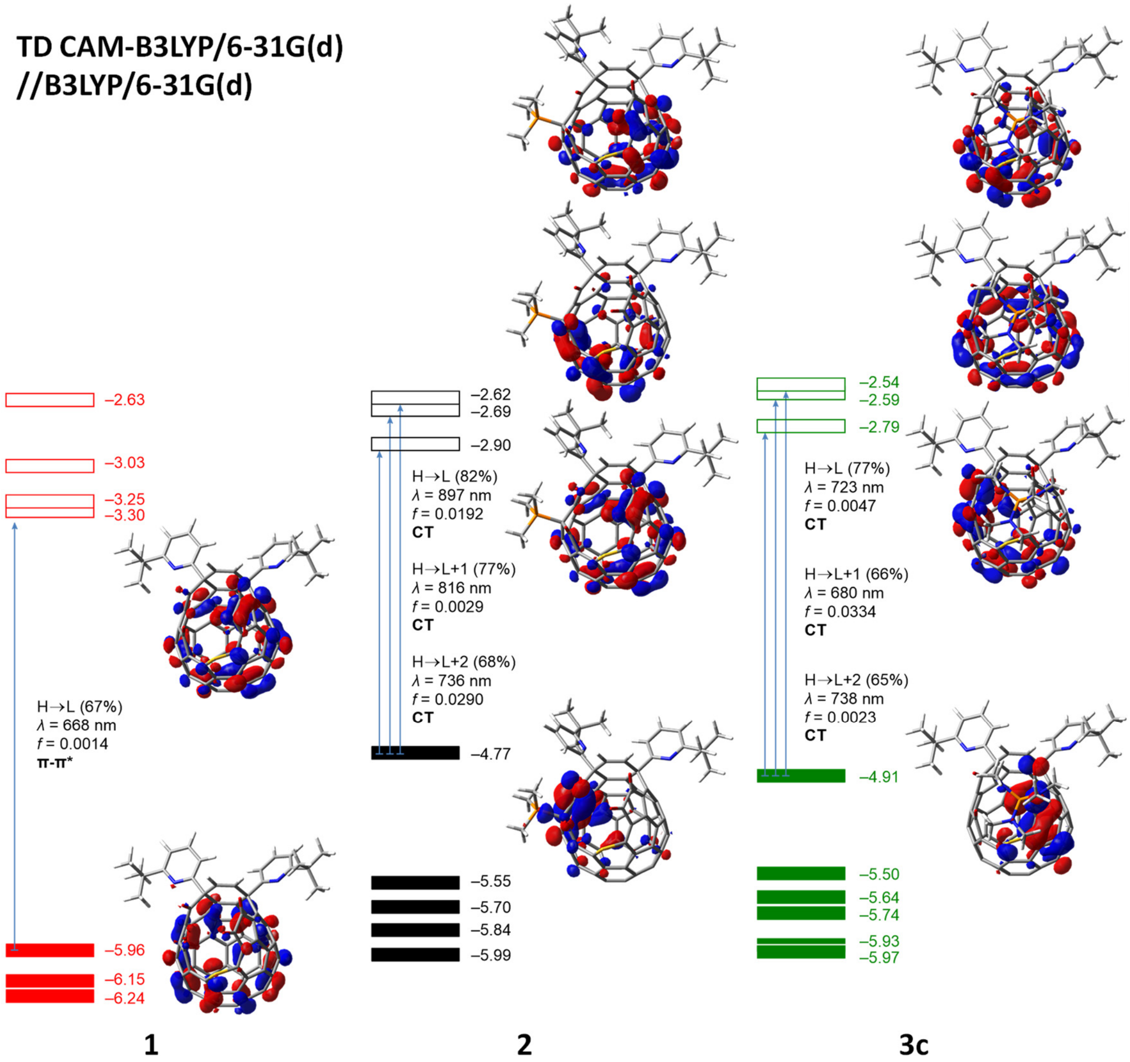

Supplementary Fig. 53. Pictorial representation of the Kohn-Sham frontier orbitals with their energy levels (B3LYP/6-31G(d)) and optical transitions with oscillator strengths (TD CAM-B3LYP/6-31G(d)) of **1**, **2**, and **3c**. The transition energies were calibrated by a factor of 0.72.

Supplementary Table 58. Optimized structure of **1** (B3LYP/6-31G(d))

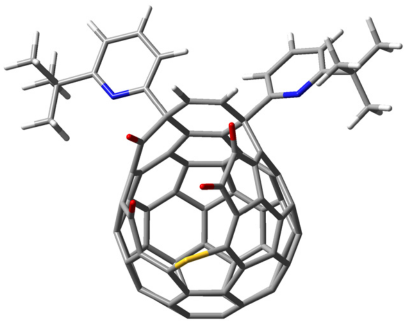

Standard orientation:

Center Number	Atomic Number	Atomic Type	Coordinates (Angstroms) X	Y	Z
1	6	0	2.981706	1.485788	1.414623
2	6	0	3.395449	0.261985	1.087954
3	6	0	2.838839	-0.573684	-0.041731
4	6	0	2.021741	-1.789127	0.471865
5	6	0	1.745531	-2.040875	1.817001
6	6	0	2.109079	-1.454387	3.162086
7	6	0	0.821774	-1.619149	4.072143
8	6	0	-0.130988	-2.426282	3.269586
9	6	0	-1.521132	-2.479728	3.224067
10	6	0	-3.335307	-0.586351	3.033042
11	6	0	-3.184466	0.785378	2.846374
12	6	0	-1.919744	1.618458	3.115680
13	6	0	-1.771789	2.457464	1.820157
14	6	0	-0.626199	2.687170	1.049713
15	6	0	0.757472	2.641483	1.624706
16	6	0	1.939447	2.264667	0.656362
17	16	0	-2.422594	-1.405047	4.324402
18	7	0	1.775158	4.681127	0.222695
19	8	0	3.156528	-1.038911	3.596389
20	8	0	0.980905	2.898938	2.787471
21	7	0	4.627296	-2.187932	-0.391588
22	6	0	1.413247	1.493192	-0.536156
23	6	0	1.211281	-0.477465	-1.977486
24	8	0	0.724343	-1.177779	5.191893
25	6	0	0.273246	2.081982	-1.202052
26	6	0	1.282541	-2.528677	-0.557205
27	8	0	-1.168997	1.528229	4.052557
28	6	0	0.010466	-2.500880	-2.700749
29	6	0	5.739094	-2.669620	-0.976770
30	6	0	-0.843425	-3.576684	-2.233206
31	6	0	4.053056	-1.077705	-0.857623
32	6	0	0.996357	-1.918751	-1.864477
33	6	0	0.407704	0.266893	-2.849942
34	6	0	1.856356	0.232281	-0.895146
35	6	0	-0.654052	-4.069590	-0.959092
36	6	0	-0.598753	-0.340027	-3.682235
37	6	0	0.019482	-3.669795	1.200756
38	6	0	-0.798710	-1.707786	-3.605820
39	6	0	-0.078924	1.566085	-2.448496
40	6	0	-0.755004	2.750765	-0.399787
41	6	0	0.439814	-3.589984	-0.163611
42	6	0	3.847217	3.630757	-0.377820
43	6	0	2.555397	3.596068	0.148931
44	6	0	-3.067269	-3.958264	-0.508345
45	6	0	-2.033738	2.899686	-0.972092
46	6	0	-1.391111	1.745337	-3.024631
47	6	0	4.331338	4.855176	-0.836060
48	6	0	0.611409	-2.870678	2.134290
49	6	0	-2.157113	-2.231293	-3.620190
50	6	0	6.308065	-2.021035	-2.079939
51	6	0	-3.947713	-3.190029	0.356252
52	6	0	-3.247851	-1.359797	-3.680224
53	6	0	-1.395731	-3.897682	1.248201
54	6	0	3.523776	5.983905	-0.749844
55	6	0	6.308587	-3.949078	-0.348109
56	6	0	4.564798	-0.365400	-1.947903
57	6	0	-2.206672	-3.135212	2.121825
58	6	0	2.233542	5.865741	-0.207774
59	6	0	-1.724420	0.567080	-3.791128
60	6	0	-4.806974	-2.431479	-0.529976
61	6	0	-4.899750	-0.680867	1.154143
62	6	0	-1.793933	-4.261745	-0.078281
63	6	0	-3.210169	2.746672	-0.159725
64	6	0	-2.186900	-3.375673	-2.736845
65	6	0	-4.391341	-1.570512	-2.808055
66	6	0	-3.520373	-2.690715	1.616318
67	6	0	5.710893	-0.857432	-2.564439
68	6	0	-3.044925	2.432749	1.180633
69	6	0	-4.049609	-1.380528	2.039463
70	6	0	-4.388239	-2.638939	-1.907790
71	6	0	-3.285630	-3.570395	-1.887264
72	6	0	-3.884958	1.423813	1.787483
73	6	0	-3.029999	0.073378	-3.783577
74	6	0	-4.049267	0.749619	-3.001252
75	6	0	-2.363320	2.408676	-2.292568
76	6	0	-5.347748	-0.092320	-1.084122
77	6	0	-3.726081	1.906073	-2.280216
78	6	0	5.235835	-5.058581	-0.440241
79	6	0	-4.247747	2.104422	-0.946007
80	6	0	-4.829842	0.755711	1.031496
81	6	0	-5.278408	-1.193625	-0.136874
82	6	0	-5.044202	1.117515	-0.356647
83	6	0	-4.883708	-0.268624	-2.390152
84	6	0	6.624171	-3.666065	1.138920
85	6	0	7.589896	-4.427885	-1.052782
86	6	0	1.311843	7.087490	-0.074758
87	6	0	1.095326	7.724682	-1.467553
88	6	0	1.979722	8.120198	0.864532
89	6	0	-0.054805	6.693302	0.510909
90	1	0	3.411007	1.990193	2.274618
91	1	0	4.159541	-0.222030	1.683928
92	1	0	4.453447	2.732253	-0.417339
93	1	0	5.333226	4.927129	-1.251481
94	1	0	7.200753	-2.409171	-2.555634
95	1	0	3.891366	6.944900	-1.094584
96	1	0	4.080082	0.538462	-2.301074
97	1	0	6.138936	-0.338767	-3.418488
98	1	0	5.590745	-5.970753	0.054015
99	1	0	5.011254	-5.305236	-1.485109
100	1	0	4.308166	-4.738857	0.041892
101	1	0	6.984166	-4.578223	1.629483
102	1	0	5.732458	-3.315440	1.665241

103	1	0	7.401981	-2.899449	1.238481
104	1	0	7.958380	-5.338446	-0.567627
105	1	0	8.390295	-3.680490	-0.998655
106	1	0	7.413212	-4.667238	-2.108159
107	1	0	0.625803	7.013983	-2.157339
108	1	0	2.035294	8.061167	-1.918816
109	1	0	0.437565	8.597675	-1.382729
110	1	0	1.336335	9.001406	0.972944
111	1	0	2.947840	8.459945	0.479758
112	1	0	2.142987	7.695550	1.861297
113	1	0	-0.682590	7.587245	0.605133
114	1	0	0.048769	6.235803	1.498600
115	1	0	-0.573640	5.975515	-0.131524

The total electronic energy was calculated to be –3948.7850269 Hartree..

Supplementary Table 59. Optimized structure of **3d** (B3LYP/6-31G(d))

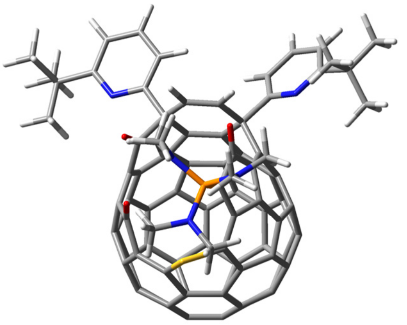

Standard orientation:

Center Number	Atomic Number	Atomic Type	Coordinates (Angstroms) X	Y	Z
1	6	0	-1.343644	2.958645	-0.642516
2	6	0	-2.040593	2.465555	0.380577
3	6	0	-1.449910	1.747137	1.571933
4	6	0	-1.785996	0.231964	1.572150
5	6	0	-2.473327	-0.422541	0.548311
6	6	0	-3.176998	-0.055990	-0.738493
7	6	0	-2.214362	-2.293678	-0.938857
8	6	0	-1.284260	-3.277187	-1.262973
9	6	0	0.967956	-3.171762	-2.615406
10	6	0	1.645193	-2.077094	-3.146708
11	6	0	1.057813	-0.687826	-3.448341
12	6	0	2.092146	0.274787	-2.809796
13	6	0	1.866531	1.388116	-1.992288
14	6	0	0.597274	2.184173	-2.042513
15	6	0	0.159329	2.944958	-0.736316
16	16	0	-0.764521	-3.389749	-2.964594
17	7	0	1.699072	4.599951	-1.708122
18	8	0	-3.915959	0.858856	-1.013052
19	8	0	-0.072860	2.265639	-3.048751
20	7	0	-3.246552	1.933909	3.204117
21	6	0	0.796169	2.305512	0.480112
22	6	0	0.764796	1.111647	2.619126
23	6	0	2.219753	2.072978	0.385094
24	6	0	-1.081769	-0.567177	2.580994
25	8	0	-0.010768	-0.435977	-3.942509
26	6	0	0.976847	-0.960749	3.930417
27	6	0	-3.902363	2.491045	4.238558
28	6	0	0.759669	-2.394529	3.883653
29	6	0	-2.056894	2.409019	2.831913
30	6	0	0.137296	-0.059446	3.228264
31	6	0	2.154493	1.262881	2.701912
32	6	0	0.079303	1.813892	1.556924
33	6	0	-0.318567	-2.880603	3.174204
34	6	0	2.993414	0.329402	3.408201
35	6	0	-1.775144	-2.605920	1.418820
36	6	0	2.412404	-0.772551	4.012117
37	6	0	2.895697	1.741977	1.558695
38	6	0	2.790745	1.656171	-0.898825
39	6	0	-1.258134	-1.967288	2.589031
40	6	0	0.128472	5.427195	-0.093748
41	6	0	0.683768	4.400775	-0.858981
42	6	0	1.101600	-4.560648	2.078873
43	6	0	4.038927	1.003189	-0.875805
44	6	0	4.183324	1.087822	1.559436
45	6	0	0.661247	6.707577	-0.236341
46	6	0	-2.298254	-1.846196	0.414017
47	6	0	3.072849	-2.066691	3.919719
48	6	0	-3.350815	3.570033	4.940665
49	6	0	1.587798	-4.820298	0.733850
50	6	0	4.265243	-2.200048	3.203318
51	6	0	-0.965721	-3.747590	1.105072
52	6	0	1.709405	6.915531	-1.126139
53	6	0	-5.262535	1.860308	4.568167
54	6	0	-1.442868	3.490018	3.474079
55	6	0	-0.533929	-3.965365	-0.224715
56	6	0	2.214088	5.829043	-1.859179
57	6	0	4.253897	0.207233	2.702452
58	6	0	3.029433	-4.694560	0.804649
59	6	0	3.014952	-3.908267	-1.495575
60	6	0	-0.139778	-3.987802	2.249963
61	6	0	4.299554	-0.087268	-1.775982
62	6	0	2.038595	-3.070771	3.806000
63	6	0	4.442140	-3.320737	2.294795
64	6	0	0.845794	-4.451983	-0.420911
65	6	0	-2.109296	4.070356	4.548712
66	6	0	3.288620	-0.480195	-2.639712
67	6	0	1.608885	-3.999266	-1.599245
68	6	0	3.413779	-4.253203	2.136653
69	6	0	2.205942	-4.144713	2.919685
70	6	0	3.017523	-1.888142	-2.829621
71	6	0	4.881422	-1.034638	2.591534
72	6	0	5.461162	-1.435797	1.322197
73	6	0	4.753170	0.720927	0.350391
74	6	0	4.850003	-3.325905	-0.135291
75	6	0	5.411950	-0.567481	0.224322
76	6	0	-5.037978	0.373373	4.927147
77	6	0	5.116341	-1.076559	-1.096676
78	6	0	3.742645	-2.838120	-2.135055
79	6	0	3.734966	-4.245721	-0.295271

80	6	0	4.836617	-2.435490	-1.271972
81	6	0	5.179371	-2.847265	1.135358
82	6	0	-6.162433	1.952634	3.314132
83	6	0	-5.963982	2.561798	5.744369
84	6	0	3.358521	6.012742	-2.867337
85	6	0	4.591567	6.606013	-2.145916
86	6	0	2.896689	6.984909	-3.979302
87	6	0	3.754429	4.672896	-3.510910
88	1	0	-1.861812	3.418092	-1.478415
89	1	0	-3.121983	2.527197	0.371300
90	1	0	-0.697554	5.230180	0.580976
91	1	0	0.256574	7.536338	0.338922
92	1	0	-3.874815	4.017694	5.776642
93	1	0	2.127579	7.908708	-1.253446
94	1	0	-0.477424	3.859332	3.145183
95	1	0	-1.665829	4.908589	5.080070
96	1	0	-5.999119	-0.121398	5.110783
97	1	0	-4.427342	0.273413	5.832769
98	1	0	-4.527673	-0.147107	4.112328
99	1	0	-7.124892	1.462154	3.502237
100	1	0	-5.685696	1.468772	2.457567
101	1	0	-6.362118	2.997455	3.047533
102	1	0	-6.928330	2.079439	5.938363
103	1	0	-6.162960	3.618634	5.530660
104	1	0	-5.375901	2.502143	6.667911
105	1	0	4.940208	5.939457	-1.348735
106	1	0	4.378306	7.583215	-1.698710
107	1	0	5.414413	6.740158	-2.857881
108	1	0	3.700802	7.129293	-4.710593
109	1	0	2.629364	7.969495	-3.579480
110	1	0	2.024028	6.587842	-4.509700
111	1	0	4.562151	4.840016	-4.233252
112	1	0	2.910665	4.213772	-4.033252
113	1	0	4.106409	3.957449	-2.761658
114	6	0	-3.350424	-1.488702	-1.542775
115	15	0	-4.336497	-1.294574	-2.654573
116	7	0	-3.826918	0.120987	-3.591440
117	6	0	-4.748578	0.564549	-4.622766
118	6	0	-2.487071	-0.030568	-4.142040
119	7	0	-5.908673	-1.202960	-1.830472
120	6	0	-7.075556	-0.995834	-2.672706
121	6	0	-5.911834	-0.230712	-0.746046
122	7	0	-4.341367	-2.896016	-3.426153
123	6	0	-4.704625	-2.928916	-4.832331
124	6	0	-5.088701	-3.905331	-2.689122
125	1	0	-4.355875	1.466537	-5.145842
126	1	0	-4.941784	-0.211537	-5.394446
127	1	0	-5.718442	0.885764	-4.187555
128	1	0	-2.449780	-0.775532	-4.967773
129	1	0	-2.115030	0.939701	-4.544761
130	1	0	-1.749316	-0.335015	-3.364981
131	1	0	-7.992324	-1.344248	-2.142591
132	1	0	-7.031640	-1.574722	-3.619047
133	1	0	-7.237828	0.074882	-2.924688
134	1	0	-6.880606	-0.252866	-0.195500
135	1	0	-5.749871	0.807003	-1.119600
136	1	0	-5.127990	-0.455780	0.012813
137	1	0	-4.591626	-3.957702	-5.245354
138	1	0	-5.754661	-2.611915	-5.013074
139	1	0	-4.026588	-2.296274	-5.445074
140	1	0	-4.832772	-4.926354	-3.055429
141	1	0	-6.189722	-3.778108	-2.796220
142	1	0	-4.834721	-3.900760	-1.604267

The total electronic energy was calculated to be –4618.6439763 Hartree..

14. Supplementary References

(1) Futagoishi, T., Murata, M., Wakamiya, A., Sasamori, T. & Murata, Y. *Org. Lett.* **15**, 2750–2753 (2013).

(2) Sheldrick, G. M. *Acta Crystallogr. A* **71**, 3–8 (2015).

(3) Liu, Y., Sun, F. & He, Z. *Tetrahedron Lett.* **59**, 4136–4148 (2018).

(4) Ramirez, F. & Desai, N. B. *J. Am. Chem. Soc.* **85**, 3252–3258 (1963).

(5) Ferao, A. E. *Inorg. Chem.* **57**, 8058−8064 (2018).

(6) Ramirez, F., Madan, O. P. & Smith, C. P. *J. Am. Chem. Soc.* **87**, 670–671 (1965).

(7) Zhang, W. & Shi, M. *Chem. Commun.* 1218–1220 (2006).
