# Supplementary material for: Organophosphorus zwitterions engaged in a conjugated macrocycle on fullerene
Source: Commun Chem. 2020 Jul 21;3:90. doi: 10.1038/s42004-020-00340-x (PMC9814461; doi:10.1038/s42004-020-00340-x)
Supplement: Supplementary file 3 — Description of Additional Supplementary Files [file 42004_2020_340_MOESM3_ESM.pdf]

Description of Additional Supplementary Files

File Name: Supplementary Data 1
Description: crystallographic information file for compound 2

File Name: Supplementary Data 2
Description: crystallographic information file for compound 3d
