# Supplementary material for: Organophosphorus zwitterions engaged in a conjugated macrocycle on fullerene
Source: Commun Chem. 2020 Jul 21;3:90. doi: 10.1038/s42004-020-00340-x (PMC9814461; doi:10.1038/s42004-020-00340-x)
Supplement: Supplementary file 1 — Peer Review File [file 42004_2020_340_MOESM1_ESM.pdf]

Reviewers' comments:

Reviewer #1 (Remarks to the Author):

The manuscript reports the reaction between an open-cage fullerene and phosphines. One of the products is an unstable phospha-Michael adduct and the other one is a phosphorus ylide. The results are important for the modification of open-cage fullerenes. But the reaction pattern and structure of the new compounds are the same as those in well established classical organic chemistry. The work is too specific and unlikely to attract the attention of general readers of nature communication chemistry.

Reviewer #2 (Remarks to the Author):

This is an interesting manuscript worthy of publication. However, there are significant problems that require the manuscript to be revised before being accepted for publication

The title: "Organophosphorus zwitterions engaged in a conjugated macrocycle on fullerene" doe not seem to be very accurate, as there does not seem to be a macrocycle present in the molecules. Maybe "The synthesis and characterization of organophosphorus zwitterions attached to the rim of open-cage substituted fullerenes."

Page 4, line 14: "Since 1 spontaneously encapsulates a water molecule inside its cage14, the 31P signal of H2O@2 (δ +37.3 ppm) was also observed." This repeats the previous sentence, the included water molecule would have little impact of the P signal. So, delete or rewrite.

Page 7: From the C-O bond lengths, it looks like the negative charges are partly localized on O3 (1pph_c) and O1 & O3 (2Pint_b). The authors should comment on this

Page 7: "The driving force for this self-assembly should be attributed to the intermolecular electrostatic attraction between +PMe3 and C–O– moieties and thus the reactive sites (phosphonium betaine) were fully covered with the four fullerene cages in total." Specify the P…O distances. Are the atoms involved in these interactions P…O1 & P… O3 (as suggested above based on C-O bond lengths)? The authors should comment on this.

Page 7: "The X-ray diffraction analysis revealed that an asymmetric unit accommodates a racemate including two enantiomeric isomers fC-2 and fA-2 arranged as two crystallographically independent molecules (nomenclature based on criteria stated by Diederich20,21). Interestingly, a unit of the racemate is assembled with another one as a tetrahedral configuration via heterochiral recognition" Are there any significant differences between the two crystallographically unique enantiomers? How distorted is the tetrahedron? What are the intermolecular interactions? Is there a refrerence?

Page 12: "Note that all the compounds shown herein encapsulate a water molecule inside their cages with an occupation level of 10–70% at room temperature." How was this determined for each compound? From the two crystal structures it looks like the cavity is fully occupied (or one fully occupied with the other maybe). There is no information on the water content for the four other structures. There are no chemical analyses for any of the compounds. What about the possibility of nitrogen?

Page 12: "conjugated macrocycle on 1" replace by "open-cage fullerene"

Synthesis:

There is no chemical analysis.

General:

The terminology “H2O@2” replace by something like “2.H2O”

The English language expression needs some minor improvement.

Crystallography:

1pph_c:

The structure seems to have been carried out reasonably well. The dichloromethane could have been dealt with using the OLEX2 solvent mask routine. There are a number of issues in the checkCIF report that should have been addressed in the refinement.

PLAT213_ALERT_2_B Atom C85 has ADP max/min Ratio ..... 4.2 prolat

DIFMX02_ALERT_1_C The maximum difference density is > 0.1*ZMAX*0.75
The relevant atom site should be identified.

PLAT077_ALERT_4_C Unitcell Contains Non-integer Number of Atoms .. Please Check
PLAT097_ALERT_2_C Large Reported Max. (Positive) Residual Density 1.68 eA-3
PLAT214_ALERT_2_C Atom C107 (Anion/Solvent) ADP max/min Ratio 4.1 prolat

PLAT601_ALERT_2_C Structure Contains Solvent Accessible VOIDS of . 49 Ang**3

PLAT899_ALERT_4_G SHELXL97 is Deprecated and Succeeded by SHELXL 2018 Note

The refinement should be carried with the latest version of SHELXL (SHELXL_2018). Atoms with unusual ADP max/min ratios should have been restrained to near isotropic values with the ISOR instruction. The large electron density peak is presumably associated with the solvent. As the disorder of the solvent is extensive, the refinement should have used OLEX2 to enable the use of the solvent mask routine to account for the disordered solvent, or the data processed with the SQUEEZE routine of Platon. With no embedded HKL data and RES file in the CIF file it is not possible to be certain of how much this would improve the refinement.

2Pint_b:

PLAT029_ALERT_3_B _diffrn_measured_fraction_theta_full value Low . 0.940 Why?
PLAT213_ALERT_2_B Atom C13 has ADP max/min Ratio ..... 5.0 oblate
PLAT420_ALERT_2_B D-H Without Acceptor O9 --H5 . Please Check
PLAT420_ALERT_2_B D-H Without Acceptor O10 --H10 . Please Check
PLAT420_ALERT_2_B D-H Without Acceptor O9 --H330 . Please Check
PLAT420_ALERT_2_B D-H Without Acceptor O10 --H331 . Please Check

THETM01_ALERT_3_C The value of sine(theta_max)/wavelength is less than 0.590
Calculated sin(theta_max)/wavelength = 0.5835
PLAT041_ALERT_1_C Calc. and Reported SumFormula Strings Differ Please Check
PLAT077_ALERT_4_C Unitcell Contains Non-integer Number of Atoms .. Please Check
PLAT082_ALERT_2_C High R1 Value .... 0.11 Report
PLAT084_ALERT_3_C High wR2 Value (i.e. > 0.25) .... 0.28 Report
PLAT202_ALERT_3_C Isotropic non-H Atoms in Anion/Solvent .... 21 Check
C183 C184 C185 C186 C187 C188 etc.
PLAT213_ALERT_2_C Atom C20 has ADP max/min Ratio ..... 3.6 oblate
PLAT213_ALERT_2_C Atom C70 has ADP max/min Ratio ..... 3.1 prolat
PLAT213_ALERT_2_C Atom C71 has ADP max/min Ratio ..... 3.7 prolat
PLAT213_ALERT_2_C Atom C72 has ADP max/min Ratio ..... 3.5 prolat
PLAT213_ALERT_2_C Atom C73 has ADP max/min Ratio ..... 3.1 prolat
PLAT213_ALERT_2_C Atom C79 has ADP max/min Ratio ..... 3.2 prolat
PLAT213_ALERT_2_C Atom C129 has ADP max/min Ratio ..... 3.2 oblate
PLAT213_ALERT_2_C Atom C158 has ADP max/min Ratio ..... 3.5 prolat
PLAT331_ALERT_2_C Small Aver Phenyl C-C Dist C190 -C234 . 1.36 Ang.
PLAT332_ALERT_2_C Large Phenyl C-C Range C183 -C188 . 0.19 Ang.
PLAT361_ALERT_2_C Long C(sp3)-C(sp3) Bond C79 - C81 .. 1.67 Ang.
PLAT361_ALERT_2_C Long C(sp3)-C(sp3) Bond C164 - C167 .. 1.65 Ang.

FORMU01_ALERT_2_G There is a discrepancy between the atom counts in the
_chemical_formula_sum and the formula from the _atom_site* data.
Atom count from _chemical_formula_sum:C213.87 H122.39 Cl3.22 N4.9 O9.2
Atom count from the _atom_site data: C213.8719 H122.4060 Cl3.222 N4.9
CELLZ01_ALERT_1_G Difference between formula and atom_site contents detected.
CELLZ01_ALERT_1_G ALERT: check formula stoichiometry or atom site occupancies.
From the CIF: _cell_formula_units_Z 4
From the CIF: _chemical_formula_sum C213.87 H122.39 Cl3.22 N4.90 O9.20
TEST: Compare cell contents of formula and atom_site data
atom Z*formula cif sites diff
C 855.48 855.49 -0.01
H 489.56 489.62 -0.06
Cl 12.88 12.89 -0.01
N 19.60 19.60 0.00
O 36.80 36.84 -0.04
P 8.00 8.00 0.00
S 8.00 8.00 0.00

PLAT045_ALERT_1_G Calculated and Reported Z Differ by a Factor ... 2.00 Check
PLAT066_ALERT_1_G Predicted and Reported Tmin&Tmax Range Identical ? Check
PLAT068_ALERT_1_G Reported F000 Differs from Calcd (or Missing)... Please Check
PLAT083_ALERT_2_G SHELXL Second Parameter in WGHT Unusually Large 130.15 Why ?
PLAT333_ALERT_2_G Large Aver C6-Ring C-C Dist C11 -C31 . 1.43 Ang.
PLAT779_ALERT_4_G Suspect or Irrelevant (Bond) Angle(s) in CIF . # 859 Check
N5 -N6 -H5 1.555 1.555 1.555 20.00 Deg.
PLAT779_ALERT_4_G Suspect or Irrelevant (Bond) Angle(s) in CIF . # 861 Check
N8 -N7 -H10 1.555 1.555 1.555 28.00 Deg.
PLAT789_ALERT_4_G Atoms with Negative _atom_site_disorder_group # 75 Check
PLAT899_ALERT_4_G SHELXL97 is Deprecated and Succeeded by SHELXL 2018 Note

The refinement should be carried with the latest version of SHELXL (SHELXL_2018). Atoms with unusual ADP max/min ratios should have been restrained to near isotropic values with the ISOR instruction. As for the atoms that were refined with isotropic displacement parameters, it should be possible to refine the atoms with anisotropic displacement parameters but, if necessary, using ISOR instructions to restrain the displacement parameters to near isotropic values. As the disorder of the solvent is extensive, the refinement could have used the OLEX2 to enable the use of the solvent mask routine to account for some of the disordered solvent, or the data processed with the SQUEEZE routine of Platon. With no embedded HKL data and RES file in the CIF file it is not possible to be certain of how much this would improve the refinement. In view of the uncertainties in the refinement of the solvent, the solvent composition, (C6H5CH3)6.114•(CHCl3)1.074 , could be rounded off to (C6H5CH3)6•(CHCl3) . How certain are the authors that there is nitrogen in the cavity, rather than the water molecule disordered over 3 positions? The electron density for both cavities sums up to 8 (excluding the H-atoms). The availability of the intensity data and RES file being available would help in verifying this.

Page 14, line 3: Is this number 198945 correct? Should it be 1988945?

As SHEXL, OLEX2 and PLATON are free, there is no reason not to use the latest versions of these programs. OLEX2 and PLATON can determine the amount of solvent (electron density) in solvent cavities and assist in solvent composition even if the disorder is able to be modelled.

Reviewer #3 (Remarks to the Author):

In this manuscript, the authors studied the reaction of the conjugated macrocycle on an open-cage fullerene derivative with phosphines, and found that betaine and ylide derivatives were kinetic and thermodynamic products, respectively, based on the experimental and calculation results. The previously elusive zwitterionic forms of betaine intermediates in the phospha-Michael addition and β-oxo-phosphorus ylides from alpha-deketones were confirmed by the single crystal X-ray analysis. A plausible mechanism involving an SN2 instead of a carbene intermediate toward the formation of β-oxo-phosphorus ylides was proposed based on the calculation results. This reviewer recommends the acceptance of this manuscript after revisions.

1) A short descriptions of 1′ and 2′ in the main text and SI should be provided for the readers to better understand what they are.
2) The synthesis of closely related fullerene derivatives with phosphorus addends should be described and cited, e.g., Tetrahedron 1996, 52, 5091; Tetrahedron Lett. 1998, 39, 9233; Org. Lett. 2005, 7, 5633; J. Org. Chem. 2009, 74, 4866; Chem. Commun. 2011, 47, 6111; J. Org. Chem. 2011, 76, 6088.

To Reviewer 1

Reviewer 1's Comments

The manuscript reports the reaction between an open-cage fullerene and phosphines. One of the products is an unstable phospha-Michael adduct and the other one is a phosphorus ylide. The results are important for the modification of open-cage fullerenes. But the reaction pattern and structure of the new compounds are the same as those in well established classical organic chemistry. The work is too specific and unlikely to attract the attention of general readers of nature communication chemistry.

Our Response

We express our gratitude to the Reviewer 1 for the evaluation and suggestions. As Reviewer 1 suggested, the structural modification using phosphonium substituents was demonstrated to be important to drastically change the electronic structure of the C_{60}-based spherical polycyclic aromatic hydrocarbon. Even though the reaction pattern and structure are the same as those reported for non-π-conjugated molecules, isolation of the synthetically and biologically important intermediate as well as clarification of the reaction mechanism have scientifically significant meaning so that we can switch or tune the electronic structure of largely π-conjugated materials by embedding phosphonium units. Thereby, we believe that our findings will attract attention from a variety of field, not confined in the fullerene research.

<u>To Reviewer 2</u>

We appreciate to the high evaluation and comments from Reviewer 2. We carefully considered all suggestions from the Reviewer 2 and responses were summarized below.

Reviewer 2's Comments

This is an interesting manuscript worthy of publication. However, there are significant problems that require the manuscript to be revised before being accepted for publication

The title: "Organophosphorus zwitterions engaged in a conjugated macrocycle on fullerene" doe not seem to be very accurate, as there does not seem to be a macrocycle present in the molecules. Maybe "The synthesis and characterization of organophosphorus zwitterions attached to the rim of open-cage substituted fullerenes."

Our Response #1: Thank you for kindly proposing a candidate title of this manuscript. The term "macrocycle" has been recognized as the rim of open-cage fullerene derivatives as can be found in papers such as: Gan, L. et al., [60]Fullerene-Based Macrocycle Ligands, *Chem. Eur. J.* **23**, 10485 (2017). Additionally, IUPAC Gold Book defines the term "macrocycle" as "a cyclic macromolecular or a macromolecular cyclic portion of a macromolecule" (*Pure Appl. Chem.* **68**, 2287 (1996)). Based on references above, the rim of the open-cage fullerene derivatives could be regarded as a macrocycle. Therefore, we would like to keep the title as is.

Page 4, line 14: "Since 1 spontaneously encapsulates a water molecule inside its cage14, the 31P signal of H2O@2 (δ +37.3 ppm) was also observed." This repeats the previous sentence, the included water molecule would have little impact of the P signal. So, delete or rewrite.

Our Response #2: Depending on the type of encapsulated molecules, the exohedoral substitution has different chemical shift perturbation. Historically in the fullerene chemistry, such change in chemical shift has been utilized as an important measure of the intramolecular interaction between the encapsulated molecules and the fullerene cage. According to Reviewer 2's suggestion, the sentences were reconsidered and revised as follows:

~~Since **1** spontaneously encapsulates a water molecule inside its cage[14], the ^{31}P signal of H_2O@**2** (δ +37.3 ppm) was also observed.~~ The ^{31}P signal observed at δ +37.3 ppm corresponds to H_2O@**2** formed via spontaneous encapsulation of a water molecule.

Page 7: From the C-O bond lengths, it looks like the negative charges are partly localized on O3 (1pph_c) and O1 & O3 (2Pint_b). The authors should comment on this

Our Response #3: In the original manuscript (P. 7) and Supplementary Information (P. S117), we had mentioned the partial delocalization of the negative charge on the O atoms in both compounds as suggested by Reviewer 2. We showed again the corresponding descriptions from page 7 on the original manuscript:

"From the bond lengths, the structure of **2** can be rationalized by describing a resonance

hybrid consisting of 1-phosphonium-5-oxabetaine and 1-phosphonium-3-carbabetaine as major contributing forms (Fig. 2c)."
"This is suggestive of the charge delocalization along with C–C–O and other C_{60} moieties (Fig. 2c), being consistent with the natural population and Wiberg bond order analyses (Supplementary Fig. 52)."

Page 7: "The driving force for this self-assembly should be attributed to the intermolecular electrostatic attraction between +PMe3 and C–O– moieties and thus the reactive sites (phosphonium betaine) were fully covered with the four fullerene cages in total." Specify the P...O distances. Are the atoms involved in these interactions P...O1 & P...O3 (as suggested above based on C-O bond lengths)? The authors should comment on this.

Our Response #4: We added the distance of P•••O in the manuscript on page 7. Since the observed P•••O distances of 3.646(4) and 3.668(4) Å are close to the sum of van der Waals radii of P and O atoms (3.32 Å estimated from values in Bondi, A. *J. Phys. Chem.* **68**, 441 (1964)), they have a strong electrostatic interaction.

"The driving force for this self-assembly should be attributed to the intermolecular electrostatic attraction between $^{+}PMe_3$ and $C–O^{-}$ moieties (3.646(4) and 3.668(4) Å for P•••O) and thus the reactive sites (phosphonium betaine) were fully covered with the four fullerene cages in total."

Page 7: "The X-ray diffraction analysis revealed that an asymmetric unit accommodates a racemate including two enantiomeric isomers fC-2 and fA-2 arranged as two crystallographically independent molecules (nomenclature based on criteria stated by Diederich20,21). Interestingly, a unit of the racemate is assembled with another one as a tetrahedral configuration via heterochiral recognition" Are there any significant differences between the two crystallographically unique enantiomers? How distorted is the tetrahedron? What are the intermolecular interactions? Is there a refrerence?

Our Response #5: There is negligible structural differences between the two enantiomers. As shown in Fig. 2, totally four molecules are assembled in a unit cell in a tetrahedral manner. Other views of assembled structures were shown below. The observed chiral recognition would be facilitated by electrostatic interaction between $^{+}PMe_3$ and $C–O^{-}$ moieties. To the best of our knowledge, this is the first report on the heterochiral self-assembly of open-cage fullerene derivatives.

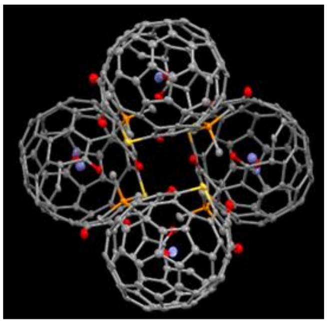

Top view

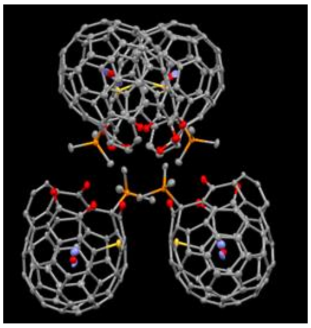

side view

Page 12: “Note that all the compounds shown herein encapsulate a water molecule inside their cages with an occupation level of 10–70% at room temperature.” How was this determined for each compound? From the two crystal structures it looks like the cavity is fully occupied (or one fully occupied with the other maybe). There is no information on the water content for the four other structures. There are no chemical analyses for any of the compounds. What about the possibility of nitrogen?

Our Response #6: The encapsulation ratio of water can be accurately determined by ^{1}H NMR analysis since the encapsulated water molecule shows its chemical shift at δ ca. –10 ppm, reflecting the shielding effect from the surrounding spherical π-surface. Once other chemical species encapsulated inside the fullerene cage, chemical shifts of the exohedoral substituent would be splitted and therefore other possibilities can be excluded as responded at Our Response #2. During the XRD measurement of the single crystals with keeping the constant temperature of 100 K under N_2 flow, the encapsulated water molecule is usually replaced with N_2 and thus crystal structure has high encapsulation ratio of N_2. All of required characterization data and spectra had been already shown in Supplementary Information (Page S5–32). Due to the word count limitation, these data could not be shown in the main text (For example, since **3a** encapsulates water molecule, we usually get a mixture of **3a** and H_2O@**3a** which should show 158 ^{13}C signals). The purity and structural characterization of all compounds are guaranteed by the given spectra in Supplementary Information.

Page 12: “conjugated macrocycle on 1” replace by “open-cage fullerene”

Our Response #7: We would like to keep as it is, as responded in Our Response #1.

Synthesis:
There is no chemical analysis.

Our Response #8: As responded in Our Response #6, due to the word count limitation, these data could not be shown in the main text (For example, since **3a** encapsulates water molecule, we usually get a mixture of **3a** and H_2O@**3a** which should show 158 ^{13}C signals). The purity and structural characterization of all compounds are guaranteed by the given spectra in Supplementary Information.

General:
The terminology “H2O@2” replace by something like “2.H2O”
The English language expression needs some minor improvement.

Our Response #9: We would like to keep as it is since this nomenclature has been accepted nearly 30 years and currently this nomenclature can be seen elsewhere in the field of host-guest chemistry. Historically, this nomenclature was advocated in 1991 by Smalley (*J. Phys. Chem.* **95**, 7564 (1991)) who is the Nobel awardee.

Crystallography:

1pph_c:
The structure seems to have been carried out reasonably well. The dichloromethane could have been dealt with using the OLEX2 solvent mask routine. There are a number of issues in the checkCIF report that should have been addressed in the refinement.

The refinement should be carried with the latest version of SHELXL (SHELXL_2018). Atoms with unusual ADP max/min ratios should have been restrained to near isotropic values with the ISOR instruction. The large electron density peak is presumably associated with the solvent. As the disorder of the solvent is extensive, the refinement should have used OLEX2 to enable the use of the solvent mask routine to account for the disordered solvent, or the data processed with the SQUEEZE routine of Platon. With no embedded HKL data and RES file in the CIF file it is not possible to be certain of how much this would improve the refinement.

> **Our Response #10:** The refinement was redone using the latest version using SHELXL_2018. Atoms with unusual ADP max/min ratios appeared as Alert B were restrained using the ISOR instruction. In case of large molecules like our compounds, the severely disordered solvent molecules are commonly found in the crystal structure which potentially increases the R_1 and wR_2 factors whereas the non-disordered main structures can be reasonably discussed. To avoid any artificial process that might be caused by SQUEEZE as small as possible, we solved structures by using appropriate solvent disordered models. Now, we prepared the fcf-embedded cif files so that the reviewers can access them. The new cif files were re-registered with keeping the same deposition numbers.

2Pint_b:
The refinement should be carried with the latest version of SHELXL (SHELXL_2018). Atoms with unusual ADP max/min ratios should have been restrained to near isotropic values with the ISOR instruction. As for the atoms that were refined with isotropic displacement parameters, it should be possible to refine the atoms with anisotropic displacement parameters but, if necessary, using ISOR instructions to restrain the displacement parameters to near isotropic values. As the disorder of the solvent is extensive, the refinement could have used the OLEX2 to enable the use of the solvent mask routine to account for some of the disordered solvent, or the data processed with the SQUEEZE routine of Platon. With no embedded HKL data and RES file in the CIF file it is not possible to be certain of how much this would improve the refinement. In view of the uncertainties in the refinement of the solvent, the solvent composition, (C6H5CH3)6.114•(CHCl3)1.074 , could be rounded off to (C6H5CH3)6•(CHCl3) . How certain are the authors that there is nitrogen in the cavity, rather than the water molecule disordered over 3 positions? The electron density for both cavities sums up to 8 (excluding the H-atoms). The availability of the intensity data and RES file being available would help in verifying this.

> **Our Response #11:** As responded at Our Response #10, the refinement was redone using the latest version using SHELXL_2018. Atoms with unusual ADP max/min ratios appeared as Alert B were restrained using the ISOR instruction. In case of large molecules like our compounds, the severely disordered solvent molecules are commonly found in the crystal structure which potentially increases the R_1 and wR_2 factors whereas the non-disordered main structures can be reasonably discussed. To

avoid any artificial process that might be caused by SQUEEZE as small as possible, we solved structures by using appropriate solvent disordered models. Now, we prepared the fcf-embedded cif files so that the reviewers can access them. The new cif files were re-registered with keeping the same deposition numbers.

By the refinement, we had found that the components of the solvent molecules are not natural numbers. We do not agree with the Reviewer 2's suggestion to round off them into natural numbers. We would like to show them with three significant figures.

From "$(C_6H_5CH_3)_{6.114}$•$(CHCl_3)_{1.074}$" to "$(C_6H_5CH_3)_{6.11}$•$(CHCl_3)_{1.07}$"

Since the cavity size of fullerene C_{60} is limited, it is generally impossible for the water molecule to geometrically move around inside the cage. Only if the cavity is largely expanded to have similar cavity size of C_{70}, the positional disorder suggested by Reviewer 2 can be observed. So far, such positional disorder was observed only one example (*Chem*. *Commun*. **54**, 13686 (2018)). Therefore, it is hard to consider the positional disorder of three water molecules. The disordered encapsulated molecules such as N_2/H_2O are commonly observed for the open-cage fullerene derivatives (*Org*. *Lett*. **15**, 2750 (2013)).

Page 14, line 3: Is this number 198945 correct? Should it be 1988945?

Our Response #12: Thank you for suggestion. We corrected typos on Page 14.

"CCDC 1988944 (**3d**) and 1988945 (**2**) contain the supplementary crystallographic data for this paper."

As SHEXL, OLEX2 and PLATON are free, there is no reason not to use the latest versions of these programs. OLEX2 and PLATON can determine the amount of solvent (electron density) in solvent cavities and assist in solvent composition even if the disorder is able to be modelled.

Our Response #13: As responded at Our Response #10 and #11, the refinement was redone using the latest version using SHELXL_2018. Now, we prepared the fcf-embedded cif files so that the reviewers can access them. The new cif files were re-registered with keeping the same deposition numbers.

<u>To Reviewer 3</u>

We express our gratitude to the Reviewer 3 for the evaluation and comments. We have made revision on our manuscript based on the Reviewer 3's comments and listed below.

Reviewer 3's Comments

In this manuscript, the authors studied the reaction of the conjugated macrocycle on an open-cage fullerene derivative with phosphines, and found that betaine and ylide derivatives were kinetic and thermodynamic products, respectively, based on the experimental and calculation results. The previously elusive zwitterionic forms of betaine intermediates in the phospha-Michael addition and β-oxo-phosphorus ylides from alpha-deketones were confirmed by the single crystal X-ray analysis. A plausible mechanism involving an SN2 instead of a carbene intermediate toward the formation of β-oxo-phosphorus ylides was proposed based on the calculation results. This reviewer recommends the acceptance of this manuscript after revisions.

1) A short descriptions of 1′ and 2′ in the main text and SI should be provided for the readers to better understand what they are.

Our Response #1: According to the comment, we added short descriptions of compounds.

Page 4. "As a conjugate macrocyclic orifice on the C_{60} cage, we made a choice of open-cage derivative **1** having α,β-unsaturated carbonyl and 1,2-dicarbonyl moieties (Fig. 1a)[14]."
Page 5. "the energy required for forming a betaine derivative is rather small for **2'** (Ar = 2-pyridyl) obtained by the reaction at C(2)"
Page 8. "**Mechanism.** To verify the possible pathway for providing **3a** from **1** and PMe_3, we performed DFT calculations (M06-2X/6-31G(d,p), ~~Ar = 2-pyridyl,~~ 298 K) (Fig. 3). The first step is the nucleophilic attack of PMe_3 to the carbonyl group in **1'** (Ar = 2-pyridyl) to give **INT1** via **TS1**"
Page S1. "Open-cage tetraketo C_{60} derivative **1** was synthesized according to a literature[1]."

2) The synthesis of closely related fullerene derivatives with phosphorus addends should be described and cited, e.g., Tetrahedron 1996, 52, 5091; Tetrahedron Lett. 1998, 39, 9233; Org. Lett. 2005, 7, 5633; J. Org. Chem. 2009, 74, 4866; Chem. Commun. 2011, 47, 6111; J. Org. Chem. 2011, 76, 6088.

Our Response #2: Thank you for suggesting us to cite related papers. Among suggested ones, we have already cite the first repot on phosphine-substituted C_{60} derivative (Yamago, S. et al., *Tetrahedron* **52**, 5091 (1996)). In addition, we decided to cite two more papers which reported synthetically different approaches.

14. Chen, S.-Y. et al., Fullerene derivatives incorporating phosphoramidous ylide and phosphoramidate: synthesis and property. *J. Org. Chem.* **74**, 4866–4869 (2009).
15. Wang, G.-W., Wang, C.-Z., Zhu, S.-E. & Murata, Y. Manganese(III) acetate-mediated radical reaction of [60]fullerene with phosphonate esters affording unprecedented separable singly-bonded [60]fullerene dimers. *Chem. Commun.* **47**, 6111–6113 (2011).

REVIEWERS' COMMENTS:

Reviewer #1 (Remarks to the Author):

I am sorry that my opinion remains the same. i.e. The work is too specific and unlikely to attract the attention of general readers of nature communication chemistry.

Reviewer #2 (Remarks to the Author):

Editor's note: this reviewer offered no further remarks to the authors.

Reviewer #3 (Remarks to the Author):

The revised manuscript has addressed this reviewer’s concerns, and can be considered for publication.

To Reviewer 1

We would like to express our gratitude to the Reviewer 1 for giving us the comment. The organophosphorus zwitterions are of particular importance for a variety of field including biology and synthetic chemistry. The significance of our report is the demonstration that we could obtain the elusive intermediates, as well as elucidation of the reaction mechanism that has long been disputed. Thus, we believe that our findings will attract attention from general readers.

Reviewer 1's Comments

I am sorry that my opinion remains the same. i.e. The work is too specific and unlikely to attract the attention of general readers of nature communication chemistry.

To Reviewer 2

We appreciate to Reviewer 2 for the acceptance of our revised manuscript.

Reviewer 2's Comments

Editor's note: this reviewer offered no further remarks to the authors.

To Reviewer 3

We are grateful to Reviewer 3 for his/her evaluation on our manuscript for publication.

Reviewer 3's Comments

The revised manuscript has addressed this reviewer's concerns, and can be considered for publication.
